# Heterogeneity in potential impact and cost-effectiveness of ETEC and *Shigella* vaccination in four sub-Saharan African countries

**DOI:** 10.1016/j.jvacx.2019.100043

**Published:** 2019-09-20

**Authors:** John D. Anderson, Farzana Muhib, Richard Rheingans, Thomas Wierzba

**Affiliations:** a Goodnight Family Department of Sustainable Development, Appalachian State University, 222 Living Learning Center, 305 Bodenheimer Drive, Boone, NC 28608, USA; b PATH, 455 Massachusetts Ave. NW, Suite 1000, Washington, DC 20001, USA

**Keywords:** ETEC, *Shigella*, Sub-Saharan Africa, Disparities, Cost-effectiveness analysis, Subnational, Diarrhoea, Stunting

## Abstract

Diarrheal disease burden has become more heterogenous in low- and lower middle-income countries as access to clean water, sanitation and health care has increased in wealthier urban populations. Enterotoxigenic Escherichia coli (ETEC) and *Shigella* are among the top five causes of diarrheal mortality in children living in sub-Saharan Africa.

Here, we explored how accounting for subnational and economic heterogeneity in ETEC and *Shigella* disease burden affects projected vaccine impact and cost-effectiveness of standalone ETEC and *Shigella* vaccines during the first decade after introduction in four sub-Saharan African countries. We developed dynamic models for provincial areas and socioeconomic subpopulations of children in the Democratic Republic of Congo (DRC), Kenya, Zambia, and Zimbabwe. We estimated deaths and morbidity due to ETEC and *Shigella* diarrhea plus additional deaths from other infectious diseases attributable to ETEC- and *Shigella*-induced stunting. We analyzed cost-effectiveness using Incremental Cost-Effectiveness Ratios (ICERs) with Disability-Adjusted Life Years (DALYs) and Moderate-and-Severe Diarrheal episodes (MSD) averted as denominators.

Other infectious disease deaths due to induced stunting accounted for 9–28% and 9–32% of the total provincial level ETEC and *Shigella* disease burden, respectively, across these four countries from years 2025 to 2034. Our results indicated that the lowest and most cost-effective provincial DALYs averted ICERs were below $600 and $500/DALY averted for ETEC and *Shigella* vaccination, respectively in Zimbabwe. ICERs were the highest in Zambia and Kenya, where all provincial ICERs where above $2000/DALY. The highest national and provincial MSD averted ICERs were in DRC, while the lowest were in Kenya and Zimbabwe. Vaccinations were most cost-effective in averting DALYs in lower wealth subpopulations living in the highest burden provincial areas.

Our approach focused on subnational heterogeneity in ETEC and *Shigella* burden and vaccination access found that impact and cost-effectiveness were more favorable if vaccinations reach the most vulnerable children in underserved provinces.

## 1 Introduction

While diarrheal mortality has declined 34% worldwide, it is still one of the leading causes of mortality for children under five [1]. However, diarrheal morbidity has declined at a much slower rate, only 10% from 2005 to 15 [1]. The majority of diarrheal disease burden is concentrated in underserved populations in low- and middle-income countries (LMICs) lacking access to maternal and child health services, safe water supply or sanitation facilities [2], [3]. Enterotoxigenic *Escherichia coli* (ETEC) and *Shigella* are two of the leading enteropathogens causing diarrheal disease burden in children under 5 years of age globally and in sub-Saharan Africa [1], [4].

Diarrheal disease severity varies by pathogen, causing a variety of symptoms beyond loose or watery stools that may result in long-term sequalae, such as childhood stunting [5], [6], defined as height-for-age z-scores (HAZ) less than two standard deviations below median WHO standards [7]. A key finding from the Global Enteric Multicenter Study (GEMS) [4] was that childhood episodes of moderate-to-severe diarrhea (MSD) were associated with negative shifts in average in HAZ scores in populations of children. Recent studies of ETEC and *Shigella* disease burden in low- and low-middle income countries estimated the increased risk of death from other infectious diseases due to diarrhea-induced stunting and found that mortality estimates increased by 24% (ETEC) and 28% (*Shigella*) over estimates of direct deaths due to these pathogens [8], [9]. Another study of global diarrheal disease burden that included long-term sequalae and other infectious diseases deaths from diarrhea-induced stunting found that DALY burden increased 39% over acute diarrheal disease burden [10].

A recent study found that ETEC vaccination was most cost-effective for children living in countries grouped into the World Health Organization (WHO) Regional Office for Africa, which included the four countries in this analysis: Kenya, the Democratic Republic of Congo (DRC), Zambia, and Zimbabwe [11]. In the same study, *Shigella* vaccination was also found to be cost-effective in Regional Office for Africa (AFRO) countries as compared to other countries but was most cost effective in Regional Office for the Eastern Mediterranean (EMRO) countries. However, national estimates of ETEC and *Shigella* vaccine impact and cost-effectiveness were estimated without considering disease heterogeneity at the sub-national level. Previous rotavirus vaccination impact studies suggest diarrheal mortality risk is heterogenous within countries due to disparities in exposure (e.g., access to safe or improved water and sanitation) and susceptibility (e.g., access to healthcare, nutritional status) across geographic and socioeconomic subpopulations [12], [13], [14].

Vaccines for ETEC and *Shigella* are currently under development and could be critical interventions to reduce geographic and socioeconomic disparities in disease burden. The United Nations’ Sustainable Development Goals (SDGs) have prioritized addressing disparities by setting goals to provide universal and equitable access to healthcare and vaccines (SDG3). Improving equity in health outcomes requires models that produce estimates at levels that can optimize intervention delivery by reaching the most vulnerable children within countries [15]. Here, our objective was to estimate impact and cost-effectiveness of introducing ETEC and *Shigella* standalone vaccines in geographic and socioeconomic subpopulations within four sub-Saharan African countries with high estimated ETEC and *Shigella* burden [16]. We explore how heterogeneity in burden and delivery result in more refined impact and cost-effectiveness estimates, which may inform implementation strategies to better address related inequalities in these countries.

## 2 Methods

We developed dynamic subnational models projecting the impact and cost-effectiveness of ETEC and *Shigella* vaccines across different geographic areas and socioeconomic subpopulations of children in DRC, Kenya, Zambia, and Zimbabwe. We estimated deaths and morbidity due to ETEC and *Shigella* diarrhea as well as burden from other infectious disease deaths attributable to ETEC- and *Shigella*-induced stunting using methodology [9] that assumed moderately-to-severely stunted (MSS) children have higher risk of mortality due to other infectious diseases [17], [18].

### 2.1 Geographic and socioeconomic subpopulations

**Fig. 1:**
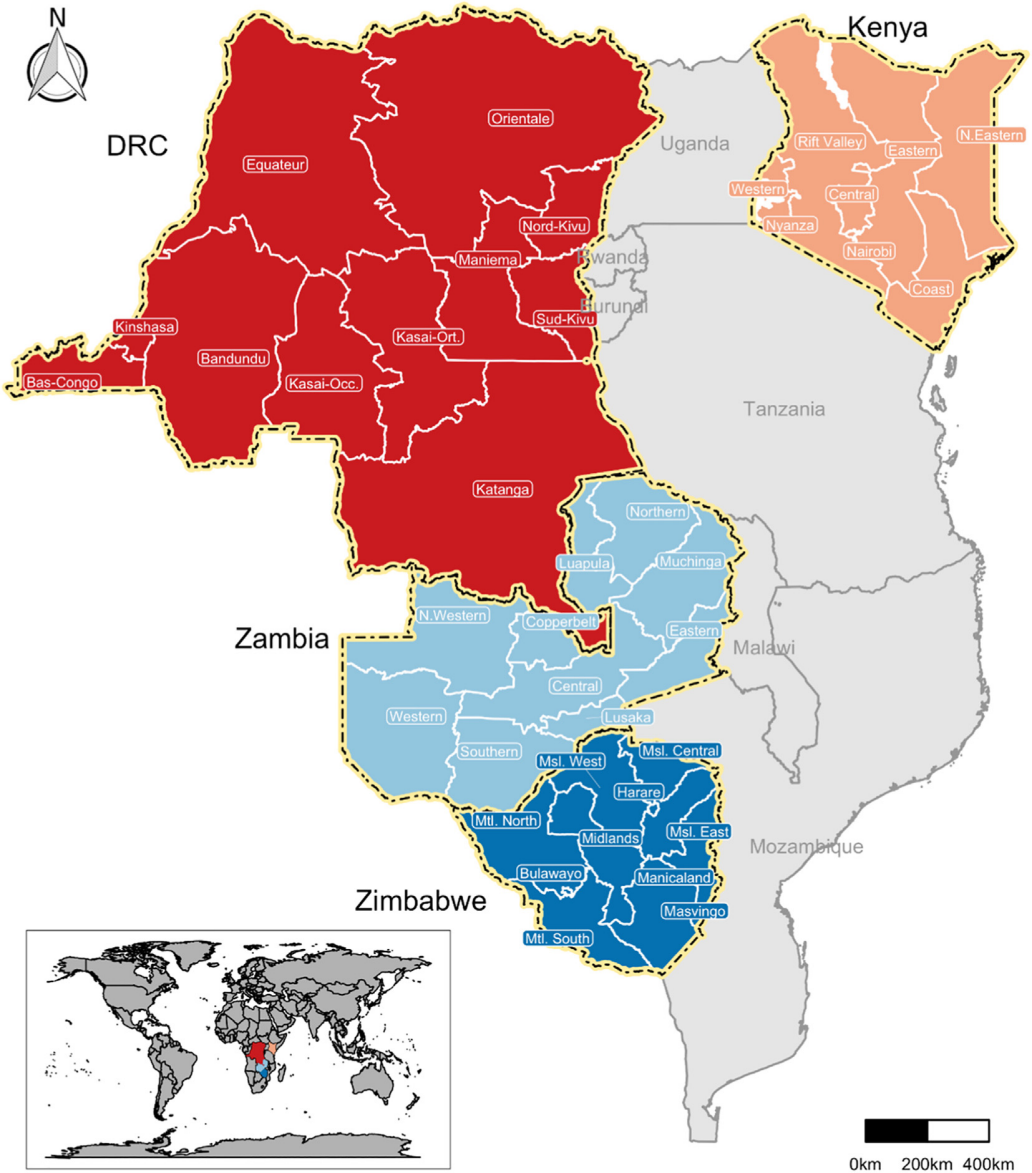
Mmap of the four sub-Saharan African study countries and provinces. DRC: Democratic Republic of Congo.

Malaria Atlas Project subnational estimates were used to project 2025–2034 populations in each country [19]. In the case of Kenya and DRC, we aggregated Demographic and Health Surveys (DHS) data to previous provinces to correspond to DHS administrative levels, since each country has recently changed their first-level administration units. Thus, for all countries the subnational geographic unit of analysis were provinces (Fig. 1). We modeled socioeconomic subpopulations by grouping individuals from zero to four years of age (children) into five wealth quintiles within each provincial area. We created wealth quintiles based on household asset index scores [20] with water and sanitation assets excluded to avoid confounding with exposure risk variables [21].

### 2.2 ETEC and *Shigella* etiological fractions

We used previously published methods to estimate the etiological fractions of diarrheal burden due to ETEC and *Shigella*. Briefly, we used bacterial culture-based estimates for AFRO countries [22] and transformed them using adjustment factors of 1·5 (50% increase) for ETEC and 2·0 (100% increase) for *Shigella*, consistent with the molecular-based re-analysis of GEMS data [23]. We assumed that etiological fractions were uniform within countries and remained static over time.

### 2.3 Diarrheal mortality and morbidity

We estimated diarrheal mortality for each country by using the mid-point of two diarrheal mortality estimates; one from the Institute for Health Measurement and Evaluation (IHME) [24] and the other from Maternal and Child Epidemiology Estimation Group (MCEE) [25] (Table 1). We used published annual regional diarrhea incidence rates [26] to produce national estimates of morbidity. We projected diarrheal mortality from 2015 to 2034 assuming continued trends in estimates of mortality from IHME and MCEE between 2000 and 2010 to account for pre-rotavirus vaccine mortality rates (Table 1). We projected diarrheal morbidity from 2015 to 2034 using estimated Years Lived with Disability (YLD) from diarrheal diseases between 2000 and 2010 from IHME [24].

**Table 1:** Model parameters for vaccine cost-effectiveness and impact analyses of ETEC and *Shigella* vaccines. All costs are presented in 2016 US$. *Values for the base case scenarios. **Range of values used in sensitivity analysis. ‘NA’ indicates parameters that were not varied in sensitivity or uncertainty analyses.

Model input	Values*	Uncertainty distribution	Sensitivity range**	Reference
**Mortality and morbidity**				
Population estimates	Varies by country and administration unit	NA	NA	[19]
Under-5 diarrheal mortality (deaths)		Lognormal (SD)		
DRC	25,840	7655	± 10%	[24], [25]
Kenya	5737	695	± 10%	[24], [25]
Zambia	2797	695	± 10%	[24], [25]
Zimbabwe	3696	680	± 10%	[24], [25]
Annual change in non-rotavirus under-5 diarrheal mortality rate		Triangular (min, max)		
DRC	−0.017	−0.021, −0.013	± 25%	[24], [25]
Kenya	−0.032	−0.040, −0.024	± 25%	[24], [25]
Zambia	−0.054	−0.068, −0.041	± 25%	[24], [25]
Zimbabwe	0.052	0.039, 0.065	± 25%	[24], [25]
Under-5 diarrheal episodes per child-year	3.3	Lognormal (SD = 0.9)	± 10%	[26]
Annual change in under-5 diarrheal episode rate				
DRC	−0.012	NA	NA	[24]
Kenya	−0.003	NA	NA	[24]
Zambia	−0.005	NA	NA	[24]
Zimbabwe	0.005	NA	NA	[24]
Etiological fraction attributed to ETEC for AFRO region	0.075	Beta (alpha = 100.7, beta = 1231.1)	± 25%	[22], [23]
Etiological fraction attributed to *Shigella* for AFRO region	0.082	Beta (alpha = 101.5, beta = 1126.3)	± 25%	[22], [23]
Stunting induced by ETEC episodes (shift in HAZ)	0.068	Beta (alpha = 156.7, beta = 1231.1)	± 50%	[4]
Stunting induced by *Shigella* episodes (shift in HAZ)	0.082	Beta (alpha = 101.5, beta = 2137.8)	± 50%	[4]
Fraction of diarrheal episodes that are moderate to severe	0.10	Beta (alpha = 21.2, beta = 182.8)	0.02 – 0.15	[4], [9], [52]
Age-adjusted and discounted DALYs per child death	34	NA	NA	[53]
Watery diarrhea disability weight	0.067	NA	NA	[40]
Duration of diarrhea (years)	0.02	NA	NA	[40]

**Diarrheal risk index**				
*Susceptibility risk factors*				
Oral rehydration treatment	93% effective	NA	NA	[32]
Underweight (weight-for-age)	Relative risk: 1–12.5	NA	NA	[18]
Vitamin A supplement	30% effective	NA	NA	[33]
*Exposure risk factors*				
Drinking water access (relative risk)				
Unimproved	1.00	NA	NA	[34]
Off-plot improved	0.89	NA	NA	[34]
On-plot improved	0.77	NA	NA	[34]
Sanitation access (relative risk)				
No, unimproved or shared	1.00	NA	NA	[34]
Improved and unshared	0.84	NA	NA	[34]
Improved sewered house connection	0.31	NA	NA	[34]

**Vaccination and medical costs**				
ETEC or *Shigella* vaccine efficacy	60%	Beta (alpha = 22.6, beta = 15.4)	± 20%	Assumption
Dose price (2016 US$)	$3.33	Lognormal; SD = 0.91	$2.00-$7.00	Assumption
Administration cost (2016 US$)	$1.93	Lognormal; SD = 0.22	± 40%	[38]
Outpatient cost of ETEC or *Shigella* illness (2016 US$/episode)		Lognormal (SD)		
DRC	$4.81	0.29	± 40%	[54], [55], [56], [57], [58], [59], [60], [61], [62], [63], [64], [65], [66], [67], [68], [69]
Kenya	$5.31	0.86	± 40%	[54], [55], [56], [57], [58], [59], [60], [61], [62], [63], [64], [65], [66], [67], [68], [69]
Zambia	$5.47	0.88	± 40%	[54], [55], [56], [57], [58], [59], [60], [61], [62], [63], [64], [65], [66], [67], [68], [69]
Zimbabwe	$4.81	0.34	± 40%	[54], [55], [56], [57], [58], [59], [60], [61], [62], [63], [64], [65], [66], [67], [68], [69]
Inpatient cost of ETEC or *Shigella* illness (2016 US$/episode)		Lognormal (SD)		
DRC	$24.24	5.41	± 40%	[54], [55], [56], [57], [58], [59], [60], [61], [62], [63], [64], [65], [66], [67], [68], [69]
Kenya	$33.42	12.07	± 40%	[54], [55], [56], [57], [58], [59], [60], [61], [62], [63], [64], [65], [66], [67], [68], [69]
Zambia	$34.34	12.40	± 40%	[54], [55], [56], [57], [58], [59], [60], [61], [62], [63], [64], [65], [66], [67], [68], [69]
Zimbabwe	$23.77	7.55	± 40%	[54], [55], [56], [57], [58], [59], [60], [61], [62], [63], [64], [65], [66], [67], [68], [69]
Inpatient visit rate (percentage of outpatient visits)	12.50%	Beta (alpha = 26.9, beta = 182.2)	± 50%	[36]
Outpatient visit rate (cases taken to healthcare facility)				
DRC	39%	NA	NA	[52]
Kenya	58%	NA	NA	[52]
Zambia	66%	NA	NA	[52]
Zimbabwe	39%	NA	NA	[52]

### 2.4 Distribution of mortality and morbidity

We assumed mortality risk from ETEC and *Shigella* was heterogeneous across economic and geographic subpopulations. We attributed mortality to each subpopulation using aggregated individual child mortality risk index scores ($R_{i}$) [8], [27]. We calculated $R_{i}$ as the product of relative risks associated with factors related to individual exposure ($E_{i}$) to enteropathogens and susceptibility ($S_{i}$) to diarrheal disease mortality (Eq. (1.1)). We calculated prevalence data for each risk factor using DHS estimates (Table 1) [28], [29], [30], [31].

(1.1) $$R_{i}=S_{i}\cdot E_{i}$$

(1.2) $$S_{i}=\prod_{k}\sum_{k=1}^{n}{(s}_{j,k}F_{i,j,k})$$

(1.3) $$E_{i}=\prod_{k}\sum_{k=1}^{n}{(e}_{j,k}G_{i,j,k})$$

Our susceptibility index ($S_{i}$) was designed to be proportional to case-fatality rates based on three known risk factors (n) for diarrheal mortality: utilization of oral rehydration therapy (ORT) [32], nutritional status (weight-for-age, [WFA]) [18], and receiving vitamin A supplements [33] (Table 1). As shown in Eq. (1.2), $S_{i}$ is the product of the relative risk (s) associated with the *j*th level of susceptibility risk factor *k* and the status of each risk factor (F) for individual (i). As only a small subset of children in DHS experienced diarrhea two weeks prior to the survey, we used a logistic regression analysis with relevant household and demographic predictors to impute the probabilities of receiving ORT values (Supp. Table 1) [12].

We assessed established diarrheal disease relative risk estimates ($e_{j,k}$) associated with household levels of water and sanitation access (Table 1) [34] to develop an exposure index [27] (Eq. (1.3)), where $G_{i,j,k}$ represents the *j*th level of household water and sanitation risk factor level k for individual child *i*. Adapted from Bagamian et al. [8], we scaled the mean individual risk index for each provincial area quintile subpopulation by overall mean risk index for the country. We assumed that the likelihood of a child experiencing an ETEC or *Shigella* diarrheal episode was associated with exposure risk. We used the same method to distribute diarrheal incidence rates as we used for mortality rates, except scaled, mean exposure index scores for each subpopulation were used in place of the overall diarrheal mortality risk index.

### 2.5 ETEC- and *Shigella*-induced stunting

We calculated the marginal effect of diarrheal episodes on increased likelihood of stunting by modeling the shift in z-scores from ETEC and *Shigella* episodes using GEMS results [4]. For each subpopulation, fractions of MSS children were estimated from DHS data [28], [29], [30], [31] to generate normal HAZ distributions and corresponding counterfactuals of distributions without ETEC or *Shigella* diarrheal episodes [9].

We estimated the shift in probability of MSS in each subpopulation using methods described previously [9]. The number of stunting cases due to ETEC and *Shigella* diarrheal episodes is the product of expected MSD episodes and the marginal effect on the probability of MSS. The probability is generated from the proportion of children below thresholds of moderate (height < −2 standard deviations (SD) below mean HAZ) and severe (height < −3 SD below mean HAZ) stunting.

We used a previously published approach [9] to estimate the number of deaths that stunted children experience from infectious diseases for which stunting is a risk factor (e.g., pneumonia, malaria, measles, and diarrheal deaths) for each subpopulation. We used the mid-point of IHME and MCEE national mortality rates for lower respiratory, malaria, measles, and non-ETEC and *Shigella* diarrhea from 2000 to 2016 as our baseline for mortality projections ranging from 2017 to 2034 (Table 1). We estimated the burden of these diseases in each provincial area by distributing the projected other infectious diseases mortality rates using the DHS under-five child mortality rate estimates. We assumed these rates did not vary across quintiles.

### 2.6 Costs

We estimated country-specific direct medical costs of ETEC and *Shigella* illness associated with inpatient and outpatient care for a diarrhea episode [11]. We based our direct medical costs on WHO-CHOICE Service Delivery Unit Cost estimates and commodity costs [35]. We assumed the proportion of children who sought care at outpatient facilities based on DHS data on seeking care at a healthcare facility after a diarrhea episode [28], [29], [30], [31]. We assumed one of eight outpatients were referred for inpatient care [36], [37].

We distributed the direct medical costs per child [11] using the estimated relative cost per child in each quintile (Table 1) [12]. We calculated subpopulation relative medical costs based on combining utilization of public and private, inpatient, and outpatient services after child diarrheal episodes from DHS with WHO-CHOICE national cost estimates adjusted to 2016 US$ [35]. As described for the probability of receiving ORT above, we imputed estimates for each child using the results of a logistic regression that included demographic and household characteristics as predictors for medical costs of illness (Supp. Table 1) [12], [13], [14].

We calculated net costs (N) for each country (c) by summing the difference of vaccination and averted costs in each quintile population (Eq. (2)). We calculated vaccination costs (V) for each quintile (q) by summing vaccine administration costs [38], vaccine price, and quantity for each vaccinated child in the subpopulation assuming 10% wastage over the first 10 years (t) post-introduction. We calculated averted costs (A) in each subpopulation as the product of vaccine effectiveness and estimated direct medical costs in quintiles (q) of each provincial area (a) population. We assumed the same costs of vaccination for ETEC and *Shigella* standalone vaccines.

(2) $$N_{c}=\sum_{t=10}^{a}\left(V_{a,q}-A_{a,q}\right)$$

All costs were discounted at 3% per year over the 10 years post-introduction and presented in 2016 US$.

### 2.7 Vaccine effectiveness and benefit

We evaluated the impact of national ETEC and *Shigella* vaccine programs separately, assuming each vaccine would prevent 60% of MSD episodes and 60% of acute mortality from diarrhea. We assumed full protection after a child received a three-dose course and did not include partial protection for less than three doses in our estimates. We estimated vaccination coverage for each regional and socioeconomic subpopulation using DHS data on diphtheria, pertussis, and tetanus vaccines (DPT3) [28], [29], [30], [31] as a proxy for ETEC and *Shigella* vaccine coverage.

As vaccine prices are uncertain until they are ready for market, we assumed that the vaccines in our study would cost US$3.30 per dose, as in our previous analysis [11], based in part on the Gavi Rotarix price of $2 per dose [39]. We used Disability-Adjusted Life Years (DALYs) to express burden from Years of Life Lost and YLD (Table 1) [40].

Vaccine benefits are calculated for each country ($B_{c}$) based on the sum of the product of quintile coverage ($C_{q}$), efficacy (${\mathrm{Ef}}_{r,q}$), and DALY burden, mortality or MSD episodes ($D_{q}$) in each provincial area (a) from 2025 to 2034 (t, Eq. (3)).

(3) $$B_{c}=\sum_{t=10}^{a}\left(C_{a,q}\cdot {\mathrm{Ef}}_{a,q}\cdot D_{a,q}\right)$$

We cumulated benefits over the first five years of life in each annual birth cohort as our measure of vaccination benefit. We assumed vaccine effectiveness did not wane, there was no effectiveness beyond five years of age, and did not include the potential of herd protection in our estimates.

As reducing disparities in access to vaccination could prevent more deaths and improve cost-effectiveness [41], we explored a hypothetical scenario simulating the impacts and cost-effectiveness of ETEC and *Shigella* vaccination if coverage in every socioeconomic subpopulation within each province was equal to the national average. We did not include costs of implementing interventions aimed at increasing coverage but did include costs of vaccinating additional children.

### 2.8 Cost-effectiveness

We calculated Incremental Cost-Effectiveness Ratios (ICERs) with net costs and outcomes cumulated over 2025–2034 projections for each subpopulation. We presented ICERs with two denominators, DALYs and MSD episodes averted. We included MSD episodes averted as a denominator to provide an alternative perspective on cost-effectiveness focused on preventing ETEC and *Shigella* episodes that induce stunting. Our comparator scenario is burden without ETEC or *Shigella* vaccination.

### 2.9 Uncertainty and sensitivity analyses

We conducted a series of analyses to assess the impact of uncertainty on our predicted outcomes using SimVoi [42] and Microsoft Excel® (version 16.27). We used probabilistic sensitivity analysis (PSA) to assess uncertainty in multiple input variables. We characterized key input variables as distributions based on our assumptions or uncertainty reported from original sources (Table 1) and conducted the PSA over 10,000 iterations to develop a distribution of estimated impact and cost-effectiveness by provincial area. We used the results from the uncertainty analysis to estimate upper (97.5%) and lower (2.5%) 95% uncertainty limits (UL) for key outputs and are reported along with point estimates.

We explored willingness-to-pay for vaccination introduction by conducting a threshold analysis on PSA results for each subpopulation. We present the results as the percentage of iterations from Monte Carlos simulations that produced ICERs below thresholds based on national GDP estimates. We compared ICERs to thresholds ranging from 0.25 to 4 times GDP [43], [44], [45]. We used a one-way sensitivity analyses to estimate the effect of changes in individual input variables. We explored ranges for key inputs (Table 1) and present results as tornado diagrams.

## 3 Results

### 3.1 ETEC and *Shigella* burden

Morbidity and mortality rates declined in every country except in Zimbabwe, where both were projected to increase from year 2025 to 2034 (Table 1). Children from DRC had the greatest number of ETEC and *Shigella* MSD episodes, while children from Zimbabwe had the highest incidence rate of MSD episodes associated with ETEC and *Shigella* (Table 2).

**Table 2:** National and regional estimates of ETEC and *Shigella* morbidity, mortality and economic costs projected for 2025–2034 in four East African countries,. All costs are presented in 2016 US$. Democratic Republic of Congo is abbreviated as ‘DRC'.

Country/region	MSD Episodes (1000 s)		MSD Episodes/100,000 children		Moderate and Severe Stunting Cases Due to MSD Diarrhoea (1000 s)		Moderate and Severe Stunting Cases Due to MSD Diarrhoea/100,000 Children		Direct Diarrhoeal Deaths		Direct Diarrhoeal Deaths/100,000 Children		Other ID Deaths Due to Diarrhoea-induced Stunting		Other ID Deaths Due to Diarrhoea-induced Stunting/100,000 Children		Direct Diarrhoeal and Other ID mortality due to Diarrhoea-induced Stunting (Total Deaths)		Total Deaths/100,000 Children		Total DALYs (1000 s)		Direct Medical Costs (US$/100,000 children)	
	ETEC	*Shigella*	ETEC	*Shigella*	ETEC	*Shigella*	ETEC	*Shigella*	ETEC	*Shigella*	ETEC	*Shigella*	ETEC	*Shigella*	ETEC	*Shigella*	ETEC	*Shigella*	ETEC	*Shigella*	ETEC	*Shigella*	ETEC	*Shigella*
DRC	4540 [2114, 8441]	4964 [2315, 9206]	2172 [1011, 4037]	2374 [1107, 4403]	841 [388, 1599]	1105 [513, 2091]	402 [186, 765]	528 [245, 1000]	22,642 [11,882, 39,595]	24,755 [12,995, 43,309]	10.8 [5.7, 18.9]	11.8 [6.2, 20.7]	5278 [2355, 10,345]	6896 [3104, 13,413]	2.5 [1.1, 4.9]	3.3 [1.5, 6.4]	27,920 [15,587, 47,119]	31,651 [17,735, 53,319]	13.4 [7.5, 22.5]	15.1 [8.5, 25.5]	971 [541, 1639]	1099 [616, 1853]	12,708 [5684, 24,580]	13,894 [6235, 26,732]
Bandundu	500 [232, 921]	547 [251, 1021]	2233 [1038, 4111]	2442 [1119, 4557]	91 [42, 172]	120 [54, 227]	408 [188, 770]	536 [243, 1015]	2271 [1183, 3968]	2483 [1294, 4339]	10.1 [5.3, 17.7]	11.1 [5.8, 19.4]	464 [204, 912]	606 [263, 1201]	2.1 [0.9, 4.1]	2.7 [1.2, 5.4]	2735 [1509, 4665]	3089 [1709, 5223]	12.2 [6.7, 20.8]	13.8 [7.6, 23.3]	95 [52, 162]	107 [59, 182]	7154 [3211, 13,973]	7822 [3505, 15,270]
Bas-Congo	183 [85, 343]	200 [94, 375]	2184 [1013, 4092]	2388 [1122, 4473]	38 [17, 73]	50 [23, 95]	456 [208, 873]	599 [276, 1132]	761 [405, 1314]	832 [440, 1443]	9.1 [4.8, 15.7]	9.9 [5.3, 17.2]	243 [108, 473]	318 [144, 613]	2.9 [1.3, 5.6]	3.8 [1.7, 7.3]	1004 [575, 1659]	1150 [660, 1908]	12.0 [6.9, 19.8]	13.7 [7.9, 22.8]	35 [20, 58]	40 [23, 66]	8979 [4076, 17,563]	9817 [4494, 19,215]
Equateur	476 [226, 882]	520 [248, 957]	2282 [1085, 4231]	2495 [1188, 4591]	83 [39, 156]	109 [51, 204]	397 [186, 748]	521 [244, 981]	2615 [1368, 4604]	2859 [1496, 5027]	12.5 [6.6, 22.1]	13.7 [7.2, 24.1]	592 [268, 1144]	773 [350, 1508]	2.8 [1.3, 5.5]	3.7 [1.7, 7.2]	3206 [1802, 5426]	3631 [2030, 6151]	15.4 [8.6, 26.0]	17.4 [9.7, 29.5]	111 [63, 189]	126 [71, 214]	7738 [3572, 15,127]	8461 [3925, 16,321]
Kasai-Occidental	386 [181, 722]	423 [199, 793]	2294 [1073, 4287]	2508 [1184, 4706]	87 [40, 167]	115 [53, 219]	518 [238, 991]	682 [317, 1300]	2089 [1113, 3692]	2284 [1204, 4041]	12.4 [6.6, 21.9]	13.6 [7.1, 24.0]	667 [298, 1298]	872 [397, 1685]	4.0 [1.8, 7.7]	5.2 [2.4, 10.0]	2755 [1561, 4617]	3155 [1795, 5296]	16.4 [9.3, 27.4]	18.7 [10.7, 31.4]	96 [54, 160]	109 [62, 184]	6169 [2801, 12,137]	6745 [3059, 13,219]
Kasai-Oriental	498 [234, 934]	544 [256, 1017]	2250 [1057, 4220]	2460 [1159, 4598]	101 [47, 193]	133 [62, 252]	458 [212, 873]	602 [280, 1139]	2770 [1470, 4822]	3028 [1591, 5279]	12.5 [6.6, 21.8]	13.7 [7.2, 23.9]	683 [304, 1343]	892 [404, 1753]	3.1 [1.4, 6.1]	4.0 [1.8, 7.9]	3453 [1932, 5759]	3921 [2213, 6585]	15.6 [8.7, 26.0]	17.7 [10.0, 29.8]	120 [67, 200]	136 [77, 229]	7970 [3583, 15,453]	8714 [3972, 16,952]
Katanga	583 [275, 1095]	638 [303, 1193]	2205 [1039, 4137]	2411 [1147, 4509]	111 [51, 211]	146 [69, 276]	419 [194, 796]	551 [260, 1042]	2890 [1522, 5088]	3160 [1677, 5535]	10.9 [5.8, 19.2]	11.9 [6.3, 20.9]	709 [320, 1380]	926 [422, 1783]	2.7 [1.2, 5.2]	3.5 [1.6, 6.7]	3599 [2044, 6084]	4086 [2328, 6828]	13.6 [7.7, 23.0]	15.4 [8.8, 25.8]	125 [71, 212]	142 [81, 237]	11,696 [5376, 22,875]	12,788 [5945, 24,941]
Kinshasa	423 [179, 735]	462 [196, 801]	1964 [833, 3417]	2148 [911, 3723]	30 [12, 53]	39 [16, 68]	137 [58, 245]	180 [76, 316]	1,093 [535, 1,767]	1,195 [593, 1,957]	5.1 [2.5, 8.2]	5.6 [2.8, 9.1]	71 [30, 128]	93 [38, 165]	0.3 [0.1, 0.6]	0.4 [0.2, 0.8]	1,165 [592, 1,861]	1,288 [668, 2,069]	5.4 [2.8, 8.6]	6.0 [3.1, 9.6]	41 [21, 65]	45 [23, 72]	23,244 [9,590, 42,033]	25,413 [10,435, 45,680]
Maniema	117 [55, 220]	127 [61, 239]	2100 [991, 3959]	2296 [1091, 4304]	21 [10, 40]	27 [13, 52]	376 [174, 715]	493 [230, 938]	604 [316, 1055]	660 [346, 1151]	10.9 [5.7, 19.0]	11.9 [6.2, 20.7]	113 [50, 221]	148 [67, 286]	2.0 [0.9, 4.0]	2.7 [1.2, 5.2]	717 [398, 1214]	808 [452, 1356]	12.9 [7.2, 21.9]	14.5 [8.1, 24.4]	25 [14, 42]	28 [16, 47]	18,850 [8571, 36,825]	20,610 [9476, 40,245]
Nord-Kivu	295 [138, 555]	323 [152, 606]	2060 [959, 3873]	2252 [1058, 4225]	62 [29, 117]	81 [38, 157]	429 [201, 817]	565 [264, 1092]	1402 [736, 2459]	1532 [810, 2696]	9.8 [5.1, 17.1]	10.7 [5.7, 18.8]	278 [123, 547]	363 [163, 716]	1.9 [0.9, 3.8]	2.5 [1.1, 5.0]	1680 [929, 2844]	1896 [1042, 3212]	11.7 [6.5, 19.8]	13.2 [7.3, 22.4]	58 [32, 99]	66 [36, 112]	10,310 [4683, 20,418]	11,272 [5170, 22,322]
Orientale	850 [402, 1597]	929 [438, 1732]	2120 [1003, 3984]	2317 [1091, 4319]	168 [78, 322]	220 [103, 417]	418 [194, 803]	549 [257, 1040]	4811 [2530, 8491]	5261 [2771, 9267]	12.0 [6.3, 21.2]	13.1 [6.9, 23.1]	1067 [477, 2127]	1394 [625, 2700]	2.7 [1.2, 5.3]	3.5 [1.6, 6.7]	5879 [3268, 10,078]	6655 [3727, 11,372]	14.7 [8.2, 25.1]	16.6 [9.3, 28.4]	204 [113, 351]	231 [129, 395]	19,476 [8878, 38,347]	21,293 [9709, 41,570]
Sud-Kivu	229 [107, 436]	250 [118, 473]	2177 [1015, 4153]	2380 [1121, 4499]	49 [23, 95]	65 [30, 124]	467 [217, 906]	615 [289, 1178]	1337 [703, 2334]	1462 [773, 2572]	12.7 [6.7, 22.2]	13.9 [7.4, 24.5]	390 [173, 772]	511 [230, 1004]	3.7 [1.7, 7.3]	4.9 [2.2, 9.6]	1727 [977, 2913]	1972 [1110, 3320]	16.4 [9.3, 27.7]	18.8 [10.6, 31.6]	60 [34, 101]	68 [39, 115]	13,021 [5899, 25,936]	14,236 [6498, 28,044]

KENYA	2413 [1131, 4471]	2638 [1231, 4885]	2493 [1169, 4619]	2725 [1272, 5048]	285 [132, 537]	373 [172, 704]	294 [136, 555]	386 [178, 727]	3772 [2722, 5149]	4124 [2984, 5605]	3.9 [2.8, 5.3]	4.3 [3.1, 5.8]	451 [206, 858]	587 [269, 1112]	0.5 [0.2, 0.9]	0.6 [0.3, 1.1]	4223 [3066, 5745]	4711 [3420, 6409]	4.4 [3.2, 5.9]	4.9 [3.5, 6.6]	150 [109, 204]	167 [121, 227]	23,650 [9978, 48,153]	25,857 [10,938, 52,730]
Central	205 [97, 381]	225 [106, 416]	2282 [1073, 4227]	2495 [1174, 4626]	17 [8, 32]	22 [10, 42]	188 [87, 355]	246 [115, 467]	252 [182, 343]	276 [199, 375]	2.8 [2.0, 3.8]	3.1 [2.2, 4.2]	18 [8, 34]	23 [11, 44]	0.2 [0.1, 0.4]	0.3 [0.1, 0.5]	270 [196, 366]	299 [217, 405]	3.0 [2.2, 4.1]	3.3 [2.4, 4.5]	10 [7, 13]	11 [8, 14]	36,108 [15,420, 75,148]	39,478 [16,763, 81,056]
Coast	199 [94, 369]	218 [103, 402]	2538 [1202, 4699]	2774 [1313, 5127]	26 [12, 50]	35 [16, 65]	337 [157, 634]	442 [206, 828]	285 [205, 389]	311 [223, 425]	3.6 [2.6, 5.0]	4.0 [2.8, 5.4]	43 [20, 81]	56 [26, 105]	0.5 [0.3, 1.0]	0.7 [0.3, 1.3]	328 [236, 445]	367 [265, 502]	4.2 [3.0, 5.7]	4.7 [3.4, 6.4]	12 [8, 16]	13 [9, 18]	27,399 [11,619, 55,888]	29,957 [13,057, 61,780]
Eastern	341 [161, 627]	373 [175, 693]	2544 [1203, 4672]	2781 [1305, 5169]	44 [21, 81]	58 [27, 109]	328 [153, 607]	430 [200, 813]	623 [448, 849]	681 [491, 927]	4.6 [3.3, 6.3]	5.1 [3.7, 6.9]	64 [30, 122]	84 [39, 160]	0.5 [0.2, 0.9]	0.6 [0.3, 1.2]	687 [496, 933]	765 [552, 1043]	5.1 [3.7, 7.0]	5.7 [4.1, 7.8]	24 [18, 33]	27 [20, 37]	25,635 [10,920, 52,014]	28,028 [11,971, 57,414]
Nairobi	146 [63, 248]	159 [69, 269]	2125 [924, 3616]	2323 [1006, 3922]	11 [5, 19]	14 [6, 24]	159 [68, 275]	209 [89, 355]	153 [107, 201]	167 [118, 219]	2.2 [1.6, 2.9]	2.4 [1.7, 3.2]	16 [7, 27]	20 [9, 35]	0.2 [0.1, 0.4]	0.3 [0.1, 0.5]	169 [119, 220]	188 [133, 244]	2.5 [1.7, 3.2]	2.7 [1.9, 3.6]	6 [4, 8]	7 [5, 9]	34,033 [13,222, 62,712]	37,210 [14,352, 68,237]
North Eastern	139 [66, 258]	152 [73, 287]	2614 [1236, 4845]	2857 [1362, 5391]	18 [8, 33]	23 [11, 44]	330 [154, 623]	432 [202, 828]	240 [174, 327]	263 [191, 361]	4.5 [3.3, 6.1]	4.9 [3.6, 6.8]	30 [14, 57]	39 [18, 75]	0.6 [0.3, 1.1]	0.7 [0.3, 1.4]	270 [196, 367]	302 [221, 414]	5.1 [3.7, 6.9]	5.7 [4.1, 7.8]	10 [7, 13]	11 [8, 15]	21,886 [9357, 45,504]	23,928 [10,333, 50,433]
Nyanza	426 [197, 792]	466 [217, 869]	2561 [1186, 4756]	2800 [1303, 5218]	46 [21, 88]	61 [28, 115]	279 [127, 527]	365 [167, 693]	616 [449, 845]	673 [488, 916]	3.7 [2.7, 5.1]	4.0 [2.9, 5.5]	92 [42, 175]	120 [55, 228]	0.6 [0.3, 1.0]	0.7 [0.3, 1.4]	708 [517, 966]	793 [578, 1080]	4.3 [3.1, 5.8]	4.8 [3.5, 6.5]	25 [18, 34]	28 [20, 38]	15,544 [6542, 31,682]	16,995 [7180, 34,729]
Rift Valley	635 [299, 1194]	695 [324, 1295]	2512 [1183, 4719]	2746 [1281, 5118]	85 [39, 163]	112 [52, 211]	338 [156, 642]	443 [204, 835]	1126 [812, 1547]	1231 [895, 1674]	4.5 [3.2, 6.1]	4.9 [3.5, 6.6]	127 [58, 245]	166 [76, 315]	0.5 [0.2, 1.0]	0.7 [0.3, 1.2]	1253 [912, 1716]	1397 [1017, 1905]	5.0 [3.6, 6.8]	5.5 [4.0, 7.5]	45 [32, 61]	50 [36, 68]	24,699 [10,543, 51,013]	27,004 [11,508, 55,771]
Western	320 [153, 603]	350 [165, 653]	2583 [1232, 4869]	2825 [1331, 5275]	37 [18, 71]	49 [23, 93]	300 [142, 577]	393 [184, 748]	478 [344, 647]	522 [378, 709]	3.9 [2.8, 5.2]	4.2 [3.0, 5.7]	61 [28, 117]	79 [37, 151]	0.5 [0.2, 0.9]	0.6 [0.3, 1.2]	538 [392, 731]	601 [437, 814]	4.3 [3.2, 5.9]	4.9 [3.5, 6.6]	19 [14, 26]	21 [16, 29]	13,832 [5902, 28,826]	15,123 [6497, 31,724]

ZAMBIA	994 [462, 1836]	1087 [504, 2012]	2348 [1136, 4511]	2567 [1238, 4944]	173 [81, 331]	228 [106, 435]	410 [199, 813]	538 [260, 1069]	1337 [773, 2164]	1462 [846, 2368]	3.2 [1.9, 5.3]	3.5 [2.1, 5.8]	322 [157, 662]	421 [206, 864]	0.8 [0.4, 1.6]	1.0 [0.5, 2.1]	1659 [1020, 2645]	1883 [1168, 3000]	3.9 [2.5, 6.5]	4.4 [2.9, 7.4]	58 [36, 93]	66 [41, 105]	28,473 [11,245, 54,288]	31,130 [12,313, 59,693]
Central	105 [49, 196]	114 [53, 215]	2382 [1124, 4467]	2604 [1212, 4886]	20 [9, 38]	26 [12, 50]	458 [214, 871]	602 [273, 1143]	152 [89, 248]	167 [97, 273]	3.5 [2.0, 5.6]	3.8 [2.2, 6.2]	40 [18, 77]	52 [24, 102]	0.9 [0.4, 1.8]	1.2 [0.5, 2.3]	192 [118, 305]	219 [135, 347]	4.4 [2.7, 6.9]	5.0 [3.1, 7.9]	7 [4, 11]	8 [5, 12]	38,987 [16,775, 81,167]	42,626 [18,386, 88,220]
Copperbelt	132 [57, 224]	144 [62, 246]	1986 [1146, 4477]	2171 [1246, 4921]	22 [10, 42]	29 [14, 56]	327 [207, 842]	430 [273, 1110]	171 [93, 259]	187 [102, 284]	2.6 [1.9, 5.2]	2.8 [2.0, 5.7]	30 [24, 98]	39 [31, 129]	0.5 [0.5, 2.0]	0.6 [0.6, 2.6]	201 [130, 330]	226 [151, 381]	3.0 [2.6, 6.6]	3.4 [3.0, 7.6]	7 [5, 12]	8 [5, 13]	34,966 [8573, 40,731]	38,229 [9295, 45,675]
Eastern	121 [57, 228]	133 [63, 251]	2427 [1144, 4553]	2654 [1259, 5011]	22 [10, 43]	29 [14, 57]	445 [207, 852]	585 [276, 1135]	160 [93, 259]	175 [102, 286]	3.2 [1.9, 5.2]	3.5 [2.0, 5.7]	52 [24, 100]	67 [32, 130]	1.0 [0.5, 2.0]	1.3 [0.6, 2.6]	212 [131, 329]	242 [153, 380]	4.2 [2.6, 6.6]	4.8 [3.1, 7.6]	7 [5, 12]	8 [5, 13]	20,079 [8560, 41,662]	21,953 [9342, 46,137]
Luapula	75 [35, 142]	82 [38, 152]	2467 [1158, 4668]	2698 [1267, 5023]	15 [7, 29]	20 [9, 38]	498 [232, 951]	654 [303, 1243]	90 [52, 147]	98 [57, 161]	3.0 [1.7, 4.8]	3.2 [1.9, 5.3]	35 [16, 68]	46 [21, 87]	1.2 [0.5, 2.2]	1.5 [0.7, 2.9]	125 [76, 197]	144 [89, 224]	4.1 [2.5, 6.5]	4.7 [2.9, 7.4]	4 [3, 7]	5 [3, 8]	13,023 [5562, 26,736]	14,239 [6111, 29,351]
Lusaka	158 [74, 296]	172 [80, 325]	2185 [1025, 4097]	2388 [1111, 4496]	25 [12, 47]	32 [15, 62]	342 [159, 657]	448 [207, 854]	144 [85, 234]	158 [92, 255]	2.0 [1.2, 3.2]	2.2 [1.3, 3.5]	34 [16, 66]	45 [21, 86]	0.5 [0.2, 0.9]	0.6 [0.3, 1.2]	179 [110, 283]	202 [126, 316]	2.5 [1.5, 3.9]	2.8 [1.7, 4.4]	6 [4, 10]	7 [4, 11]	21,904 [9223, 45,009]	23,948 [10,089, 49,850]
Muchinga	59 [27, 108]	64 [30, 119]	2540 [1181, 4693]	2777 [1296, 5159]	11 [5, 20]	14 [6, 26]	458 [212, 865]	601 [277, 1130]	106 [62, 175]	116 [68, 191]	4.6 [2.7, 7.6]	5.0 [2.9, 8.3]	24 [11, 46]	31 [14, 59]	1.0 [0.5, 2.0]	1.3 [0.6, 2.6]	130 [78, 209]	147 [90, 236]	5.6 [3.4, 9.0]	6.3 [3.9, 10.2]	5 [3, 7]	5 [3, 8]	36,722 [15,562, 75,941]	40,149 [16,966, 82,349]
Northern	57 [27, 107]	63 [29, 118]	2583 [1203, 4822]	2824 [1311, 5302]	12 [6, 23]	16 [7, 30]	545 [251, 1034]	717 [329, 1360]	111 [64, 182]	121 [71, 199]	5.0 [2.9, 8.2]	5.5 [3.2, 9.0]	30 [13, 59]	39 [18, 77]	1.4 [0.6, 2.7]	1.8 [0.8, 3.5]	141 [85, 224]	161 [98, 257]	6.4 [3.9, 10.1]	7.3 [4.4, 11.6]	5 [3, 8]	6 [3, 9]	23,751 [10,078, 48,693]	25,968 [11,169, 53,258]
North Western	91 [43, 168]	99 [47, 183]	2543 [1193, 4712]	2781 [1313, 5138]	15 [7, 28]	19 [9, 36]	413 [192, 782]	542 [255, 1019]	131 [77, 215]	144 [84, 231]	3.7 [2.2, 6.0]	4.0 [2.3, 6.5]	25 [11, 48]	32 [15, 62]	0.7 [0.3, 1.3]	0.9 [0.4, 1.7]	156 [95, 250]	176 [107, 276]	4.4 [2.7, 7.0]	4.9 [3.0, 7.8]	5 [3, 9]	6 [4, 10]	43,219 [18,578, 88,823]	47,252 [20,419, 96,247]
Southern	125 [59, 233]	137 [64, 257]	2423 [1147, 4528]	2650 [1240, 4994]	20 [9, 38]	26 [12, 51]	391 [182, 740]	513 [240, 983]	160 [93, 262]	175 [101, 287]	3.1 [1.8, 5.1]	3.4 [2.0, 5.6]	31 [14, 61]	41 [18, 79]	0.6 [0.3, 1.2]	0.8 [0.4, 1.5]	191 [115, 309]	216 [131, 343]	3.7 [2.2, 6.0]	4.2 [2.5, 6.7]	7 [4, 11]	8 [5, 12]	28,731 [12,203, 58,800]	31,413 [13,383, 65,425]
Western	72 [34, 134]	79 [37, 146]	2576 [1207, 4784]	2816 [1312, 5237]	12 [6, 23]	16 [7, 30]	425 [200, 808]	558 [256, 1067]	112 [65, 182]	122 [71, 200]	4.0 [2.3, 6.5]	4.4 [2.6, 7.2]	21 [10, 41]	28 [13, 54]	0.8 [0.4, 1.5]	1.0 [0.5, 1.9]	133 [81, 210]	150 [91, 240]	4.8 [2.9, 7.5]	5.4 [3.2, 8.6]	5 [3, 7]	5 [3, 8]	22,921 [9769, 46,909]	25,060 [10,712, 51,704]

ZIMBABWE	928 [434, 1741]	1015 [475, 1895]	2806 [1313, 5263]	3068 [1437, 5730]	110 [51, 210]	120 [55, 228]	333 [153, 636]	364 [168, 689]	8872 [5818, 13,228]	9700 [6349, 14,437]	26.8 [17.6, 40.0]	29.3 [19.2, 43.6]	735 [323, 1450]	799 [351, 1574]	2.2 [1.0, 4.4]	2.4 [1.1, 4.8]	9607 [6330, 14,262]	10,499 [6903, 15,572]	29.0 [19.1, 43.1]	31.7 [20.9, 47.1]	339 [224, 504]	371 [244, 550]	14,478 [7585, 26,449]	15,829 [8262, 29,019]
Manicaland	127 [59, 240]	139 [65, 256]	2871 [1340, 5423]	3139 [1475, 5785]	17 [8, 32]	18 [8, 34]	376 [172, 723]	411 [190, 774]	1394 [918, 2088]	1524 [1004, 2262]	31.5 [20.7, 47.2]	34.4 [22.7, 51.1]	130 [57, 253]	141 [63, 277]	2.9 [1.3, 5.7]	3.2 [1.4, 6.3]	1523 [1011, 2259]	1665 [1100, 2471]	34.4 [22.8, 51.0]	37.6 [24.8, 55.8]	54 [36, 80]	59 [39, 87]	16,043 [8485, 29,487]	17,540 [9169, 32,189]
Mashonaland Central	92 [43, 172]	101 [48, 188]	3088 [1453, 5756]	3376 [1596, 6298]	11 [5, 21]	12 [6, 23]	371 [172, 710]	406 [188, 762]	924 [598, 1388]	1010 [662, 1505]	31.0 [20.1, 46.6]	33.9 [22.2, 50.5]	79 [35, 157]	86 [38, 169]	2.7 [1.2, 5.3]	2.9 [1.3, 5.7]	1004 [654, 1497]	1097 [722, 1623]	33.7 [21.9, 50.2]	36.8 [24.2, 54.5]	35 [23, 53]	39 [25, 57]	7313 [3859, 13,414]	7995 [4210, 14,656]
Mashonaland East	115 [54, 216]	126 [59, 233]	2903 [1356, 5450]	3174 [1483, 5895]	13 [6, 25]	14 [7, 27]	329 [151, 626]	360 [165, 682]	1184 [778, 1761]	1294 [839, 1939]	29.9 [19.7, 44.5]	32.7 [21.2, 49.0]	93 [40, 184]	101 [44, 197]	2.3 [1.0, 4.7]	2.5 [1.1, 5.0]	1277 [843, 1892]	1395 [907, 2077]	32.3 [21.3, 47.8]	35.3 [22.9, 52.5]	45 [30, 67]	49 [32, 73]	11,571 [5998, 20,974]	12,651 [6545, 23,103]
Mashonaland West	109 [51, 209]	120 [56, 230]	2964 [1391, 5666]	3241 [1527, 6228]	15 [7, 29]	16 [7, 31]	398 [184, 775]	435 [200, 839]	1129 [744, 1680]	1234 [814, 1837]	30.6 [20.1, 45.5]	33.4 [22.0, 49.7]	110 [49, 218]	119 [53, 236]	3.0 [1.3, 5.9]	3.2 [1.4, 6.4]	1238 [819, 1827]	1353 [898, 2006]	33.5 [22.2, 49.5]	36.6 [24.3, 54.3]	44 [29, 65]	48 [32, 71]	8348 [4364, 15,621]	9127 [4760, 16,796]
Matabeleland North	59 [27, 109]	64 [30, 119]	3007 [1408, 5573]	3288 [1540, 6081]	6 [3, 11]	6 [3, 12]	292 [134, 549]	319 [148, 608]	318 [208, 474]	347 [227, 516]	16.3 [10.7, 24.3]	17.8 [11.6, 26.5]	21 [9, 41]	23 [10, 44]	1.1 [0.5, 2.1]	1.2 [0.5, 2.3]	339 [224, 502]	370 [242, 548]	17.3 [11.5, 25.7]	19.0 [12.4, 28.1]	12 [8, 18]	13 [9, 19]	30,945 [16,192, 56,583]	33,833 [17,742, 61,942]
Matabeleland South	49 [23, 92]	54 [25, 102]	2867 [1346, 5335]	3134 [1469, 5956]	6 [3, 12]	7 [3, 13]	361 [166, 684]	395 [183, 758]	370 [243, 547]	405 [265, 602]	21.5 [14.1, 31.8]	23.5 [15.4, 35.0]	33 [15, 65]	36 [16, 72]	1.9 [0.9, 3.8]	2.1 [0.9, 4.2]	404 [266, 598]	441 [291, 654]	23.5 [15.5, 34.8]	25.7 [16.9, 38.1]	14 [9, 21]	16 [10, 23]	16,298 [8454, 29,452]	17,819 [9197, 32,693]
Midlands	122 [57, 231]	134 [62, 247]	2917 [1346, 5500]	3189 [1472, 5882]	16 [7, 30]	17 [8, 33]	378 [173, 724]	414 [188, 777]	1529 [1003, 2282]	1672 [1093, 2490]	36.4 [23.9, 54.3]	39.8 [26.0, 59.3]	136 [59, 270]	148 [64, 294]	3.2 [1.4, 6.4]	3.5 [1.5, 7.0]	1665 [1095, 2492]	1820 [1197, 2700]	39.7 [26.1, 59.3]	43.3 [28.5, 64.3]	59 [39, 88]	64 [42, 95]	9476 [4940, 17,387]	10,360 [5378, 18,920]
Masvingo	112 [53, 210]	123 [57, 231]	2975 [1395, 5557]	3253 [1510, 6104]	13 [6, 24]	14 [6, 27]	339 [159, 644]	371 [171, 702]	1087 [713, 1622]	1188 [778, 1768]	28.7 [18.9, 42.9]	31.4 [20.6, 46.8]	81 [36, 160]	88 [38, 173]	2.1 [0.9, 4.2]	2.3 [1.0, 4.6]	1167 [770, 1738]	1276 [837, 1895]	30.9 [20.4, 46.0]	33.8 [22.1, 50.1]	41 [27, 61]	45 [30, 67]	16,646 [8680, 30,008]	18,200 [9498, 32,886]
Harare	114 [53, 210]	124 [59, 231]	2398 [1125, 4430]	2622 [1237, 4880]	12 [5, 22]	13 [6, 24]	248 [113, 469]	271 [125, 509]	831 [544, 1228]	909 [591, 1343]	17.6 [11.5, 25.9]	19.2 [12.5, 28.4]	48 [21, 93]	53 [23, 103]	1.0 [0.4, 2.0]	1.1 [0.5, 2.2]	880 [576, 1293]	961 [629, 1419]	18.6 [12.2, 27.3]	20.3 [13.3, 30.0]	31 [20, 46]	34 [22, 50]	17,176 [9015, 31,341]	18,779 [9807, 35,057]
Bulawayo	28 [13, 53]	31 [15, 58]	1728 [824, 3243]	1890 [901, 3570]	2 [1, 4]	2 [1, 5]	140 [66, 266]	153 [72, 290]	107 [70, 158]	117 [76, 173]	6.5 [4.3, 9.6]	7.1 [4.7, 10.6]	4 [2, 8]	5 [2, 9]	0.3 [0.1, 0.5]	0.3 [0.1, 0.5]	111 [73, 164]	121 [80, 179]	6.8 [4.4, 10.0]	7.4 [4.9, 11.0]	4 [3, 6]	4 [3, 6]	22,611 [11,977, 41,413]	24,721 [13,023, 44,867]

Within countries, children from the provinces of Kasai-Occidental (DRC), North Eastern (Kenya), Northern (Zambia), and Mashonaland Central (Zimbabwe) had the highest predicted incidence rates of ETEC and *Shigella* MSD episodes from 2025 to 2034 (Table 2).

**Fig. 2:**
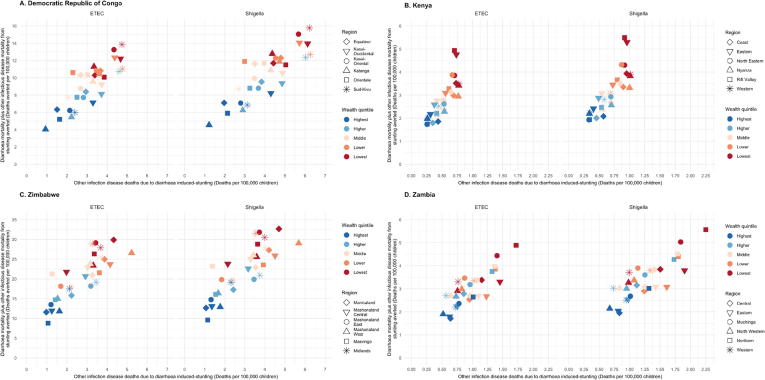
Total mortality from ETEC or *Shigella* diarrheal deaths and other infectious disease deaths for children from each wealth quintile by the fraction of stunted children under five years of age. The six provinces with the highest total death rates are displayed for each study country.

**Fig. 3:**
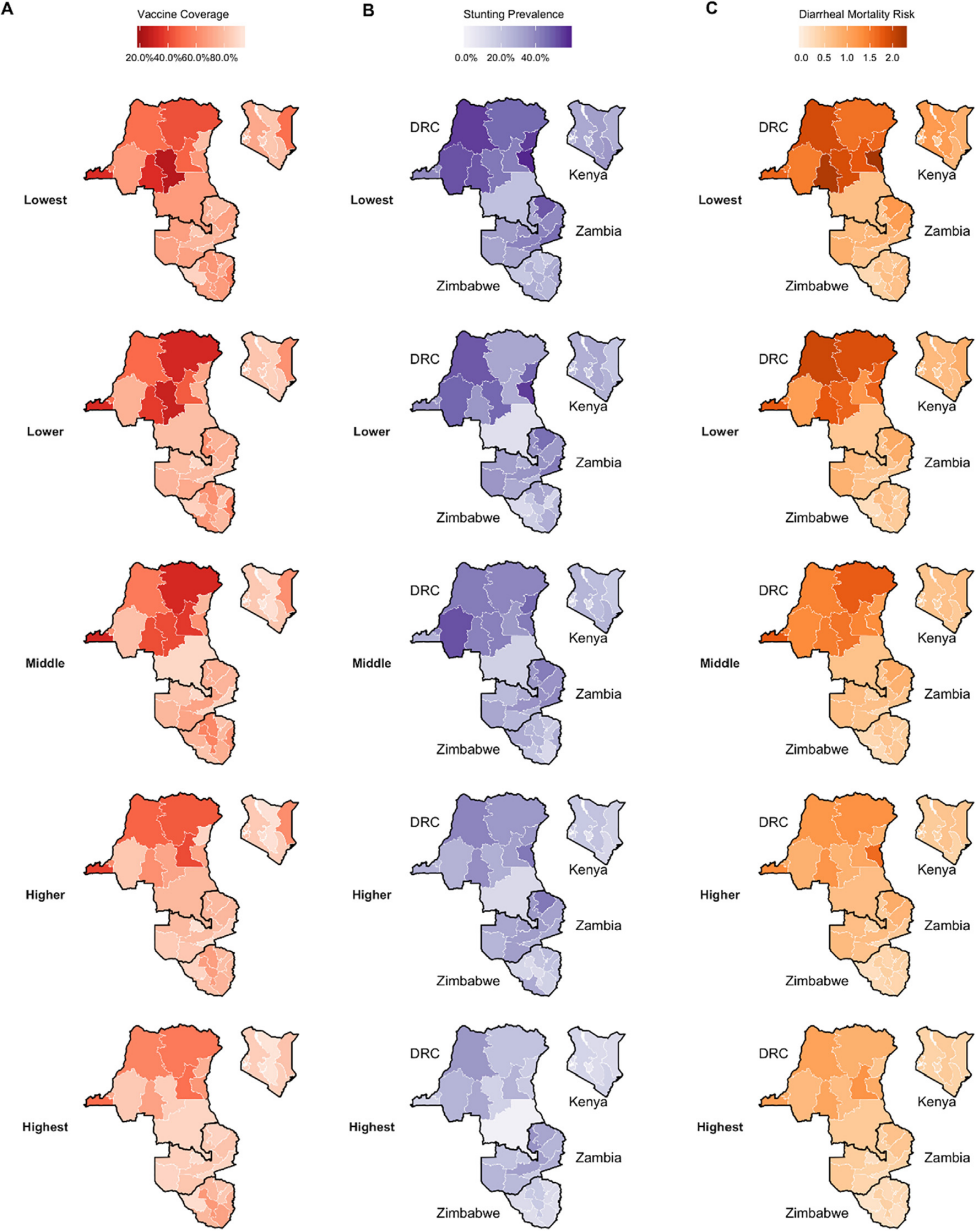
Distribution of key model inputs for under five children across geographic and socioeconomic subpopulations. (A) ETEC and *Shigella* vaccine coverage, modeled using Diphtheria, Pertussis and Tetanus dose 3 coverage from the most recent Demographic and Health Surveys. (B) Stunting prevalence (proportion of children in each wealth quintile and province with height-for-age z-scores less than −2 standard deviations below the median WHO reference height), modeled using DHS data on child nutritional status. (C) Subpopulation diarrheal mortality risk was calculated by averaging individual index scores for children in each subpopulation. Higher scores represent higher risk of mortality from ETEC or *Shigella* diarrhea.

Children from these provinces were estimated to be among the highest rates of total mortality (ETEC and *Shigella* diarrheal mortality plus other infectious disease deaths due to ETEC- and *Shigella*-induced stunting). In DRC, *Shigella* total mortality rates were greatest in Sud-Kivu and ETEC total mortality rates were greatest and equivalent in Kasai-Occidental and Sud-Kivu (Table 2). In Kenya, total mortality rates were greatest in North Eastern, but equivalent to rates in Eastern for ETEC and *Shigella*. In Zimbabwe, children from Midlands province had the highest total mortality rates (Table 2). In the highest total mortality provinces in all four countries, children in the lowest and lower quintiles are projected to experience the highest total mortality rates (Fig. 2, Supplemental Table 1). This is a reflection of higher percentages of stunted children and higher diarrheal mortality risk index scores for children in these provinces (Fig. 3B and C).

### 3.2 ETEC- and *Shigella*-induced stunting burden

ETEC and *Shigella* MSD diarrhea was projected to cause the highest number of stunted children in DRC, with the highest rates of stunted children in Zambia, followed closely by DRC (Table 2) from 2025 to 2034. Burden from other infectious disease deaths accounted for between 8 (Zimbabwe: 735/9607 deaths) and 19% (Zambia: 322/1659 deaths and DRC: 5278/27,920) of total ETEC burden and between 8 (Zimbabwe: 799/10,499 deaths) and 22% (Zambia: 421/1883 deaths and DRC: 6896/31,651) of *Shigella* total burden. Burden from other infectious disease deaths due to ETEC or *Shigella* stunting accounted for the highest percentage of total burden in Kasai-Occidental (ETEC: 24% [667/2755 deaths]; *Shigella*: 28% [872/3155 deaths]) and Bas-Congo (ETEC: 24% [243/1004 deaths]; *Shigella*: 28% [318/1150 deaths]) in DRC, Coast (ETEC: 13% [43/328 deaths]; *Shigella*: 15% [56/367 deaths]) in Kenya, Luapula (ETEC: 28% [35/125 deaths]; *Shigella*: 32% [46/144 deaths]) in Zambia, and Manicaland (ETEC: 9% [130/1523 deaths]; *Shigella*: 9% [141/1665 deaths]) and Mashonaland West (ETEC: 9% [110/1238 deaths]; *Shigella*: 9% [119/1353 deaths]) in Zimbabwe. In the highest total mortality rate provinces, the highest rates of induced stunting burden were found in the lowest and lower wealth quintiles, with the exception of Midlands, Zimbabwe where the middle quintile was equivalent to the lowest quintile estimate (Fig. 2).

### 3.3 Vaccine effectiveness

Our estimates showed that over the decade following vaccine introduction, nearly 50% of the ETEC and *Shigella* morbidity and mortality burden could be prevented in Kenya (49% [UL: 43%; 52%]) and Zambia (48% [43%; 49%]), where estimated vaccination coverage was higher and more equitable (Fig. 3A). Our model predicted lower estimated reductions in Zimbabwe (43% [39%; 45%]) and DRC (30% [28%; 31%]) (Table 3). Vaccination coverage estimates were much lower and disparities in coverage were much higher, particularly in DRC (Fig. 3A), leading to the largest provincial disparities in reduction in DRC (range; 22% [16%; 27%]–48% [33%; 58%]). Provincial disparities in reduction were the smallest in Kenya (range; 37% [27%; 46%]–53% [39%; 65%]) (Table 3).

**Table 3:** National and regional estimates of health and economic impact and benefits of ETEC and *Shigella* vaccination from 2025 to 2034 in four East African countries. Abbreviations: DRC, Democratic Republic of Congo; ICER, Incremental Cost-Effectiveness Ratio. Costs and ICERs are presented in 2016 US$.

Country/region	Fully Vaccinated Children (1000 s)	Burden Reduction	MSD Diarrhoeal Episodes Averted (1000 s)		MSD Diarrhoeal Episodes Averted/100,000 FVC		Moderate and Severe Stunting Cases Due to MSD Diarrhoea Averted (1000 s)		Direct Diarrhoeal Deaths Averted		Other ID Deaths Due to Diarrhoea-induced Stunting Averted		Direct Diarrhoeal and Other ID mortality Averted due to Diarrhoea-induced Stunting Averted (Total Deaths Averted)		Total Deaths Averted/100,000 FVC		Total DALYs Averted (1000 s)		Costs Averted (1000 s US$)		ICER (US$/DALY averted)		ICER (US$/MSD episode averted)		Equitable Coverage ICER (US$/DALY averted)	
	Both*	Both*	ETEC	*Shigella*	ETEC	*Shigella*	ETEC	*Shigella*	ETEC	*Shigella*	ETEC	*Shigella*	ETEC	*Shigella*	ETEC	*Shigella*	ETEC	*Shigella*	ETEC	*Shigella*	ETEC	*Shigella*	ETEC	*Shigella*	ETEC	*Shigella*
DRC	115,704	30% [28%, 31%]	1488 [641, 2845]	1626 [701, 3102]	1286 [554, 2459]	1406 [606, 2681]	262 [113, 513]	345 [148, 671]	6897 [3337, 12,412]	7541 [3638, 13,618]	1596 [663, 3207]	2086 [874, 4173]	8494 [4358, 14,829]	9627 [4943, 16,832]	7.3 [3.8, 12.8]	8.3 [4.3, 14.5]	295 [151, 516]	334 [172, 585]	9348 [3884, 18,407]	10,220 [4261, 20,032]	1428 [735, 3107]	1258 [649, 2742]	283 [128, 688]	259 [117, 624]	1310 [585, 2475]	1154 [517, 2189]
Bandundu	13,757	36% [26%, 45%]	184 [78, 352]	201 [86, 388]	1336 [568, 2556]	1460 [628, 2819]	33 [14, 64]	43 [18, 84]	823 [398, 1469]	900 [432, 1617]	168 [68, 338]	219 [89, 442]	991 [508, 1728]	1119 [567, 1963]	7.2 [3.7, 12.6]	8.1 [4.1, 14.3]	34 [18, 60]	39 [20, 68]	630 [262, 1262]	689 [291, 1378]	1469 [749, 3152]	1300 [661, 2793]	276 [130, 693]	252 [117, 629]	1445 [597, 2484]	1279 [527, 2184]
Bas-Congo	6504	46% [33%, 57%]	85 [37, 164]	93 [41, 180]	1306 [567, 2515]	1428 [625, 2773]	18 [7, 34]	23 [10, 45]	349 [171, 621]	382 [185, 689]	111 [47, 220]	145 [62, 286]	460 [241, 790]	527 [276, 905]	7.1 [3.7, 12.1]	8.1 [4.2, 13.9]	16 [8, 27]	18 [10, 31]	357 [151, 717]	391 [169, 780]	1495 [774, 3139]	1305 [673, 2712]	281 [131, 688]	257 [119, 620]	1471 [564, 2283]	1283 [492, 1971]
Equateur	7822	22% [16%, 27%]	107 [47, 205]	117 [51, 224]	1365 [602, 2625]	1492 [657, 2859]	18 [8, 35]	24 [10, 46]	571 [274, 1041]	624 [301, 1149]	128 [53, 257]	167 [71, 338]	699 [359, 1229]	791 [409, 1401]	8.9 [4.6, 15.7]	10.1 [5.2, 17.9]	24 [12, 43]	27 [14, 49]	397 [170, 801]	434 [186, 865]	1183 [594, 2541]	1044 [529, 2237]	269 [126, 660]	246 [116, 594]	1147 [525, 2161]	1012 [464, 1901]
Kasai-Occidental	8825	31% [23%, 39%]	121 [53, 233]	133 [57, 252]	1375 [596, 2640]	1503 [646, 2853]	27 [12, 53]	36 [15, 70]	650 [318, 1189]	711 [346, 1294]	208 [86, 412]	272 [114, 536]	859 [448, 1510]	983 [513, 1710]	9.7 [5.1, 17.1]	11.1 [5.8, 19.4]	30 [16, 52]	34 [18, 59]	341 [144, 683]	373 [156, 740]	1092 [553, 2286]	953 [482, 2016]	268 [124, 660]	245 [113, 598]	1083 [439, 1823]	946 [388, 1583]
Kasai-Oriental	10,878	27% [20%, 34%]	145 [63, 281]	159 [70, 304]	1336 [582, 2581]	1460 [641, 2796]	29 [12, 57]	38 [16, 73]	758 [372, 1362]	829 [406, 1505]	190 [80, 381]	248 [104, 502]	948 [490, 1658]	1077 [561, 1896]	8.7 [4.5, 15.2]	9.9 [5.2, 17.4]	33 [17, 58]	37 [19, 66]	587 [250, 1177]	642 [273, 1275]	1212 [615, 2552]	1066 [537, 2250]	275 [127, 673]	251 [116, 609]	1130 [407, 1684]	995 [359, 1479]
Katanga	13,321	25% [18%, 31%]	171 [75, 332]	187 [83, 363]	1283 [564, 2489]	1402 [620, 2722]	29 [12, 57]	38 [17, 74]	729 [354, 1320]	798 [387, 1444]	179 [75, 359]	233 [99, 468]	908 [471, 1584]	1031 [534, 1817]	6.8 [3.5, 11.9]	7.7 [4.0, 13.6]	32 [16, 55]	36 [19, 63]	1072 [455, 2172]	1172 [507, 2364]	1538 [780, 3277]	1354 [682, 2879]	284 [130, 693]	259 [119, 631]	1289 [477, 1985]	1134 [424, 1772]
Kinshasa	17,236	48% [33%, 58%]	203 [80, 361]	221 [87, 393]	1175 [463, 2092]	1285 [504, 2280]	14 [5, 25]	18 [7, 32]	523 [235, 870]	571 [258, 962]	33 [13, 61]	43 [16, 79]	556 [261, 915]	615 [287, 1018]	3.2 [1.5, 5.3]	3.6 [1.7, 5.9]	19 [9, 32]	21 [10, 36]	2422 [941, 4461]	2648 [1026, 4876]	3186 [1439, 6296]	2871 [1275, 5639]	305 [123, 676]	278 [111, 621]	3165 [1449, 6388]	2852 [1303, 5745]
Maniema	2479	27% [20%, 33%]	31 [14, 60]	34 [15, 66]	1259 [554, 2421]	1377 [613, 2654]	6 [2, 11]	7 [3, 14]	162 [80, 293]	178 [86, 322]	30 [13, 60]	39 [17, 78]	192 [99, 339]	217 [112, 382]	7.8 [4.0, 13.7]	8.7 [4.5, 15.4]	7 [3, 12]	8 [4, 13]	284 [121, 569]	311 [134, 623]	1337 [672, 2866]	1185 [596, 2534]	287 [129, 701]	262 [119, 636]	1340 [517, 2242]	1187 [463, 1996]
Nord-Kivu	11,169	46% [34%, 57%]	138 [60, 266]	151 [66, 292]	1235 [536, 2384]	1350 [588, 2618]	29 [12, 57]	38 [16, 75]	650 [316, 1173]	710 [345, 1299]	130 [54, 262]	170 [71, 344]	780 [396, 1374]	880 [446, 1555]	7.0 [3.5, 12.3]	7.9 [4.0, 13.9]	27 [14, 48]	31 [15, 54]	692 [292, 1383]	756 [323, 1522]	1509 [765, 3218]	1335 [682, 2835]	297 [139, 727]	271 [127, 663]	1499 [551, 2388]	1327 [482, 2111]
Orientale	16,552	24% [17%, 29%]	209 [93, 408]	229 [101, 443]	1265 [562, 2464]	1383 [609, 2674]	41 [18, 81]	53 [23, 105]	1132 [552, 2072]	1237 [600, 2240]	258 [108, 527]	337 [141, 672]	1390 [722, 2463]	1575 [819, 2759]	8.4 [4.4, 14.9]	9.5 [4.9, 16.7]	48 [25, 86]	55 [28, 96]	2012 [864, 4056]	2200 [938, 4391]	1234 [611, 2606]	1087 [546, 2302]	285 [128, 701]	260 [117, 639]	1180 [519, 2193]	1040 [453, 1944]
Sud-Kivu	7161	41% [30%, 51%]	94 [41, 184]	102 [45, 198]	1307 [577, 2570]	1429 [626, 2765]	20 [9, 40]	27 [12, 52]	549 [265, 1003]	601 [291, 1097]	162 [67, 331]	212 [90, 429]	711 [363, 1239]	813 [421, 1426]	9.9 [5.1, 17.3]	11.3 [5.9, 19.9]	25 [13, 43]	28 [15, 49]	553 [235, 1125]	605 [257, 1218]	1058 [538, 2245]	925 [476, 1963]	279 [127, 692]	255 [117, 628]	1064 [387, 1596]	931 [338, 1391]

KENYA	80,295	49% [43%, 52%]	1196 [521, 2269]	1308 [572, 2491]	1490 [649, 2826]	1629 [712, 3103]	140 [60, 270]	183 [79, 354]	1848 [1178, 2702]	2020 [1286, 2939]	220 [94, 428]	287 [124, 556]	2068 [1322, 3014]	2307 [1472, 3355]	2.6 [1.6, 3.8]	2.9 [1.8, 4.2]	74 [47, 107]	82 [52, 119]	11,594 [4575, 24,145]	12,676 [5015, 26,443]	3911 [2442, 7553]	3496 [2184, 6771]	240 [108, 599]	219 [98, 545]	3850 [1805, 5517]	3442 [1603, 4943]
Central	8039	53% [39%, 66%]	110 [48, 208]	120 [53, 229]	1366 [600, 2592]	1493 [656, 2847]	9 [4, 17]	12 [5, 23]	134 [86, 195]	147 [94, 213]	9 [4, 18]	12 [5, 24]	144 [92, 208]	159 [102, 230]	1.8 [1.1, 2.6]	2.0 [1.3, 2.9]	5 [3, 7]	6 [4, 8]	1750 [697, 3676]	1914 [762, 4011]	5503 [3131, 9908]	4945 [2838, 8855]	257 [114, 652]	234 [103, 588]	5475 [2580, 7864]	4920 [2275, 7179]
Coast	6540	50% [36%, 62%]	99 [43, 191]	109 [48, 208]	1521 [656, 2914]	1663 [735, 3177]	13 [6, 26]	17 [8, 33]	142 [89, 208]	155 [98, 226]	21 [9, 42]	28 [12, 54]	163 [103, 238]	183 [116, 266]	2.5 [1.6, 3.6]	2.8 [1.8, 4.1]	6 [4, 8]	6 [4, 9]	1082 [430, 2277]	1183 [478, 2480]	4021 [2311, 7246]	3576 [2056, 6467]	234 [106, 592]	213 [95, 528]	4001 [1669, 5122]	3558 [1485, 4571]
Eastern	6540	53% [39%, 65%]	181 [80, 344]	198 [87, 377]	1522 [673, 2891]	1664 [728, 3166]	23 [10, 44]	30 [13, 59]	328 [209, 481]	358 [228, 520]	34 [15, 65]	44 [19, 86]	361 [231, 527]	402 [257, 585]	3.0 [1.9, 4.4]	3.4 [2.2, 4.9]	13 [8, 19]	14 [9, 21]	1848 [736, 3870]	2021 [808, 4297]	3306 [1919, 5975]	2961 [1706, 5391]	235 [107, 587]	214 [96, 541]	3267 [1254, 3773]	2926 [1115, 3367]
Nairobi	5827	51% [36%, 62%]	74 [30, 129]	81 [33, 140]	1274 [520, 2213]	1393 [565, 2404]	6 [2, 10]	7 [3, 13]	78 [48, 109]	85 [52, 118]	8 [3, 14]	11 [4, 19]	86 [53, 119]	96 [59, 132]	1.5 [0.9, 2.0]	1.6 [1.0, 2.3]	3 [2, 4]	3 [2, 5]	1193 [436, 2240]	1304 [475, 2424]	6704 [3594, 11,023]	5995 [3247, 9893]	276 [111, 628]	251 [101, 570]	6716 [2619, 8344]	6007 [2335, 7392]
North Eastern	5827	37% [27%, 46%]	53 [23, 102]	58 [26, 114]	1552 [683, 2980]	1697 [749, 3312]	7 [3, 13]	9 [4, 17]	90 [58, 132]	98 [63, 145]	11 [5, 22]	14 [6, 29]	101 [65, 149]	113 [73, 166]	2.9 [1.9, 4.3]	3.3 [2.1, 4.8]	4 [2, 5]	4 [3, 6]	478 [191, 1022]	523 [211, 1121]	3429 [1931, 6111]	3063 [1723, 5369]	231 [103, 572]	211 [92, 526]	3324 [1560, 4665]	2970 [1387, 4222]
Nyanza	13,707	49% [36%, 61%]	210 [92, 399]	230 [102, 443]	1535 [674, 2912]	1678 [743, 3230]	23 [10, 44]	30 [13, 58]	303 [195, 443]	331 [211, 482]	45 [20, 88]	59 [26, 114]	348 [225, 508]	390 [250, 568]	2.5 [1.6, 3.7]	2.8 [1.8, 4.1]	12 [8, 18]	14 [9, 20]	1292 [516, 2726]	1412 [569, 2956]	4026 [2341, 7073]	3588 [2070, 6382]	237 [110, 582]	216 [99, 531]	4010 [1789, 5433]	3573 [1589, 4832]
Rift Valley	20,565	48% [35%, 59%]	309 [135, 589]	338 [148, 649]	1501 [655, 2863]	1641 [717, 3154]	41 [18, 80]	54 [23, 105]	537 [341, 786]	587 [377, 856]	61 [26, 119]	80 [34, 155]	598 [381, 874]	667 [428, 972]	2.9 [1.9, 4.3]	3.2 [2.1, 4.7]	21 [14, 31]	24 [15, 35]	3082 [1223, 6488]	3369 [1337, 7153]	3457 [2006, 6135]	3092 [1784, 5546]	238 [106, 598]	217 [97, 542]	3384 [1203, 3667]	3027 [1073, 3289]
Western	10,278	50% [36%, 61%]	159 [69, 307]	174 [76, 332]	1548 [672, 2988]	1692 [741, 3235]	18 [8, 36]	24 [10, 47]	237 [152, 348]	259 [163, 378]	30 [13, 59]	39 [17, 76]	266 [171, 390]	298 [188, 436]	2.6 [1.7, 3.8]	2.9 [1.8, 4.2]	9 [6, 14]	11 [7, 15]	869 [346, 1846]	950 [376, 2002]	3952 [2304, 6956]	3534 [2050, 6267]	235 [109, 582]	215 [99, 535]	3931 [1767, 5264]	3515 [1561, 4693]

ZAMBIA	34,252	48% [43%, 49%]	479 [206, 902]	524 [225, 987]	1398 [632, 2,773]	1,529 [690, 3,033]	83 [36, 162]	109 [47, 211]	637 [332, 1,065]	697 [365, 1,164]	153 [69, 321]	200 [90, 418]	790 [436, 1,305]	897 [498, 1,480]	2.3 [1.3, 4.0]	2.6 [1.5, 4.5]	28 [15, 46]	31 [17, 52]	5,967 [2,078, 11,020]	6,524 [2,284, 12,124]	4,376 [2,127, 8,234]	3,845 [1,863, 7,182]	254 [109, 611]	231 [99, 555]	3,716 [1,545, 5,875]	3,262 [1,351, 5,163]
Central	3410	46% [34%, 57%]	49 [21, 94]	53 [23, 103]	1425 [624, 2752]	1558 [685, 3017]	9 [4, 18]	12 [5, 24]	70 [37, 119]	77 [41, 132]	19 [8, 37]	24 [10, 49]	89 [50, 148]	101 [56, 169]	2.6 [1.5, 4.3]	3.0 [1.6, 4.9]	3 [2, 5]	4 [2, 6]	811 [327, 1730]	886 [359, 1896]	3803 [1949, 7762]	3326 [1670, 6785]	245 [106, 617]	222 [95, 561]	3262 [1308, 5169]	2850 [1147, 4506]
Copperbelt	5855	52% [36%, 61%]	69 [26, 115]	76 [28, 127]	1183 [630, 2783]	1294 [686, 3056]	11 [5, 21]	15 [6, 28]	89 [42, 134]	98 [46, 146]	16 [11, 49]	21 [14, 64]	105 [58, 171]	118 [67, 196]	1.8 [1.4, 4.1]	2.0 [1.6, 4.7]	4 [2, 6]	4 [2, 7]	1241 [199, 1067]	1356 [218, 1188]	5548 [2163, 8176]	4908 [1862, 7071]	297 [114, 628]	270 [103, 570]	4741 [1614, 6169]	4191 [1448, 5491]
Eastern	4142	49% [36%, 61%]	60 [26, 115]	66 [29, 127]	1454 [638, 2787]	1590 [698, 3075]	11 [5, 21]	14 [6, 28]	79 [42, 133]	86 [46, 147]	25 [11, 50]	33 [14, 65]	104 [58, 170]	119 [67, 195]	2.5 [1.4, 4.1]	2.9 [1.6, 4.7]	4 [2, 6]	4 [2, 7]	508 [201, 1072]	555 [221, 1193]	4086 [2157, 8043]	3562 [1872, 7012]	248 [113, 613]	226 [101, 560]	3506 [1452, 5351]	3054 [1271, 4648]
Luapula	2237	44% [32%, 54%]	33 [15, 64]	36 [16, 69]	1477 [650, 2859]	1615 [724, 3086]	7 [3, 13]	9 [4, 17]	39 [21, 67]	43 [23, 73]	15 [7, 30]	20 [9, 39]	55 [31, 90]	63 [35, 103]	2.4 [1.4, 4.0]	2.8 [1.6, 4.6]	2 [1, 3]	2 [1, 4]	188 [75, 400]	205 [83, 429]	4259 [2272, 8381]	3690 [1988, 7295]	247 [114, 607]	225 [105, 547]	3666 [1975, 7140]	3176 [1694, 6181]
Lusaka	6205	51% [37%, 63%]	81 [36, 157]	89 [39, 172]	1307 [578, 2534]	1429 [629, 2772]	13 [6, 25]	17 [7, 32]	74 [39, 125]	81 [43, 136]	17 [8, 35]	23 [10, 45]	91 [50, 150]	103 [58, 169]	1.5 [0.8, 2.4]	1.7 [0.9, 2.7]	3 [2, 5]	4 [2, 6]	824 [325, 1750]	901 [358, 1936]	6952 [3668, 13,976]	6118 [3215, 12,133]	275 [124, 690]	251 [111, 623]	5966 [2321, 8563]	5247 [2028, 7537]
Muchinga	1754	45% [33%, 56%]	27 [12, 51]	29 [13, 55]	1522 [659, 2890]	1665 [719, 3153]	5 [2, 9]	6 [3, 12]	48 [25, 82]	52 [28, 88]	11 [4, 21]	14 [6, 27]	58 [32, 98]	66 [36, 109]	3.3 [1.8, 5.6]	3.8 [2.0, 6.2]	2 [1, 3]	2 [1, 4]	393 [154, 831]	430 [172, 913]	2990 [1524, 6191]	2629 [1348, 5387]	230 [102, 589]	209 [91, 534]	2554 [1006, 3975]	2244 [885, 3476]
Northern	1752	47% [34%, 58%]	27 [12, 52]	30 [13, 56]	1547 [668, 2947]	1691 [727, 3214]	6 [2, 11]	8 [3, 14]	52 [27, 89]	57 [30, 97]	14 [6, 28]	19 [8, 36]	67 [36, 110]	76 [41, 126]	3.8 [2.1, 6.3]	4.3 [2.4, 7.2]	2 [1, 4]	3 [1, 4]	258 [102, 530]	282 [112, 586]	2683 [1415, 5430]	2350 [1237, 4754]	231 [105, 591]	211 [95, 537]	2310 [966, 3660]	2022 [840, 3222]
North Western	2803	47% [34%, 58%]	43 [19, 81]	47 [20, 89]	1523 [662, 2904]	1665 [730, 3162]	7 [3, 13]	9 [4, 18]	62 [33, 104]	67 [36, 113]	12 [5, 23]	15 [6, 30]	73 [40, 121]	82 [46, 135]	2.6 [1.4, 4.3]	2.9 [1.6, 4.8]	3 [1, 4]	3 [2, 5]	739 [293, 1536]	808 [324, 1686]	3769 [1894, 7806]	3325 [1681, 6792]	227 [98, 585]	206 [90, 533]	3225 [1511, 6140]	2842 [1312, 5408]
Southern	3886	45% [33%, 56%]	56 [25, 108]	61 [27, 117]	1447 [640, 2779]	1582 [689, 3022]	9 [4, 18]	12 [5, 23]	72 [38, 123]	79 [42, 133]	14 [6, 28]	18 [8, 36]	86 [47, 144]	97 [54, 161]	2.2 [1.2, 3.7]	2.5 [1.4, 4.2]	3 [2, 5]	3 [2, 6]	691 [275, 1445]	756 [300, 1580]	4558 [2366, 9331]	4026 [2105, 8152]	245 [111, 606]	223 [100, 553]	3919 [1877, 7159]	3460 [1626, 6308]
Western	2206	47% [34%, 58%]	34 [15, 65]	37 [16, 71]	1541 [671, 2942]	1684 [732, 3238]	6 [2, 11]	7 [3, 14]	52 [28, 89]	57 [31, 97]	10 [4, 20]	13 [5, 26]	62 [35, 104]	70 [39, 117]	2.8 [1.6, 4.7]	3.2 [1.8, 5.3]	2 [1, 4]	2 [1, 4]	315 [126, 661]	345 [137, 718]	3599 [1861, 7240]	3184 [1658, 6440]	233 [105, 588]	212 [95, 535]	3088 [1418, 5424]	2731 [1255, 4857]

ZIMBABWE	24,380	43% [39%, 45%]	407 [177, 787]	445 [193, 857]	1670 [726, 3227]	1826 [793, 3513]	47 [20, 93]	52 [22, 101]	3805 [2249, 5969]	4160 [2450, 6528]	310 [128, 628]	337 [138, 681]	4115 [2444, 6439]	4497 [2657, 7048]	16.9 [10.0, 26.4]	18.4 [10.9, 28.9]	145 [86, 228]	159 [94, 249]	2191 [1059, 4129]	2395 [1154, 4510]	610 [458, 1555]	557 [417, 1425]	218 [103, 558]	199 [95, 509]	487 [283, 951]	445 [257, 871]
Manicaland	2923	38% [28%, 47%]	50 [21, 96]	54 [24, 104]	1702 [732, 3283]	1861 [805, 3563]	6 [3, 12]	7 [3, 14]	532 [316, 834]	582 [343, 910]	49 [20, 99]	53 [22, 107]	581 [346, 909]	635 [376, 992]	19.9 [11.8, 31.1]	21.7 [12.9, 33.9]	21 [12, 32]	22 [13, 35]	291 [142, 551]	319 [152, 595]	517 [284, 996]	471 [263, 889]	213 [98, 542]	194 [90, 494]	410 [162, 530]	374 [148, 489]
Mashonaland Central	2268	45% [33%, 56%]	42 [18, 81]	46 [20, 88]	1846 [813, 3579]	2018 [881, 3879]	5 [2, 10]	5 [2, 11]	419 [245, 661]	458 [268, 716]	36 [15, 73]	39 [16, 79]	455 [269, 714]	497 [291, 776]	20.1 [11.9, 31.5]	21.9 [12.8, 34.2]	16 [10, 25]	18 [10, 27]	103 [50, 193]	112 [54, 211]	519 [291, 978]	475 [265, 894]	199 [92, 487]	182 [85, 442]	426 [224, 738]	390 [205, 682]
Mashonaland East	3142	47% [34%, 59%]	55 [23, 106]	60 [26, 115]	1736 [745, 3363]	1898 [823, 3667]	6 [3, 12]	7 [3, 13]	559 [331, 868]	611 [361, 963]	44 [18, 89]	47 [19, 96]	602 [358, 938]	658 [389, 1037]	19.2 [11.4, 29.8]	20.9 [12.4, 33.0]	21 [13, 33]	23 [14, 37]	222 [106, 421]	243 [117, 457]	540 [302, 1018]	493 [275, 942]	211 [97, 523]	192 [90, 480]	441 [167, 551]	402 [152, 504]
Mashonaland West	2485	39% [29%, 49%]	44 [19, 87]	48 [21, 94]	1763 [774, 3487]	1928 [843, 3785]	6 [3, 12]	6 [3, 13]	446 [265, 704]	487 [287, 766]	43 [18, 88]	47 [20, 95]	489 [292, 765]	534 [317, 838]	19.7 [11.8, 30.8]	21.5 [12.8, 33.7]	17 [10, 27]	19 [11, 30]	133 [64, 257]	145 [70, 279]	529 [297, 998]	483 [272, 905]	208 [94, 514]	190 [85, 469]	427 [209, 695]	391 [192, 636]
Matabeleland North	1661	51% [37%, 63%]	30 [13, 57]	33 [14, 63]	1798 [790, 3456]	1966 [859, 3779]	3 [1, 6]	3 [1, 6]	161 [95, 252]	176 [104, 277]	10 [4, 21]	11 [5, 23]	171 [102, 268]	187 [111, 293]	10.3 [6.1, 16.1]	11.3 [6.7, 17.6]	6 [4, 9]	7 [4, 10]	311 [151, 590]	340 [166, 639]	972 [535, 1833]	885 [487, 1682]	197 [89, 494]	179 [81, 450]	788 [344, 1192]	717 [311, 1087]
Matabeleland South	1287	44% [32%, 55%]	22 [10, 43]	24 [10, 47]	1715 [757, 3317]	1875 [815, 3653]	3 [1, 5]	3 [1, 6]	164 [96, 256]	179 [106, 280]	15 [6, 30]	16 [7, 32]	179 [105, 279]	195 [115, 305]	13.9 [8.2, 21.7]	15.2 [9.0, 23.7]	6 [4, 10]	7 [4, 11]	127 [60, 237]	138 [66, 265]	740 [409, 1420]	676 [373, 1293]	212 [97, 523]	193 [88, 478]	601 [250, 846]	548 [229, 785]
Midlands	2787	40% [29%, 49%]	49 [21, 95]	53 [23, 102]	1750 [756, 3405]	1914 [828, 3674]	6 [3, 12]	7 [3, 13]	609 [358, 961]	666 [393, 1049]	54 [22, 112]	59 [24, 121]	663 [394, 1046]	725 [429, 1142]	23.8 [14.1, 37.5]	26.0 [15.4, 41.0]	23 [14, 37]	26 [15, 40]	160 [78, 300]	175 [85, 326]	436 [239, 814]	399 [219, 747]	210 [96, 523]	191 [89, 474]	361 [176, 591]	329 [160, 540]
Masvingo	2765	44% [32%, 54%]	49 [22, 95]	54 [23, 104]	1788 [782, 3429]	1955 [845, 3768]	6 [2, 11]	6 [3, 12]	476 [283, 746]	520 [305, 814]	36 [15, 71]	39 [16, 78]	511 [305, 799]	559 [329, 875]	18.5 [11.0, 28.9]	20.2 [11.9, 31.6]	18 [11, 28]	20 [12, 31]	275 [133, 519]	301 [145, 564]	555 [308, 1030]	506 [276, 960]	203 [94, 491]	185 [85, 456]	456 [182, 620]	416 [166, 563]
Harare	3706	47% [34%, 57%]	53 [23, 100]	58 [25, 109]	1431 [618, 2711]	1565 [674, 2951]	5 [2, 10]	6 [2, 11]	387 [228, 603]	423 [250, 662]	22 [9, 43]	23 [10, 47]	409 [241, 636]	447 [264, 697]	11.0 [6.5, 17.2]	12.0 [7.1, 18.8]	14 [9, 23]	16 [9, 25]	387 [185, 720]	423 [202, 798]	929 [522, 1750]	848 [479, 1598]	253 [118, 640]	231 [109, 578]	757 [318, 1055]	690 [285, 968]
Bulawayo	1356	50% [36%, 62%]	14 [6, 27]	15 [7, 29]	1034 [453, 1992]	1131 [500, 2170]	1 [0, 2]	1 [1, 2]	53 [31, 83]	58 [34, 91]	2 [1, 4]	2 [1, 4]	55 [33, 86]	60 [36, 94]	4.1 [2.4, 6.3]	4.4 [2.6, 6.9]	2 [1, 3]	2 [1, 3]	183 [89, 341]	200 [98, 374]	2499 [1393, 4718]	2278 [1261, 4339]	347 [158, 844]	316 [144, 773]	2052 [795, 2690]	1869 [724, 2457]

Disparities in rates of total mortality averted by wealth status were higher in DRC and Zimbabwe as compared with Zambia and Kenya. In Zimbabwe, total mortality rates averted in Manicaland and Mashonaland Central were over 5 times higher than in urban Bulawayo (Table 3). In DRC, total mortality rates averted in Kasai-Occidental were 4 (ETEC) and 3.5 (*Shigella*) times higher than those predicted for urban Kinshasa. The total deaths averted were greatest in the lowest and lower subpopulations, where there were larger fractions of stunted children (Fig. 1) and higher ETEC and *Shigella* induced stunting burden rates (Fig. 2). Rates of total mortality averted were higher in provincial areas where mortality rates were among the highest, despite lower vaccination effectiveness (% reduction), in provinces such as Kasai-Occidental (DRC), North Eastern (Kenya), and Manicaland (Zimbabwe) (Table 2).

### 3.4 Cost-effectiveness

Both vaccines had ICERs below $1000 per DALY in all provinces except Bulawayo in Zimbabwe (Table 3). Provincial ICERs in DRC ranged between $1058 [$538; $2245] in Sud-Kivu and $3186 [$1439; $6296] per DALY in Kinshasa for ETEC vaccination and $925 [$476; $1963] in Sud-Kivu and $2871[$1275; $5639] per DALY in Kinshasa for *Shigella* vaccination. The lowest provincial ICERs for ETEC vaccines were $3306 [$1919; $5975] in Eastern, Kenya and $2683 [$1415; $5430] per DALY averted in Northern, Zambia (Table 3). Following the same pattern, the lowest provincial ICERs for *Shigella* vaccines were $2961 [$1706; $5391] in Eastern, Kenya and $2350 [$1237; $4754] per DALY averted in Northern, Zambia.

Subnational ICERs with MSD averted as the denominator showed a different pattern, with lower variation between countries than DALY ICERs. The highest provincial MSD ICER was found in Bulawayo and the lowest in Matabeleland North, both in Zimbabwe (Table 3). Provincial disparities across wealth quintiles in MSD episode ICERs were highest in Zimbabwe, ranging from $197 [$89; $494] in Matabeleland North to $347 [$158; $844] in Bulawayo (ETEC) and $179 [$81; $450] in Matabeleland North to $316 [$144; $773] in Bulawayo (*Shigella*) per MSD episode averted.

**Fig. 4:**
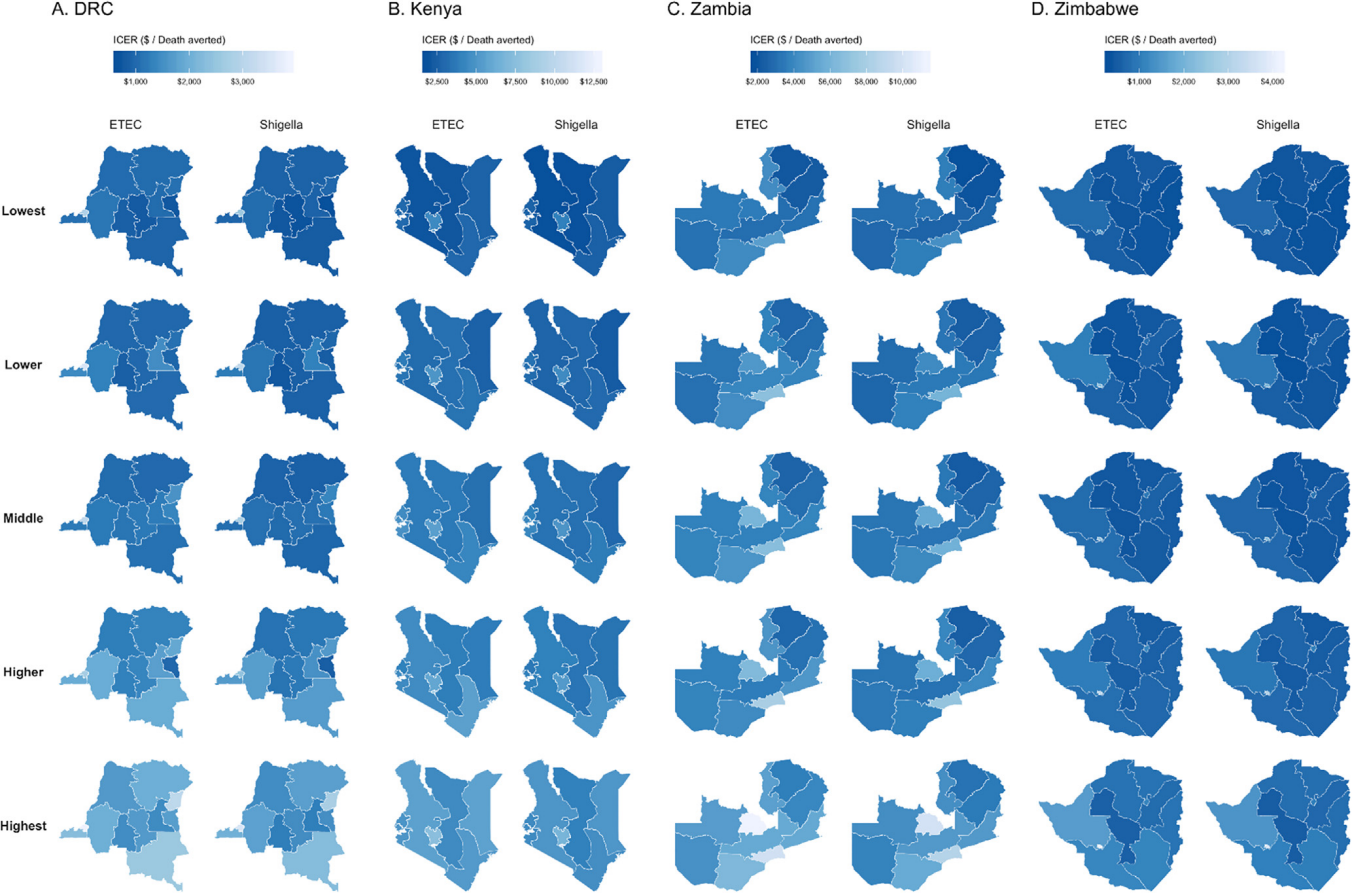
Estimates of Incremental Cost-Effectiveness Ratios (ICERs) for ETEC and *Shigella* vaccination for under five children in each wealth quintile projected for the first 10 years (2025–2034) post vaccine introduction. ICERs are presented in 2016 US$.

**Fig. 5:**
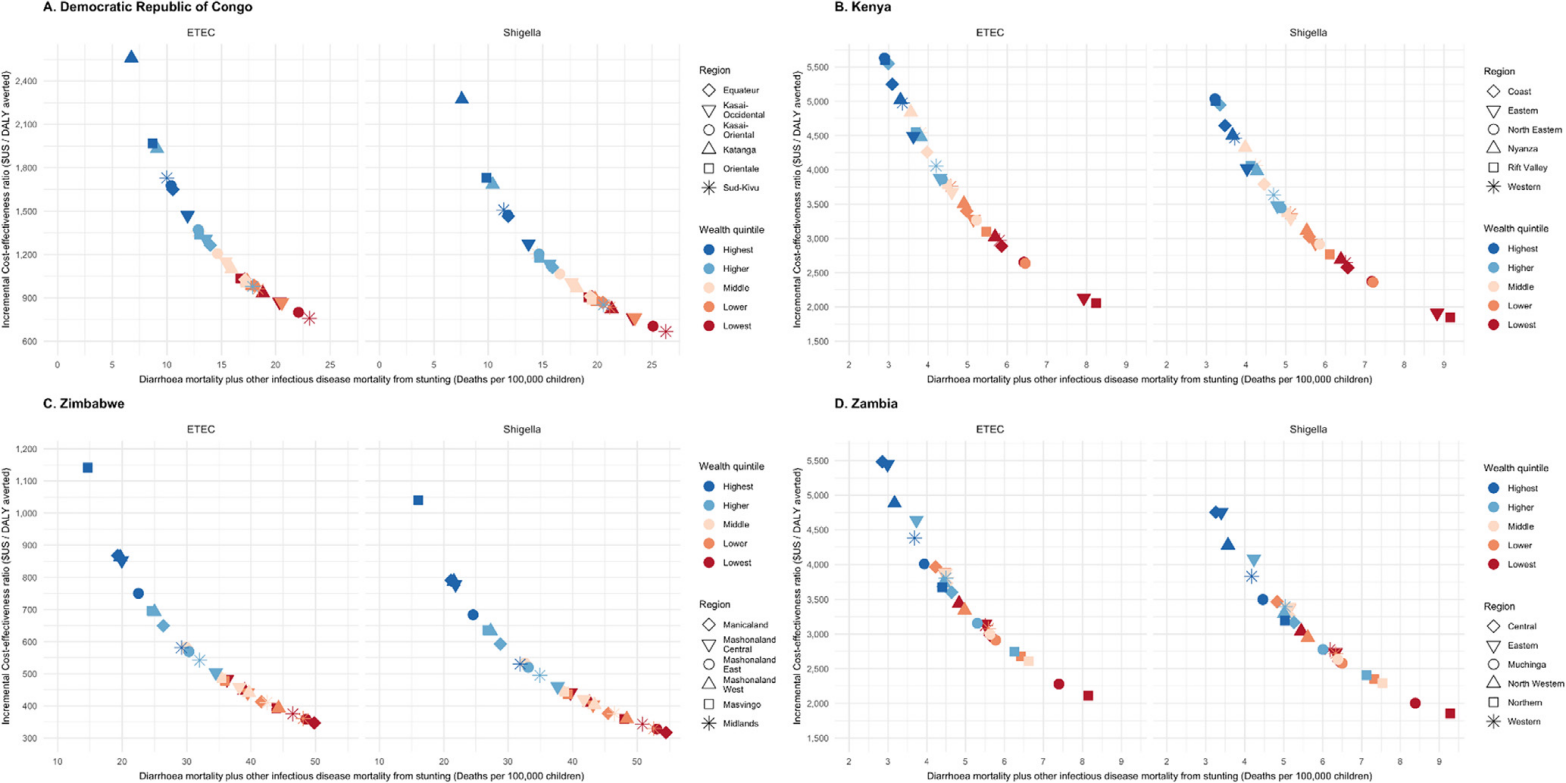
Incremental Cost-Effectiveness Ratio estimates of vaccination of under five children in each wealth quintile by total mortality. Total mortality includes deaths due to ETEC or *Shigella* diarrhea directly or from deaths from other infectious disease deaths resulting from ETEC and *Shigella*-associated stunting. The six provinces with the highest total death rates are displayed for each study country. ICERs are presented in 2016 US$.

The lowest quintile subpopulations had more favorable DALY ICERs for ETEC, ranging from $759 [$385; $1618] (Sud-Kivu) to $2895 [$1309; $5705] (Kinshasa) in DRC, $2055 [$1204, $3620] (Rift Valley) to $4680 [$2514; $7731] (Nairobi) in Kenya, $2112 [$1124; $4248] (Northern) to $4948 [$2648; $9898] (Lusaka) in Zambia, and $347 [$192; $666] (Manicaland) to $1743 [$976; $3285] (Bulawayo) in Zimbabwe (Fig. 4, Supplement Table 2). More cost-effective results were found in the lowest wealth quintiles for *Shigella* as well, with DALY ICERs ranging from $667 [$343; $1413] (Sud-Kivu) to $2606 [$1161; $5105] (Kinshasa) in DRC, $1845 [$1080; $3288] (Rift Valley) to $4123 [$2222; $6850] (Nairobi) in Kenya, $1852 [$988; $3720] (Northern) to $4352 [$2318; $8537] (Lusaka) in Zambia, and $317 [$178; $596] (Manicaland) to $1591 [$887; $3000] (Bulawayo) in Zimbabwe (Fig. 4). Generally, ETEC and *Shigella* vaccinations were most cost-effective in subpopulations with the greatest burden (Fig. 5).

**Fig. 6:**
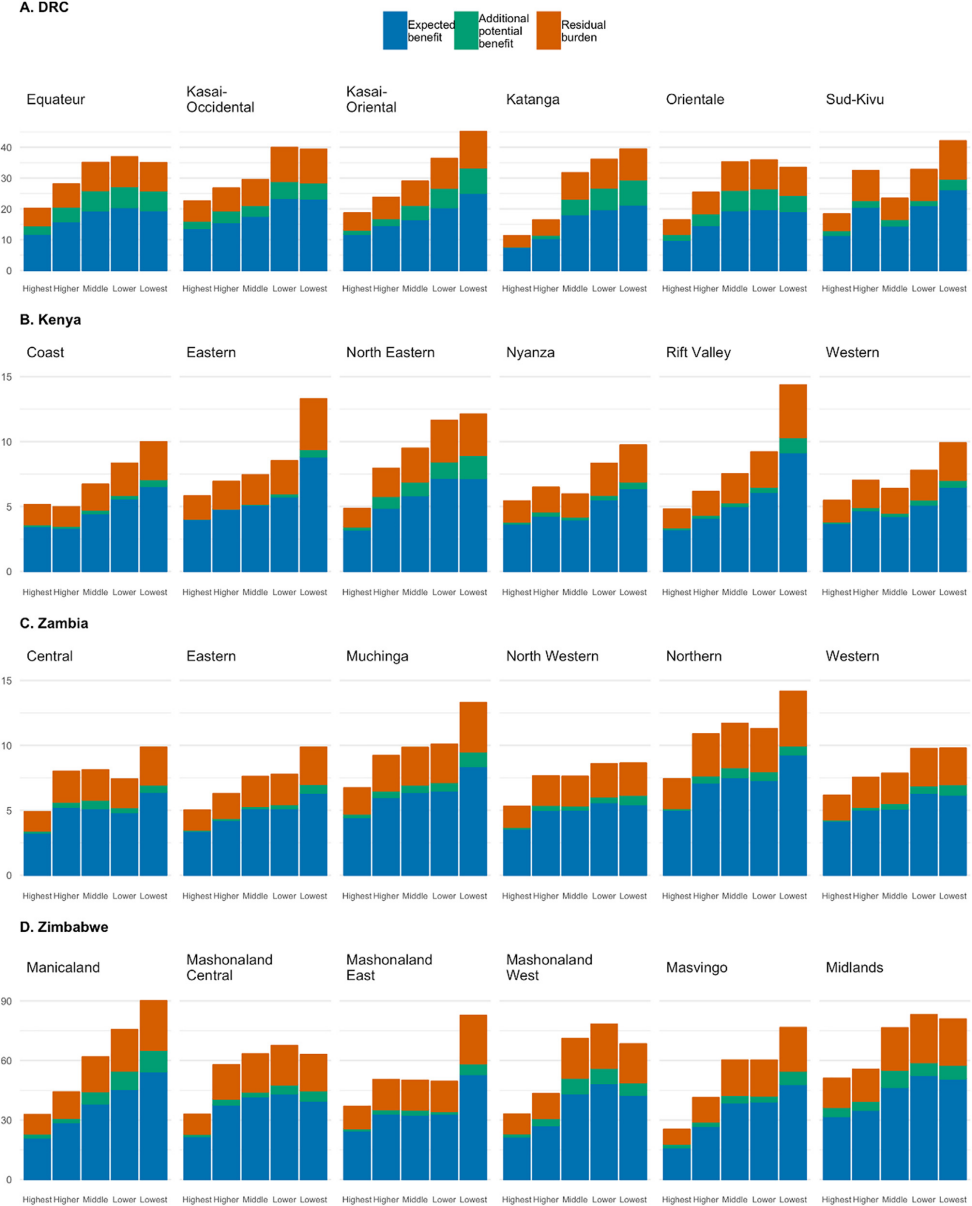
Estimated expected and potential rates of averted deaths per 100,000 children of *Shigella* vaccination for quintiles within six provinces for each study country. Expected benefit (blue bars) represents impact of base case estimates, while potential benefit (green bars) is the burden averted if every socioeconomic subpopulation had vaccination coverage equal to the national average in each country. Estimated residual deaths per 100,000 children (orange bars) are the rates of mortality that would not be averted after subtracting expected and potential benefit in each subpopulation. The six provinces with the highest total death rates are displayed for each study country. (For interpretation of the references to color in this figure legend, the reader is referred to the web version of this article.)

Under our equitable scenario (vaccination coverage of each subpopulation being equal to the national average), the most significant gains in deaths averted were for children in the middle, lower, and lowest quintiles in Equateur, Kasai-Oriental, and Katanga provinces in DRC, North Eastern province in Kenya, and Manicaland provinces in Zimbabwe (Fig. 6). National ICERs declined by 20%, 15%, 8%, and 2% in Zimbabwe, Zambia, DRC, and Kenya, respectively (Table 3).

Based on the threshold analysis, subpopulations within Zimbabwe met willingness-to-pay thresholds of ICERs being less than 1 times GDP in greater than 50% of our simulation iterations in seven of the ten provincial areas (Supplemental Fig. 1). ICERs were less than three times GDP in more than 90% of model iterations in all subpopulations in all regions with the exception of the wealthiest subpopulations in Bulaweyo. In DRC, Kenya, and Zambia, ICERs in some of the poorest subpopulations were less than two times GDP in over 50% of simulation iterations (Supplemental Figs. 2–4). With the exceptions of Kinshasa (DRC) and Lusaka (Zambia) ICERs estimated for the poorest subpopulations were below three times GDP in 50% or higher of simulation iterations.

**Fig. 7:**
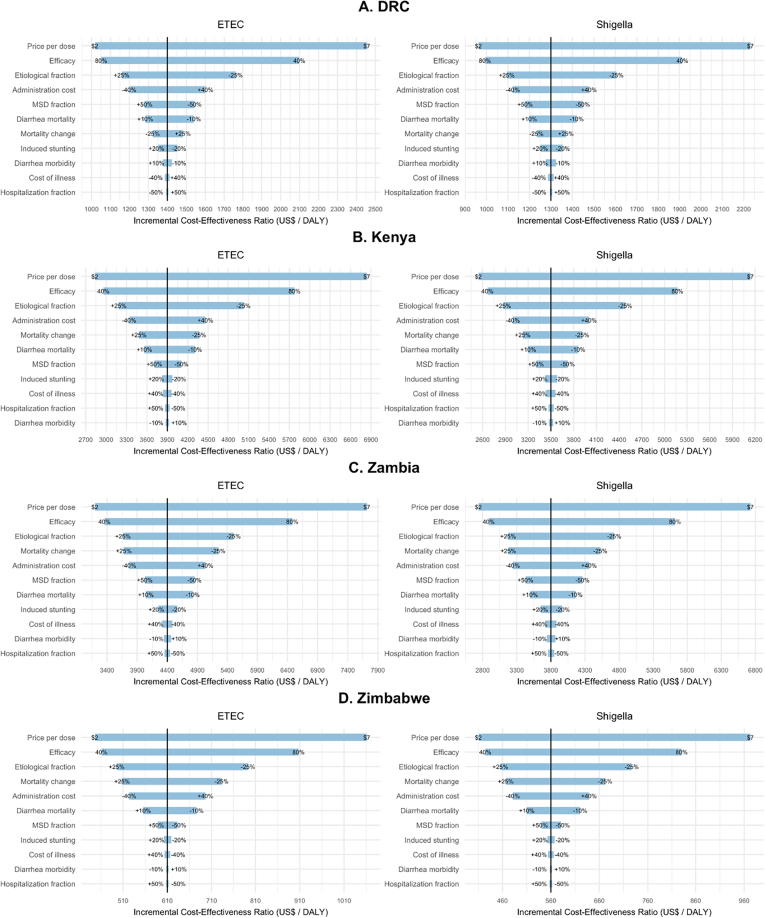
Tornado diagram showing results of one-way PSA exploring the affect key input variables have on cost-effectiveness of ETEC and *Shigella* standalone vaccines in for sub-Saharan African countries from 2025 to 2034. Ranges of variables (listed in Table 1) are displayed at the end of the corresponding bar. Price per dose is varied by 2016 $US ranging from a low estimate of $2/dose to a high estimate of $7/dose. ‘Etiological fraction’ is variation in the fraction of overall diarrheal mortality attributed to ETEC and *Shigella* diarrhea. ‘Mortality change’ is variation in the rates of diarrheal mortality projected in years 2025 to 2034. ‘Induced stunting’ refers to the number of other infectious disease deaths caused by ETEC or *Shigella* induced stunting. ‘Hospitalization fraction’ is variation in the fraction of children hospitalized (1 in 8 referred to inpatient facility).

The model inputs tested in the sensitivity analysis that had the greatest impact on DALY ICERs in order from greatest to least impactful were price per dose, efficacy and etiological fractions for both ETEC and *Shigella* vaccination in all countries (Fig. 7). These three inputs accounted for the majority of ICER value swings with different patterns in the impact of variation in the fourth and fifth most impactful model inputs across countries. In Kenya, the next two most impactful inputs were administration cost followed by mortality change over time, while in Zambia and Zimbabwe the order was reversed with mortality change having more impact than administration cost. In DRC, ICER sensitivity to variation in model inputs was higher for estimates of administration costs followed by the fraction of episodes estimated to be MSD.

## 4 Discussion

We are the first to explore how geographic heterogeneity and socioeconomic status can influence the potential impact and cost-effectiveness of ETEC and *Shigella* vaccine introduction within countries. Previous studies have demonstrated the importance of considering heterogeneity in ETEC and *Shigella* diarrheal burden and the burden other infectious disease deaths due to ETEC and *Shigella* induced stunting [8], [9] and vaccination for these pathogens in LMICs [11]. Both vaccines were most cost-effective (lower ICERs) in lower and lowest quintiles of higher burden subnational areas in all countries, with few exceptions where middle and higher wealth subpopulation estimates were similar to the lowest two quintiles. Stunting burden from other infectious disease deaths attributed to ETEC and *Shigella* diarrhea contributed between 8–19% and 8–22%, respectively, of the national total burden in four countries. In every country, the vaccines were projected to avert the most deaths in the populations with the highest burden of disease, in some cases, even despite lower vaccination coverage.

Our analysis provides useful information to national and local policymakers as they consider future interventions to reduce diarrheal disease burden. ETEC and *Shigella* vaccines are most cost-effective when the most vulnerable and impoverished populations are vaccinated. Subnational vaccination via the Expanded Programme on Immunization (EPI) is a relatively new concept, but as new vaccines are developed, there is the potential to increase reductions in burden and cost-effectiveness by prioritizing vaccination for the most vulnerable children. Many countries already implement interventions in select areas or high-risk populations [46], [47].

Improving vaccination coverage for all subpopulations to the national average by 2025 would decrease total deaths 16% to 62% in our four study countries. Strategies that show promise for reducing inequity in health care in other settings that could be relevant within these countries are: increasing the number of lay health workers; implementing vaccination campaigns targeting high-risk populations; increasing the number of health facilities where vaccination is given; and investing in transportation and storage capacity [48]. Vaccines can take advantage of existing infrastructure to lower enteric infections in the near term as countries move towards universal coverage in healthcare and in water and sanitation to meet long term goals of reducing diarrheal disease burden [49].

As the relationship between morbidity and long-term consequences are better understood, the cost per MSD episode may be a useful metric to consider when assessing cost-effectiveness. Childhood stunting in childhood has been associated with poor cognitive development that can lead to poorer educational outcomes, reduced wages and increased risk of noncommunicable diseases as adults [50]. An important next step is to include estimates of these longer-term consequences of ETEC and *Shigella* episodes to understand the expanded value of vaccination.

## 5 Limitations

As with any scientific study, there were limitations to our approach. First, we assumed that etiological fractions of disease burden from ETEC and *Shigella* infection were the same across countries and static over the study period due to limited data available on variation in estimates both across and within countries. Variation in estimates of etiological fractions were the third most important on DALY ICERs for both vaccines in all four countries. Second, we were limited to using regional estimates for WHO African Region countries as our incidence rate in morbidity estimates in the absence of published rates estimated at the national or provincial levels for each country. As a result, the differences in ranges of MSD episode ICERs were much smaller between countries and subpopulations than in DALY ICERs. Third, our projections of mortality for diarrheal and other infectious diseases are based on MCEE and IHME trends starting in 2000 or 1990. With vaccination introduction starting in 2025, it is likely that mortality trends could change before or over the 10-year time window. Fourth, our assumptions about shifts in the distribution of stunting due to MSD episodes were based on GEMS study results that included a site in Kenya and sites in three other countries in sub-Saharan Africa. However, our extrapolation of the relationship between diarrheal episodes and stunting to children from countries in this study would benefit by future surveillance and enteric etiology studies to validate or refine our approach. Finally, ETEC and *Shigella* candidate vaccines are still in development and while they are being tested in clinical trials, there is substantial uncertainty around their efficacy and potential pricing. These two parameters were major contributors to uncertainty around predicted outcomes. Another assumption about vaccine efficacy in this model is that efficacy is uniform across geographic and socioeconomic subpopulations within countries. However, there is evidence of lower rotavirus vaccine efficacy in lower income as compared to higher income regions [51]. In the absence of efficacy studies of enteric vaccines with sufficient power to account for variation across subnational or geographic or socioeconomic populations, it is difficult to address in the current model, but should be addressed by future research.

## 6 Conclusion

According to our analyses, ETEC and *Shigella* vaccines show promise for significantly reducing morbidity, mortality, and long-term impacts of ETEC and *Shigella* infection. Our results indicate that considering the subnational variation in ETEC and *Shigella* burden and access to vaccination when designing vaccination programs may lead to higher impact and improved cost-effectiveness of vaccine deployment programs. Childhood ETEC and *Shigella* vaccines will be critical tools to reducing disease burden and inequality, but funding and effective programs aimed at improving access to EPI vaccination for the underserved would vastly improve overall impact of these vaccines in all four countries. As vaccination coverage is variable across the geographic and socioeconomic populations within countries, achieving universal coverage of ETEC and *Shigella* and other EPI vaccines will require strategies tailored to the needs of each population.

## Declaration of Competing Interest

The authors declare that they have no known competing financial interests or personal relationships that could have appeared to influence the work reported in this paper.
