# Supplementary material for: Heterogeneity in potential impact and cost-effectiveness of ETEC and Shigella vaccination in four sub-Saharan African countries
Source: Vaccine X. 2019 Sep 20;3:100043. doi: 10.1016/j.jvacx.2019.100043 (PMC6819873; doi:10.1016/j.jvacx.2019.100043)

**Supplemental Figures**

**Supplemental Figure 1.** Threshold analysis of Incremental Cost-Effectiveness Ratios (ICERs) generated from simulation results for quintile and regional (overall) subpopulations within Zimbabwe, projected for the first 10 years after introduction (2025-2034). The fraction of ICERs from 10,000 iterations that fell below national Gross Domestic Product (GDP) thresholds are presented as percentages. Thresholds from 0.25 to 4 times GDP were included in the analysis.


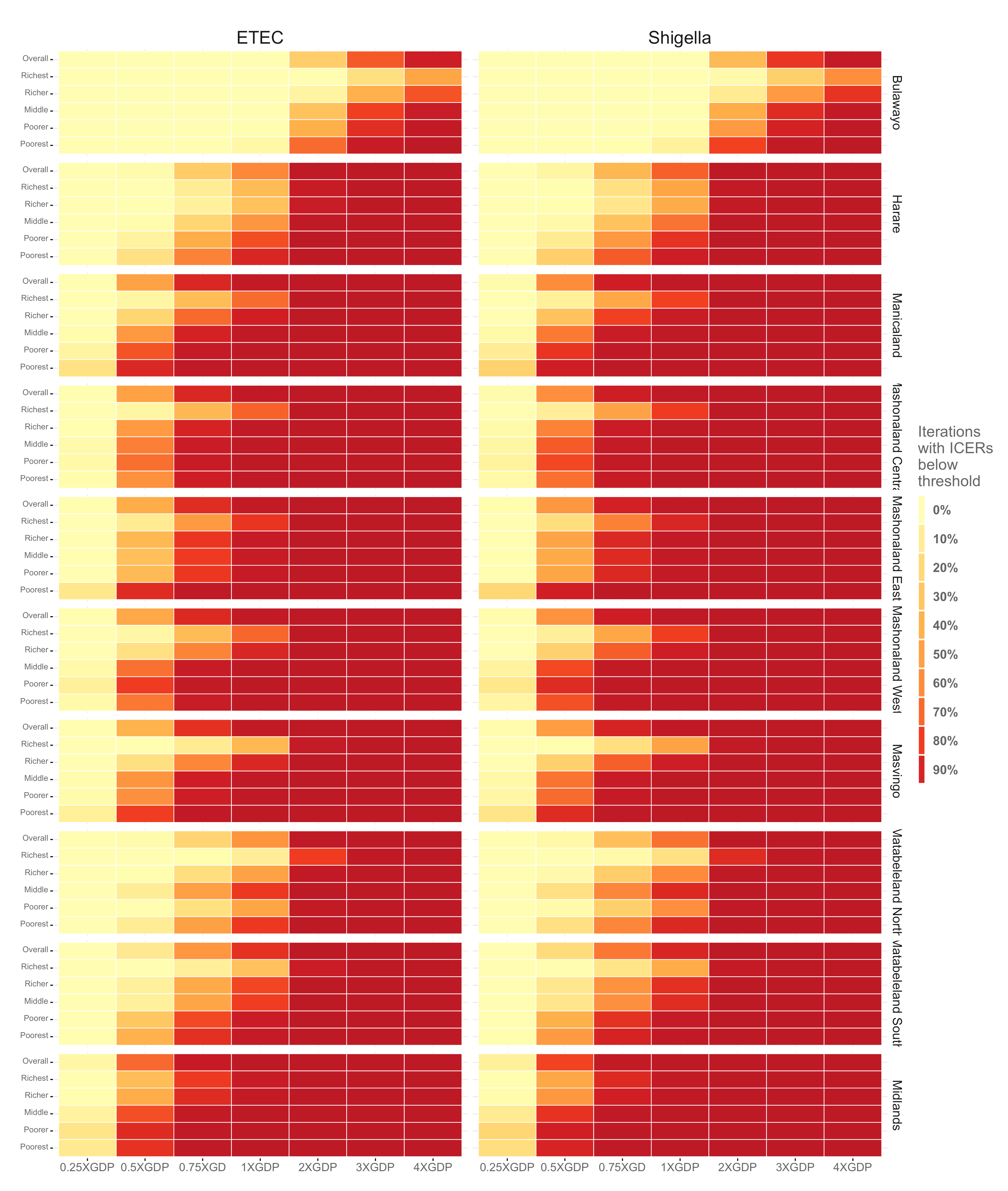


**Supplemental Figure 2.** Threshold analysis of Incremental Cost-Effectiveness Ratios (ICERs) generated from simulation results for quintile and regional (overall) subpopulations within the Democratic Republic of Congo, projected for the first 10 years after introduction (2025-2034). The fraction of ICERs from 10,000 iterations that fell below national Gross Domestic Product (GDP) thresholds are presented as percentages. Thresholds from 0.25 to 4 times GDP were included in the analysis.


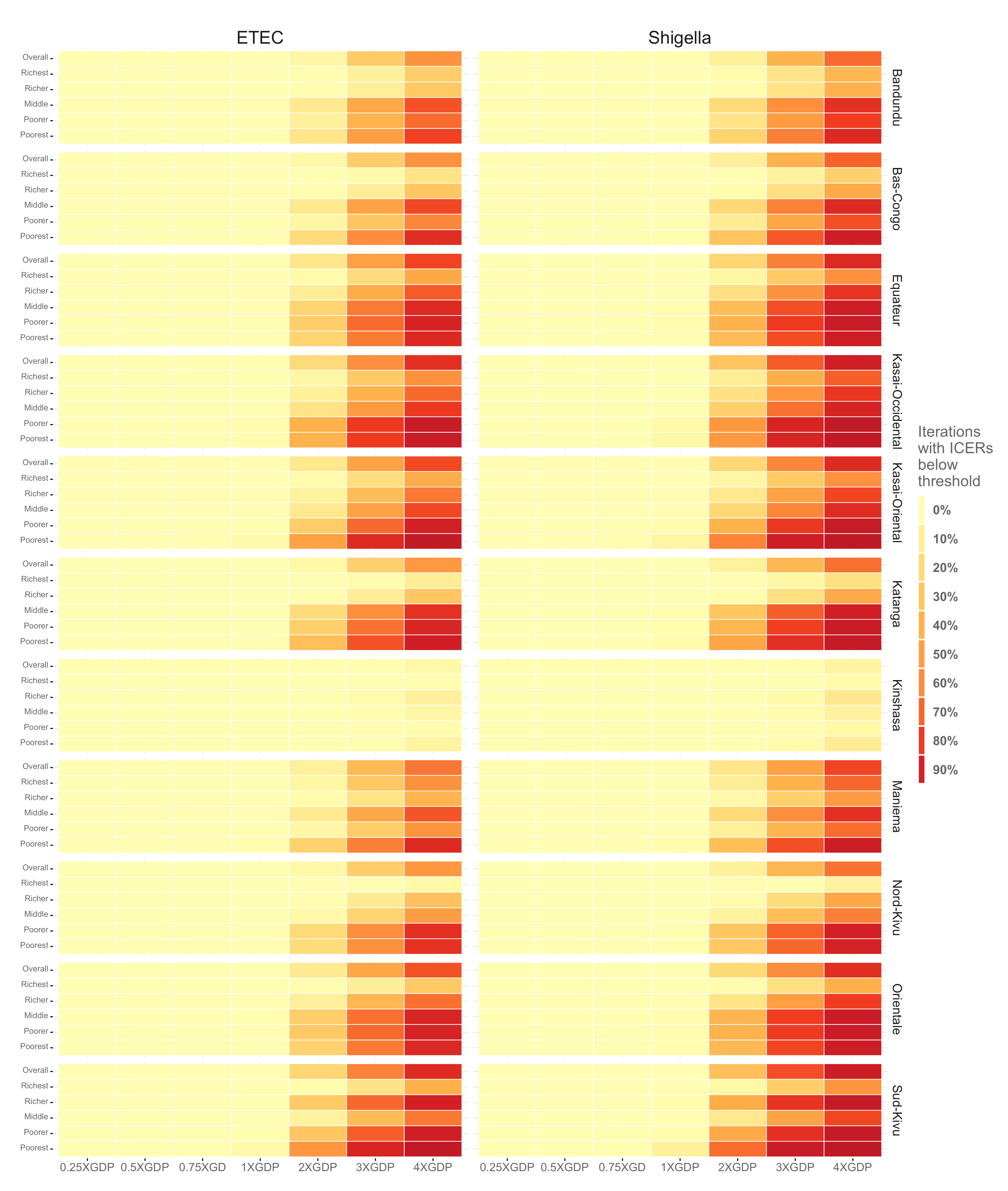


**Supplemental Figure 3.** Threshold analysis of Incremental Cost-Effectiveness Ratios (ICERs) generated from simulation results for quintile and regional (overall) subpopulations within Kenya, projected for the first 10 years after introduction (2025-2034). The fraction of ICERs from 10,000 iterations that fell below national Gross Domestic Product (GDP) thresholds are presented as percentages. Thresholds from 0.25 to 4 times GDP were included in the analysis.


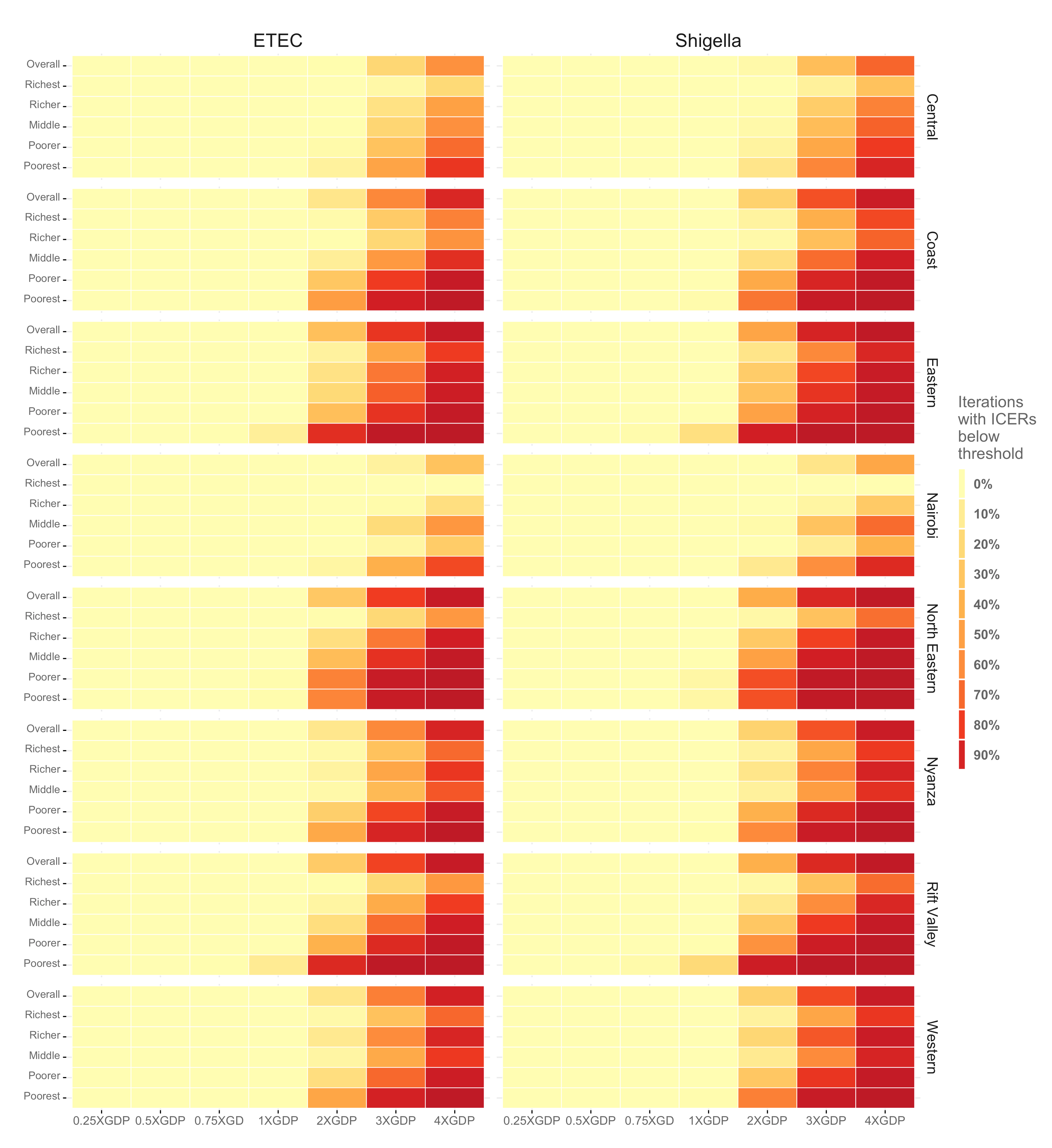


**Supplemental Figure 4.** Threshold analysis of Incremental Cost-Effectiveness Ratios (ICERs) generated from simulation results for quintile and regional (overall) subpopulations within Zambia, projected for the first 10 years after introduction (2025-2034). The fraction of ICERs from 10,000 iterations that fell below national Gross Domestic Product (GDP) thresholds are presented as percentages. Thresholds from 0.25 to 4 times GDP were included in the analysis.


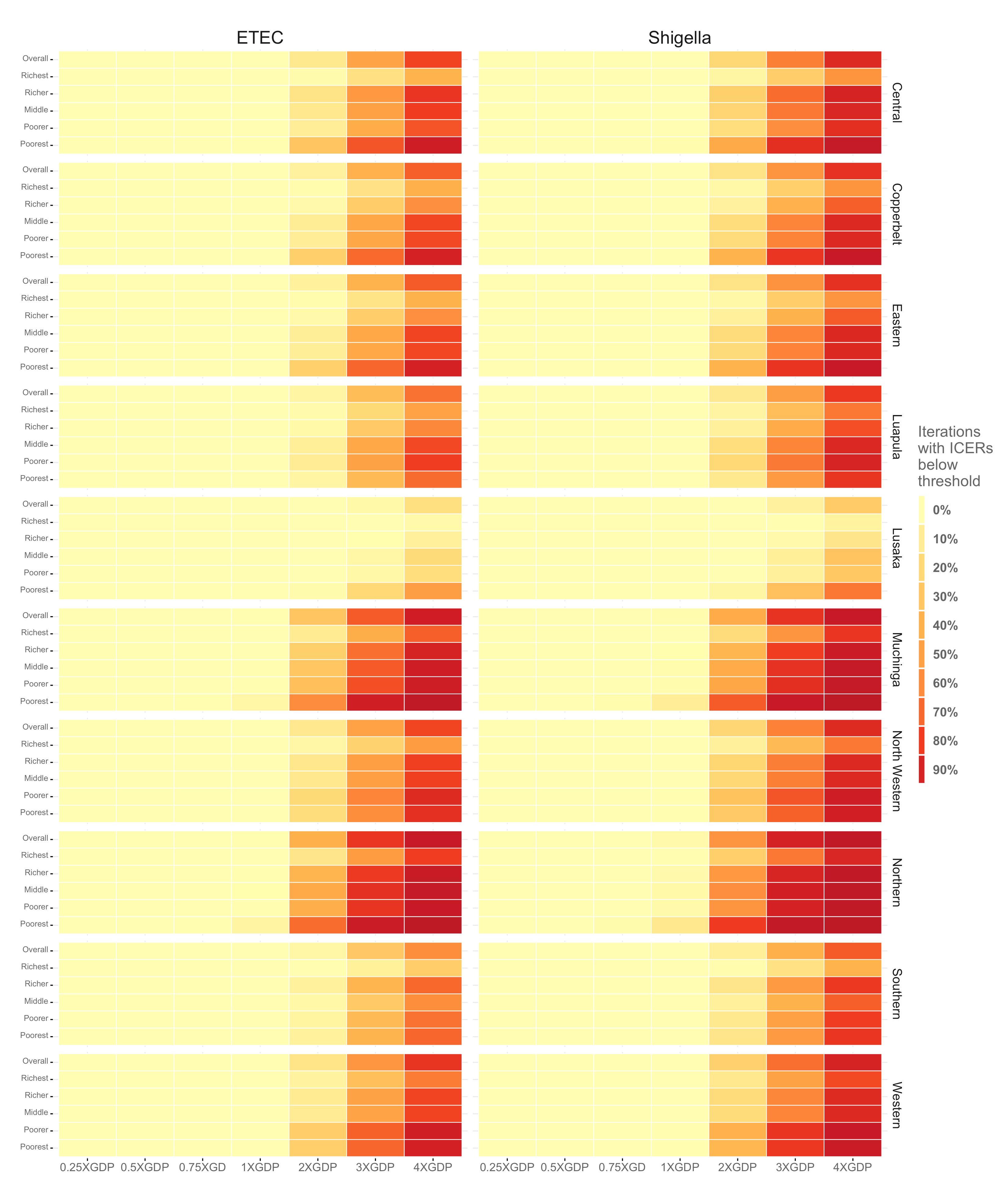

Supplement: Supplementary data 1 [file mmc1.docx]
