# Supplementary material for: Heterogeneity in potential impact and cost-effectiveness of ETEC and Shigella vaccination in four sub-Saharan African countries
Source: Vaccine X. 2019 Sep 20;3:100043. doi: 10.1016/j.jvacx.2019.100043 (PMC6819873; doi:10.1016/j.jvacx.2019.100043)
Supplement: Supplementary data 2 [file mmc2.docx]

**Supplemental Tables**

**Supplemental Table 1.** Logistic regression and linear regression results for receiving home or packaged oral rehydration therapy (ORT) and medical costs of treating diarrhea, respectively*.* Brackets indicate 95% confidence intervals for each estimate. *P*-values less than alpha = 0.05 are indicated with a “*”.

| DRC | Dependent variable | |
| --- | --- | --- |
|  | Receiving any ORT | Medical costs of diarrhea |
| Independent variables | Odds ratio | Beta |
| Wealth quintiles |  |  |
| Lowest | 1.00 | - |
| Lower | 1.12 [0.74, 1.71] | -0.21 [-0.43, 0.01] |
| Middle | 1.50 [0.97, 2.31] | 0.02 [-0.24, 0.29] |
| Higher | 1.06 [0.69, 1.61] | 0.05 [-0.20, 0.30] |
| Highest | 0.92 [0.50, 1.69] | 0.52* [0.15, 0.89] |
| Setting |  |  |
| Urban | 1.00 | - |
| Rural | 0.74 [0.54, 1.00] | -0.03 [-0.30, 0.24] |
| Sex of child |  |  |
| Male | 1.00 | - |
| Female | 1.06 [0.86, 1.30] | - |
| Maternal education |  |  |
| None | 1.00 | - |
| Primary | 1.12 [0.80, 1.57] | - |
| Secondary or above | 1.42 [0.95, 2.12] | - |
| Age of child |  |  |
| < 1 yr old | 1.00 | - |
| 1 yr old | 1.57* [1.15, 2.14] | - |
| 2 yrs old | 1.11 [0.78, 1.59] | - |
| 3 yrs old | 1.21 [0.81, 1.80] | - |
| 4 yrs old | 1.11 [0.73, 1.70] | - |
| Province |  |  |
| Bandundu | 1.00 | - |
| Bas-Congo | 1.52 [0.81, 2.85] | 0.10 [-0.24, 0.45] |
| Equateur | 0.48* [0.29, 0.78] | 0.03 [-0.26, 0.32] |
| Kasai-Occidental | 0.90 [0.53, 1.53] | -0.06 [-0.29, 0.17] |
| Kasai-Oriental | 0.67 [0.41, 1.10] | 0.04 [-0.22, 0.30] |
| Katanga | 0.66 [0.40, 1.11] | 0.25 [-0.08, 0.58] |
| Kinshasa | 0.60 [0.32, 1.13] | 0.90* [0.36, 1.44] |
| Maniema | 1.15 [0.71, 1.85] | 0.67 [-0.29, 1.62] |
| Nord-Kivu | 0.67 [0.34, 1.31] | 0.18 [-0.27, 0.63] |
| Orientale | 0.46* [0.27, 0.81] | 0.71 [-0.12, 1.53] |
| Sud-Kivu | 0.70 [0.39, 1.26] | 0.34 [-0.04, 0.72] |
| Constant | 0.76 [0.40, 1.42] | 0.36 [0.00, 0.72] |
| Model tests |  |  |
| F | 3.15 | 3.02 |
| Prob > F | <0.00001 | 0.0001 |
| R squared | - | 0.05 |
|  |  |  |

| Kenya | Dependent variable | |
| --- | --- | --- |
|  | Receiving any ORT | Medical costs of diarrhea |
| Independent variables | Odds ratio | Beta |
| Wealth quintiles |  |  |
| Lowest | 1.00 | - |
| Lower | 1.48* [1.09, 2.01] | -0.02 [-2.31, 2.27] |
| Middle | 1.33 [0.97, 1.83] | -0.65 [-2.87, 1.57] |
| Higher | 1.05 [0.73, 1.51] | 1.71 [-0.95, 4.37] |
| Highest | 0.84 [0.54, 1.31] | 6.55* [3.31, 9.79] |
| Setting |  |  |
| Urban | 1.00 | - |
| Rural | 0.89 [0.69, 1.16] | -3.88* [-5.99, -1.77] |
| Sex of child |  |  |
| Male | 1.00 | - |
| Female | 0.82* [0.67, 0.99] | - |
| Maternal education |  |  |
| None | 1.00 | - |
| Primary | 1.11 [0.78, 1.59] | - |
| Secondary or above | 1.42 [0.92, 2.18] | - |
| Age of child |  |  |
| < 1 yr old | 1.00 | - |
| 1 yr old | 1.48* [1.15, 1.91] | - |
| 2 yrs old | 0.97 [0.73, 1.29] | - |
| 3 yrs old | 1.25 [0.90, 1.73] | - |
| 4 yrs old | 0.75 [0.51, 1.11] | - |
| Province |  |  |
| Central | 1.00 | - |
| Coast | 1.51 [0.88, 2.62] | -3.09 [-7.56, 1.39] |
| Eastern | 0.83 [0.49, 1.42] | -3.22 [-7.87, 1.42] |
| Nairobi | 1.31 [0.67, 2.55] | -2.91 [-8.62, 2.80] |
| North Eastern | 1.85 [0.82, 4.17] | -4.75 [-10.76, 1.25] |
| Nyanza | 0.82 [0.49, 1.37] | -7.12* [-11.24, -3.00] |
| Rift Valley | 1.16 [0.71, 1.89] | -3.62 [-7.77, 0.53] |
| Western | 0.79 [0.47, 1.32] | -7.30* [-11.61, -2.99] |
| Constant | 1.43 [0.73, 2.81] | 14.36* [9.81, 18.92] |
| Model tests |  |  |
| F | 2.73 | 7.65 |
| Prob > F | 0.0001 | <0.00001 |
| R squared | - | 0.07 |
|  |  |  |

| Zambia | Dependent variable | |
| --- | --- | --- |
|  | Receiving any ORT | Medical costs of diarrhea |
| Independent variables | Odds ratio | Beta |
| Wealth quintiles |  |  |
| Lowest | 1.00 | - |
| Lower | 0.93 [0.66, 1.32] | 0.01 [-1.43, 1.46] |
| Middle | 0.89 [0.64, 1.26] | -0.56 [-1.88, 0.76] |
| Higher | 1.43 [0.88, 2.30] | -0.29 [-1.74, 1.17] |
| Highest | 1.17 [0.64, 2.15] | 4.56* [1.63, 7.49] |
| Setting |  |  |
| Urban | 1.00 | - |
| Rural | 1.11 [0.79, 1.56] | -4.42* [-6.66, -2.18] |
| Sex of child |  |  |
| Male | 1.00 | - |
| Female | 1.02 [0.82, 1.28] | - |
| Maternal education |  |  |
| None | 1.00 | - |
| Primary | 1.98* [1.39, 2.83] | - |
| Secondary or above | 1.86* [1.23, 2.82] | - |
| Age of child |  |  |
| < 1 yr old | 1.00 | - |
| 1 yr old | 1.45* [1.06, 2.00] | - |
| 2 yrs old | 1.00 [0.68, 1.45] | - |
| 3 yrs old | 0.80 [0.54, 1.20] | - |
| 4 yrs old | 0.96 [0.58, 1.60] | - |
| Province |  |  |
| Central | 1.00 | - |
| Copperbelt | 1.04 [0.60, 1.82] | -3.22 [-7.02, 0.58] |
| Eastern | 1.14 [0.65, 2.01] | -2.82* [-5.57, -0.07] |
| Luapula | 1.94* [1.10, 3.42] | -4.41* [-6.98, -1.84] |
| Lusaka | 1.56 [0.82, 2.99] | -6.06* [-9.78, -2.34] |
| Muchinga | 0.70 [0.39, 1.27] | -0.25 [-3.58, 3.08] |
| Northern | 0.74 [0.42, 1.31] | -2.45 [-5.17, 0.28] |
| North Western | 0.94 [0.55, 1.61] | 0.62 [-2.97, 4.21] |
| Southern | 1.15 [0.62, 2.11] | -1.81 [-4.90, 1.27] |
| Western | 1.14 [0.61, 2.12] | -2.58 [-5.53, 0.38] |
| Constant | 0.99 [0.50, 1.98] |  |
| Model tests |  |  |
| F | 2.53 | 5.76 |
| Prob > F | 0.0002 | <0.00001 |
| R squared | - | 0.07 |
|  |  |  |

| Zimbabwe | Dependent variable | |
| --- | --- | --- |
|  | Receiving any ORT | Medical costs of diarrhea |
| Independent variables | Odds ratio | Beta |
| Wealth quintiles |  |  |
| Lowest | 1.00 | - |
| Lower | 0.86 [0.54, 1.38] | 0.54 [-0.61, 1.70] |
| Middle | 1.07 [0.64, 1.79] | -0.06 [-0.90, 0.78] |
| Higher | 1.38 [0.78, 2.44] | 0.86 [-0.10, 1.82] |
| Highest | 0.96 [0.46, 2.02] | 2.73* [0.89, 4.58] |
| Setting |  |  |
| Urban | 1.00 | - |
| Rural | 1.49 [0.80, 2.79] | 0.24 [-1.12, 1.60] |
| Sex of child |  |  |
| Male | 1.00 | - |
| Female | 1.03 [0.74, 1.42] | - |
| Maternal education |  |  |
| None | 1.00 | - |
| Primary | 0.52 [0.17, 1.62] | - |
| Secondary or above | 0.91 [0.29, 2.89] | - |
| Age of child |  |  |
| < 1 yr old | 1.00 | - |
| 1 yr old | 2.41* [1.58, 3.69] | - |
| 2 yrs old | 2.90* [1.71, 4.92] | - |
| 3 yrs old | 1.72 [0.93, 3.18] | - |
| 4 yrs old | 2.47* [1.30, 4.68] | - |
| Province |  |  |
| Manicaland | 1.00 | - |
| Mashonaland Central | 1.25 [0.68, 2.29] | -1.29 [-2.94, 0.35] |
| Mashonaland East | 0.80 [0.36, 1.78] | -0.65 [-2.45, 1.15] |
| Mashonaland West | 1.17 [0.66, 2.07] | -1.09 [-2.74, 0.56] |
| Matabeleland North | 3.12* [1.35, 7.18] | 2.17 [-0.24, 4.58] |
| Matabeleland South | 2.10 [0.75, 5.92] | 0.05 [-1.86, 1.97] |
| Midlands | 0.78 [0.44, 1.38] | -0.92 [-2.63, 0.78] |
| Masvingo | 0.98 [0.53, 1.81] | 0.08 [-2.13, 2.30] |
| Harare | 1.50 [0.76, 2.97] | 0.36 [-1.63, 2.34] |
| Bulawayo | 4.38* [1.60, 11.98] | 1.17 [-1.57, 3.91] |
| Constant | 1.01 [0.32, 3.21] | 1.32 [-0.68, 3.32] |
| Model tests |  |  |
| F | 3.02 | 3.82 |
| Prob > F | <0.00001 | <0.00001 |
| R squared | - | 0.06 |
|  |  |  |

**Supplemental Table 2.** Regional and quintile estimates of ETEC and *Shigella* morbidity, mortality and economic costs projected for 2025-2034 in four East African countries. All costs are presented in 2016 US$. Democratic Republic of Congo is abbreviated as ‘DRC'.

|  | MSD Episodes (1000s) | | MSD Episodes / 100,000 children | | Moderate and Severe Stunting Cases Due to MSD Diarrhoea (1000s) | | Moderate and Severe Stunting Cases Due to MSD Diarrhoea / 100,000 Children | | Direct Diarrhoeal Deaths | | Direct Diarrhoeal Deaths / 100,000 Children | | Other ID Deaths Due to Diarrhoea-induced Stunting | | Other ID Deaths Due to Diarrhoea-induced Stunting / 100,000 Children | | Direct Diarrhoeal and Other ID mortality due to Diarrhoea-induced Stunting (Total Deaths) | | Total Deaths / 100,000 Children | | Total DALYs (1000s) | | ETEC Direct Medical Costs (2016 US$) / 100,000 children | |
| --- | --- | --- | --- | --- | --- | --- | --- | --- | --- | --- | --- | --- | --- | --- | --- | --- | --- | --- | --- | --- | --- | --- | --- | --- |
|  | ETEC | *Shigella* | ETEC | *Shigella* | ETEC | *Shigella* | ETEC | *Shigella* | ETEC | *Shigella* | ETEC | *Shigella* | ETEC | *Shigella* | ETEC | *Shigella* | ETEC | *Shigella* | ETEC | *Shigella* | ETEC | *Shigella* | ETEC | *Shigella* |
| DRC |  |  |  |  |  |  |  |  |  |  |  |  |  |  |  |  |  |  |  |  |  |  |  |  |
| Sud-Kivu | 229 [107, 436] | 250 [118, 473] | 2,177 [1,015, 4,153] | 2,380 [1,121, 4,499] | 49 [23, 95] | 65 [30, 124] | 467 [217, 906] | 615 [289, 1,178] | 1,337 [703, 2,334] | 1,462 [773, 2,572] | 13 [7, 22] | 14 [7, 24] | 390 [173, 772] | 511 [230, 1,004] | 3.7 [1.7, 7.3] | 4.9 [2.2, 9.6] | 1,727 [977, 2,913] | 1,972 [1,110, 3,320] | 16 [9, 28] | 19 [11, 32] | 60 [34, 101] | 68 [39, 115] | 13,021 [5,899, 25,936] | 14,236 [6,498, 28,044] |
| Lowest | 47 [22, 89] | 51 [24, 96] | 2,222 [1,036, 4,240] | 2,430 [1,144, 4,594] | 12 [5, 23] | 15 [7, 30] | 558 [258, 1,082] | 736 [345, 1,408] | 385 [203, 673] | 421 [223, 742] | 18 [10, 32] | 20 [11, 35] | 100 [44, 197] | 130 [59, 256] | 4.7 [2.1, 9.4] | 6.2 [2.8, 12.2] | 485 [273, 818] | 552 [310, 932] | 23 [13, 39] | 26 [15, 44] | 17 [9, 28] | 19 [11, 32] | 11,670 [5,287, 23,245] | 12,759 [5,823, 25,134] |
| Lower | 47 [22, 90] | 51 [24, 97] | 2,234 [1,042, 4,262] | 2,443 [1,150, 4,618] | 12 [5, 23] | 16 [7, 30] | 564 [261, 1,094] | 744 [349, 1,424] | 286 [150, 499] | 313 [165, 550] | 14 [7, 24] | 15 [8, 26] | 100 [45, 198] | 131 [59, 258] | 4.8 [2.1, 9.4] | 6.3 [2.8, 12.3] | 386 [219, 650] | 444 [251, 743] | 18 [10, 31] | 21 [12, 35] | 13 [8, 23] | 15 [9, 26] | 7,958 [3,606, 15,852] | 8,701 [3,971, 17,140] |
| Middle | 47 [22, 89] | 51 [24, 96] | 2,214 [1,032, 4,224] | 2,421 [1,140, 4,577] | 8 [4, 16] | 11 [5, 20] | 384 [178, 745] | 505 [237, 967] | 227 [119, 396] | 248 [131, 436] | 11 [6, 19] | 12 [6, 21] | 43 [19, 86] | 56 [25, 112] | 2.1 [0.9, 4.1] | 2.7 [1.2, 5.3] | 270 [150, 458] | 304 [169, 518] | 13 [7, 22] | 14 [8, 25] | 9 [5, 16] | 11 [6, 18] | 12,043 [5,456, 23,988] | 13,167 [6,010, 25,938] |
| Higher | 45 [21, 87] | 50 [23, 94] | 2,159 [1,007, 4,119] | 2,361 [1,112, 4,463] | 11 [5, 22] | 15 [7, 28] | 538 [249, 1,043] | 708 [332, 1,356] | 279 [147, 488] | 305 [161, 537] | 13 [7, 23] | 15 [8, 26] | 97 [43, 191] | 127 [57, 248] | 4.6 [2.0, 9.1] | 6.0 [2.7, 11.8] | 376 [213, 633] | 432 [244, 724] | 18 [10, 30] | 21 [12, 34] | 13 [7, 22] | 15 [8, 25] | 12,496 [5,662, 24,891] | 13,663 [6,236, 26,914] |
| Highest | 43 [20, 82] | 47 [22, 89] | 2,054 [958, 3,919] | 2,246 [1,058, 4,246] | 6 [3, 12] | 8 [4, 15] | 293 [136, 568] | 384 [180, 734] | 160 [84, 278] | 174 [92, 307] | 8 [4, 13] | 8 [4, 15] | 50 [22, 100] | 66 [30, 129] | 2.4 [1.1, 4.7] | 3.1 [1.4, 6.2] | 210 [119, 353] | 240 [135, 404] | 10 [6, 17] | 11 [6, 19] | 7 [4, 12] | 8 [5, 14] | 20,937 [9,486, 41,704] | 22,891 [10,448, 45,094] |
| Orientale | 850 [402, 1,597] | 929 [438, 1,732] | 2,120 [1,003, 3,984] | 2,317 [1,091, 4,319] | 168 [78, 322] | 220 [103, 417] | 418 [194, 803] | 549 [257, 1,040] | 4,811 [2,530, 8,491] | 5,261 [2,771, 9,267] | 12 [6, 21] | 13 [7, 23] | 1,067 [477, 2,127] | 1,394 [625, 2,700] | 2.7 [1.2, 5.3] | 3.5 [1.6, 6.7] | 5,879 [3,268, 10,078] | 6,655 [3,727, 11,372] | 15 [8, 25] | 17 [9, 28] | 204 [113, 351] | 231 [129, 395] | 19,476 [8,878, 38,347] | 21,293 [9,709, 41,570] |
| Lowest | 178 [84, 334] | 194 [92, 362] | 2,217 [1,049, 4,166] | 2,424 [1,141, 4,517] | 44 [20, 84] | 57 [27, 109] | 543 [252, 1,045] | 715 [335, 1,354] | 1,037 [545, 1,830] | 1,134 [597, 1,998] | 13 [7, 23] | 14 [7, 25] | 309 [138, 615] | 404 [182, 781] | 3.9 [1.7, 7.7] | 5.0 [2.3, 9.7] | 1,346 [754, 2,300] | 1,538 [865, 2,619] | 17 [9, 29] | 19 [11, 33] | 47 [26, 80] | 53 [30, 91] | 18,083 [8,243, 35,604] | 19,770 [9,014, 38,597] |
| Lower | 173 [82, 324] | 189 [89, 352] | 2,153 [1,019, 4,046] | 2,354 [1,108, 4,386] | 31 [14, 60] | 41 [19, 78] | 389 [181, 747] | 511 [239, 967] | 1,234 [649, 2,178] | 1,349 [711, 2,377] | 15 [8, 27] | 17 [9, 30] | 184 [82, 367] | 240 [107, 465] | 2.3 [1.0, 4.6] | 3.0 [1.3, 5.8] | 1,418 [779, 2,442] | 1,589 [877, 2,734] | 18 [10, 30] | 20 [11, 34] | 49 [27, 85] | 55 [31, 95] | 14,391 [6,560, 28,334] | 15,734 [7,174, 30,716] |
| Middle | 172 [81, 323] | 188 [89, 351] | 2,146 [1,016, 4,033] | 2,346 [1,105, 4,373] | 37 [17, 71] | 49 [23, 92] | 463 [215, 890] | 609 [285, 1,152] | 1,139 [599, 2,010] | 1,245 [656, 2,193] | 14 [7, 25] | 16 [8, 27] | 243 [109, 485] | 318 [142, 615] | 3.0 [1.4, 6.0] | 4.0 [1.8, 7.7] | 1,382 [767, 2,371] | 1,563 [874, 2,672] | 17 [10, 30] | 19 [11, 33] | 48 [27, 82] | 54 [30, 93] | 18,509 [8,437, 36,443] | 20,236 [9,227, 39,507] |
| Higher | 169 [80, 318] | 185 [87, 344] | 2,107 [997, 3,959] | 2,303 [1,085, 4,293] | 33 [15, 63] | 43 [20, 82] | 410 [190, 787] | 538 [252, 1,019] | 837 [440, 1,477] | 915 [482, 1,612] | 10 [5, 18] | 11 [6, 20] | 200 [89, 399] | 261 [117, 506] | 2.5 [1.1, 5.0] | 3.3 [1.5, 6.3] | 1,037 [576, 1,777] | 1,176 [660, 2,009] | 13 [7, 22] | 15 [8, 25] | 36 [20, 62] | 41 [23, 70] | 18,961 [8,643, 37,333] | 20,731 [9,452, 40,472] |
| Highest | 158 [75, 298] | 173 [82, 323] | 1,976 [936, 3,714] | 2,161 [1,018, 4,027] | 23 [11, 44] | 30 [14, 57] | 285 [132, 547] | 373 [175, 705] | 565 [297, 997] | 618 [325, 1,088] | 7 [4, 12] | 8 [4, 14] | 131 [59, 262] | 171 [76, 331] | 1.6 [0.7, 3.3] | 2.1 [1.0, 4.1] | 696 [387, 1,194] | 789 [442, 1,348] | 9 [5, 15] | 10 [6, 17] | 24 [13, 41] | 27 [15, 47] | 27,435 [12,506, 54,019] | 29,996 [13,676, 58,560] |
| Nord-Kivu | 295 [138, 555] | 323 [152, 606] | 2,060 [959, 3,873] | 2,252 [1,058, 4,225] | 62 [29, 117] | 81 [38, 157] | 429 [201, 817] | 565 [264, 1,092] | 1,402 [736, 2,459] | 1,532 [810, 2,696] | 10 [5, 17] | 11 [6, 19] | 278 [123, 547] | 363 [163, 716] | 1.9 [0.9, 3.8] | 2.5 [1.1, 5.0] | 1,680 [929, 2,844] | 1,896 [1,042, 3,212] | 12 [6, 20] | 13 [7, 22] | 58 [32, 99] | 66 [36, 112] | 10,310 [4,683, 20,418] | 11,272 [5,170, 22,322] |
| Lowest | 60 [28, 113] | 66 [31, 124] | 2,102 [979, 3,952] | 2,298 [1,080, 4,311] | 15 [7, 29] | 20 [9, 39] | 531 [248, 1,012] | 700 [327, 1,353] | 386 [203, 677] | 422 [223, 742] | 13 [7, 24] | 15 [8, 26] | 74 [33, 146] | 97 [44, 192] | 2.6 [1.2, 5.1] | 3.4 [1.5, 6.7] | 460 [254, 779] | 519 [285, 879] | 16 [9, 27] | 18 [10, 31] | 16 [9, 27] | 18 [10, 31] | 8,853 [4,022, 17,533] | 9,679 [4,440, 19,168] |
| Lower | 61 [29, 115] | 67 [32, 126] | 2,139 [996, 4,020] | 2,338 [1,099, 4,386] | 15 [7, 29] | 20 [10, 39] | 538 [251, 1,025] | 709 [331, 1,370] | 392 [206, 688] | 428 [227, 754] | 14 [7, 24] | 15 [8, 26] | 76 [34, 149] | 99 [44, 195] | 2.6 [1.2, 5.2] | 3.5 [1.5, 6.8] | 468 [258, 791] | 527 [290, 893] | 16 [9, 28] | 18 [10, 31] | 16 [9, 28] | 18 [10, 31] | 5,121 [2,326, 10,141] | 5,599 [2,568, 11,087] |
| Middle | 58 [27, 110] | 64 [30, 120] | 2,035 [948, 3,825] | 2,224 [1,045, 4,172] | 13 [6, 25] | 17 [8, 33] | 451 [211, 859] | 594 [277, 1,148] | 267 [140, 468] | 292 [154, 513] | 9 [5, 16] | 10 [5, 18] | 56 [25, 111] | 73 [33, 145] | 2.0 [0.9, 3.9] | 2.6 [1.1, 5.1] | 323 [179, 546] | 365 [201, 618] | 11 [6, 19] | 13 [7, 22] | 11 [6, 19] | 13 [7, 21] | 9,275 [4,213, 18,368] | 10,140 [4,651, 20,080] |
| Higher | 58 [27, 109] | 63 [30, 119] | 2,019 [940, 3,796] | 2,208 [1,037, 4,141] | 12 [6, 23] | 16 [8, 31] | 426 [199, 810] | 559 [261, 1,081] | 214 [112, 376] | 234 [124, 412] | 7 [4, 13] | 8 [4, 14] | 55 [24, 108] | 72 [32, 142] | 1.9 [0.8, 3.8] | 2.5 [1.1, 4.9] | 269 [150, 453] | 306 [169, 517] | 9 [5, 16] | 11 [6, 18] | 9 [5, 16] | 11 [6, 18] | 9,876 [4,487, 19,560] | 10,798 [4,953, 21,384] |
| Highest | 58 [27, 108] | 63 [30, 118] | 2,006 [934, 3,771] | 2,193 [1,031, 4,114] | 6 [3, 11] | 8 [4, 15] | 201 [94, 383] | 263 [123, 508] | 143 [75, 251] | 156 [83, 275] | 5 [3, 9] | 5 [3, 10] | 17 [7, 33] | 22 [10, 43] | 0.6 [0.3, 1.1] | 0.8 [0.3, 1.5] | 160 [87, 273] | 178 [97, 305] | 6 [3, 10] | 6 [3, 11] | 6 [3, 10] | 6 [3, 11] | 18,424 [8,369, 36,487] | 20,143 [9,239, 39,890] |
| Maniema | 117 [55, 220] | 127 [61, 239] | 2,100 [991, 3,959] | 2,296 [1,091, 4,304] | 21 [10, 40] | 27 [13, 52] | 376 [174, 715] | 493 [230, 938] | 604 [316, 1,055] | 660 [346, 1,151] | 11 [6, 19] | 12 [6, 21] | 113 [50, 221] | 148 [67, 286] | 2.0 [0.9, 4.0] | 2.7 [1.2, 5.2] | 717 [398, 1,214] | 808 [452, 1,356] | 13 [7, 22] | 15 [8, 24] | 25 [14, 42] | 28 [16, 47] | 18,850 [8,571, 36,825] | 20,610 [9,476, 40,245] |
| Lowest | 24 [11, 44] | 26 [12, 48] | 2,120 [1,001, 3,997] | 2,318 [1,101, 4,345] | 5 [2, 9] | 6 [3, 12] | 423 [196, 805] | 555 [259, 1,057] | 161 [85, 282] | 177 [93, 308] | 15 [8, 25] | 16 [8, 28] | 26 [12, 51] | 34 [15, 66] | 2.3 [1.0, 4.6] | 3.1 [1.4, 5.9] | 187 [104, 318] | 210 [117, 355] | 17 [9, 29] | 19 [11, 32] | 7 [4, 11] | 7 [4, 12] | 17,437 [7,929, 34,065] | 19,065 [8,765, 37,229] |
| Lower | 23 [11, 43] | 25 [12, 46] | 2,036 [961, 3,838] | 2,226 [1,057, 4,172] | 4 [2, 7] | 5 [2, 9] | 339 [157, 645] | 445 [208, 845] | 111 [58, 195] | 122 [64, 212] | 10 [5, 18] | 11 [6, 19] | 20 [9, 39] | 26 [12, 50] | 1.8 [0.8, 3.5] | 2.3 [1.1, 4.5] | 131 [73, 223] | 148 [82, 248] | 12 [7, 20] | 13 [7, 22] | 5 [3, 8] | 5 [3, 9] | 13,701 [6,229, 26,765] | 14,979 [6,887, 29,251] |
| Middle | 24 [11, 44] | 26 [12, 48] | 2,125 [1,003, 4,007] | 2,324 [1,104, 4,355] | 5 [2, 9] | 6 [3, 12] | 431 [200, 820] | 566 [264, 1,076] | 128 [67, 224] | 140 [74, 245] | 12 [6, 20] | 13 [7, 22] | 28 [13, 55] | 37 [17, 71] | 2.5 [1.1, 5.0] | 3.3 [1.5, 6.4] | 157 [88, 265] | 177 [100, 297] | 14 [8, 24] | 16 [9, 27] | 5 [3, 9] | 6 [3, 10] | 17,851 [8,117, 34,874] | 19,517 [8,973, 38,112] |
| Higher | 24 [11, 45] | 26 [12, 48] | 2,127 [1,004, 4,010] | 2,326 [1,105, 4,360] | 4 [2, 8] | 5 [3, 10] | 368 [171, 701] | 483 [226, 919] | 90 [47, 157] | 98 [52, 171] | 8 [4, 14] | 9 [5, 15] | 21 [9, 41] | 27 [12, 53] | 1.9 [0.8, 3.7] | 2.5 [1.1, 4.8] | 111 [62, 187] | 126 [71, 210] | 10 [6, 17] | 11 [6, 19] | 4 [2, 7] | 4 [2, 7] | 18,373 [8,354, 35,893] | 20,088 [9,236, 39,227] |
| Highest | 23 [11, 44] | 25 [12, 48] | 2,091 [987, 3,942] | 2,286 [1,086, 4,286] | 4 [2, 7] | 5 [2, 9] | 318 [147, 605] | 417 [194, 792] | 113 [59, 197] | 123 [65, 215] | 10 [5, 18] | 11 [6, 19] | 18 [8, 35] | 24 [11, 46] | 1.6 [0.7, 3.2] | 2.1 [1.0, 4.1] | 131 [72, 222] | 147 [82, 247] | 12 [7, 20] | 13 [7, 22] | 5 [3, 8] | 5 [3, 9] | 26,889 [12,226, 52,529] | 29,398 [13,516, 57,407] |
| Kinshasa | 423 [179, 735] | 462 [196, 801] | 1,964 [833, 3,417] | 2,148 [911, 3,723] | 30 [12, 53] | 39 [16, 68] | 137 [58, 245] | 180 [76, 316] | 1,093 [535, 1,767] | 1,195 [593, 1,957] | 5 [2, 8] | 6 [3, 9] | 71 [30, 128] | 93 [38, 165] | 0.3 [0.1, 0.6] | 0.4 [0.2, 0.8] | 1,165 [592, 1,861] | 1,288 [668, 2,069] | 5 [3, 9] | 6 [3, 10] | 41 [21, 65] | 45 [23, 72] | 23,244 [9,590, 42,033] | 25,413 [10,435, 45,680] |
| Lowest | 90 [38, 156] | 98 [42, 170] | 2,089 [886, 3,635] | 2,284 [969, 3,960] | 10 [4, 17] | 13 [5, 22] | 223 [93, 397] | 293 [124, 514] | 238 [117, 385] | 260 [129, 426] | 6 [3, 9] | 6 [3, 10] | 17 [7, 31] | 22 [9, 40] | 0.4 [0.2, 0.7] | 0.5 [0.2, 0.9] | 255 [131, 407] | 283 [147, 453] | 6 [3, 9] | 7 [3, 11] | 9 [5, 14] | 10 [5, 16] | 21,925 [9,046, 39,648] | 23,971 [9,843, 43,088] |
| Lower | 85 [36, 148] | 93 [40, 162] | 1,983 [841, 3,450] | 2,168 [920, 3,758] | 5 [2, 9] | 7 [3, 12] | 124 [52, 220] | 161 [68, 284] | 198 [97, 321] | 217 [108, 355] | 5 [2, 7] | 5 [2, 8] | 14 [6, 25] | 18 [8, 33] | 0.3 [0.1, 0.6] | 0.4 [0.2, 0.8] | 213 [109, 339] | 235 [122, 377] | 5 [3, 8] | 5 [3, 9] | 7 [4, 12] | 8 [4, 13] | 18,205 [7,511, 32,922] | 19,905 [8,173, 35,778] |
| Middle | 85 [36, 147] | 93 [39, 161] | 1,968 [835, 3,424] | 2,152 [913, 3,730] | 7 [3, 12] | 9 [4, 16] | 162 [68, 289] | 212 [90, 373] | 225 [110, 364] | 246 [122, 403] | 5 [3, 8] | 6 [3, 9] | 14 [6, 25] | 18 [8, 32] | 0.3 [0.1, 0.6] | 0.4 [0.2, 0.8] | 239 [121, 382] | 264 [137, 425] | 6 [3, 9] | 6 [3, 10] | 8 [4, 13] | 9 [5, 15] | 22,296 [9,199, 40,320] | 24,377 [10,010, 43,818] |
| Higher | 82 [35, 143] | 90 [38, 156] | 1,907 [809, 3,318] | 2,085 [885, 3,614] | 8 [3, 14] | 10 [4, 18] | 178 [75, 317] | 233 [99, 410] | 244 [120, 395] | 267 [132, 437] | 6 [3, 9] | 6 [3, 10] | 26 [11, 47] | 34 [14, 60] | 0.6 [0.3, 1.1] | 0.8 [0.3, 1.4] | 270 [140, 428] | 301 [158, 477] | 6 [3, 10] | 7 [4, 11] | 9 [5, 15] | 10 [6, 17] | 22,748 [9,385, 41,136] | 24,871 [10,212, 44,705] |
| Highest | 81 [34, 140] | 88 [37, 153] | 1,873 [795, 3,259] | 2,048 [869, 3,550] | 0 [0, 0] | 0 [0, 0] | 0 [0, 0] | 0 [0, 0] | 188 [92, 303] | 205 [102, 336] | 4 [2, 7] | 5 [2, 8] | 0 [0, 0] | 0 [0, 0] | 0.0 [0.0, 0.0] | 0.0 [0.0, 0.0] | 188 [92, 303] | 205 [102, 336] | 4 [2, 7] | 5 [2, 8] | 7 [3, 11] | 7 [4, 12] | 31,044 [12,808, 56,139] | 33,942 [13,937, 61,009] |
| Katanga | 583 [275, 1,095] | 638 [303, 1,193] | 2,205 [1,039, 4,137] | 2,411 [1,147, 4,509] | 111 [51, 211] | 146 [69, 276] | 419 [194, 796] | 551 [260, 1,042] | 2,890 [1,522, 5,088] | 3,160 [1,677, 5,535] | 11 [6, 19] | 12 [6, 21] | 709 [320, 1,380] | 926 [422, 1,783] | 2.7 [1.2, 5.2] | 3.5 [1.6, 6.7] | 3,599 [2,044, 6,084] | 4,086 [2,328, 6,828] | 14 [8, 23] | 15 [9, 26] | 125 [71, 212] | 142 [81, 237] | 11,696 [5,376, 22,875] | 12,788 [5,945, 24,941] |
| Lowest | 123 [58, 231] | 135 [64, 252] | 2,329 [1,097, 4,370] | 2,546 [1,211, 4,762] | 27 [13, 51] | 36 [17, 67] | 511 [237, 969] | 672 [317, 1,271] | 820 [432, 1,443] | 896 [476, 1,570] | 15 [8, 27] | 17 [9, 30] | 177 [80, 344] | 231 [105, 445] | 3.3 [1.5, 6.5] | 4.4 [2.0, 8.4] | 997 [563, 1,689] | 1,128 [641, 1,890] | 19 [11, 32] | 21 [12, 36] | 35 [20, 59] | 39 [22, 66] | 10,168 [4,674, 19,887] | 11,117 [5,169, 21,683] |
| Lower | 123 [58, 231] | 135 [64, 252] | 2,331 [1,098, 4,374] | 2,548 [1,212, 4,766] | 28 [13, 54] | 37 [18, 71] | 536 [248, 1,016] | 705 [333, 1,334] | 729 [384, 1,284] | 797 [423, 1,397] | 14 [7, 24] | 15 [8, 26] | 191 [86, 372] | 250 [114, 481] | 3.6 [1.6, 7.0] | 4.7 [2.2, 9.1] | 920 [523, 1,551] | 1,047 [600, 1,749] | 17 [10, 29] | 20 [11, 33] | 32 [18, 54] | 36 [21, 61] | 6,457 [2,968, 12,630] | 7,060 [3,282, 13,770] |
| Middle | 122 [57, 228] | 133 [63, 249] | 2,300 [1,083, 4,315] | 2,514 [1,196, 4,702] | 26 [12, 50] | 35 [16, 65] | 496 [230, 941] | 652 [308, 1,234] | 670 [353, 1,179] | 732 [389, 1,283] | 13 [7, 22] | 14 [7, 24] | 174 [78, 338] | 227 [104, 437] | 3.3 [1.5, 6.4] | 4.3 [2.0, 8.3] | 844 [480, 1,423] | 960 [549, 1,602] | 16 [9, 27] | 18 [10, 30] | 29 [17, 49] | 33 [19, 56] | 10,658 [4,899, 20,846] | 11,653 [5,418, 22,728] |
| Higher | 110 [52, 207] | 121 [57, 226] | 2,086 [982, 3,913] | 2,280 [1,085, 4,264] | 19 [9, 36] | 25 [12, 48] | 363 [168, 689] | 476 [225, 901] | 364 [192, 640] | 398 [211, 697] | 7 [4, 12] | 8 [4, 13] | 118 [53, 229] | 153 [70, 296] | 2.2 [1.0, 4.3] | 2.9 [1.3, 5.6] | 481 [275, 808] | 551 [317, 917] | 9 [5, 15] | 10 [6, 17] | 17 [10, 28] | 19 [11, 32] | 11,433 [5,255, 22,361] | 12,500 [5,812, 24,380] |
| Highest | 105 [49, 197] | 115 [54, 214] | 1,980 [933, 3,715] | 2,165 [1,030, 4,048] | 10 [5, 19] | 13 [6, 25] | 191 [89, 362] | 250 [118, 472] | 308 [162, 541] | 336 [178, 589] | 6 [3, 10] | 6 [3, 11] | 49 [22, 97] | 64 [29, 124] | 0.9 [0.4, 1.8] | 1.2 [0.6, 2.3] | 357 [199, 610] | 401 [225, 676] | 7 [4, 12] | 8 [4, 13] | 12 [7, 21] | 14 [8, 24] | 19,763 [9,084, 38,653] | 21,607 [10,046, 42,142] |
| Kasai-Oriental | 498 [234, 934] | 544 [256, 1,017] | 2,250 [1,057, 4,220] | 2,460 [1,159, 4,598] | 101 [47, 193] | 133 [62, 252] | 458 [212, 873] | 602 [280, 1,139] | 2,770 [1,470, 4,822] | 3,028 [1,591, 5,279] | 13 [7, 22] | 14 [7, 24] | 683 [304, 1,343] | 892 [404, 1,753] | 3.1 [1.4, 6.1] | 4.0 [1.8, 7.9] | 3,453 [1,932, 5,759] | 3,921 [2,213, 6,585] | 16 [9, 26] | 18 [10, 30] | 120 [67, 200] | 136 [77, 229] | 7,970 [3,583, 15,453] | 8,714 [3,972, 16,952] |
| Lowest | 103 [48, 193] | 113 [53, 211] | 2,328 [1,093, 4,366] | 2,546 [1,199, 4,758] | 25 [12, 48] | 33 [16, 63] | 573 [265, 1,093] | 755 [350, 1,429] | 786 [417, 1,368] | 859 [451, 1,498] | 18 [9, 31] | 19 [10, 34] | 192 [86, 377] | 251 [114, 491] | 4.3 [1.9, 8.5] | 5.7 [2.6, 11.1] | 978 [547, 1,631] | 1,110 [627, 1,864] | 22 [12, 37] | 25 [14, 42] | 34 [19, 57] | 39 [22, 65] | 6,483 [2,914, 12,569] | 7,088 [3,231, 13,788] |
| Lower | 102 [48, 191] | 112 [53, 209] | 2,307 [1,084, 4,327] | 2,523 [1,188, 4,715] | 22 [10, 41] | 28 [13, 53] | 486 [225, 927] | 639 [297, 1,208] | 645 [343, 1,124] | 706 [371, 1,230] | 15 [8, 25] | 16 [8, 28] | 154 [68, 302] | 201 [91, 394] | 3.5 [1.5, 6.8] | 4.5 [2.1, 8.9] | 799 [447, 1,333] | 906 [512, 1,524] | 18 [10, 30] | 20 [12, 34] | 28 [16, 46] | 31 [18, 53] | 2,778 [1,249, 5,385] | 3,037 [1,384, 5,908] |
| Middle | 102 [48, 191] | 111 [53, 208] | 2,304 [1,082, 4,321] | 2,519 [1,187, 4,709] | 20 [9, 37] | 26 [12, 48] | 441 [204, 840] | 580 [269, 1,095] | 529 [281, 921] | 578 [304, 1,008] | 12 [6, 21] | 13 [7, 23] | 118 [53, 233] | 154 [70, 304] | 2.7 [1.2, 5.3] | 3.5 [1.6, 6.9] | 647 [362, 1,083] | 733 [412, 1,233] | 15 [8, 24] | 17 [9, 28] | 23 [13, 38] | 25 [14, 43] | 6,905 [3,104, 13,387] | 7,549 [3,441, 14,685] |
| Higher | 98 [46, 183] | 107 [50, 200] | 2,208 [1,037, 4,141] | 2,414 [1,137, 4,513] | 19 [9, 37] | 26 [12, 48] | 440 [204, 839] | 578 [269, 1,093] | 445 [236, 774] | 486 [255, 847] | 10 [5, 17] | 11 [6, 19] | 125 [55, 245] | 163 [74, 320] | 2.8 [1.3, 5.5] | 3.7 [1.7, 7.2] | 569 [320, 949] | 649 [367, 1,090] | 13 [7, 21] | 15 [8, 25] | 20 [11, 33] | 23 [13, 38] | 7,631 [3,431, 14,796] | 8,344 [3,803, 16,231] |
| Highest | 93 [44, 174] | 102 [48, 190] | 2,103 [987, 3,943] | 2,299 [1,083, 4,297] | 15 [7, 29] | 20 [9, 38] | 350 [162, 667] | 459 [213, 867] | 365 [194, 635] | 399 [209, 695] | 8 [4, 14] | 9 [5, 16] | 95 [42, 186] | 124 [56, 243] | 2.1 [1.0, 4.2] | 2.8 [1.3, 5.5] | 459 [257, 766] | 522 [295, 878] | 10 [6, 17] | 12 [7, 20] | 16 [9, 27] | 18 [10, 30] | 16,054 [7,217, 31,126] | 17,553 [8,001, 34,146] |
| Kasai-Occidental | 386 [181, 722] | 423 [199, 793] | 2,294 [1,073, 4,287] | 2,508 [1,184, 4,706] | 87 [40, 167] | 115 [53, 219] | 518 [238, 991] | 682 [317, 1,300] | 2,089 [1,113, 3,692] | 2,284 [1,204, 4,041] | 12 [7, 22] | 14 [7, 24] | 667 [298, 1,298] | 872 [397, 1,685] | 4.0 [1.8, 7.7] | 5.2 [2.4, 10.0] | 2,755 [1,561, 4,617] | 3,155 [1,795, 5,296] | 16 [9, 27] | 19 [11, 31] | 96 [54, 160] | 109 [62, 184] | 6,169 [2,801, 12,137] | 6,745 [3,059, 13,219] |
| Lowest | 76 [35, 142] | 83 [39, 156] | 2,251 [1,053, 4,207] | 2,461 [1,162, 4,618] | 19 [9, 37] | 25 [12, 48] | 569 [261, 1,088] | 749 [349, 1,429] | 528 [281, 934] | 578 [304, 1,022] | 16 [8, 28] | 17 [9, 30] | 158 [70, 307] | 206 [94, 399] | 4.7 [2.1, 9.1] | 6.1 [2.8, 11.8] | 686 [389, 1,151] | 784 [446, 1,317] | 20 [12, 34] | 23 [13, 39] | 24 [13, 40] | 27 [15, 46] | 4,739 [2,152, 9,324] | 5,182 [2,350, 10,156] |
| Lower | 79 [37, 148] | 86 [41, 162] | 2,348 [1,098, 4,388] | 2,567 [1,212, 4,817] | 19 [9, 36] | 25 [12, 47] | 561 [257, 1,072] | 738 [344, 1,408] | 546 [291, 965] | 597 [315, 1,056] | 16 [9, 29] | 18 [9, 31] | 147 [66, 287] | 193 [88, 372] | 4.4 [2.0, 8.5] | 5.7 [2.6, 11.1] | 693 [392, 1,165] | 790 [447, 1,326] | 21 [12, 35] | 23 [13, 39] | 24 [14, 40] | 27 [16, 46] | 1,027 [466, 2,020] | 1,123 [509, 2,201] |
| Middle | 78 [36, 146] | 85 [40, 160] | 2,313 [1,082, 4,323] | 2,529 [1,194, 4,745] | 17 [8, 33] | 22 [10, 43] | 507 [233, 970] | 667 [311, 1,273] | 393 [210, 695] | 430 [227, 761] | 12 [6, 21] | 13 [7, 23] | 126 [56, 245] | 164 [75, 317] | 3.7 [1.7, 7.3] | 4.9 [2.2, 9.4] | 519 [294, 870] | 594 [338, 998] | 15 [9, 26] | 18 [10, 30] | 18 [10, 30] | 21 [12, 35] | 5,163 [2,344, 10,157] | 5,644 [2,560, 11,062] |
| Higher | 78 [37, 146] | 85 [40, 160] | 2,316 [1,084, 4,328] | 2,532 [1,195, 4,751] | 17 [8, 32] | 22 [10, 42] | 499 [229, 955] | 657 [306, 1,252] | 331 [177, 586] | 362 [191, 641] | 10 [5, 17] | 11 [6, 19] | 125 [56, 244] | 164 [75, 316] | 3.7 [1.7, 7.2] | 4.9 [2.2, 9.4] | 457 [259, 764] | 526 [300, 880] | 14 [8, 23] | 16 [9, 26] | 16 [9, 26] | 18 [10, 31] | 5,696 [2,587, 11,206] | 6,228 [2,824, 12,206] |
| Highest | 76 [35, 141] | 83 [39, 155] | 2,241 [1,049, 4,189] | 2,451 [1,157, 4,598] | 15 [7, 29] | 20 [9, 38] | 454 [209, 868] | 597 [278, 1,138] | 290 [154, 512] | 317 [167, 561] | 9 [5, 15] | 9 [5, 17] | 111 [49, 216] | 145 [66, 280] | 3.3 [1.5, 6.4] | 4.3 [2.0, 8.3] | 401 [227, 671] | 462 [263, 772] | 12 [7, 20] | 14 [8, 23] | 14 [8, 23] | 16 [9, 27] | 14,221 [6,458, 27,977] | 15,548 [7,051, 30,472] |
| Equateur | 476 [226, 882] | 520 [248, 957] | 2,282 [1,085, 4,231] | 2,495 [1,188, 4,591] | 83 [39, 156] | 109 [51, 204] | 397 [186, 748] | 521 [244, 981] | 2,615 [1,368, 4,604] | 2,859 [1,496, 5,027] | 13 [7, 22] | 14 [7, 24] | 592 [268, 1,144] | 773 [350, 1,508] | 2.8 [1.3, 5.5] | 3.7 [1.7, 7.2] | 3,206 [1,802, 5,426] | 3,631 [2,030, 6,151] | 15 [9, 26] | 17 [10, 30] | 111 [63, 189] | 126 [71, 214] | 7,738 [3,572, 15,127] | 8,461 [3,925, 16,321] |
| Lowest | 97 [46, 179] | 106 [50, 194] | 2,316 [1,101, 4,294] | 2,533 [1,205, 4,660] | 20 [9, 38] | 26 [12, 50] | 481 [225, 905] | 632 [296, 1,189] | 574 [301, 1,011] | 628 [329, 1,104] | 14 [7, 24] | 15 [8, 26] | 141 [64, 273] | 185 [84, 360] | 3.4 [1.5, 6.6] | 4.4 [2.0, 8.6] | 716 [404, 1,208] | 813 [455, 1,374] | 17 [10, 29] | 19 [11, 33] | 25 [14, 42] | 28 [16, 48] | 6,345 [2,929, 12,404] | 6,938 [3,218, 13,383] |
| Lower | 97 [46, 180] | 106 [50, 195] | 2,323 [1,104, 4,307] | 2,540 [1,209, 4,673] | 20 [10, 38] | 27 [13, 50] | 490 [229, 922] | 644 [301, 1,211] | 599 [313, 1,054] | 655 [343, 1,151] | 14 [8, 25] | 16 [8, 28] | 153 [69, 295] | 200 [91, 390] | 3.7 [1.7, 7.1] | 4.8 [2.2, 9.4] | 752 [425, 1,269] | 855 [480, 1,443] | 18 [10, 30] | 20 [12, 35] | 26 [15, 44] | 30 [17, 50] | 2,635 [1,217, 5,152] | 2,881 [1,337, 5,558] |
| Middle | 96 [45, 177] | 105 [50, 193] | 2,296 [1,091, 4,256] | 2,510 [1,195, 4,618] | 16 [7, 30] | 21 [10, 39] | 383 [179, 720] | 502 [235, 944] | 605 [317, 1,065] | 661 [346, 1,163] | 15 [8, 26] | 16 [8, 28] | 112 [51, 217] | 146 [66, 285] | 2.7 [1.2, 5.2] | 3.5 [1.6, 6.8] | 717 [400, 1,218] | 807 [449, 1,379] | 17 [10, 29] | 19 [11, 33] | 25 [14, 42] | 28 [16, 48] | 6,769 [3,125, 13,232] | 7,401 [3,433, 14,276] |
| Higher | 95 [45, 176] | 104 [49, 191] | 2,276 [1,082, 4,219] | 2,488 [1,184, 4,578] | 17 [8, 31] | 22 [10, 41] | 398 [186, 748] | 522 [244, 981] | 460 [241, 810] | 503 [263, 884] | 11 [6, 19] | 12 [6, 21] | 123 [56, 237] | 160 [73, 312] | 2.9 [1.3, 5.7] | 3.8 [1.7, 7.5] | 582 [330, 980] | 663 [372, 1,116] | 14 [8, 24] | 16 [9, 27] | 20 [11, 34] | 23 [13, 39] | 7,212 [3,329, 14,098] | 7,885 [3,658, 15,211] |
| Highest | 92 [44, 170] | 100 [48, 185] | 2,201 [1,046, 4,081] | 2,407 [1,145, 4,428] | 10 [5, 18] | 13 [6, 24] | 235 [110, 442] | 308 [144, 578] | 377 [197, 663] | 412 [216, 724] | 9 [5, 16] | 10 [5, 17] | 63 [28, 122] | 82 [37, 160] | 1.5 [0.7, 2.9] | 2.0 [0.9, 3.8] | 440 [245, 749] | 494 [273, 845] | 11 [6, 18] | 12 [7, 20] | 15 [8, 26] | 17 [10, 29] | 15,730 [7,261, 30,750] | 17,198 [7,978, 33,176] |
| Bas-Congo | 183 [85, 343] | 200 [94, 375] | 2,184 [1,013, 4,092] | 2,388 [1,122, 4,473] | 38 [17, 73] | 50 [23, 95] | 456 [208, 873] | 599 [276, 1,132] | 761 [405, 1,314] | 832 [440, 1,443] | 9 [5, 16] | 10 [5, 17] | 243 [108, 473] | 318 [144, 613] | 2.9 [1.3, 5.6] | 3.8 [1.7, 7.3] | 1,004 [575, 1,659] | 1,150 [660, 1,908] | 12 [7, 20] | 14 [8, 23] | 35 [20, 58] | 40 [23, 66] | 8,979 [4,076, 17,563] | 9,817 [4,494, 19,215] |
| Lowest | 39 [18, 72] | 42 [20, 79] | 2,303 [1,068, 4,315] | 2,518 [1,183, 4,716] | 10 [4, 19] | 13 [6, 24] | 582 [266, 1,115] | 767 [353, 1,448] | 203 [108, 351] | 222 [117, 385] | 12 [6, 21] | 13 [7, 23] | 68 [30, 132] | 89 [40, 172] | 4.1 [1.8, 7.9] | 5.3 [2.4, 10.3] | 271 [155, 447] | 311 [180, 515] | 16 [9, 27] | 19 [11, 31] | 9 [5, 16] | 11 [6, 18] | 7,556 [3,430, 14,781] | 8,262 [3,782, 16,171] |
| Lower | 38 [17, 70] | 41 [19, 77] | 2,241 [1,040, 4,200] | 2,451 [1,151, 4,590] | 9 [4, 17] | 11 [5, 21] | 515 [235, 987] | 678 [312, 1,281] | 153 [82, 265] | 168 [89, 291] | 9 [5, 16] | 10 [5, 17] | 56 [25, 108] | 73 [33, 140] | 3.3 [1.5, 6.5] | 4.3 [2.0, 8.4] | 209 [120, 345] | 240 [139, 397] | 12 [7, 21] | 14 [8, 24] | 7 [4, 12] | 8 [5, 14] | 3,837 [1,742, 7,505] | 4,195 [1,920, 8,211] |
| Middle | 37 [17, 70] | 41 [19, 77] | 2,235 [1,037, 4,188] | 2,444 [1,148, 4,578] | 9 [4, 17] | 12 [5, 22] | 534 [244, 1,023] | 703 [324, 1,327] | 184 [98, 318] | 202 [107, 350] | 11 [6, 19] | 12 [6, 21] | 59 [26, 115] | 78 [35, 150] | 3.5 [1.6, 6.9] | 4.6 [2.1, 8.9] | 244 [140, 402] | 279 [161, 463] | 15 [8, 24] | 17 [10, 28] | 8 [5, 14] | 10 [6, 16] | 7,937 [3,603, 15,525] | 8,677 [3,972, 16,985] |
| Higher | 36 [17, 67] | 39 [18, 73] | 2,140 [993, 4,010] | 2,340 [1,099, 4,383] | 6 [3, 11] | 8 [4, 15] | 357 [163, 683] | 468 [215, 884] | 118 [63, 204] | 129 [68, 224] | 7 [4, 12] | 8 [4, 13] | 36 [16, 70] | 47 [21, 91] | 2.1 [1.0, 4.2] | 2.8 [1.3, 5.4] | 154 [88, 255] | 176 [101, 292] | 9 [5, 15] | 10 [6, 17] | 5 [3, 9] | 6 [3, 10] | 8,452 [3,837, 16,534] | 9,241 [4,230, 18,089] |
| Highest | 34 [16, 63] | 37 [17, 69] | 2,000 [928, 3,748] | 2,187 [1,027, 4,097] | 5 [2, 9] | 6 [3, 12] | 290 [133, 556] | 381 [175, 719] | 102 [54, 176] | 112 [59, 194] | 6 [3, 11] | 7 [4, 12] | 24 [11, 47] | 31 [14, 61] | 1.4 [0.6, 2.8] | 1.9 [0.8, 3.6] | 126 [72, 210] | 143 [82, 239] | 8 [4, 13] | 9 [5, 14] | 4 [2, 7] | 5 [3, 8] | 17,111 [7,767, 33,471] | 18,708 [8,564, 36,618] |
| Bandundu | 500 [232, 921] | 547 [251, 1,021] | 2,233 [1,038, 4,111] | 2,442 [1,119, 4,557] | 91 [42, 172] | 120 [54, 227] | 408 [188, 770] | 536 [243, 1,015] | 2,271 [1,183, 3,968] | 2,483 [1,294, 4,339] | 10 [5, 18] | 11 [6, 19] | 464 [204, 912] | 606 [263, 1,201] | 2.1 [0.9, 4.1] | 2.7 [1.2, 5.4] | 2,735 [1,509, 4,665] | 3,089 [1,709, 5,223] | 12 [7, 21] | 14 [8, 23] | 95 [52, 162] | 107 [59, 182] | 7,154 [3,211, 13,973] | 7,822 [3,505, 15,270] |
| Lowest | 104 [48, 191] | 113 [52, 211] | 2,313 [1,075, 4,257] | 2,528 [1,158, 4,719] | 22 [10, 41] | 29 [13, 54] | 488 [224, 920] | 641 [291, 1,214] | 559 [291, 977] | 611 [318, 1,068] | 12 [6, 22] | 14 [7, 24] | 119 [52, 233] | 155 [67, 307] | 2.6 [1.2, 5.2] | 3.5 [1.5, 6.8] | 678 [374, 1,156] | 766 [425, 1,293] | 15 [8, 26] | 17 [9, 29] | 24 [13, 40] | 27 [15, 45] | 5,770 [2,590, 11,271] | 6,309 [2,827, 12,316] |
| Lower | 102 [48, 189] | 112 [51, 209] | 2,287 [1,063, 4,210] | 2,500 [1,145, 4,667] | 21 [10, 39] | 27 [12, 52] | 463 [213, 874] | 609 [276, 1,154] | 502 [262, 877] | 549 [286, 959] | 11 [6, 20] | 12 [6, 21] | 108 [47, 212] | 141 [61, 280] | 2.4 [1.1, 4.7] | 3.1 [1.4, 6.2] | 610 [337, 1,041] | 690 [383, 1,165] | 14 [8, 23] | 15 [9, 26] | 21 [12, 36] | 24 [13, 40] | 2,054 [922, 4,012] | 2,246 [1,006, 4,384] |
| Middle | 101 [47, 186] | 110 [51, 206] | 2,253 [1,047, 4,147] | 2,463 [1,128, 4,597] | 21 [10, 40] | 28 [12, 52] | 467 [215, 882] | 614 [279, 1,164] | 540 [282, 944] | 591 [308, 1,033] | 12 [6, 21] | 13 [7, 23] | 107 [47, 211] | 140 [61, 277] | 2.4 [1.0, 4.7] | 3.1 [1.4, 6.2] | 648 [357, 1,104] | 731 [405, 1,238] | 14 [8, 25] | 16 [9, 28] | 23 [12, 38] | 25 [14, 43] | 6,175 [2,772, 12,061] | 6,751 [3,026, 13,180] |
| Higher | 99 [46, 182] | 108 [49, 201] | 2,203 [1,024, 4,055] | 2,408 [1,103, 4,495] | 15 [7, 28] | 20 [9, 37] | 335 [154, 631] | 439 [199, 831] | 341 [177, 595] | 372 [194, 651] | 8 [4, 13] | 8 [4, 15] | 68 [30, 133] | 88 [38, 175] | 1.5 [0.7, 3.0] | 2.0 [0.9, 3.9] | 408 [225, 697] | 461 [255, 780] | 9 [5, 16] | 10 [6, 17] | 14 [8, 24] | 16 [9, 27] | 6,643 [2,982, 12,976] | 7,263 [3,255, 14,180] |
| Highest | 95 [44, 174] | 103 [47, 193] | 2,111 [981, 3,885] | 2,307 [1,057, 4,307] | 13 [6, 24] | 17 [8, 32] | 287 [132, 541] | 376 [171, 713] | 329 [171, 574] | 359 [187, 628] | 7 [4, 13] | 8 [4, 14] | 63 [27, 123] | 82 [35, 162] | 1.4 [0.6, 2.7] | 1.8 [0.8, 3.6] | 391 [216, 667] | 441 [244, 748] | 9 [5, 15] | 10 [5, 17] | 14 [8, 23] | 15 [8, 26] | 15,127 [6,791, 29,547] | 16,539 [7,413, 32,289] |
| KENYA |  |  |  |  |  |  |  |  |  |  |  |  |  |  |  |  |  |  |  |  |  |  |  |  |
| Western | 320 [153, 603] | 350 [165, 653] | 2,583 [1,232, 4,869] | 2,825 [1,331, 5,275] | 37 [18, 71] | 49 [23, 93] | 300 [142, 577] | 393 [184, 748] | 478 [344, 647] | 522 [378, 709] | 4 [3, 5] | 4 [3, 6] | 61 [28, 117] | 79 [37, 151] | 0.5 [0.2, 0.9] | 0.6 [0.3, 1.2] | 538 [392, 731] | 601 [437, 814] | 4 [3, 6] | 5 [4, 7] | 19 [14, 26] | 21 [16, 29] | 13,832 [5,902, 28,826] | 15,123 [6,497, 31,724] |
| Lowest | 66 [32, 125] | 73 [34, 136] | 2,679 [1,277, 5,049] | 2,929 [1,380, 5,470] | 11 [5, 20] | 14 [6, 26] | 427 [202, 821] | 560 [262, 1,066] | 124 [90, 169] | 136 [98, 185] | 5 [4, 7] | 5 [4, 7] | 19 [9, 37] | 25 [12, 48] | 0.8 [0.4, 1.5] | 1.0 [0.5, 1.9] | 144 [105, 196] | 161 [117, 219] | 6 [4, 8] | 7 [5, 9] | 5 [4, 7] | 6 [4, 8] | 8,770 [3,742, 18,278] | 9,589 [4,119, 20,116] |
| Lower | 66 [31, 124] | 72 [34, 134] | 2,651 [1,264, 4,997] | 2,899 [1,366, 5,414] | 8 [4, 16] | 11 [5, 21] | 334 [158, 642] | 437 [205, 832] | 100 [72, 136] | 110 [79, 149] | 4 [3, 5] | 4 [3, 6] | 13 [6, 25] | 17 [8, 32] | 0.5 [0.2, 1.0] | 0.7 [0.3, 1.3] | 113 [83, 154] | 127 [92, 172] | 5 [3, 6] | 5 [4, 7] | 4 [3, 5] | 5 [3, 6] | 9,244 [3,944, 19,264] | 10,106 [4,342, 21,201] |
| Middle | 64 [31, 121] | 70 [33, 132] | 2,602 [1,241, 4,903] | 2,844 [1,340, 5,312] | 7 [3, 13] | 9 [4, 17] | 272 [129, 523] | 356 [167, 677] | 83 [60, 113] | 91 [66, 124] | 3 [2, 5] | 4 [3, 5] | 11 [5, 21] | 14 [6, 26] | 0.4 [0.2, 0.8] | 0.6 [0.3, 1.1] | 94 [69, 128] | 105 [77, 142] | 4 [3, 5] | 4 [3, 6] | 3 [2, 5] | 4 [3, 5] | 7,702 [3,286, 16,051] | 8,421 [3,618, 17,665] |
| Higher | 65 [31, 122] | 71 [33, 132] | 2,610 [1,245, 4,920] | 2,854 [1,345, 5,330] | 7 [3, 14] | 10 [4, 18] | 294 [139, 566] | 386 [180, 734] | 93 [67, 126] | 102 [74, 138] | 4 [3, 5] | 4 [3, 6] | 11 [5, 21] | 14 [7, 27] | 0.4 [0.2, 0.9] | 0.6 [0.3, 1.1] | 104 [76, 141] | 116 [85, 157] | 4 [3, 6] | 5 [3, 6] | 4 [3, 5] | 4 [3, 6] | 14,118 [6,024, 29,423] | 15,436 [6,631, 32,380] |
| Highest | 59 [28, 111] | 64 [30, 120] | 2,375 [1,132, 4,476] | 2,597 [1,224, 4,849] | 4 [2, 8] | 6 [3, 11] | 173 [82, 332] | 226 [106, 429] | 76 [55, 103] | 83 [60, 113] | 3 [2, 4] | 3 [2, 5] | 7 [3, 13] | 9 [4, 17] | 0.3 [0.1, 0.5] | 0.4 [0.2, 0.7] | 83 [60, 112] | 92 [67, 124] | 3 [2, 5] | 4 [3, 5] | 3 [2, 4] | 3 [2, 4] | 29,324 [12,513, 61,113] | 32,061 [13,774, 67,257] |
| Rift Valley | 635 [299, 1,194] | 695 [324, 1,295] | 2,512 [1,183, 4,719] | 2,746 [1,281, 5,118] | 85 [39, 163] | 112 [52, 211] | 338 [156, 642] | 443 [204, 835] | 1,126 [812, 1,547] | 1,231 [895, 1,674] | 4 [3, 6] | 5 [4, 7] | 127 [58, 245] | 166 [76, 315] | 0.5 [0.2, 1.0] | 0.7 [0.3, 1.2] | 1,253 [912, 1,716] | 1,397 [1,017, 1,905] | 5 [4, 7] | 6 [4, 8] | 45 [32, 61] | 50 [36, 68] | 24,699 [10,543, 51,013] | 27,004 [11,508, 55,771] |
| Lowest | 140 [66, 262] | 153 [71, 284] | 2,758 [1,299, 5,181] | 3,015 [1,406, 5,619] | 22 [10, 42] | 29 [13, 55] | 439 [202, 836] | 576 [265, 1,087] | 381 [275, 523] | 417 [303, 566] | 8 [5, 10] | 8 [6, 11] | 36 [16, 69] | 47 [21, 88] | 0.7 [0.3, 1.4] | 0.9 [0.4, 1.7] | 417 [303, 570] | 463 [337, 631] | 8 [6, 11] | 9 [7, 12] | 15 [11, 20] | 16 [12, 22] | 18,598 [7,939, 38,412] | 20,334 [8,666, 41,995] |
| Lower | 135 [64, 255] | 148 [69, 276] | 2,678 [1,261, 5,030] | 2,928 [1,365, 5,456] | 21 [10, 39] | 27 [12, 51] | 408 [188, 777] | 536 [247, 1,011] | 246 [177, 337] | 269 [195, 365] | 5 [4, 7] | 5 [4, 7] | 31 [14, 60] | 41 [18, 77] | 0.6 [0.3, 1.2] | 0.8 [0.4, 1.5] | 277 [201, 380] | 309 [225, 421] | 5 [4, 8] | 6 [4, 8] | 10 [7, 13] | 11 [8, 15] | 18,971 [8,098, 39,182] | 20,741 [8,839, 42,837] |
| Middle | 132 [62, 247] | 144 [67, 268] | 2,600 [1,225, 4,885] | 2,843 [1,326, 5,298] | 18 [8, 35] | 24 [11, 45] | 362 [167, 688] | 474 [218, 895] | 200 [144, 275] | 219 [159, 298] | 4 [3, 5] | 4 [3, 6] | 26 [12, 51] | 34 [16, 65] | 0.5 [0.2, 1.0] | 0.7 [0.3, 1.3] | 227 [165, 311] | 253 [184, 345] | 4 [3, 6] | 5 [4, 7] | 8 [6, 11] | 9 [7, 12] | 17,922 [7,650, 37,016] | 19,595 [8,351, 40,469] |
| Higher | 122 [58, 230] | 134 [62, 250] | 2,421 [1,140, 4,548] | 2,647 [1,234, 4,932] | 14 [7, 27] | 19 [9, 36] | 285 [131, 542] | 373 [172, 704] | 166 [119, 227] | 181 [132, 246] | 3 [2, 4] | 4 [3, 5] | 21 [9, 40] | 27 [12, 52] | 0.4 [0.2, 0.8] | 0.5 [0.2, 1.0] | 186 [135, 256] | 208 [151, 284] | 4 [3, 5] | 4 [3, 6] | 7 [5, 9] | 7 [5, 10] | 25,906 [11,058, 53,505] | 28,324 [12,071, 58,496] |
| Highest | 106 [50, 200] | 116 [54, 217] | 2,103 [990, 3,950] | 2,299 [1,072, 4,284] | 10 [5, 19] | 13 [6, 24] | 194 [89, 368] | 253 [117, 478] | 134 [96, 184] | 146 [106, 199] | 3 [2, 4] | 3 [2, 4] | 13 [6, 26] | 17 [8, 33] | 0.3 [0.1, 0.5] | 0.3 [0.2, 0.6] | 147 [107, 201] | 163 [119, 222] | 3 [2, 4] | 3 [2, 4] | 5 [4, 7] | 6 [4, 8] | 42,098 [17,970, 86,948] | 46,027 [19,616, 95,059] |
| Nyanza | 426 [197, 792] | 466 [217, 869] | 2,561 [1,186, 4,756] | 2,800 [1,303, 5,218] | 46 [21, 88] | 61 [28, 115] | 279 [127, 527] | 365 [167, 693] | 616 [449, 845] | 673 [488, 916] | 4 [3, 5] | 4 [3, 6] | 92 [42, 175] | 120 [55, 228] | 0.6 [0.3, 1.0] | 0.7 [0.3, 1.4] | 708 [517, 966] | 793 [578, 1,080] | 4 [3, 6] | 5 [3, 6] | 25 [18, 34] | 28 [20, 38] | 15,544 [6,542, 31,682] | 16,995 [7,180, 34,729] |
| Lowest | 89 [41, 166] | 98 [45, 182] | 2,681 [1,242, 4,980] | 2,931 [1,364, 5,463] | 12 [6, 23] | 16 [7, 31] | 368 [168, 696] | 483 [221, 917] | 164 [119, 225] | 179 [130, 244] | 5 [4, 7] | 5 [4, 7] | 26 [12, 49] | 34 [15, 64] | 0.8 [0.4, 1.5] | 1.0 [0.5, 1.9] | 190 [138, 259] | 213 [155, 290] | 6 [4, 8] | 6 [5, 9] | 7 [5, 9] | 8 [5, 10] | 9,751 [4,104, 19,875] | 10,661 [4,504, 21,786] |
| Lower | 88 [41, 164] | 97 [45, 180] | 2,655 [1,230, 4,931] | 2,903 [1,351, 5,410] | 12 [6, 23] | 16 [7, 30] | 365 [166, 690] | 478 [219, 908] | 138 [101, 189] | 151 [109, 205] | 4 [3, 6] | 5 [3, 6] | 26 [12, 48] | 33 [15, 63] | 0.8 [0.4, 1.5] | 1.0 [0.5, 1.9] | 163 [119, 224] | 184 [134, 251] | 5 [4, 7] | 6 [4, 8] | 6 [4, 8] | 7 [5, 9] | 10,362 [4,361, 21,120] | 11,329 [4,787, 23,151] |
| Middle | 87 [40, 162] | 95 [44, 178] | 2,618 [1,213, 4,863] | 2,863 [1,332, 5,335] | 8 [4, 15] | 11 [5, 20] | 241 [110, 456] | 315 [144, 599] | 104 [76, 143] | 114 [83, 155] | 3 [2, 4] | 3 [2, 5] | 14 [6, 27] | 18 [8, 35] | 0.4 [0.2, 0.8] | 0.6 [0.3, 1.0] | 118 [87, 161] | 132 [96, 180] | 4 [3, 5] | 4 [3, 5] | 4 [3, 6] | 5 [3, 6] | 9,347 [3,934, 19,050] | 10,219 [4,317, 20,882] |
| Higher | 85 [40, 159] | 93 [43, 174] | 2,564 [1,188, 4,763] | 2,804 [1,305, 5,225] | 9 [4, 16] | 11 [5, 21] | 260 [118, 492] | 340 [156, 645] | 109 [79, 149] | 119 [86, 162] | 3 [2, 4] | 4 [3, 5] | 18 [8, 34] | 23 [11, 44] | 0.5 [0.2, 1.0] | 0.7 [0.3, 1.3] | 127 [92, 173] | 142 [104, 194] | 4 [3, 5] | 4 [3, 6] | 4 [3, 6] | 5 [4, 7] | 16,276 [6,850, 33,174] | 17,795 [7,518, 36,364] |
| Highest | 76 [35, 141] | 83 [39, 155] | 2,286 [1,059, 4,245] | 2,499 [1,163, 4,657] | 5 [2, 10] | 7 [3, 13] | 159 [72, 301] | 208 [95, 394] | 101 [74, 139] | 110 [80, 150] | 3 [2, 4] | 3 [2, 5] | 9 [4, 16] | 11 [5, 21] | 0.3 [0.1, 0.5] | 0.3 [0.2, 0.6] | 110 [81, 149] | 122 [89, 165] | 3 [2, 4] | 4 [3, 5] | 4 [3, 5] | 4 [3, 6] | 31,985 [13,462, 65,193] | 34,970 [14,775, 71,462] |
| North Eastern | 139 [66, 258] | 152 [73, 287] | 2,614 [1,236, 4,845] | 2,857 [1,362, 5,391] | 18 [8, 33] | 23 [11, 44] | 330 [154, 623] | 432 [202, 828] | 240 [174, 327] | 263 [191, 361] | 5 [3, 6] | 5 [4, 7] | 30 [14, 57] | 39 [18, 75] | 0.6 [0.3, 1.1] | 0.7 [0.3, 1.4] | 270 [196, 367] | 302 [221, 414] | 5 [4, 7] | 6 [4, 8] | 10 [7, 13] | 11 [8, 15] | 21,886 [9,357, 45,504] | 23,928 [10,333, 50,433] |
| Lowest | 30 [14, 55] | 32 [15, 61] | 2,772 [1,311, 5,139] | 3,030 [1,444, 5,717] | 5 [2, 9] | 6 [3, 12] | 441 [206, 833] | 579 [270, 1,110] | 61 [44, 83] | 67 [48, 91] | 6 [4, 8] | 6 [5, 9] | 8 [4, 14] | 10 [5, 19] | 0.7 [0.3, 1.3] | 0.9 [0.4, 1.8] | 68 [50, 93] | 76 [56, 105] | 6 [5, 9] | 7 [5, 10] | 2 [2, 3] | 3 [2, 4] | 15,291 [6,537, 31,793] | 16,718 [7,220, 35,236] |
| Lower | 30 [14, 55] | 32 [15, 61] | 2,773 [1,312, 5,142] | 3,032 [1,445, 5,721] | 4 [2, 8] | 5 [2, 10] | 377 [176, 712] | 494 [231, 947] | 62 [45, 84] | 67 [49, 93] | 6 [4, 8] | 6 [5, 9] | 7 [3, 14] | 9 [4, 18] | 0.7 [0.3, 1.3] | 0.9 [0.4, 1.7] | 69 [50, 93] | 77 [56, 105] | 6 [5, 9] | 7 [5, 10] | 2 [2, 3] | 3 [2, 4] | 15,953 [6,820, 33,169] | 17,442 [7,532, 36,761] |
| Middle | 29 [14, 53] | 31 [15, 59] | 2,696 [1,275, 4,998] | 2,947 [1,405, 5,561] | 4 [2, 7] | 5 [2, 10] | 367 [171, 692] | 480 [224, 921] | 49 [35, 66] | 53 [39, 73] | 5 [3, 6] | 5 [4, 7] | 7 [3, 13] | 9 [4, 17] | 0.7 [0.3, 1.2] | 0.8 [0.4, 1.6] | 56 [41, 76] | 62 [46, 86] | 5 [4, 7] | 6 [4, 8] | 2 [1, 3] | 2 [2, 3] | 14,613 [6,248, 30,383] | 15,977 [6,900, 33,674] |
| Higher | 28 [13, 51] | 30 [14, 57] | 2,599 [1,230, 4,818] | 2,842 [1,354, 5,361] | 3 [1, 6] | 4 [2, 8] | 294 [137, 554] | 384 [179, 737] | 41 [30, 56] | 45 [32, 61] | 4 [3, 5] | 4 [3, 6] | 6 [3, 11] | 7 [3, 14] | 0.5 [0.2, 1.0] | 0.7 [0.3, 1.3] | 47 [34, 63] | 52 [38, 71] | 4 [3, 6] | 5 [4, 7] | 2 [1, 2] | 2 [1, 3] | 22,142 [9,466, 46,037] | 24,208 [10,454, 51,023] |
| Highest | 24 [11, 44] | 26 [12, 49] | 2,228 [1,054, 4,130] | 2,436 [1,161, 4,595] | 2 [1, 3] | 2 [1, 5] | 170 [80, 322] | 223 [104, 427] | 28 [20, 38] | 31 [22, 42] | 3 [2, 4] | 3 [2, 4] | 3 [1, 5] | 4 [2, 7] | 0.3 [0.1, 0.5] | 0.3 [0.2, 0.6] | 31 [22, 42] | 34 [25, 47] | 3 [2, 4] | 3 [2, 4] | 1 [1, 1] | 1 [1, 2] | 41,430 [17,713, 86,140] | 45,296 [19,561, 95,469] |
| Nairobi | 146 [63, 248] | 159 [69, 269] | 2,125 [924, 3,616] | 2,323 [1,006, 3,922] | 11 [5, 19] | 14 [6, 24] | 159 [68, 275] | 209 [89, 355] | 153 [107, 201] | 167 [118, 219] | 2 [2, 3] | 2 [2, 3] | 16 [7, 27] | 20 [9, 35] | 0.2 [0.1, 0.4] | 0.3 [0.1, 0.5] | 169 [119, 220] | 188 [133, 244] | 2 [2, 3] | 3 [2, 4] | 6 [4, 8] | 7 [5, 9] | 34,033 [13,222, 62,712] | 37,210 [14,352, 68,237] |
| Lowest | 34 [15, 57] | 37 [16, 62] | 2,455 [1,068, 4,177] | 2,684 [1,163, 4,532] | 5 [2, 9] | 7 [3, 11] | 373 [160, 643] | 489 [209, 833] | 40 [28, 53] | 44 [31, 58] | 3 [2, 4] | 3 [2, 4] | 9 [4, 15] | 11 [5, 20] | 0.6 [0.3, 1.1] | 0.8 [0.4, 1.4] | 49 [34, 64] | 55 [39, 72] | 4 [3, 5] | 4 [3, 5] | 2 [1, 2] | 2 [1, 3] | 30,048 [11,674, 55,370] | 32,853 [12,672, 60,248] |
| Lower | 32 [14, 55] | 35 [15, 59] | 2,340 [1,018, 3,982] | 2,558 [1,108, 4,320] | 2 [1, 3] | 2 [1, 4] | 130 [56, 224] | 170 [73, 290] | 32 [22, 41] | 34 [24, 45] | 2 [2, 3] | 3 [2, 3] | 1 [0, 2] | 1 [1, 2] | 0.1 [0.0, 0.1] | 0.1 [0.0, 0.2] | 33 [23, 43] | 36 [25, 47] | 2 [2, 3] | 3 [2, 3] | 1 [1, 2] | 1 [1, 2] | 29,995 [11,653, 55,272] | 32,795 [12,650, 60,141] |
| Middle | 31 [13, 53] | 34 [15, 57] | 2,259 [983, 3,845] | 2,470 [1,070, 4,171] | 3 [1, 4] | 3 [1, 6] | 183 [78, 315] | 239 [102, 407] | 36 [25, 48] | 40 [28, 52] | 3 [2, 3] | 3 [2, 4] | 4 [2, 7] | 6 [2, 9] | 0.3 [0.1, 0.5] | 0.4 [0.2, 0.7] | 41 [29, 53] | 45 [32, 59] | 3 [2, 4] | 3 [2, 4] | 1 [1, 2] | 2 [1, 2] | 28,353 [11,015, 52,245] | 30,999 [11,957, 56,848] |
| Higher | 27 [12, 47] | 30 [13, 51] | 2,001 [871, 3,406] | 2,188 [948, 3,695] | 2 [1, 3] | 2 [1, 3] | 111 [48, 191] | 145 [62, 247] | 28 [19, 36] | 30 [21, 40] | 2 [1, 3] | 2 [2, 3] | 2 [1, 3] | 2 [1, 3] | 0.1 [0.0, 0.2] | 0.1 [0.1, 0.3] | 29 [21, 38] | 32 [23, 42] | 2 [2, 3] | 2 [2, 3] | 1 [1, 1] | 1 [1, 2] | 34,532 [13,415, 63,631] | 37,755 [14,563, 69,238] |
| Highest | 22 [9, 37] | 24 [10, 40] | 1,568 [682, 2,668] | 1,714 [743, 2,895] | 0 [0, 0] | 0 [0, 0] | 0 [0, 0] | 0 [0, 0] | 17 [12, 23] | 19 [13, 25] | 1 [1, 2] | 1 [1, 2] | 0 [0, 0] | 0 [0, 0] | 0.0 [0.0, 0.0] | 0.0 [0.0, 0.0] | 17 [12, 23] | 19 [13, 25] | 1 [1, 2] | 1 [1, 2] | 1 [0, 1] | 1 [0, 1] | 47,237 [18,351, 87,042] | 51,646 [19,921, 94,711] |
| Eastern | 341 [161, 627] | 373 [175, 693] | 2,544 [1,203, 4,672] | 2,781 [1,305, 5,169] | 44 [21, 81] | 58 [27, 109] | 328 [153, 607] | 430 [200, 813] | 623 [448, 849] | 681 [491, 927] | 5 [3, 6] | 5 [4, 7] | 64 [30, 122] | 84 [39, 160] | 0.5 [0.2, 0.9] | 0.6 [0.3, 1.2] | 687 [496, 933] | 765 [552, 1,043] | 5 [4, 7] | 6 [4, 8] | 24 [18, 33] | 27 [20, 37] | 25,635 [10,920, 52,014] | 28,028 [11,971, 57,414] |
| Lowest | 74 [35, 137] | 81 [38, 151] | 2,772 [1,311, 5,091] | 3,031 [1,422, 5,632] | 12 [6, 23] | 16 [8, 31] | 460 [215, 852] | 604 [281, 1,143] | 193 [139, 263] | 211 [152, 287] | 7 [5, 10] | 8 [6, 11] | 20 [9, 37] | 26 [12, 49] | 0.7 [0.3, 1.4] | 1.0 [0.4, 1.8] | 213 [154, 289] | 237 [171, 323] | 8 [6, 11] | 9 [6, 12] | 8 [5, 10] | 8 [6, 11] | 19,707 [8,395, 39,985] | 21,546 [9,202, 44,136] |
| Lower | 71 [34, 131] | 78 [37, 145] | 2,655 [1,256, 4,876] | 2,903 [1,362, 5,394] | 10 [5, 19] | 14 [6, 26] | 389 [182, 720] | 510 [238, 966] | 123 [89, 168] | 135 [97, 183] | 5 [3, 6] | 5 [4, 7] | 15 [7, 28] | 19 [9, 37] | 0.6 [0.3, 1.1] | 0.7 [0.3, 1.4] | 138 [100, 188] | 154 [111, 211] | 5 [4, 7] | 6 [4, 8] | 5 [4, 7] | 5 [4, 7] | 20,504 [8,734, 41,603] | 22,418 [9,574, 45,921] |
| Middle | 70 [33, 129] | 77 [36, 143] | 2,623 [1,241, 4,816] | 2,868 [1,346, 5,328] | 9 [4, 16] | 11 [5, 21] | 317 [148, 588] | 415 [194, 786] | 112 [81, 153] | 123 [89, 167] | 4 [3, 6] | 5 [3, 6] | 11 [5, 21] | 15 [7, 28] | 0.4 [0.2, 0.8] | 0.5 [0.3, 1.0] | 124 [89, 168] | 137 [99, 188] | 5 [3, 6] | 5 [4, 7] | 4 [3, 6] | 5 [4, 7] | 18,980 [8,085, 38,511] | 20,752 [8,863, 42,509] |
| Higher | 65 [31, 120] | 72 [34, 133] | 2,439 [1,154, 4,479] | 2,666 [1,251, 4,955] | 7 [3, 14] | 10 [5, 18] | 275 [129, 510] | 360 [168, 682] | 105 [76, 144] | 115 [83, 157] | 4 [3, 5] | 4 [3, 6] | 10 [5, 19] | 13 [6, 25] | 0.4 [0.2, 0.7] | 0.5 [0.2, 0.9] | 116 [83, 157] | 128 [93, 175] | 4 [3, 6] | 5 [3, 7] | 4 [3, 6] | 5 [3, 6] | 26,661 [11,357, 54,095] | 29,149 [12,449, 59,711] |
| Highest | 60 [28, 110] | 65 [31, 122] | 2,232 [1,056, 4,098] | 2,440 [1,145, 4,534] | 5 [2, 10] | 7 [3, 13] | 197 [92, 366] | 258 [120, 488] | 89 [64, 121] | 97 [70, 132] | 3 [2, 5] | 4 [3, 5] | 8 [4, 16] | 11 [5, 21] | 0.3 [0.1, 0.6] | 0.4 [0.2, 0.8] | 97 [70, 132] | 108 [78, 147] | 4 [3, 5] | 4 [3, 5] | 3 [2, 5] | 4 [3, 5] | 42,325 [18,030, 85,877] | 46,275 [19,764, 94,792] |
| Coast | 199 [94, 369] | 218 [103, 402] | 2,538 [1,202, 4,699] | 2,774 [1,313, 5,127] | 26 [12, 50] | 35 [16, 65] | 337 [157, 634] | 442 [206, 828] | 285 [205, 389] | 311 [223, 425] | 4 [3, 5] | 4 [3, 5] | 43 [20, 81] | 56 [26, 105] | 0.5 [0.3, 1.0] | 0.7 [0.3, 1.3] | 328 [236, 445] | 367 [265, 502] | 4 [3, 6] | 5 [3, 6] | 12 [8, 16] | 13 [9, 18] | 27,399 [11,619, 55,888] | 29,957 [13,057, 61,780] |
| Lowest | 42 [20, 78] | 46 [22, 85] | 2,689 [1,274, 4,979] | 2,940 [1,392, 5,432] | 7 [3, 14] | 10 [4, 18] | 462 [215, 868] | 606 [282, 1,135] | 80 [58, 110] | 88 [63, 120] | 5 [4, 7] | 6 [4, 8] | 11 [5, 22] | 15 [7, 28] | 0.7 [0.3, 1.4] | 1.0 [0.4, 1.8] | 92 [66, 125] | 103 [74, 141] | 6 [4, 8] | 7 [5, 9] | 3 [2, 4] | 4 [3, 5] | 20,066 [8,509, 40,929] | 21,938 [9,562, 45,244] |
| Lower | 42 [20, 77] | 45 [22, 84] | 2,649 [1,255, 4,906] | 2,896 [1,371, 5,352] | 6 [3, 12] | 8 [4, 16] | 402 [187, 756] | 528 [246, 988] | 67 [48, 92] | 74 [53, 100] | 4 [3, 6] | 5 [3, 6] | 11 [5, 20] | 14 [7, 26] | 0.7 [0.3, 1.3] | 0.9 [0.4, 1.7] | 78 [56, 106] | 88 [63, 120] | 5 [4, 7] | 6 [4, 8] | 3 [2, 4] | 3 [2, 4] | 19,904 [8,440, 40,600] | 21,762 [9,485, 44,880] |
| Middle | 40 [19, 74] | 44 [21, 81] | 2,563 [1,214, 4,746] | 2,802 [1,326, 5,178] | 6 [3, 11] | 7 [3, 14] | 356 [166, 670] | 467 [218, 875] | 54 [39, 74] | 59 [42, 80] | 3 [2, 5] | 4 [3, 5] | 8 [4, 16] | 11 [5, 20] | 0.5 [0.2, 1.0] | 0.7 [0.3, 1.3] | 62 [45, 85] | 70 [50, 96] | 4 [3, 5] | 4 [3, 6] | 2 [2, 3] | 2 [2, 3] | 20,096 [8,522, 40,990] | 21,971 [9,576, 45,311] |
| Higher | 39 [18, 72] | 42 [20, 78] | 2,470 [1,170, 4,575] | 2,701 [1,279, 4,991] | 3 [2, 6] | 4 [2, 8] | 219 [102, 411] | 286 [133, 535] | 41 [30, 57] | 45 [32, 62] | 3 [2, 4] | 3 [2, 4] | 5 [3, 10] | 7 [3, 13] | 0.3 [0.2, 0.7] | 0.5 [0.2, 0.9] | 47 [34, 64] | 52 [38, 72] | 3 [2, 4] | 3 [2, 5] | 2 [1, 2] | 2 [1, 3] | 31,220 [13,239, 63,682] | 34,134 [14,878, 70,395] |
| Highest | 36 [17, 67] | 40 [19, 73] | 2,318 [1,098, 4,292] | 2,534 [1,200, 4,683] | 4 [2, 7] | 5 [2, 10] | 248 [115, 465] | 324 [151, 607] | 42 [30, 57] | 45 [33, 62] | 3 [2, 4] | 3 [2, 4] | 7 [3, 13] | 9 [4, 17] | 0.4 [0.2, 0.8] | 0.6 [0.3, 1.1] | 48 [35, 66] | 54 [39, 74] | 3 [2, 4] | 3 [2, 5] | 2 [1, 2] | 2 [1, 3] | 45,711 [19,384, 93,240] | 49,977 [21,783, 103,068] |
| Central | 205 [97, 381] | 225 [106, 416] | 2,282 [1,073, 4,227] | 2,495 [1,174, 4,626] | 17 [8, 32] | 22 [10, 42] | 188 [87, 355] | 246 [115, 467] | 252 [182, 343] | 276 [199, 375] | 3 [2, 4] | 3 [2, 4] | 18 [8, 34] | 23 [11, 44] | 0.2 [0.1, 0.4] | 0.3 [0.1, 0.5] | 270 [196, 366] | 299 [217, 405] | 3 [2, 4] | 3 [2, 5] | 10 [7, 13] | 11 [8, 14] | 36,108 [15,420, 75,148] | 39,478 [16,763, 81,056] |
| Lowest | 46 [21, 85] | 50 [23, 93] | 2,537 [1,193, 4,699] | 2,773 [1,305, 5,142] | 5 [2, 9] | 6 [3, 12] | 275 [127, 521] | 360 [169, 684] | 63 [45, 85] | 68 [49, 93] | 3 [3, 5] | 4 [3, 5] | 5 [2, 9] | 6 [3, 12] | 0.3 [0.1, 0.5] | 0.3 [0.2, 0.7] | 67 [49, 91] | 75 [54, 101] | 4 [3, 5] | 4 [3, 6] | 2 [2, 3] | 3 [2, 4] | 28,872 [12,330, 60,088] | 31,567 [13,404, 64,812] |
| Lower | 43 [20, 80] | 47 [22, 88] | 2,402 [1,130, 4,449] | 2,626 [1,236, 4,870] | 4 [2, 8] | 6 [3, 11] | 241 [111, 455] | 315 [148, 599] | 55 [40, 75] | 60 [44, 82] | 3 [2, 4] | 3 [2, 5] | 4 [2, 8] | 6 [3, 11] | 0.2 [0.1, 0.5] | 0.3 [0.1, 0.6] | 60 [43, 81] | 66 [48, 90] | 3 [2, 4] | 4 [3, 5] | 2 [2, 3] | 2 [2, 3] | 29,641 [12,658, 61,688] | 32,407 [13,761, 66,538] |
| Middle | 41 [19, 76] | 45 [21, 84] | 2,291 [1,077, 4,244] | 2,505 [1,179, 4,645] | 3 [2, 7] | 5 [2, 9] | 193 [89, 365] | 252 [118, 479] | 51 [37, 69] | 56 [40, 76] | 3 [2, 4] | 3 [2, 4] | 4 [2, 7] | 5 [2, 9] | 0.2 [0.1, 0.4] | 0.3 [0.1, 0.5] | 55 [40, 74] | 61 [44, 82] | 3 [2, 4] | 3 [2, 5] | 2 [1, 3] | 2 [2, 3] | 29,997 [12,810, 62,430] | 32,797 [13,926, 67,338] |
| Higher | 40 [19, 74] | 44 [21, 81] | 2,213 [1,041, 4,100] | 2,420 [1,139, 4,487] | 2 [1, 5] | 3 [2, 6] | 138 [64, 261] | 180 [85, 342] | 48 [34, 65] | 52 [38, 71] | 3 [2, 4] | 3 [2, 4] | 3 [1, 5] | 3 [2, 6] | 0.1 [0.1, 0.3] | 0.2 [0.1, 0.4] | 50 [37, 68] | 55 [40, 75] | 3 [2, 4] | 3 [2, 4] | 2 [1, 2] | 2 [1, 3] | 39,169 [16,727, 81,519] | 42,825 [18,184, 87,927] |
| Highest | 35 [17, 66] | 39 [18, 72] | 1,967 [925, 3,643] | 2,150 [1,012, 3,987] | 2 [1, 3] | 2 [1, 4] | 93 [43, 176] | 121 [57, 230] | 36 [26, 49] | 39 [28, 53] | 2 [1, 3] | 2 [2, 3] | 2 [1, 4] | 3 [1, 6] | 0.1 [0.1, 0.2] | 0.2 [0.1, 0.3] | 38 [28, 52] | 42 [31, 57] | 2 [2, 3] | 2 [2, 3] | 1 [1, 2] | 2 [1, 2] | 52,861 [22,574, 110,015] | 57,795 [24,541, 118,663] |
| ZAMBIA |  |  |  |  |  |  |  |  |  |  |  |  |  |  |  |  |  |  |  |  |  |  |  |  |
| Western | 72 [34, 134] | 79 [37, 146] | 2,576 [1,207, 4,784] | 2,816 [1,312, 5,237] | 12 [6, 23] | 16 [7, 30] | 425 [200, 808] | 558 [256, 1,067] | #REF! | 122 [71, 200] | #REF! | 4 [3, 7] | 21 [10, 41] | 28 [13, 54] | 0.8 [0.4, 1.5] | 1.0 [0.5, 1.9] | 133 [81, 210] | 150 [91, 240] | 5 [3, 8] | 5 [3, 9] | 5 [3, 7] | 5 [3, 8] | 22,921 [9,769, 46,909] | 25,060 [10,712, 51,704] |
| Lowest | 15 [7, 28] | 16 [8, 30] | 2,685 [1,258, 4,988] | 2,936 [1,367, 5,460] | 2 [1, 5] | 3 [1, 6] | 445 [209, 846] | 585 [268, 1,118] | 27 [15, 43] | 29 [17, 48] | 5 [3, 8] | 5 [3, 9] | 4 [2, 8] | 6 [3, 11] | 0.8 [0.3, 1.5] | 1.0 [0.4, 1.9] | 31 [19, 49] | 35 [21, 55] | 6 [3, 9] | 6 [4, 10] | 1 [1, 2] | 1 [1, 2] | 15,214 [6,484, 31,136] | 16,634 [7,110, 34,319] |
| Lower | 15 [7, 27] | 16 [8, 30] | 2,647 [1,240, 4,916] | 2,894 [1,348, 5,382] | 3 [1, 5] | 4 [2, 7] | 515 [241, 977] | 676 [310, 1,292] | 25 [15, 42] | 28 [16, 46] | 5 [3, 7] | 5 [3, 8] | 6 [3, 11] | 7 [3, 14] | 1.0 [0.5, 2.0] | 1.3 [0.6, 2.6] | 31 [19, 49] | 35 [21, 56] | 6 [3, 9] | 6 [4, 10] | 1 [1, 2] | 1 [1, 2] | 15,438 [6,580, 31,596] | 16,879 [7,215, 34,826] |
| Middle | 15 [7, 27] | 16 [7, 30] | 2,601 [1,219, 4,832] | 2,844 [1,325, 5,290] | 2 [1, 5] | 3 [1, 6] | 432 [202, 819] | 567 [259, 1,083] | 21 [12, 35] | 23 [14, 38] | 4 [2, 6] | 4 [2, 7] | 4 [2, 8] | 5 [2, 10] | 0.7 [0.3, 1.4] | 1.0 [0.4, 1.9] | 25 [15, 40] | 29 [17, 46] | 5 [3, 7] | 5 [3, 8] | 1 [1, 1] | 1 [1, 2] | 12,950 [5,519, 26,504] | 14,159 [6,052, 29,213] |
| Higher | 14 [7, 27] | 16 [7, 29] | 2,582 [1,210, 4,796] | 2,823 [1,315, 5,250] | 2 [1, 4] | 3 [1, 5] | 359 [168, 681] | 471 [216, 900] | 22 [13, 36] | 24 [14, 39] | 4 [2, 6] | 4 [3, 7] | 3 [1, 6] | 4 [2, 8] | 0.6 [0.3, 1.1] | 0.7 [0.3, 1.4] | 25 [15, 40] | 28 [17, 45] | 4 [3, 7] | 5 [3, 8] | 1 [1, 1] | 1 [1, 2] | 17,000 [7,245, 34,793] | 18,587 [7,945, 38,349] |
| Highest | 13 [6, 25] | 14 [7, 27] | 2,363 [1,107, 4,389] | 2,583 [1,203, 4,804] | 2 [1, 4] | 3 [1, 5] | 376 [176, 713] | 493 [226, 942] | 17 [10, 27] | 18 [11, 30] | 3 [2, 5] | 3 [2, 5] | 4 [2, 8] | 5 [2, 10] | 0.7 [0.3, 1.4] | 0.9 [0.4, 1.8] | 21 [13, 33] | 23 [14, 37] | 4 [2, 6] | 4 [3, 7] | 1 [0, 1] | 1 [0, 1] | 54,001 [23,015, 110,518] | 59,041 [25,237, 121,815] |
| Southern | 125 [59, 233] | 137 [64, 257] | 2,423 [1,147, 4,528] | 2,650 [1,240, 4,994] | 20 [9, 38] | 26 [12, 51] | 391 [182, 740] | 513 [240, 983] | #REF! | 175 [101, 287] | #REF! | 3 [2, 6] | 31 [14, 61] | 41 [18, 79] | 0.6 [0.3, 1.2] | 0.8 [0.4, 1.5] | 191 [115, 309] | 216 [131, 343] | 4 [2, 6] | 4 [3, 7] | 7 [4, 11] | 8 [5, 12] | 28,731 [12,203, 58,800] | 31,413 [13,383, 65,425] |
| Lowest | 27 [13, 50] | 29 [14, 55] | 2,588 [1,225, 4,836] | 2,830 [1,324, 5,334] | 5 [2, 9] | 6 [3, 12] | 468 [217, 885] | 614 [287, 1,177] | 35 [20, 57] | 38 [22, 62] | 3 [2, 6] | 4 [2, 6] | 8 [4, 16] | 11 [5, 20] | 0.8 [0.4, 1.5] | 1.0 [0.5, 2.0] | 43 [26, 69] | 49 [29, 77] | 4 [3, 7] | 5 [3, 7] | 2 [1, 2] | 2 [1, 3] | 19,507 [8,285, 39,922] | 21,327 [9,087, 44,420] |
| Lower | 26 [12, 49] | 29 [13, 54] | 2,551 [1,207, 4,766] | 2,789 [1,305, 5,257] | 4 [2, 8] | 5 [3, 11] | 406 [189, 769] | 533 [249, 1,022] | 35 [20, 58] | 38 [22, 63] | 3 [2, 6] | 4 [2, 6] | 6 [3, 12] | 8 [4, 16] | 0.6 [0.3, 1.2] | 0.8 [0.4, 1.6] | 42 [25, 67] | 47 [28, 74] | 4 [2, 7] | 5 [3, 7] | 1 [1, 2] | 2 [1, 3] | 19,663 [8,352, 40,242] | 21,499 [9,159, 44,777] |
| Middle | 26 [12, 49] | 28 [13, 54] | 2,526 [1,196, 4,720] | 2,762 [1,293, 5,206] | 3 [2, 7] | 5 [2, 9] | 337 [157, 639] | 443 [207, 848] | 34 [20, 56] | 38 [22, 61] | 3 [2, 5] | 4 [2, 6] | 4 [2, 9] | 6 [3, 11] | 0.4 [0.2, 0.8] | 0.6 [0.3, 1.1] | 39 [23, 63] | 43 [26, 69] | 4 [2, 6] | 4 [3, 7] | 1 [1, 2] | 2 [1, 2] | 17,519 [7,441, 35,853] | 19,154 [8,160, 39,893] |
| Higher | 26 [12, 48] | 28 [13, 53] | 2,489 [1,178, 4,651] | 2,722 [1,274, 5,130] | 4 [2, 8] | 6 [3, 11] | 431 [200, 815] | 566 [264, 1,084] | 35 [20, 58] | 39 [22, 63] | 3 [2, 6] | 4 [2, 6] | 7 [3, 14] | 9 [4, 18] | 0.7 [0.3, 1.3] | 0.9 [0.4, 1.7] | 42 [26, 68] | 48 [29, 76] | 4 [2, 7] | 5 [3, 7] | 1 [1, 2] | 2 [1, 3] | 24,221 [10,288, 49,570] | 26,482 [11,283, 55,156] |
| Highest | 20 [10, 38] | 22 [10, 42] | 1,962 [928, 3,665] | 2,145 [1,004, 4,042] | 3 [1, 6] | 4 [2, 8] | 312 [145, 591] | 409 [191, 784] | 20 [12, 34] | 22 [13, 37] | 2 [1, 3] | 2 [1, 4] | 5 [2, 10] | 7 [3, 14] | 0.5 [0.2, 1.0] | 0.7 [0.3, 1.3] | 26 [16, 42] | 29 [18, 47] | 3 [2, 4] | 3 [2, 5] | 1 [1, 1] | 1 [1, 2] | 62,745 [26,650, 128,412] | 68,601 [29,228, 142,881] |
| North Western | 91 [43, 168] | 99 [47, 183] | 2,543 [1,193, 4,712] | 2,781 [1,313, 5,138] | 15 [7, 28] | 19 [9, 36] | 413 [192, 782] | 542 [255, 1,019] | #REF! | 144 [84, 231] | #REF! | 4 [2, 6] | 25 [11, 48] | 32 [15, 62] | 0.7 [0.3, 1.3] | 0.9 [0.4, 1.7] | 156 [95, 250] | 176 [107, 276] | 4 [3, 7] | 5 [3, 8] | 5 [3, 9] | 6 [4, 10] | 43,219 [18,578, 88,823] | 47,252 [20,419, 96,247] |
| Lowest | 19 [9, 35] | 20 [10, 38] | 2,625 [1,232, 4,863] | 2,870 [1,355, 5,302] | 3 [2, 6] | 4 [2, 8] | 454 [211, 860] | 596 [281, 1,121] | 29 [17, 48] | 32 [19, 51] | 4 [2, 7] | 4 [3, 7] | 5 [2, 10] | 7 [3, 13] | 0.8 [0.3, 1.5] | 1.0 [0.5, 1.9] | 34 [21, 55] | 39 [23, 61] | 5 [3, 8] | 5 [3, 9] | 1 [1, 2] | 1 [1, 2] | 34,674 [14,905, 71,262] | 37,910 [16,382, 77,218] |
| Lower | 18 [9, 34] | 20 [10, 37] | 2,592 [1,216, 4,802] | 2,834 [1,338, 5,236] | 3 [2, 6] | 4 [2, 8] | 468 [218, 887] | 615 [290, 1,156] | 30 [17, 48] | 32 [19, 52] | 4 [2, 7] | 5 [3, 7] | 6 [3, 11] | 8 [4, 15] | 0.8 [0.4, 1.6] | 1.1 [0.5, 2.0] | 35 [22, 57] | 40 [24, 63] | 5 [3, 8] | 6 [3, 9] | 1 [1, 2] | 1 [1, 2] | 35,092 [15,084, 72,122] | 38,368 [16,579, 78,150] |
| Middle | 18 [9, 34] | 20 [9, 37] | 2,568 [1,205, 4,758] | 2,808 [1,326, 5,188] | 3 [1, 5] | 4 [2, 7] | 390 [181, 738] | 512 [241, 962] | 27 [16, 45] | 30 [17, 48] | 4 [2, 6] | 4 [2, 7] | 5 [2, 9] | 6 [3, 11] | 0.6 [0.3, 1.2] | 0.8 [0.4, 1.6] | 32 [19, 51] | 36 [22, 56] | 4 [3, 7] | 5 [3, 8] | 1 [1, 2] | 1 [1, 2] | 32,316 [13,891, 66,417] | 35,333 [15,268, 71,967] |
| Higher | 18 [9, 34] | 20 [9, 37] | 2,575 [1,208, 4,771] | 2,815 [1,330, 5,201] | 3 [1, 6] | 4 [2, 8] | 428 [199, 811] | 562 [265, 1,057] | 26 [15, 43] | 29 [17, 47] | 4 [2, 6] | 4 [2, 7] | 5 [2, 10] | 7 [3, 13] | 0.7 [0.3, 1.4] | 1.0 [0.4, 1.8] | 32 [19, 51] | 36 [22, 56] | 4 [3, 7] | 5 [3, 8] | 1 [1, 2] | 1 [1, 2] | 37,505 [16,122, 77,081] | 41,006 [17,719, 83,523] |
| Highest | 17 [8, 31] | 18 [9, 34] | 2,357 [1,106, 4,367] | 2,577 [1,217, 4,761] | 2 [1, 4] | 3 [1, 6] | 324 [151, 613] | 425 [200, 798] | 19 [11, 31] | 21 [12, 33] | 3 [2, 4] | 3 [2, 5] | 4 [2, 7] | 5 [2, 9] | 0.5 [0.2, 1.0] | 0.7 [0.3, 1.3] | 23 [14, 36] | 25 [15, 40] | 3 [2, 5] | 4 [2, 6] | 1 [0, 1] | 1 [1, 1] | 76,506 [32,886, 157,235] | 83,646 [36,145, 170,376] |
| Northern | 57 [27, 107] | 63 [29, 118] | 2,583 [1,203, 4,822] | 2,824 [1,311, 5,302] | 12 [6, 23] | 16 [7, 30] | 545 [251, 1,034] | 717 [329, 1,360] | #REF! | 121 [71, 199] | #REF! | 5 [3, 9] | 30 [13, 59] | 39 [18, 77] | 1.4 [0.6, 2.7] | 1.8 [0.8, 3.5] | 141 [85, 224] | 161 [98, 257] | 6 [4, 10] | 7 [4, 12] | 5 [3, 8] | 6 [3, 9] | 23,751 [10,078, 48,693] | 25,968 [11,169, 53,258] |
| Lowest | 12 [5, 22] | 13 [6, 24] | 2,657 [1,238, 4,960] | 2,905 [1,349, 5,454] | 3 [1, 5] | 4 [2, 7] | 634 [291, 1,203] | 836 [383, 1,584] | 29 [17, 47] | 31 [18, 51] | 6 [4, 11] | 7 [4, 12] | 8 [3, 15] | 10 [4, 19] | 1.7 [0.8, 3.4] | 2.3 [1.0, 4.4] | 36 [22, 57] | 41 [25, 66] | 8 [5, 13] | 9 [6, 15] | 1 [1, 2] | 1 [1, 2] | 16,279 [6,907, 33,373] | 17,798 [7,655, 36,502] |
| Lower | 12 [5, 22] | 13 [6, 24] | 2,660 [1,239, 4,967] | 2,909 [1,350, 5,462] | 2 [1, 5] | 3 [2, 6] | 562 [258, 1,064] | 739 [339, 1,401] | 22 [13, 37] | 24 [14, 40] | 5 [3, 8] | 6 [3, 9] | 6 [3, 12] | 8 [4, 16] | 1.4 [0.6, 2.7] | 1.8 [0.8, 3.5] | 28 [17, 45] | 32 [20, 52] | 6 [4, 10] | 7 [4, 12] | 1 [1, 2] | 1 [1, 2] | 16,248 [6,895, 33,311] | 17,765 [7,640, 36,434] |
| Middle | 12 [5, 22] | 13 [6, 24] | 2,643 [1,231, 4,934] | 2,890 [1,342, 5,426] | 2 [1, 5] | 3 [1, 6] | 558 [256, 1,057] | 734 [336, 1,391] | 23 [14, 38] | 25 [15, 42] | 5 [3, 9] | 6 [3, 9] | 6 [3, 12] | 8 [4, 15] | 1.4 [0.6, 2.7] | 1.8 [0.8, 3.5] | 29 [18, 47] | 33 [20, 53] | 7 [4, 10] | 8 [5, 12] | 1 [1, 2] | 1 [1, 2] | 14,330 [6,081, 29,378] | 15,667 [6,738, 32,132] |
| Higher | 11 [5, 21] | 12 [6, 23] | 2,559 [1,192, 4,777] | 2,798 [1,299, 5,253] | 2 [1, 5] | 3 [1, 6] | 540 [248, 1,024] | 711 [326, 1,347] | 22 [13, 36] | 24 [14, 39] | 5 [3, 8] | 5 [3, 9] | 6 [3, 11] | 8 [3, 15] | 1.3 [0.6, 2.6] | 1.7 [0.8, 3.4] | 28 [17, 44] | 32 [19, 51] | 6 [4, 10] | 7 [4, 11] | 1 [1, 2] | 1 [1, 2] | 18,335 [7,780, 37,589] | 20,046 [8,622, 41,114] |
| Highest | 11 [5, 20] | 12 [5, 22] | 2,395 [1,116, 4,472] | 2,619 [1,216, 4,917] | 2 [1, 4] | 3 [1, 5] | 433 [199, 819] | 568 [261, 1,077] | 15 [9, 25] | 16 [10, 27] | 3 [2, 6] | 4 [2, 6] | 4 [2, 9] | 6 [3, 11] | 1.0 [0.4, 2.0] | 1.3 [0.6, 2.6] | 20 [12, 31] | 22 [14, 36] | 4 [3, 7] | 5 [3, 8] | 1 [0, 1] | 1 [0, 1] | 53,565 [22,729, 109,813] | 58,564 [25,188, 120,110] |
| Muchinga | 59 [27, 108] | 64 [30, 119] | 2,540 [1,181, 4,693] | 2,777 [1,296, 5,159] | 11 [5, 20] | 14 [6, 26] | 458 [212, 865] | 601 [277, 1,130] | #REF! | 116 [68, 191] | #REF! | 5 [3, 8] | 24 [11, 46] | 31 [14, 59] | 1.0 [0.5, 2.0] | 1.3 [0.6, 2.6] | 130 [78, 209] | 147 [90, 236] | 6 [3, 9] | 6 [4, 10] | 5 [3, 7] | 5 [3, 8] | 36,722 [15,562, 75,941] | 40,149 [16,966, 82,349] |
| Lowest | 12 [6, 22] | 13 [6, 24] | 2,579 [1,199, 4,763] | 2,819 [1,316, 5,237] | 3 [1, 5] | 3 [2, 6] | 556 [257, 1,051] | 731 [337, 1,375] | 28 [16, 46] | 30 [18, 50] | 6 [3, 10] | 7 [4, 11] | 7 [3, 13] | 9 [4, 16] | 1.4 [0.6, 2.7] | 1.8 [0.8, 3.5] | 34 [21, 55] | 39 [24, 62] | 7 [4, 12] | 8 [5, 13] | 1 [1, 2] | 1 [1, 2] | 28,408 [12,039, 58,748] | 31,059 [13,125, 63,705] |
| Lower | 12 [6, 22] | 13 [6, 24] | 2,569 [1,194, 4,745] | 2,808 [1,311, 5,217] | 2 [1, 4] | 3 [1, 5] | 441 [204, 834] | 579 [267, 1,089] | 23 [13, 37] | 25 [14, 41] | 5 [3, 8] | 5 [3, 9] | 4 [2, 8] | 5 [2, 10] | 0.9 [0.4, 1.7] | 1.1 [0.5, 2.2] | 27 [16, 43] | 30 [18, 48] | 6 [3, 9] | 6 [4, 10] | 1 [1, 2] | 1 [1, 2] | 28,780 [12,196, 59,517] | 31,466 [13,297, 64,539] |
| Middle | 12 [6, 22] | 13 [6, 24] | 2,586 [1,202, 4,777] | 2,828 [1,320, 5,253] | 2 [1, 4] | 3 [1, 6] | 482 [223, 912] | 634 [292, 1,191] | 21 [12, 35] | 23 [13, 38] | 5 [3, 8] | 5 [3, 8] | 5 [2, 10] | 6 [3, 13] | 1.1 [0.5, 2.1] | 1.4 [0.6, 2.7] | 26 [16, 42] | 30 [18, 47] | 6 [3, 9] | 6 [4, 10] | 1 [1, 1] | 1 [1, 2] | 27,700 [11,739, 57,284] | 30,285 [12,798, 62,118] |
| Higher | 12 [6, 22] | 13 [6, 24] | 2,577 [1,198, 4,761] | 2,818 [1,315, 5,234] | 2 [1, 4] | 3 [1, 5] | 446 [207, 843] | 586 [270, 1,101] | 20 [12, 33] | 22 [13, 36] | 4 [3, 7] | 5 [3, 8] | 4 [2, 9] | 6 [3, 11] | 1.0 [0.4, 1.9] | 1.3 [0.6, 2.4] | 25 [15, 40] | 28 [17, 45] | 5 [3, 9] | 6 [4, 10] | 1 [1, 1] | 1 [1, 2] | 32,044 [13,580, 66,268] | 35,035 [14,805, 71,860] |
| Highest | 11 [5, 20] | 12 [6, 22] | 2,391 [1,112, 4,417] | 2,614 [1,220, 4,856] | 2 [1, 3] | 2 [1, 4] | 363 [168, 687] | 476 [220, 895] | 15 [8, 24] | 16 [9, 26] | 3 [2, 5] | 3 [2, 6] | 4 [2, 7] | 5 [2, 9] | 0.8 [0.4, 1.5] | 1.0 [0.5, 2.0] | 18 [11, 29] | 21 [13, 33] | 4 [2, 6] | 4 [3, 7] | 1 [0, 1] | 1 [0, 1] | 66,676 [28,256, 137,887] | 72,899 [30,806, 149,523] |
| Lusaka | 158 [74, 296] | 172 [80, 325] | 2,185 [1,025, 4,097] | 2,388 [1,111, 4,496] | 25 [12, 47] | 32 [15, 62] | 342 [159, 657] | 448 [207, 854] | #REF! | 158 [92, 255] | #REF! | 2 [1, 4] | 34 [16, 66] | 45 [21, 86] | 0.5 [0.2, 0.9] | 0.6 [0.3, 1.2] | 179 [110, 283] | 202 [126, 316] | 2 [2, 4] | 3 [2, 4] | 6 [4, 10] | 7 [4, 11] | 21,904 [9,223, 45,009] | 23,948 [10,089, 49,850] |
| Lowest | 35 [16, 65] | 38 [18, 71] | 2,404 [1,128, 4,509] | 2,629 [1,223, 4,948] | 7 [3, 13] | 9 [4, 17] | 479 [223, 922] | 630 [291, 1,201] | 40 [24, 66] | 44 [26, 71] | 3 [2, 5] | 3 [2, 5] | 10 [5, 20] | 13 [6, 26] | 0.7 [0.3, 1.4] | 0.9 [0.4, 1.8] | 51 [31, 80] | 57 [36, 90] | 4 [2, 6] | 4 [2, 6] | 2 [1, 3] | 2 [1, 3] | 12,163 [5,121, 24,994] | 13,299 [5,602, 27,682] |
| Lower | 34 [16, 63] | 37 [17, 69] | 2,333 [1,094, 4,375] | 2,551 [1,187, 4,801] | 5 [2, 10] | 7 [3, 13] | 354 [165, 682] | 465 [214, 886] | 29 [17, 48] | 32 [19, 52] | 2 [1, 3] | 2 [1, 4] | 7 [3, 13] | 9 [4, 17] | 0.5 [0.2, 0.9] | 0.6 [0.3, 1.2] | 36 [22, 57] | 41 [26, 64] | 3 [2, 4] | 3 [2, 4] | 1 [1, 2] | 1 [1, 2] | 18,046 [7,598, 37,082] | 19,730 [8,312, 41,070] |
| Middle | 33 [16, 62] | 36 [17, 68] | 2,294 [1,076, 4,301] | 2,508 [1,167, 4,720] | 5 [2, 10] | 7 [3, 13] | 365 [170, 702] | 479 [221, 912] | 29 [17, 48] | 32 [19, 52] | 2 [1, 3] | 2 [1, 4] | 8 [4, 15] | 10 [5, 19] | 0.5 [0.2, 1.0] | 0.7 [0.3, 1.3] | 37 [23, 59] | 42 [26, 66] | 3 [2, 4] | 3 [2, 5] | 1 [1, 2] | 1 [1, 2] | 16,249 [6,842, 33,390] | 17,766 [7,484, 36,980] |
| Higher | 32 [15, 60] | 35 [16, 66] | 2,226 [1,044, 4,173] | 2,433 [1,132, 4,580] | 4 [2, 8] | 6 [3, 11] | 297 [139, 572] | 390 [180, 743] | 26 [15, 42] | 28 [17, 46] | 2 [1, 3] | 2 [1, 3] | 5 [2, 9] | 6 [3, 12] | 0.3 [0.2, 0.6] | 0.4 [0.2, 0.8] | 31 [19, 49] | 35 [21, 54] | 2 [1, 3] | 2 [1, 4] | 1 [1, 2] | 1 [1, 2] | 18,145 [7,640, 37,286] | 19,839 [8,358, 41,296] |
| Highest | 24 [11, 45] | 26 [12, 50] | 1,666 [782, 3,125] | 1,822 [848, 3,429] | 3 [1, 6] | 4 [2, 8] | 213 [99, 409] | 279 [129, 531] | 19 [11, 31] | 21 [12, 34] | 1 [1, 2] | 1 [1, 2] | 5 [2, 9] | 6 [3, 12] | 0.3 [0.2, 0.6] | 0.4 [0.2, 0.8] | 24 [15, 38] | 27 [17, 42] | 2 [1, 3] | 2 [1, 3] | 1 [1, 1] | 1 [1, 1] | 44,916 [18,912, 92,296] | 49,108 [20,688, 102,222] |
| Luapula | 75 [35, 142] | 82 [38, 152] | 2,467 [1,158, 4,668] | 2,698 [1,267, 5,023] | 15 [7, 29] | 20 [9, 38] | 498 [232, 951] | 654 [303, 1,243] | #REF! | 98 [57, 161] | #REF! | 3 [2, 5] | 35 [16, 68] | 46 [21, 87] | 1.2 [0.5, 2.2] | 1.5 [0.7, 2.9] | 125 [76, 197] | 144 [89, 224] | 4 [2, 6] | 5 [3, 7] | 4 [3, 7] | 5 [3, 8] | 13,023 [5,562, 26,736] | 14,239 [6,111, 29,351] |
| Lowest | 16 [7, 30] | 17 [8, 32] | 2,595 [1,218, 4,909] | 2,837 [1,333, 5,282] | 3 [1, 6] | 4 [2, 7] | 493 [230, 942] | 648 [300, 1,229] | 18 [11, 30] | 20 [12, 33] | 3 [2, 5] | 3 [2, 5] | 7 [3, 14] | 9 [4, 18] | 1.2 [0.5, 2.3] | 1.5 [0.7, 2.9] | 25 [15, 40] | 29 [18, 46] | 4 [3, 7] | 5 [3, 8] | 1 [1, 1] | 1 [1, 2] | 5,451 [2,328, 11,191] | 5,960 [2,558, 12,285] |
| Lower | 15 [7, 29] | 17 [8, 31] | 2,503 [1,175, 4,735] | 2,737 [1,286, 5,095] | 3 [2, 6] | 4 [2, 8] | 548 [256, 1,046] | 720 [334, 1,368] | 20 [12, 33] | 22 [13, 36] | 3 [2, 5] | 4 [2, 6] | 8 [4, 16] | 11 [5, 20] | 1.4 [0.6, 2.6] | 1.8 [0.8, 3.4] | 28 [17, 45] | 33 [20, 51] | 5 [3, 7] | 5 [3, 8] | 1 [1, 2] | 1 [1, 2] | 5,328 [2,275, 10,937] | 5,825 [2,500, 12,007] |
| Middle | 15 [7, 28] | 16 [8, 31] | 2,478 [1,163, 4,688] | 2,709 [1,273, 5,045] | 3 [1, 6] | 4 [2, 8] | 522 [244, 998] | 687 [318, 1,304] | 20 [12, 33] | 22 [13, 36] | 3 [2, 5] | 4 [2, 6] | 8 [3, 15] | 10 [5, 19] | 1.2 [0.6, 2.4] | 1.6 [0.8, 3.1] | 28 [17, 43] | 32 [20, 49] | 5 [3, 7] | 5 [3, 8] | 1 [1, 2] | 1 [1, 2] | 5,013 [2,141, 10,290] | 5,480 [2,352, 11,297] |
| Higher | 15 [7, 28] | 16 [8, 30] | 2,454 [1,152, 4,642] | 2,683 [1,260, 4,996] | 3 [1, 6] | 4 [2, 7] | 477 [223, 911] | 627 [291, 1,191] | 17 [10, 28] | 19 [11, 31] | 3 [2, 5] | 3 [2, 5] | 6 [3, 12] | 8 [4, 16] | 1.0 [0.5, 2.0] | 1.4 [0.6, 2.6] | 23 [14, 37] | 27 [17, 42] | 4 [2, 6] | 4 [3, 7] | 1 [1, 1] | 1 [1, 1] | 6,717 [2,868, 13,789] | 7,344 [3,152, 15,137] |
| Highest | 14 [7, 26] | 15 [7, 29] | 2,307 [1,083, 4,364] | 2,522 [1,185, 4,696] | 3 [1, 5] | 4 [2, 7] | 449 [209, 857] | 589 [273, 1,119] | 14 [8, 23] | 15 [9, 25] | 2 [1, 4] | 3 [1, 4] | 6 [3, 11] | 8 [4, 15] | 1.0 [0.5, 1.9] | 1.3 [0.6, 2.4] | 20 [12, 31] | 23 [14, 36] | 3 [2, 5] | 4 [2, 6] | 1 [0, 1] | 1 [0, 1] | 42,609 [18,196, 87,473] | 46,586 [19,993, 96,028] |
| Eastern | 121 [57, 228] | 133 [63, 251] | 2,427 [1,144, 4,553] | 2,654 [1,259, 5,011] | 22 [10, 43] | 29 [14, 57] | 445 [207, 852] | 585 [276, 1,135] | #REF! | 175 [102, 286] | #REF! | 3 [2, 6] | 52 [24, 100] | 67 [32, 130] | 1.0 [0.5, 2.0] | 1.3 [0.6, 2.6] | 212 [131, 329] | 242 [153, 380] | 4 [3, 7] | 5 [3, 8] | 7 [5, 12] | 8 [5, 13] | 20,079 [8,560, 41,662] | 21,953 [9,342, 46,137] |
| Lowest | 25 [12, 47] | 27 [13, 52] | 2,501 [1,178, 4,691] | 2,734 [1,298, 5,163] | 5 [3, 10] | 7 [3, 14] | 549 [255, 1,049] | 722 [340, 1,400] | 41 [24, 66] | 44 [26, 72] | 4 [2, 7] | 4 [3, 7] | 15 [7, 28] | 19 [9, 37] | 1.5 [0.7, 2.8] | 1.9 [0.9, 3.7] | 55 [34, 86] | 63 [40, 99] | 6 [3, 9] | 6 [4, 10] | 2 [1, 3] | 2 [1, 3] | 13,601 [5,799, 28,222] | 14,871 [6,328, 31,253] |
| Lower | 25 [12, 47] | 27 [13, 52] | 2,513 [1,184, 4,713] | 2,747 [1,304, 5,188] | 5 [2, 10] | 7 [3, 13] | 510 [237, 976] | 671 [316, 1,301] | 32 [19, 52] | 35 [21, 58] | 3 [2, 5] | 4 [2, 6] | 12 [6, 24] | 16 [8, 31] | 1.2 [0.6, 2.4] | 1.6 [0.8, 3.1] | 45 [28, 69] | 51 [32, 80] | 4 [3, 7] | 5 [3, 8] | 2 [1, 2] | 2 [1, 3] | 13,641 [5,816, 28,305] | 14,915 [6,347, 31,345] |
| Middle | 25 [12, 46] | 27 [13, 51] | 2,464 [1,161, 4,622] | 2,694 [1,278, 5,087] | 5 [2, 9] | 6 [3, 12] | 471 [219, 902] | 620 [292, 1,201] | 34 [20, 55] | 37 [22, 61] | 3 [2, 5] | 4 [2, 6] | 11 [5, 21] | 14 [7, 28] | 1.1 [0.5, 2.1] | 1.4 [0.7, 2.8] | 45 [28, 70] | 51 [32, 81] | 4 [3, 7] | 5 [3, 8] | 2 [1, 2] | 2 [1, 3] | 10,698 [4,561, 22,197] | 11,696 [4,977, 24,582] |
| Higher | 24 [11, 45] | 26 [12, 50] | 2,407 [1,134, 4,514] | 2,631 [1,249, 4,968] | 4 [2, 7] | 5 [2, 9] | 367 [170, 702] | 482 [227, 933] | 30 [17, 48] | 33 [19, 53] | 3 [2, 5] | 3 [2, 5] | 8 [3, 15] | 10 [5, 19] | 0.8 [0.3, 1.5] | 1.0 [0.5, 1.9] | 37 [23, 58] | 42 [27, 67] | 4 [2, 6] | 4 [3, 7] | 1 [1, 2] | 1 [1, 2] | 13,053 [5,565, 27,084] | 14,271 [6,073, 29,993] |
| Highest | 23 [11, 42] | 25 [12, 46] | 2,251 [1,061, 4,223] | 2,462 [1,168, 4,648] | 3 [2, 6] | 4 [2, 8] | 330 [153, 630] | 433 [204, 838] | 24 [14, 38] | 26 [15, 42] | 2 [1, 4] | 3 [2, 4] | 6 [3, 12] | 8 [4, 16] | 0.6 [0.3, 1.2] | 0.8 [0.4, 1.6] | 30 [18, 46] | 34 [21, 53] | 3 [2, 5] | 3 [2, 5] | 1 [1, 2] | 1 [1, 2] | 49,400 [21,061, 102,503] | 54,011 [22,984, 113,513] |
| Copperbelt | 132 [57, 224] | 144 [62, 246] | 1,986 [1,146, 4,477] | 2,171 [1,246, 4,921] | 22 [10, 42] | 29 [14, 56] | 327 [207, 842] | 430 [273, 1,110] | #REF! | 187 [102, 284] | #REF! | 3 [2, 6] | 30 [24, 98] | 39 [31, 129] | 0.5 [0.5, 2.0] | 0.6 [0.6, 2.6] | 201 [130, 330] | 226 [151, 381] | 3 [3, 7] | 3 [3, 8] | 7 [5, 12] | 8 [5, 13] | 34,966 [8,573, 40,731] | 38,229 [9,295, 45,675] |
| Lowest | 34 [12, 46] | 37 [13, 51] | 2,581 [1,181, 4,613] | 2,822 [1,284, 5,070] | 6 [3, 10] | 8 [3, 14] | 481 [255, 1,038] | 632 [337, 1,369] | 55 [24, 66] | 60 [26, 72] | 4 [2, 7] | 4 [3, 7] | 9 [7, 28] | 12 [9, 36] | 0.7 [0.7, 2.8] | 0.9 [0.9, 3.6] | 64 [34, 86] | 72 [39, 99] | 5 [3, 9] | 5 [4, 10] | 2 [1, 3] | 3 [1, 3] | 16,138 [5,807, 27,591] | 17,644 [6,296, 30,941] |
| Lower | 32 [12, 46] | 35 [13, 51] | 2,402 [1,186, 4,635] | 2,626 [1,290, 5,094] | 5 [2, 10] | 7 [3, 13] | 400 [237, 965] | 525 [313, 1,272] | 40 [19, 52] | 44 [21, 57] | 3 [2, 5] | 3 [2, 6] | 8 [6, 23] | 10 [7, 31] | 0.6 [0.6, 2.3] | 0.8 [0.7, 3.1] | 47 [27, 70] | 54 [32, 81] | 4 [3, 7] | 4 [3, 8] | 2 [1, 2] | 2 [1, 3] | 30,777 [5,825, 27,672] | 33,650 [6,315, 31,032] |
| Middle | 29 [12, 45] | 32 [13, 50] | 2,178 [1,163, 4,545] | 2,381 [1,265, 4,995] | 4 [2, 9] | 6 [3, 12] | 332 [219, 892] | 436 [289, 1,175] | 31 [20, 55] | 34 [22, 60] | 2 [2, 5] | 3 [2, 6] | 6 [5, 21] | 7 [7, 27] | 0.4 [0.5, 2.1] | 0.5 [0.7, 2.7] | 37 [28, 70] | 41 [32, 81] | 3 [3, 7] | 3 [3, 8] | 1 [1, 2] | 1 [1, 3] | 30,491 [4,568, 21,701] | 33,337 [4,952, 24,336] |
| Higher | 22 [11, 44] | 24 [12, 49] | 1,643 [1,136, 4,439] | 1,797 [1,235, 4,879] | 4 [2, 7] | 5 [2, 9] | 284 [171, 694] | 373 [225, 914] | 29 [17, 48] | 32 [19, 53] | 2 [2, 5] | 2 [2, 5] | 5 [3, 14] | 7 [5, 19] | 0.4 [0.3, 1.4] | 0.5 [0.5, 1.9] | 34 [23, 58] | 39 [26, 66] | 3 [2, 6] | 3 [3, 7] | 1 [1, 2] | 1 [1, 2] | 34,725 [5,573, 26,478] | 37,966 [6,042, 29,693] |
| Highest | 15 [11, 42] | 16 [12, 46] | 1,125 [1,063, 4,153] | 1,230 [1,156, 4,564] | 2 [2, 6] | 2 [2, 8] | 139 [153, 623] | 182 [202, 820] | 16 [14, 38] | 18 [15, 42] | 1 [1, 4] | 1 [2, 4] | 2 [3, 12] | 3 [4, 16] | 0.2 [0.3, 1.2] | 0.2 [0.4, 1.6] | 19 [18, 47] | 21 [21, 53] | 1 [2, 5] | 2 [2, 5] | 1 [1, 2] | 1 [1, 2] | 62,697 [21,093, 100,211] | 68,549 [22,868, 112,377] |
| Central | 105 [49, 196] | 114 [53, 215] | 2,382 [1,124, 4,467] | 2,604 [1,212, 4,886] | 20 [9, 38] | 26 [12, 50] | 458 [214, 871] | 602 [273, 1,143] | #REF! | 167 [97, 273] | #REF! | 4 [2, 6] | 40 [18, 77] | 52 [24, 102] | 0.9 [0.4, 1.8] | 1.2 [0.5, 2.3] | 192 [118, 305] | 219 [135, 347] | 4 [3, 7] | 5 [3, 8] | 7 [4, 11] | 8 [5, 12] | 38,987 [16,775, 81,167] | 42,626 [18,386, 88,220] |
| Lowest | 22 [10, 41] | 24 [11, 45] | 2,491 [1,175, 4,672] | 2,724 [1,268, 5,111] | 5 [2, 9] | 6 [3, 12] | 537 [251, 1,020] | 706 [321, 1,342] | 39 [23, 64] | 43 [25, 71] | 4 [3, 7] | 5 [3, 8] | 10 [5, 19] | 13 [6, 26] | 1.2 [0.5, 2.2] | 1.5 [0.7, 2.9] | 50 [30, 79] | 56 [35, 89] | 6 [3, 9] | 6 [4, 10] | 2 [1, 3] | 2 [1, 3] | 30,555 [13,147, 63,613] | 33,407 [14,410, 69,141] |
| Lower | 22 [10, 40] | 24 [11, 44] | 2,455 [1,158, 4,604] | 2,684 [1,249, 5,036] | 4 [2, 8] | 6 [3, 10] | 477 [223, 907] | 627 [285, 1,191] | 29 [17, 47] | 32 [18, 52] | 3 [2, 5] | 4 [2, 6] | 8 [4, 16] | 11 [5, 21] | 1.0 [0.4, 1.8] | 1.2 [0.6, 2.4] | 37 [23, 59] | 42 [26, 67] | 4 [3, 7] | 5 [3, 8] | 1 [1, 2] | 1 [1, 2] | 31,132 [13,396, 64,814] | 34,038 [14,682, 70,447] |
| Middle | 22 [10, 41] | 24 [11, 45] | 2,475 [1,168, 4,641] | 2,706 [1,259, 5,077] | 4 [2, 8] | 6 [3, 11] | 481 [225, 914] | 632 [287, 1,201] | 31 [18, 51] | 34 [20, 56] | 4 [2, 6] | 4 [2, 6] | 8 [4, 16] | 11 [5, 21] | 1.0 [0.4, 1.8] | 1.3 [0.6, 2.4] | 40 [24, 63] | 45 [28, 72] | 5 [3, 7] | 5 [3, 8] | 1 [1, 2] | 2 [1, 3] | 29,287 [12,602, 60,972] | 32,020 [13,811, 66,271] |
| Higher | 22 [10, 41] | 24 [11, 44] | 2,457 [1,159, 4,608] | 2,686 [1,250, 5,040] | 4 [2, 7] | 5 [2, 10] | 444 [208, 844] | 583 [265, 1,107] | 33 [19, 54] | 36 [21, 60] | 4 [2, 6] | 4 [2, 7] | 8 [3, 15] | 10 [4, 19] | 0.9 [0.4, 1.6] | 1.1 [0.5, 2.2] | 41 [25, 65] | 46 [28, 73] | 5 [3, 7] | 5 [3, 8] | 1 [1, 2] | 2 [1, 3] | 32,735 [14,085, 68,151] | 35,791 [15,438, 74,074] |
| Highest | 18 [8, 33] | 20 [9, 37] | 2,030 [958, 3,808] | 2,220 [1,033, 4,165] | 3 [1, 6] | 4 [2, 8] | 351 [164, 668] | 461 [210, 876] | 19 [11, 32] | 21 [12, 35] | 2 [1, 4] | 2 [1, 4] | 6 [3, 11] | 7 [3, 14] | 0.6 [0.3, 1.2] | 0.8 [0.4, 1.6] | 25 [15, 40] | 29 [18, 45] | 3 [2, 5] | 3 [2, 5] | 1 [1, 1] | 1 [1, 2] | 71,224 [30,646, 148,282] | 77,872 [33,589, 161,168] |
| ZIMBABWE |  |  |  |  |  |  |  |  |  |  |  |  |  |  |  |  |  |  |  |  |  |  |  |  |
| Bulawayo | 28 [13, 53] | 31 [15, 58] | 1,728 [824, 3,243] | 1,890 [901, 3,570] | 2 [1, 4] | 2 [1, 5] | 140 [66, 266] | 153 [72, 290] | 107 [70, 158] | 117 [76, 173] | 7 [4, 10] | 7 [5, 11] | 4 [2, 8] | 5 [2, 9] | 0.3 [0.1, 0.5] | 0.3 [0.1, 0.5] | 111 [73, 164] | 121 [80, 179] | 7 [4, 10] | 7 [5, 11] | 4 [3, 6] | 4 [3, 6] | 22,611 [11,977, 41,413] | 24,721 [13,023, 44,867] |
| Lowest | 7 [3, 14] | 8 [4, 15] | 2,219 [1,058, 4,164] | 2,426 [1,157, 4,584] | 1 [0, 1] | 1 [0, 2] | 237 [113, 451] | 259 [123, 493] | 30 [20, 44] | 33 [21, 49] | 9 [6, 14] | 10 [7, 15] | 2 [1, 4] | 2 [1, 4] | 0.6 [0.3, 1.2] | 0.7 [0.3, 1.3] | 32 [21, 47] | 35 [23, 52] | 10 [6, 14] | 11 [7, 16] | 1 [1, 2] | 1 [1, 2] | 17,030 [9,021, 31,191] | 18,619 [9,809, 33,792] |
| Lower | 7 [3, 13] | 8 [4, 14] | 2,117 [1,010, 3,972] | 2,315 [1,104, 4,373] | 1 [0, 1] | 1 [0, 1] | 174 [83, 332] | 191 [90, 363] | 25 [16, 36] | 27 [18, 40] | 7 [5, 11] | 8 [5, 12] | 1 [0, 2] | 1 [0, 2] | 0.3 [0.1, 0.5] | 0.3 [0.1, 0.6] | 25 [17, 38] | 28 [18, 41] | 8 [5, 11] | 8 [6, 13] | 1 [1, 1] | 1 [1, 1] | 20,751 [10,992, 38,006] | 22,687 [11,952, 41,176] |
| Middle | 6 [3, 11] | 6 [3, 12] | 1,725 [822, 3,236] | 1,886 [899, 3,563] | 0 [0, 1] | 0 [0, 1] | 138 [66, 263] | 151 [72, 287] | 23 [15, 34] | 25 [16, 37] | 7 [5, 10] | 8 [5, 11] | 1 [0, 1] | 1 [0, 1] | 0.2 [0.1, 0.4] | 0.2 [0.1, 0.4] | 24 [15, 35] | 26 [17, 38] | 7 [5, 11] | 8 [5, 12] | 1 [1, 1] | 1 [1, 1] | 16,611 [8,799, 30,424] | 18,161 [9,567, 32,961] |
| Higher | 4 [2, 8] | 5 [2, 9] | 1,267 [604, 2,378] | 1,386 [661, 2,618] | 0 [0, 1] | 0 [0, 1] | 85 [40, 161] | 93 [44, 176] | 16 [11, 24] | 18 [12, 27] | 5 [3, 7] | 5 [4, 8] | 0 [0, 1] | 1 [0, 1] | 0.1 [0.1, 0.3] | 0.2 [0.1, 0.3] | 17 [11, 25] | 18 [12, 27] | 5 [3, 8] | 6 [4, 8] | 1 [0, 1] | 1 [0, 1] | 22,916 [12,139, 41,972] | 25,055 [13,199, 45,473] |
| Highest | 4 [2, 8] | 5 [2, 9] | 1,313 [626, 2,464] | 1,436 [685, 2,713] | 0 [0, 0] | 0 [0, 0] | 64 [30, 121] | 70 [33, 132] | 13 [8, 19] | 14 [9, 21] | 4 [3, 6] | 4 [3, 6] | 0 [0, 0] | 0 [0, 0] | 0.1 [0.0, 0.1] | 0.1 [0.0, 0.1] | 13 [8, 19] | 14 [9, 21] | 4 [3, 6] | 4 [3, 6] | 0 [0, 1] | 1 [0, 1] | 35,748 [18,936, 65,474] | 39,085 [20,590, 70,935] |
| Harare | 114 [53, 210] | 124 [59, 231] | 2,398 [1,125, 4,430] | 2,622 [1,237, 4,880] | 12 [5, 22] | 13 [6, 24] | 248 [113, 469] | 271 [125, 509] | 831 [544, 1,228] | 909 [591, 1,343] | 18 [11, 26] | 19 [12, 28] | 48 [21, 93] | 53 [23, 103] | 1.0 [0.4, 2.0] | 1.1 [0.5, 2.2] | 880 [576, 1,293] | 961 [629, 1,419] | 19 [12, 27] | 20 [13, 30] | 31 [20, 46] | 34 [22, 50] | 17,176 [9,015, 31,341] | 18,779 [9,807, 35,057] |
| Lowest | 25 [12, 46] | 27 [13, 51] | 2,649 [1,243, 4,894] | 2,896 [1,366, 5,391] | 4 [2, 8] | 5 [2, 9] | 474 [217, 897] | 518 [239, 974] | 216 [141, 320] | 237 [154, 350] | 23 [15, 34] | 25 [16, 37] | 21 [9, 41] | 23 [10, 45] | 2.3 [1.0, 4.3] | 2.5 [1.1, 4.8] | 238 [156, 350] | 260 [170, 382] | 25 [16, 37] | 27 [18, 40] | 8 [6, 12] | 9 [6, 13] | 11,860 [6,224, 21,640] | 12,967 [6,771, 24,206] |
| Lower | 26 [12, 48] | 28 [13, 53] | 2,728 [1,280, 5,040] | 2,983 [1,407, 5,551] | 4 [2, 7] | 4 [2, 7] | 384 [176, 727] | 420 [194, 790] | 186 [121, 274] | 203 [132, 300] | 20 [13, 29] | 21 [14, 32] | 14 [6, 27] | 15 [7, 30] | 1.5 [0.6, 2.8] | 1.6 [0.7, 3.1] | 200 [131, 293] | 218 [143, 322] | 21 [14, 31] | 23 [15, 34] | 7 [5, 10] | 8 [5, 11] | 15,339 [8,051, 27,990] | 16,771 [8,758, 31,308] |
| Middle | 24 [11, 45] | 26 [12, 49] | 2,555 [1,198, 4,719] | 2,793 [1,318, 5,198] | 2 [1, 3] | 2 [1, 3] | 162 [74, 306] | 177 [82, 332] | 165 [108, 244] | 180 [117, 267] | 17 [11, 26] | 19 [12, 28] | 3 [1, 5] | 3 [1, 6] | 0.3 [0.1, 0.5] | 0.3 [0.1, 0.6] | 168 [110, 247] | 183 [120, 271] | 18 [12, 26] | 19 [13, 29] | 6 [4, 9] | 6 [4, 10] | 11,090 [5,820, 20,236] | 12,125 [6,332, 22,635] |
| Higher | 22 [10, 40] | 24 [11, 44] | 2,305 [1,081, 4,259] | 2,520 [1,189, 4,691] | 1 [1, 3] | 1 [1, 3] | 140 [64, 265] | 153 [71, 288] | 130 [85, 191] | 142 [92, 209] | 14 [9, 20] | 15 [10, 22] | 8 [3, 15] | 8 [4, 16] | 0.8 [0.4, 1.6] | 0.9 [0.4, 1.7] | 137 [90, 202] | 150 [98, 221] | 14 [9, 21] | 16 [10, 23] | 5 [3, 7] | 5 [3, 8] | 17,379 [9,121, 31,712] | 19,001 [9,923, 35,471] |
| Highest | 17 [8, 31] | 18 [9, 34] | 1,753 [822, 3,239] | 1,917 [904, 3,567] | 1 [0, 1] | 1 [0, 2] | 78 [36, 148] | 85 [40, 160] | 135 [88, 199] | 147 [96, 218] | 14 [9, 21] | 16 [10, 23] | 3 [1, 5] | 3 [1, 6] | 0.3 [0.1, 0.6] | 0.3 [0.1, 0.6] | 137 [90, 203] | 150 [98, 222] | 15 [9, 21] | 16 [10, 23] | 5 [3, 7] | 5 [3, 8] | 30,211 [15,856, 55,126] | 33,031 [17,249, 61,662] |
| Masvingo | 112 [53, 210] | 123 [57, 231] | 2,975 [1,395, 5,557] | 3,253 [1,510, 6,104] | 13 [6, 24] | 14 [6, 27] | 339 [159, 644] | 371 [171, 702] | 1,087 [713, 1,622] | 1,188 [778, 1,768] | 29 [19, 43] | 31 [21, 47] | 81 [36, 160] | 88 [38, 173] | 2.1 [0.9, 4.2] | 2.3 [1.0, 4.6] | 1,167 [770, 1,738] | 1,276 [837, 1,895] | 31 [20, 46] | 34 [22, 50] | 41 [27, 61] | 45 [30, 67] | 16,646 [8,680, 30,008] | 18,200 [9,498, 32,886] |
| Lowest | 26 [12, 49] | 28 [13, 53] | 3,438 [1,612, 6,422] | 3,759 [1,745, 7,053] | 4 [2, 7] | 4 [2, 8] | 499 [233, 947] | 546 [251, 1,032] | 307 [201, 458] | 335 [219, 499] | 41 [27, 61] | 44 [29, 66] | 25 [11, 50] | 28 [12, 54] | 3.4 [1.5, 6.6] | 3.6 [1.6, 7.2] | 332 [219, 494] | 363 [238, 538] | 44 [29, 65] | 48 [31, 71] | 12 [8, 17] | 13 [8, 19] | 11,211 [5,846, 20,210] | 12,257 [6,397, 22,147] |
| Lower | 25 [12, 46] | 27 [13, 51] | 3,260 [1,528, 6,090] | 3,565 [1,654, 6,689] | 4 [2, 7] | 4 [2, 8] | 519 [243, 985] | 568 [261, 1,074] | 244 [160, 365] | 267 [175, 398] | 32 [21, 48] | 35 [23, 53] | 27 [12, 54] | 30 [13, 58] | 3.6 [1.6, 7.1] | 3.9 [1.7, 7.7] | 272 [179, 404] | 297 [195, 441] | 36 [24, 53] | 39 [26, 58] | 10 [6, 14] | 10 [7, 16] | 14,957 [7,799, 26,963] | 16,353 [8,535, 29,548] |
| Middle | 24 [11, 45] | 26 [12, 49] | 3,156 [1,479, 5,894] | 3,450 [1,601, 6,474] | 2 [1, 3] | 2 [1, 4] | 244 [114, 462] | 266 [122, 503] | 258 [169, 385] | 282 [185, 420] | 34 [22, 51] | 37 [24, 56] | 10 [4, 19] | 10 [4, 20] | 1.3 [0.6, 2.5] | 1.4 [0.6, 2.7] | 268 [176, 399] | 293 [193, 436] | 35 [23, 53] | 39 [25, 58] | 9 [6, 14] | 10 [7, 15] | 10,814 [5,639, 19,494] | 11,823 [6,170, 21,363] |
| Higher | 23 [11, 42] | 25 [11, 46] | 2,986 [1,400, 5,577] | 3,264 [1,515, 6,125] | 2 [1, 4] | 2 [1, 4] | 264 [123, 500] | 288 [133, 545] | 175 [115, 261] | 191 [125, 284] | 23 [15, 35] | 25 [17, 38] | 11 [5, 21] | 12 [5, 23] | 1.4 [0.6, 2.8] | 1.5 [0.7, 3.0] | 185 [122, 276] | 203 [133, 301] | 25 [16, 37] | 27 [18, 40] | 7 [4, 10] | 7 [5, 11] | 17,024 [8,877, 30,689] | 18,613 [9,714, 33,632] |
| Highest | 15 [7, 29] | 17 [8, 32] | 2,036 [954, 3,802] | 2,226 [1,033, 4,176] | 1 [1, 2] | 1 [1, 3] | 171 [80, 325] | 187 [86, 354] | 103 [68, 154] | 113 [74, 167] | 14 [9, 20] | 15 [10, 22] | 8 [4, 16] | 9 [4, 17] | 1.1 [0.5, 2.1] | 1.1 [0.5, 2.3] | 111 [73, 165] | 121 [80, 180] | 15 [10, 22] | 16 [11, 24] | 4 [3, 6] | 4 [3, 6] | 29,226 [15,239, 52,685] | 31,954 [16,676, 57,737] |
| Midlands | 122 [57, 231] | 134 [62, 247] | 2,917 [1,346, 5,500] | 3,189 [1,472, 5,882] | 16 [7, 30] | 17 [8, 33] | 378 [173, 724] | 414 [188, 777] | 1,529 [1,003, 2,282] | 1,672 [1,093, 2,490] | 36 [24, 54] | 40 [26, 59] | 136 [59, 270] | 148 [64, 294] | 3.2 [1.4, 6.4] | 3.5 [1.5, 7.0] | 1,665 [1,095, 2,492] | 1,820 [1,197, 2,700] | 40 [26, 59] | 43 [29, 64] | 59 [39, 88] | 64 [42, 95] | 9,476 [4,940, 17,387] | 10,360 [5,378, 18,920] |
| Lowest | 28 [13, 53] | 31 [14, 57] | 3,347 [1,545, 6,312] | 3,660 [1,690, 6,750] | 4 [2, 8] | 5 [2, 8] | 490 [224, 937] | 536 [244, 1,007] | 360 [236, 537] | 393 [257, 586] | 43 [28, 64] | 47 [31, 70] | 31 [13, 61] | 34 [15, 67] | 3.7 [1.6, 7.3] | 4.0 [1.7, 7.9] | 391 [257, 584] | 427 [281, 634] | 47 [31, 70] | 51 [33, 75] | 14 [9, 21] | 15 [10, 22] | 4,365 [2,275, 8,009] | 4,772 [2,477, 8,715] |
| Lower | 27 [12, 51] | 29 [14, 54] | 3,190 [1,472, 6,015] | 3,488 [1,610, 6,433] | 3 [2, 6] | 4 [2, 7] | 393 [180, 752] | 430 [196, 807] | 377 [247, 562] | 412 [269, 613] | 45 [29, 67] | 49 [32, 73] | 27 [12, 54] | 30 [13, 59] | 3.3 [1.4, 6.5] | 3.5 [1.5, 7.1] | 404 [266, 604] | 442 [291, 655] | 48 [32, 72] | 53 [35, 78] | 14 [9, 21] | 16 [10, 23] | 8,086 [4,215, 14,837] | 8,840 [4,589, 16,145] |
| Middle | 26 [12, 49] | 28 [13, 52] | 3,074 [1,418, 5,796] | 3,360 [1,552, 6,198] | 4 [2, 7] | 4 [2, 8] | 446 [204, 853] | 488 [222, 917] | 326 [214, 486] | 356 [233, 531] | 39 [25, 58] | 42 [28, 63] | 31 [13, 62] | 34 [15, 67] | 3.7 [1.6, 7.3] | 4.0 [1.7, 8.0] | 357 [235, 535] | 390 [257, 579] | 43 [28, 64] | 46 [31, 69] | 13 [8, 19] | 14 [9, 20] | 3,762 [1,961, 6,903] | 4,113 [2,135, 7,511] |
| Higher | 24 [11, 45] | 26 [12, 48] | 2,851 [1,316, 5,376] | 3,117 [1,439, 5,749] | 3 [1, 5] | 3 [1, 5] | 319 [146, 609] | 348 [159, 654] | 240 [157, 357] | 262 [171, 390] | 29 [19, 43] | 31 [20, 46] | 29 [13, 57] | 31 [14, 63] | 3.5 [1.5, 6.8] | 3.7 [1.6, 7.4] | 269 [177, 402] | 293 [193, 436] | 32 [21, 48] | 35 [23, 52] | 9 [6, 14] | 10 [7, 15] | 9,587 [4,998, 17,592] | 10,482 [5,442, 19,142] |
| Highest | 18 [8, 34] | 19 [9, 36] | 2,121 [979, 4,000] | 2,319 [1,071, 4,278] | 2 [1, 4] | 2 [1, 4] | 244 [112, 467] | 267 [122, 501] | 227 [149, 339] | 248 [162, 370] | 27 [18, 40] | 30 [19, 44] | 18 [8, 35] | 19 [8, 38] | 2.1 [0.9, 4.2] | 2.3 [1.0, 4.6] | 245 [161, 366] | 268 [176, 397] | 29 [19, 44] | 32 [21, 47] | 9 [6, 13] | 9 [6, 14] | 21,580 [11,249, 39,596] | 23,594 [12,248, 43,087] |
| Matabeleland South | 49 [23, 92] | 54 [25, 102] | 2,867 [1,346, 5,335] | 3,134 [1,469, 5,956] | 6 [3, 12] | 7 [3, 13] | 361 [166, 684] | 395 [183, 758] | 370 [243, 547] | 405 [265, 602] | 22 [14, 32] | 24 [15, 35] | 33 [15, 65] | 36 [16, 72] | 1.9 [0.9, 3.8] | 2.1 [0.9, 4.2] | 404 [266, 598] | 441 [291, 654] | 23 [15, 35] | 26 [17, 38] | 14 [9, 21] | 16 [10, 23] | 16,298 [8,454, 29,452] | 17,819 [9,197, 32,693] |
| Lowest | 11 [5, 20] | 12 [6, 22] | 3,132 [1,471, 5,828] | 3,424 [1,605, 6,506] | 2 [1, 3] | 2 [1, 4] | 499 [230, 944] | 545 [253, 1,047] | 98 [64, 145] | 108 [70, 160] | 29 [19, 42] | 31 [20, 46] | 10 [4, 19] | 10 [5, 21] | 2.8 [1.2, 5.5] | 3.0 [1.4, 6.0] | 108 [71, 160] | 118 [78, 175] | 31 [21, 46] | 34 [23, 51] | 4 [3, 6] | 4 [3, 6] | 11,034 [5,723, 19,939] | 12,064 [6,226, 22,133] |
| Lower | 10 [5, 19] | 11 [5, 21] | 3,005 [1,411, 5,593] | 3,286 [1,540, 6,244] | 1 [1, 3] | 2 [1, 3] | 406 [187, 768] | 444 [206, 851] | 88 [58, 131] | 97 [63, 143] | 26 [17, 38] | 28 [18, 42] | 10 [4, 19] | 10 [5, 21] | 2.8 [1.2, 5.4] | 3.0 [1.3, 6.0] | 98 [65, 145] | 107 [70, 158] | 28 [19, 42] | 31 [20, 46] | 3 [2, 5] | 4 [2, 6] | 14,755 [7,653, 26,662] | 16,132 [8,326, 29,596] |
| Middle | 10 [5, 19] | 11 [5, 21] | 2,920 [1,371, 5,434] | 3,192 [1,496, 6,066] | 1 [1, 3] | 1 [1, 3] | 397 [183, 751] | 434 [202, 833] | 67 [44, 99] | 73 [48, 109] | 20 [13, 29] | 21 [14, 32] | 9 [4, 17] | 9 [4, 18] | 2.5 [1.1, 4.8] | 2.7 [1.2, 5.3] | 76 [50, 112] | 83 [54, 122] | 22 [14, 33] | 24 [16, 36] | 3 [2, 4] | 3 [2, 4] | 10,521 [5,457, 19,012] | 11,503 [5,937, 21,104] |
| Higher | 10 [5, 19] | 11 [5, 21] | 2,898 [1,361, 5,392] | 3,168 [1,485, 6,020] | 1 [1, 2] | 1 [1, 3] | 347 [160, 656] | 379 [176, 728] | 70 [46, 104] | 77 [50, 114] | 20 [13, 30] | 22 [15, 33] | 4 [2, 7] | 4 [2, 8] | 1.0 [0.4, 2.0] | 1.1 [0.5, 2.2] | 74 [48, 108] | 80 [53, 119] | 21 [14, 32] | 23 [15, 35] | 3 [2, 4] | 3 [2, 4] | 16,673 [8,648, 30,129] | 18,229 [9,408, 33,444] |
| Highest | 8 [4, 15] | 9 [4, 17] | 2,379 [1,117, 4,427] | 2,601 [1,219, 4,942] | 1 [0, 1] | 1 [0, 1] | 159 [73, 301] | 174 [81, 334] | 47 [30, 69] | 51 [33, 76] | 14 [9, 20] | 15 [10, 22] | 2 [1, 4] | 2 [1, 5] | 0.6 [0.3, 1.3] | 0.7 [0.3, 1.4] | 49 [32, 72] | 53 [35, 79] | 14 [9, 21] | 15 [10, 23] | 2 [1, 3] | 2 [1, 3] | 28,509 [14,787, 51,518] | 31,170 [16,087, 57,187] |
| Matabeleland North | 59 [27, 109] | 64 [30, 119] | 3,007 [1,408, 5,573] | 3,288 [1,540, 6,081] | 6 [3, 11] | 6 [3, 12] | 292 [134, 549] | 319 [148, 608] | 318 [208, 474] | 347 [227, 516] | 16 [11, 24] | 18 [12, 26] | 21 [9, 41] | 23 [10, 44] | 1.1 [0.5, 2.1] | 1.2 [0.5, 2.3] | 339 [224, 502] | 370 [242, 548] | 17 [11, 26] | 19 [12, 28] | 12 [8, 18] | 13 [9, 19] | 30,945 [16,192, 56,583] | 33,833 [17,742, 61,942] |
| Lowest | 13 [6, 23] | 14 [6, 26] | 3,248 [1,520, 6,019] | 3,551 [1,664, 6,567] | 1 [1, 3] | 2 [1, 3] | 352 [162, 662] | 385 [179, 733] | 81 [53, 121] | 89 [58, 132] | 21 [14, 31] | 23 [15, 34] | 5 [2, 10] | 5 [2, 10] | 1.3 [0.6, 2.5] | 1.4 [0.6, 2.7] | 86 [57, 127] | 94 [62, 139] | 22 [15, 33] | 24 [16, 36] | 3 [2, 5] | 3 [2, 5] | 25,539 [13,363, 46,698] | 27,923 [14,643, 51,121] |
| Lower | 12 [6, 23] | 14 [6, 25] | 3,165 [1,482, 5,866] | 3,461 [1,621, 6,400] | 1 [0, 1] | 1 [0, 2] | 200 [92, 377] | 219 [102, 417] | 61 [40, 91] | 67 [44, 99] | 16 [10, 23] | 17 [11, 25] | 1 [1, 3] | 1 [1, 3] | 0.3 [0.1, 0.7] | 0.4 [0.2, 0.7] | 62 [41, 93] | 68 [45, 101] | 16 [11, 24] | 17 [11, 26] | 2 [1, 3] | 2 [2, 4] | 29,260 [15,310, 53,502] | 31,991 [16,776, 58,570] |
| Middle | 13 [6, 23] | 14 [6, 25] | 3,209 [1,502, 5,947] | 3,509 [1,644, 6,489] | 2 [1, 4] | 2 [1, 4] | 490 [225, 921] | 535 [248, 1,019] | 78 [51, 116] | 85 [56, 126] | 20 [13, 30] | 22 [14, 32] | 8 [4, 16] | 9 [4, 18] | 2.1 [1.0, 4.2] | 2.3 [1.0, 4.5] | 86 [57, 127] | 94 [62, 140] | 22 [15, 33] | 24 [16, 36] | 3 [2, 4] | 3 [2, 5] | 25,120 [13,144, 45,932] | 27,465 [14,403, 50,283] |
| Higher | 12 [6, 22] | 13 [6, 24] | 3,033 [1,419, 5,620] | 3,316 [1,553, 6,132] | 1 [0, 2] | 1 [1, 2] | 268 [123, 505] | 293 [136, 558] | 58 [38, 87] | 64 [42, 95] | 15 [10, 22] | 16 [11, 24] | 5 [2, 9] | 5 [2, 10] | 1.2 [0.5, 2.4] | 1.3 [0.6, 2.6] | 63 [42, 94] | 69 [45, 102] | 16 [11, 24] | 18 [12, 26] | 2 [1, 3] | 2 [2, 4] | 31,425 [16,443, 57,462] | 34,358 [18,018, 62,905] |
| Highest | 9 [4, 17] | 10 [5, 19] | 2,381 [1,114, 4,413] | 2,603 [1,220, 4,815] | 1 [0, 1] | 1 [0, 1] | 149 [69, 281] | 163 [76, 310] | 39 [26, 59] | 43 [28, 64] | 10 [7, 15] | 11 [7, 16] | 2 [1, 3] | 2 [1, 3] | 0.4 [0.2, 0.8] | 0.5 [0.2, 0.9] | 41 [27, 61] | 45 [29, 66] | 10 [7, 16] | 11 [8, 17] | 1 [1, 2] | 2 [1, 2] | 43,380 [22,698, 79,320] | 47,428 [24,872, 86,833] |
| Mashonaland West | 109 [51, 209] | 120 [56, 230] | 2,964 [1,391, 5,666] | 3,241 [1,527, 6,228] | 15 [7, 29] | 16 [7, 31] | 398 [184, 775] | 435 [200, 839] | 1,129 [744, 1,680] | 1,234 [814, 1,837] | 31 [20, 45] | 33 [22, 50] | 110 [49, 218] | 119 [53, 236] | 3.0 [1.3, 5.9] | 3.2 [1.4, 6.4] | 1,238 [819, 1,827] | 1,353 [898, 2,006] | 34 [22, 49] | 37 [24, 54] | 44 [29, 65] | 48 [32, 71] | 8,348 [4,364, 15,621] | 9,127 [4,760, 16,796] |
| Lowest | 24 [11, 47] | 27 [13, 51] | 3,299 [1,548, 6,306] | 3,607 [1,699, 6,931] | 3 [1, 6] | 3 [2, 7] | 433 [200, 843] | 474 [218, 913] | 264 [174, 392] | 288 [190, 429] | 36 [24, 53] | 39 [26, 58] | 24 [11, 49] | 27 [12, 53] | 3.3 [1.5, 6.6] | 3.6 [1.6, 7.1] | 288 [190, 425] | 315 [209, 467] | 39 [26, 58] | 43 [28, 63] | 10 [7, 15] | 11 [7, 16] | 3,224 [1,685, 6,032] | 3,525 [1,838, 6,486] |
| Lower | 24 [11, 46] | 26 [12, 50] | 3,230 [1,516, 6,175] | 3,532 [1,664, 6,786] | 4 [2, 8] | 5 [2, 9] | 584 [269, 1,136] | 638 [294, 1,230] | 288 [190, 429] | 315 [208, 470] | 39 [26, 58] | 43 [28, 64] | 39 [17, 77] | 42 [19, 83] | 5.2 [2.3, 10.4] | 5.7 [2.5, 11.2] | 327 [216, 484] | 357 [238, 530] | 44 [29, 66] | 48 [32, 72] | 12 [8, 17] | 13 [8, 19] | 6,945 [3,631, 12,995] | 7,593 [3,960, 13,973] |
| Middle | 23 [11, 43] | 25 [12, 48] | 3,077 [1,444, 5,882] | 3,364 [1,585, 6,465] | 3 [1, 6] | 3 [2, 7] | 428 [197, 833] | 468 [215, 902] | 270 [178, 402] | 295 [194, 439] | 37 [24, 54] | 40 [26, 59] | 23 [10, 46] | 25 [11, 50] | 3.2 [1.4, 6.3] | 3.4 [1.5, 6.8] | 293 [194, 433] | 320 [212, 474] | 40 [26, 59] | 43 [29, 64] | 10 [7, 15] | 11 [8, 17] | 2,521 [1,318, 4,717] | 2,756 [1,437, 5,072] |
| Higher | 21 [10, 40] | 23 [11, 44] | 2,832 [1,329, 5,414] | 3,096 [1,459, 5,950] | 2 [1, 4] | 2 [1, 4] | 287 [132, 559] | 314 [145, 606] | 173 [114, 258] | 189 [125, 282] | 23 [15, 35] | 26 [17, 38] | 11 [5, 23] | 12 [5, 25] | 1.5 [0.7, 3.1] | 1.7 [0.7, 3.3] | 185 [122, 273] | 202 [134, 300] | 25 [17, 37] | 27 [18, 41] | 7 [4, 10] | 7 [5, 11] | 8,560 [4,475, 16,018] | 9,359 [4,881, 17,223] |
| Highest | 18 [8, 34] | 19 [9, 37] | 2,383 [1,118, 4,555] | 2,605 [1,227, 5,007] | 2 [1, 4] | 2 [1, 4] | 258 [119, 503] | 283 [130, 545] | 133 [88, 199] | 146 [96, 217] | 18 [12, 27] | 20 [13, 29] | 12 [5, 24] | 13 [6, 26] | 1.6 [0.7, 3.2] | 1.8 [0.8, 3.5] | 145 [96, 215] | 159 [105, 236] | 20 [13, 29] | 22 [14, 32] | 5 [3, 8] | 6 [4, 8] | 20,489 [10,712, 38,340] | 22,402 [11,684, 41,226] |
| Mashonaland East | 115 [54, 216] | 126 [59, 233] | 2,903 [1,356, 5,450] | 3,174 [1,483, 5,895] | 13 [6, 25] | 14 [7, 27] | 329 [151, 626] | 360 [165, 682] | 1,184 [778, 1,761] | 1,294 [839, 1,939] | 30 [20, 45] | 33 [21, 49] | 93 [40, 184] | 101 [44, 197] | 2.3 [1.0, 4.7] | 2.5 [1.1, 5.0] | 1,277 [843, 1,892] | 1,395 [907, 2,077] | 32 [21, 48] | 35 [23, 53] | 45 [30, 67] | 49 [32, 73] | 11,571 [5,998, 20,974] | 12,651 [6,545, 23,103] |
| Lowest | 26 [12, 49] | 29 [13, 53] | 3,310 [1,546, 6,215] | 3,619 [1,692, 6,722] | 4 [2, 7] | 4 [2, 8] | 485 [222, 923] | 530 [243, 1,004] | 357 [234, 530] | 390 [253, 584] | 45 [30, 67] | 49 [32, 74] | 27 [12, 54] | 29 [13, 58] | 3.4 [1.5, 6.8] | 3.7 [1.6, 7.3] | 384 [253, 569] | 419 [273, 625] | 49 [32, 72] | 53 [34, 79] | 14 [9, 20] | 15 [10, 22] | 6,221 [3,224, 11,275] | 6,801 [3,518, 12,420] |
| Lower | 24 [11, 46] | 27 [12, 49] | 3,071 [1,434, 5,766] | 3,358 [1,569, 6,237] | 2 [1, 4] | 2 [1, 4] | 271 [125, 516] | 297 [136, 562] | 226 [148, 336] | 247 [160, 370] | 29 [19, 42] | 31 [20, 47] | 13 [6, 27] | 15 [6, 29] | 1.7 [0.7, 3.4] | 1.8 [0.8, 3.6] | 239 [158, 354] | 261 [169, 390] | 30 [20, 45] | 33 [21, 49] | 8 [6, 13] | 9 [6, 14] | 9,941 [5,153, 18,019] | 10,869 [5,623, 19,849] |
| Middle | 24 [11, 44] | 26 [12, 48] | 2,984 [1,394, 5,601] | 3,262 [1,525, 6,059] | 2 [1, 5] | 3 [1, 5] | 312 [143, 594] | 341 [157, 646] | 219 [144, 325] | 239 [155, 358] | 28 [18, 41] | 30 [20, 45] | 17 [8, 35] | 19 [8, 37] | 2.2 [1.0, 4.4] | 2.4 [1.0, 4.7] | 236 [156, 350] | 258 [168, 384] | 30 [20, 44] | 33 [21, 49] | 8 [6, 12] | 9 [6, 14] | 5,751 [2,981, 10,423] | 6,287 [3,253, 11,481] |
| Higher | 23 [11, 42] | 25 [12, 46] | 2,862 [1,337, 5,372] | 3,129 [1,462, 5,811] | 3 [1, 6] | 3 [2, 6] | 393 [181, 748] | 430 [197, 815] | 215 [141, 319] | 235 [152, 352] | 27 [18, 40] | 30 [19, 44] | 25 [11, 50] | 27 [12, 54] | 3.2 [1.4, 6.3] | 3.5 [1.5, 6.8] | 240 [158, 356] | 262 [171, 391] | 30 [20, 45] | 33 [22, 49] | 8 [6, 13] | 9 [6, 14] | 11,885 [6,160, 21,542] | 12,994 [6,722, 23,729] |
| Highest | 18 [8, 34] | 20 [9, 37] | 2,288 [1,068, 4,295] | 2,501 [1,169, 4,645] | 1 [1, 3] | 2 [1, 3] | 184 [85, 350] | 201 [92, 381] | 168 [111, 250] | 184 [119, 276] | 21 [14, 32] | 23 [15, 35] | 10 [4, 19] | 10 [5, 20] | 1.2 [0.5, 2.4] | 1.3 [0.6, 2.6] | 178 [118, 264] | 194 [126, 290] | 22 [15, 33] | 25 [16, 37] | 6 [4, 9] | 7 [4, 10] | 24,060 [12,471, 43,609] | 26,306 [13,609, 48,037] |
| Mashonaland Central | 92 [43, 172] | 101 [48, 188] | 3,088 [1,453, 5,756] | 3,376 [1,596, 6,298] | 11 [5, 21] | 12 [6, 23] | 371 [172, 710] | 406 [188, 762] | 924 [598, 1,388] | 1,010 [662, 1,505] | 31 [20, 47] | 34 [22, 51] | 79 [35, 157] | 86 [38, 169] | 2.7 [1.2, 5.3] | 2.9 [1.3, 5.7] | 1,004 [654, 1,497] | 1,097 [722, 1,623] | 34 [22, 50] | 37 [24, 54] | 35 [23, 53] | 39 [25, 57] | 7,313 [3,859, 13,414] | 7,995 [4,210, 14,656] |
| Lowest | 20 [9, 37] | 22 [10, 40] | 3,323 [1,564, 6,196] | 3,634 [1,718, 6,779] | 2 [1, 4] | 2 [1, 4] | 348 [161, 666] | 380 [176, 714] | 205 [132, 307] | 224 [146, 333] | 34 [22, 52] | 38 [25, 56] | 12 [5, 23] | 13 [6, 25] | 2.0 [0.9, 3.9] | 2.1 [0.9, 4.2] | 216 [141, 324] | 236 [155, 350] | 36 [24, 54] | 40 [26, 59] | 8 [5, 11] | 8 [5, 12] | 1,834 [968, 3,365] | 2,006 [1,056, 3,676] |
| Lower | 19 [9, 36] | 21 [10, 40] | 3,255 [1,531, 6,068] | 3,559 [1,682, 6,639] | 3 [1, 6] | 3 [2, 6] | 518 [240, 991] | 567 [262, 1,064] | 211 [136, 316] | 230 [151, 343] | 35 [23, 53] | 39 [25, 58] | 25 [11, 49] | 27 [12, 52] | 4.1 [1.8, 8.2] | 4.5 [2.0, 8.8] | 235 [153, 352] | 257 [169, 380] | 39 [26, 59] | 43 [28, 64] | 8 [5, 12] | 9 [6, 13] | 5,555 [2,931, 10,190] | 6,074 [3,198, 11,134] |
| Middle | 19 [9, 35] | 21 [10, 39] | 3,169 [1,491, 5,908] | 3,465 [1,638, 6,464] | 2 [1, 4] | 3 [1, 5] | 391 [181, 747] | 427 [198, 801] | 210 [136, 315] | 229 [150, 341] | 35 [23, 53] | 38 [25, 57] | 18 [8, 36] | 20 [9, 38] | 3.0 [1.3, 6.0] | 3.3 [1.5, 6.4] | 228 [148, 340] | 249 [164, 368] | 38 [25, 57] | 42 [27, 62] | 8 [5, 12] | 9 [6, 13] | 1,409 [743, 2,584] | 1,540 [811, 2,823] |
| Higher | 18 [8, 33] | 19 [9, 36] | 2,978 [1,401, 5,552] | 3,256 [1,539, 6,074] | 2 [1, 4] | 3 [1, 5] | 391 [181, 748] | 428 [198, 802] | 188 [122, 282] | 206 [135, 306] | 32 [20, 47] | 34 [23, 51] | 17 [8, 35] | 19 [8, 37] | 2.9 [1.3, 5.8] | 3.2 [1.4, 6.2] | 205 [134, 307] | 224 [148, 332] | 34 [22, 51] | 38 [25, 56] | 7 [5, 11] | 8 [5, 12] | 7,647 [4,035, 14,026] | 8,360 [4,403, 15,325] |
| Highest | 16 [8, 30] | 18 [8, 33] | 2,713 [1,276, 5,057] | 2,966 [1,402, 5,533] | 1 [1, 2] | 1 [1, 3] | 209 [97, 400] | 229 [106, 429] | 111 [72, 167] | 122 [80, 181] | 19 [12, 28] | 20 [13, 30] | 7 [3, 15] | 8 [4, 16] | 1.2 [0.6, 2.5] | 1.4 [0.6, 2.7] | 119 [77, 178] | 130 [85, 192] | 20 [13, 30] | 22 [14, 32] | 4 [3, 6] | 5 [3, 7] | 20,118 [10,616, 36,904] | 21,996 [11,583, 40,322] |
| Manicaland | 127 [59, 240] | 139 [65, 256] | 2,871 [1,340, 5,423] | 3,139 [1,475, 5,785] | 17 [8, 32] | 18 [8, 34] | 376 [172, 723] | 411 [190, 774] | 1,394 [918, 2,088] | 1,524 [1,004, 2,262] | 31 [21, 47] | 34 [23, 51] | 130 [57, 253] | 141 [63, 277] | 2.9 [1.3, 5.7] | 3.2 [1.4, 6.3] | 1,523 [1,011, 2,259] | 1,665 [1,100, 2,471] | 34 [23, 51] | 38 [25, 56] | 54 [36, 80] | 59 [39, 87] | 16,043 [8,485, 29,487] | 17,540 [9,169, 32,189] |
| Lowest | 27 [13, 52] | 30 [14, 55] | 3,098 [1,446, 5,852] | 3,387 [1,592, 6,243] | 5 [2, 9] | 5 [2, 9] | 514 [236, 989] | 562 [259, 1,059] | 403 [265, 603] | 440 [290, 654] | 45 [30, 68] | 50 [33, 74] | 38 [17, 75] | 42 [19, 82] | 4.3 [1.9, 8.4] | 4.7 [2.1, 9.2] | 441 [293, 654] | 482 [318, 715] | 50 [33, 74] | 54 [36, 81] | 16 [10, 23] | 17 [11, 25] | 10,674 [5,646, 19,619] | 11,670 [6,101, 21,417] |
| Lower | 28 [13, 53] | 31 [15, 57] | 3,194 [1,491, 6,033] | 3,492 [1,641, 6,436] | 4 [2, 8] | 4 [2, 8] | 464 [213, 892] | 507 [234, 956] | 334 [220, 501] | 366 [241, 543] | 38 [25, 57] | 41 [27, 61] | 34 [15, 67] | 37 [17, 73] | 3.9 [1.7, 7.6] | 4.2 [1.9, 8.3] | 369 [245, 547] | 403 [266, 598] | 42 [28, 62] | 46 [30, 68] | 13 [9, 19] | 14 [9, 21] | 14,387 [7,610, 26,444] | 15,730 [8,223, 28,867] |
| Middle | 26 [12, 49] | 29 [13, 53] | 2,953 [1,379, 5,578] | 3,228 [1,518, 5,951] | 4 [2, 7] | 4 [2, 8] | 432 [198, 832] | 473 [218, 891] | 281 [185, 420] | 307 [202, 456] | 32 [21, 47] | 35 [23, 51] | 29 [13, 56] | 31 [14, 62] | 3.3 [1.4, 6.4] | 3.5 [1.6, 7.0] | 309 [205, 459] | 338 [223, 502] | 35 [23, 52] | 38 [25, 57] | 11 [7, 16] | 12 [8, 18] | 10,174 [5,381, 18,700] | 11,124 [5,815, 20,414] |
| Higher | 24 [11, 46] | 27 [12, 49] | 2,747 [1,282, 5,190] | 3,003 [1,412, 5,536] | 3 [1, 5] | 3 [1, 6] | 310 [142, 596] | 339 [157, 639] | 214 [141, 320] | 234 [154, 347] | 24 [16, 36] | 26 [17, 39] | 20 [9, 39] | 21 [10, 42] | 2.2 [1.0, 4.4] | 2.4 [1.1, 4.8] | 233 [155, 346] | 255 [168, 378] | 26 [17, 39] | 29 [19, 43] | 8 [5, 12] | 9 [6, 13] | 16,474 [8,713, 30,279] | 18,011 [9,416, 33,054] |
| Highest | 21 [10, 40] | 23 [11, 42] | 2,362 [1,103, 4,463] | 2,583 [1,214, 4,761] | 1 [1, 3] | 2 [1, 3] | 158 [72, 304] | 173 [80, 326] | 162 [107, 243] | 177 [117, 263] | 18 [12, 27] | 20 [13, 30] | 9 [4, 17] | 9 [4, 19] | 1.0 [0.4, 1.9] | 1.1 [0.5, 2.1] | 171 [113, 254] | 187 [123, 277] | 19 [13, 29] | 21 [14, 31] | 6 [4, 9] | 7 [4, 10] | 28,505 [15,077, 52,393] | 31,166 [16,292, 57,194] |
|  |  |  |  |  |  |  |  |  |  |  |  |  |  |  |  |  |  |  |  |  |  |  |  |  |

**Supplemental Table 3.** Regional and quintile estimates of health and economic impact and benefits of ETEC and Shigella vaccination from 2025-2034 in four East African countries. Abbreviations: DRC, Democratic Republic of Congo; ICER, Incremental Cost-Effectiveness Ratio. Costs and ICERs are presented in 2016 US$.

|  | Fully Vaccinated Children (1000s) | | MSD Diarrhoeal Episodes Averted (1000s) | | MSD Diarrhoeal Episodes Averted / 100,000 FVC | | Moderate and Severe Stunting Cases Due to MSD Diarrhoea Averted (1000s) | | Direct Diarrhoeal Deaths Averted | | Other ID Deaths Due to Diarrhoea-induced Stunting Averted | | Direct Diarrhoeal and Other ID mortality Averted due to Diarrhoea-induced Stunting Averted (Total Deaths Averted) | | Total Deaths Averted / 100,000 FVC | | Total DALYs Averted (1000s) | | Costs Averted (1000s 2016 US$) | | ICER ($/DALY averted) | | ICER ($/MSD episode averted) | |
| --- | --- | --- | --- | --- | --- | --- | --- | --- | --- | --- | --- | --- | --- | --- | --- | --- | --- | --- | --- | --- | --- | --- | --- | --- |
|  | Both* | *Both** | ETEC | *Shigella* | ETEC | *Shigella* | ETEC | *Shigella* | ETEC | *Shigella* | ETEC | *Shigella* | ETEC | *Shigella* | ETEC | *Shigella* | ETEC | *Shigella* | ETEC | *Shigella* | ETEC | *Shigella* | ETEC | *Shigella* |
| DRC |  |  |  |  |  |  |  |  |  |  |  |  |  |  |  |  |  |  |  |  |  |  |  |  |
| Sud-Kivu | 7,161 | 41% [30%; 51%] | 94 [41; 184] | 102 [45; 198] | 1,307 [577; 2,570] | 1,429 [626; 2,765] | 20 [9; 40] | 27 [12; 52] | 549 [265; 1,003] | 601 [291; 1,097] | 162 [67; 331] | 212 [90; 429] | 711 [363; 1,239] | 813 [421; 1,426] | 9.9 [5.1; 17.3] | 11.3 [5.9; 19.9] | 25 [13; 43] | 28 [15; 49] | 553 [235; 1,125] | 605 [257; 1,218] | 1,058 [538; 2,245] | 925 [476; 1,963] | 279 [127; 692] | 255 [117; 628] |
| Lowest | 1,393 | 40% [29%; 50%] | 19 [8; 37] | 20 [9; 39] | 1,333 [588; 2,622] | 1,458 [639; 2,822] | 5 [2; 9] | 6 [3; 12] | 153 [74; 280] | 168 [81; 306] | 40 [16; 81] | 52 [22; 105] | 193 [98; 337] | 220 [113; 384] | 13.9 [7.0; 24.2] | 15.8 [8.1; 27.6] | 7 [3; 12] | 8 [4; 13] | 98 [41; 198] | 107 [45; 215] | 759 [385; 1,618] | 667 [343; 1,413] | 274 [125; 679] | 250 [116; 616] |
| Lower | 1,577 | 45% [33%; 56%] | 21 [9; 42] | 23 [10; 45] | 1,340 [591; 2,636] | 1,466 [642; 2,836] | 5 [2; 11] | 7 [3; 14] | 129 [62; 235] | 141 [68; 257] | 45 [19; 92] | 59 [25; 119] | 174 [89; 303] | 200 [104; 351] | 11.0 [5.7; 19.2] | 12.7 [6.6; 22.2] | 6 [3; 10] | 7 [4; 12] | 75 [32; 153] | 82 [35; 166] | 960 [493; 2,022] | 835 [433; 1,761] | 274 [126; 677] | 251 [117; 615] |
| Middle | 1,339 | 38% [28%; 48%] | 18 [8; 35] | 19 [9; 38] | 1,329 [586; 2,612] | 1,453 [636; 2,811] | 3 [1; 6] | 4 [2; 8] | 87 [42; 158] | 95 [46; 173] | 17 [7; 34] | 22 [9; 44] | 103 [52; 183] | 116 [60; 205] | 7.7 [3.9; 13.6] | 8.7 [4.4; 15.3] | 4 [2; 6] | 4 [2; 7] | 97 [41; 197] | 106 [45; 213] | 1,361 [690; 2,915] | 1,206 [618; 2,583] | 275 [126; 681] | 251 [116; 618] |
| Higher | 1,476 | 42% [31%; 53%] | 19 [8; 38] | 21 [9; 40] | 1,296 [572; 2,548] | 1,416 [620; 2,741] | 5 [2; 9] | 6 [3; 12] | 118 [57; 215] | 129 [62; 235] | 41 [17; 83] | 53 [23; 108] | 159 [81; 276] | 182 [95; 320] | 10.7 [5.5; 18.7] | 12.3 [6.4; 21.6] | 6 [3; 10] | 6 [3; 11] | 111 [47; 225] | 121 [52; 244] | 980 [500; 2,069] | 852 [439; 1,802] | 282 [129; 699] | 257 [119; 634] |
| Highest | 1,376 | 39% [29%; 49%] | 17 [7; 33] | 19 [8; 36] | 1,232 [544; 2,424] | 1,348 [590; 2,608] | 2 [1; 5] | 3 [1; 6] | 63 [30; 114] | 69 [33; 125] | 20 [8; 41] | 26 [11; 52] | 82 [42; 144] | 94 [49; 165] | 6.0 [3.1; 10.4] | 6.9 [3.6; 12.0] | 3 [1; 5] | 3 [2; 6] | 173 [73; 352] | 189 [80; 380] | 1,730 [871; 3,682] | 1,508 [767; 3,210] | 292 [131; 730] | 266 [121; 662] |
| Orientale | 16,552 | 24% [17%; 29%] | 209 [93; 408] | 229 [101; 443] | 1,265 [562; 2,464] | 1,383 [609; 2,674] | 41 [18; 81] | 53 [23; 105] | 1,132 [552; 2,072] | 1,237 [600; 2,240] | 258 [108; 527] | 337 [141; 672] | 1,390 [722; 2,463] | 1,575 [819; 2,759] | 8.4 [4.4; 14.9] | 9.5 [4.9; 16.7] | 48 [25; 86] | 55 [28; 96] | 2,012 [864; 4,056] | 2,200 [938; 4,391] | 1,234 [611; 2,606] | 1,087 [546; 2,302] | 285 [128; 701] | 260 [117; 639] |
| Lowest | 3,425 | 26% [19%; 32%] | 46 [20; 89] | 50 [22; 96] | 1,330 [591; 2,592] | 1,454 [640; 2,812] | 11 [5; 22] | 15 [6; 29] | 266 [130; 486] | 291 [141; 526] | 79 [33; 161] | 104 [43; 206] | 345 [180; 608] | 394 [205; 691] | 10.1 [5.3; 17.8] | 11.5 [6.0; 20.2] | 12 [6; 21] | 14 [7; 24] | 372 [160; 749] | 406 [173; 811] | 1,034 [518; 2,174] | 903 [455; 1,897] | 272 [123; 668] | 248 [112; 609] |
| Lower | 2,533 | 19% [14%; 23%] | 33 [15; 64] | 36 [16; 69] | 1,292 [574; 2,517] | 1,412 [622; 2,731] | 6 [3; 12] | 8 [3; 15] | 234 [114; 428] | 256 [124; 463] | 35 [15; 71] | 45 [19; 91] | 269 [138; 480] | 301 [155; 534] | 10.6 [5.4; 18.9] | 11.9 [6.1; 21.1] | 9 [5; 17] | 10 [5; 19] | 219 [94; 441] | 239 [102; 477] | 985 [492; 2,104] | 878 [441; 1,866] | 282 [128; 689] | 257 [117; 628] |
| Middle | 2,586 | 19% [14%; 24%] | 33 [15; 65] | 36 [16; 70] | 1,288 [572; 2,509] | 1,408 [620; 2,723] | 7 [3; 14] | 9 [4; 19] | 220 [107; 403] | 241 [117; 436] | 47 [20; 96] | 62 [26; 123] | 267 [138; 475] | 302 [157; 531] | 10.3 [5.3; 18.4] | 11.7 [6.1; 20.5] | 9 [5; 17] | 11 [5; 18] | 287 [123; 579] | 314 [134; 627] | 1,005 [498; 2,124] | 887 [446; 1,885] | 281 [127; 690] | 256 [115; 629] |
| Higher | 3,613 | 27% [20%; 33%] | 46 [20; 89] | 50 [22; 97] | 1,264 [562; 2,463] | 1,382 [609; 2,673] | 9 [4; 18] | 12 [5; 23] | 226 [110; 414] | 247 [120; 448] | 54 [23; 110] | 71 [29; 141] | 280 [146; 496] | 318 [165; 557] | 7.8 [4.0; 13.7] | 8.8 [4.6; 15.4] | 10 [5; 17] | 11 [6; 19] | 411 [176; 829] | 449 [192; 897] | 1,340 [664; 2,824] | 1,178 [593; 2,491] | 286 [129; 702] | 261 [117; 640] |
| Highest | 4,396 | 33% [24%; 41%] | 52 [23; 102] | 57 [25; 110] | 1,186 [527; 2,310] | 1,296 [571; 2,507] | 8 [3; 15] | 10 [4; 19] | 186 [91; 340] | 203 [98; 368] | 43 [18; 88] | 56 [23; 112] | 229 [119; 405] | 259 [135; 455] | 5.2 [2.7; 9.2] | 5.9 [3.1; 10.4] | 8 [4; 14] | 9 [5; 16] | 724 [311; 1,459] | 791 [337; 1,579] | 1,967 [971; 4,171] | 1,730 [860; 3,678] | 300 [133; 744] | 273 [121; 678] |
| Nord-Kivu | 11,169 | 46% [34%; 57%] | 138 [60; 266] | 151 [66; 292] | 1,235 [536; 2,384] | 1,350 [588; 2,618] | 29 [12; 57] | 38 [16; 75] | 650 [316; 1,173] | 710 [345; 1,299] | 130 [54; 262] | 170 [71; 344] | 780 [396; 1,374] | 880 [446; 1,555] | 7.0 [3.5; 12.3] | 7.9 [4.0; 13.9] | 27 [14; 48] | 31 [15; 54] | 692 [292; 1,383] | 756 [323; 1,522] | 1,509 [765; 3,218] | 1,335 [682; 2,835] | 297 [139; 727] | 271 [127; 663] |
| Lowest | 2,276 | 48% [35%; 59%] | 29 [12; 55] | 31 [14; 61] | 1,261 [547; 2,435] | 1,379 [600; 2,674] | 7 [3; 14] | 10 [4; 19] | 184 [89; 331] | 201 [98; 367] | 35 [15; 71] | 46 [19; 94] | 219 [111; 386] | 247 [125; 437] | 9.6 [4.9; 17.0] | 10.9 [5.5; 19.2] | 8 [4; 13] | 9 [4; 15] | 121 [51; 242] | 132 [56; 266] | 1,097 [556; 2,338] | 971 [497; 2,059] | 291 [137; 713] | 266 [125; 650] |
| Lower | 2,021 | 42% [31%; 52%] | 26 [11; 50] | 28 [12; 55] | 1,283 [557; 2,477] | 1,403 [611; 2,720] | 7 [3; 13] | 9 [4; 17] | 166 [81; 299] | 181 [88; 331] | 32 [13; 64] | 42 [18; 85] | 198 [100; 348] | 223 [113; 394] | 9.8 [5.0; 17.2] | 11.0 [5.6; 19.5] | 7 [3; 12] | 8 [4; 14] | 62 [26; 124] | 68 [29; 137] | 1,086 [552; 2,315] | 962 [493; 2,038] | 288 [136; 703] | 263 [125; 641] |
| Middle | 2,218 | 46% [34%; 57%] | 27 [12; 52] | 30 [13; 57] | 1,221 [530; 2,357] | 1,335 [581; 2,588] | 6 [3; 12] | 8 [3; 16] | 124 [60; 223] | 135 [66; 247] | 26 [11; 53] | 34 [14; 69] | 150 [76; 264] | 169 [86; 299] | 6.8 [3.4; 11.9] | 7.6 [3.9; 13.5] | 5 [3; 9] | 6 [3; 10] | 123 [52; 247] | 135 [58; 271] | 1,562 [793; 3,327] | 1,381 [706; 2,931] | 301 [141; 736] | 275 [129; 671] |
| Higher | 2,516 | 53% [38%; 65%] | 30 [13; 59] | 33 [15; 65] | 1,212 [526; 2,339] | 1,325 [577; 2,568] | 6 [3; 13] | 8 [4; 17] | 113 [55; 203] | 123 [60; 225] | 29 [12; 58] | 38 [16; 77] | 142 [72; 249] | 161 [82; 283] | 5.6 [2.9; 9.9] | 6.4 [3.2; 11.3] | 5 [3; 9] | 6 [3; 10] | 149 [63; 298] | 163 [70; 328] | 1,874 [954; 3,962] | 1,648 [836; 3,497] | 303 [142; 741] | 276 [129; 676] |
| Highest | 2,139 | 45% [32%; 55%] | 26 [11; 50] | 28 [12; 55] | 1,204 [522; 2,324] | 1,316 [573; 2,551] | 3 [1; 5] | 3 [1; 7] | 64 [31; 116] | 70 [34; 128] | 7 [3; 15] | 10 [4; 20] | 71 [36; 127] | 80 [40; 142] | 3.3 [1.7; 5.9] | 3.7 [1.9; 6.7] | 2 [1; 4] | 3 [1; 5] | 236 [100; 472] | 258 [110; 520] | 3,107 [1,553; 6,752] | 2,780 [1,394; 5,993] | 300 [138; 743] | 274 [126; 676] |
| Maniema | 2,479 | 27% [20%; 33%] | 31 [14; 60] | 34 [15; 66] | 1,259 [554; 2,421] | 1,377 [613; 2,654] | 6 [2; 11] | 7 [3; 14] | 162 [80; 293] | 178 [86; 322] | 30 [13; 60] | 39 [17; 78] | 192 [99; 339] | 217 [112; 382] | 7.8 [4.0; 13.7] | 8.7 [4.5; 15.4] | 7 [3; 12] | 8 [4; 13] | 284 [121; 569] | 311 [134; 623] | 1,337 [672; 2,866] | 1,185 [596; 2,534] | 287 [129; 701] | 262 [119; 636] |
| Lowest | 531 | 29% [21%; 36%] | 7 [3; 13] | 7 [3; 14] | 1,272 [560; 2,446] | 1,391 [619; 2,681] | 1 [1; 3] | 2 [1; 4] | 46 [23; 84] | 51 [25; 92] | 7 [3; 15] | 10 [4; 19] | 54 [28; 95] | 60 [31; 107] | 10.1 [5.2; 17.9] | 11.4 [5.8; 20.1] | 2 [1; 3] | 2 [1; 4] | 56 [24; 111] | 61 [26; 122] | 1,027 [517; 2,205] | 913 [460; 1,951] | 285 [128; 695] | 260 [119; 630] |
| Lower | 504 | 27% [20%; 34%] | 6 [3; 12] | 7 [3; 13] | 1,221 [537; 2,349] | 1,335 [594; 2,575] | 1 [0; 2] | 1 [1; 3] | 30 [15; 55] | 33 [16; 60] | 5 [2; 11] | 7 [3; 14] | 36 [18; 63] | 40 [21; 71] | 7.1 [3.6; 12.5] | 8.0 [4.1; 14.1] | 1 [1; 2] | 1 [1; 2] | 41 [18; 83] | 45 [20; 91] | 1,476 [748; 3,154] | 1,310 [664; 2,790] | 298 [135; 726] | 272 [126; 658] |
| Middle | 410 | 22% [16%; 27%] | 5 [2; 10] | 6 [3; 11] | 1,275 [561; 2,452] | 1,394 [620; 2,688] | 1 [0; 2] | 1 [1; 3] | 28 [14; 51] | 31 [15; 56] | 6 [3; 12] | 8 [3; 16] | 35 [18; 61] | 39 [20; 69] | 8.5 [4.4; 14.9] | 9.6 [5.0; 16.8] | 1 [1; 2] | 1 [1; 2] | 44 [19; 88] | 48 [21; 96] | 1,229 [619; 2,613] | 1,084 [548; 2,307] | 284 [128; 693] | 259 [118; 628] |
| Higher | 459 | 25% [18%; 31%] | 6 [3; 11] | 6 [3; 12] | 1,276 [561; 2,454] | 1,395 [621; 2,690] | 1 [0; 2] | 1 [1; 3] | 22 [11; 40] | 24 [12; 44] | 5 [2; 10] | 7 [3; 14] | 27 [14; 48] | 31 [16; 55] | 6.0 [3.1; 10.5] | 6.8 [3.5; 11.9] | 1 [0; 2] | 1 [1; 2] | 51 [21; 101] | 55 [24; 111] | 1,737 [873; 3,685] | 1,529 [772; 3,256] | 283 [127; 692] | 258 [118; 627] |
| Highest | 575 | 31% [23%; 39%] | 7 [3; 14] | 8 [4; 15] | 1,255 [552; 2,413] | 1,372 [611; 2,645] | 1 [0; 2] | 1 [1; 3] | 35 [17; 63] | 38 [19; 69] | 6 [2; 11] | 7 [3; 15] | 41 [21; 72] | 46 [23; 81] | 7.1 [3.6; 12.5] | 7.9 [4.1; 14.0] | 1 [1; 3] | 2 [1; 3] | 93 [39; 186] | 101 [44; 203] | 1,450 [719; 3,140] | 1,287 [641; 2,776] | 284 [125; 699] | 259 [116; 634] |
| Kinshasa | 17,236 | 48% [33%; 58%] | 203 [80; 361] | 221 [87; 393] | 1,175 [463; 2,092] | 1,285 [504; 2,280] | 14 [5; 25] | 18 [7; 32] | 523 [235; 870] | 571 [258; 962] | 33 [13; 61] | 43 [16; 79] | 556 [261; 915] | 615 [287; 1,018] | 3.2 [1.5; 5.3] | 3.6 [1.7; 5.9] | 19 [9; 32] | 21 [10; 36] | 2,422 [941; 4,461] | 2,648 [1,026; 4,876] | 3,186 [1,439; 6,296] | 2,871 [1,275; 5,639] | 305 [123; 676] | 278 [111; 621] |
| Lowest | 2,892 | 40% [28%; 49%] | 36 [14; 65] | 40 [16; 70] | 1,254 [494; 2,231] | 1,371 [538; 2,432] | 4 [2; 7] | 5 [2; 9] | 96 [43; 160] | 105 [47; 177] | 7 [3; 13] | 9 [3; 16] | 103 [49; 169] | 114 [53; 189] | 3.6 [1.7; 5.9] | 3.9 [1.8; 6.5] | 4 [2; 6] | 4 [2; 7] | 380 [148; 701] | 416 [161; 766] | 2,895 [1,309; 5,705] | 2,606 [1,161; 5,105] | 287 [116; 634] | 261 [105; 583] |
| Lower | 3,422 | 48% [33%; 58%] | 41 [16; 72] | 45 [17; 79] | 1,190 [469; 2,118] | 1,301 [510; 2,308] | 3 [1; 5] | 3 [1; 6] | 95 [43; 158] | 103 [47; 174] | 7 [3; 12] | 9 [3; 16] | 101 [48; 167] | 112 [53; 186] | 3.0 [1.4; 4.9] | 3.3 [1.5; 5.4] | 4 [2; 6] | 4 [2; 6] | 374 [145; 689] | 409 [158; 753] | 3,498 [1,591; 6,880] | 3,152 [1,415; 6,160] | 304 [124; 670] | 277 [112; 616] |
| Middle | 3,810 | 53% [37%; 64%] | 45 [18; 80] | 49 [19; 87] | 1,181 [465; 2,102] | 1,291 [507; 2,291] | 4 [1; 7] | 5 [2; 9] | 119 [54; 199] | 131 [59; 220] | 7 [3; 14] | 10 [4; 18] | 127 [59; 209] | 140 [65; 233] | 3.3 [1.6; 5.5] | 3.7 [1.7; 6.1] | 4 [2; 7] | 5 [2; 8] | 510 [198; 939] | 557 [216; 1,026] | 3,089 [1,395; 6,107] | 2,785 [1,240; 5,469] | 304 [123; 673] | 277 [111; 619] |
| Higher | 3,346 | 47% [33%; 56%] | 38 [15; 68] | 42 [16; 74] | 1,144 [451; 2,037] | 1,251 [491; 2,220] | 4 [1; 6] | 5 [2; 8] | 114 [51; 190] | 125 [56; 210] | 12 [5; 22] | 16 [6; 29] | 126 [60; 206] | 140 [67; 231] | 3.8 [1.8; 6.2] | 4.2 [2.0; 6.9] | 4 [2; 7] | 5 [2; 8] | 457 [177; 841] | 499 [193; 919] | 2,734 [1,244; 5,349] | 2,448 [1,096; 4,751] | 314 [126; 694] | 286 [114; 638] |
| Highest | 3,766 | 53% [37%; 63%] | 42 [17; 75] | 46 [18; 82] | 1,124 [443; 2,000] | 1,229 [482; 2,180] | 0 [0; 0] | 0 [0; 0] | 99 [44; 164] | 108 [49; 181] | 0 [0; 0] | 0 [0; 0] | 99 [44; 164] | 108 [49; 181] | 2.6 [1.2; 4.4] | 2.9 [1.3; 4.8] | 3 [2; 6] | 4 [2; 6] | 702 [273; 1,292] | 767 [297; 1,412] | 3,871 [1,717; 7,830] | 3,523 [1,529; 7,071] | 315 [124; 702] | 287 [112; 645] |
| Katanga | 13,321 | 25% [18%; 31%] | 171 [75; 332] | 187 [83; 363] | 1,283 [564; 2,489] | 1,402 [620; 2,722] | 29 [12; 57] | 38 [17; 74] | 729 [354; 1,320] | 798 [387; 1,444] | 179 [75; 359] | 233 [99; 468] | 908 [471; 1,584] | 1,031 [534; 1,817] | 6.8 [3.5; 11.9] | 7.7 [4.0; 13.6] | 32 [16; 55] | 36 [19; 63] | 1,072 [455; 2,172] | 1,172 [507; 2,364] | 1,538 [780; 3,277] | 1,354 [682; 2,879] | 284 [130; 693] | 259 [119; 631] |
| Lowest | 1,316 | 15% [11%; 19%] | 18 [8; 36] | 20 [9; 39] | 1,397 [615; 2,711] | 1,528 [675; 2,965] | 4 [2; 8] | 5 [2; 10] | 122 [59; 221] | 134 [65; 242] | 26 [11; 53] | 34 [15; 69] | 149 [77; 260] | 168 [87; 298] | 11.3 [5.8; 19.7] | 12.8 [6.6; 22.6] | 5 [3; 9] | 6 [3; 10] | 80 [34; 163] | 88 [38; 177] | 932 [473; 1,990] | 824 [415; 1,750] | 262 [120; 638] | 239 [110; 580] |
| Lower | 1,553 | 18% [13%; 22%] | 22 [10; 42] | 24 [10; 46] | 1,399 [615; 2,714] | 1,529 [676; 2,967] | 5 [2; 10] | 7 [3; 13] | 128 [62; 232] | 140 [68; 254] | 34 [14; 68] | 44 [19; 88] | 162 [84; 283] | 184 [96; 325] | 10.4 [5.4; 18.2] | 11.9 [6.2; 20.9] | 6 [3; 10] | 6 [3; 11] | 60 [26; 122] | 66 [28; 133] | 1,017 [519; 2,154] | 893 [454; 1,885] | 264 [122; 639] | 241 [112; 581] |
| Middle | 2,202 | 25% [18%; 31%] | 30 [13; 59] | 33 [15; 64] | 1,380 [607; 2,677] | 1,509 [667; 2,928] | 7 [3; 13] | 9 [4; 17] | 167 [81; 303] | 183 [89; 331] | 43 [18; 87] | 57 [24; 114] | 211 [110; 367] | 240 [124; 422] | 9.6 [5.0; 16.7] | 10.9 [5.6; 19.1] | 7 [4; 13] | 8 [4; 15] | 141 [60; 285] | 154 [67; 310] | 1,102 [561; 2,340] | 968 [490; 2,050] | 265 [122; 645] | 242 [111; 588] |
| Higher | 3,744 | 42% [31%; 53%] | 47 [21; 91] | 51 [23; 99] | 1,251 [551; 2,428] | 1,368 [605; 2,655] | 8 [3; 16] | 11 [5; 21] | 154 [75; 280] | 169 [82; 306] | 50 [21; 100] | 65 [28; 131] | 204 [107; 355] | 234 [122; 410] | 5.5 [2.9; 9.5] | 6.2 [3.3; 10.9] | 7 [4; 12] | 8 [4; 14] | 257 [109; 520] | 281 [121; 566] | 1,931 [986; 4,088] | 1,685 [848; 3,554] | 292 [134; 711] | 267 [122; 648] |
| Highest | 4,506 | 51% [37%; 63%] | 54 [24; 104] | 59 [26; 114] | 1,188 [523; 2,305] | 1,299 [574; 2,520] | 5 [2; 10] | 7 [3; 13] | 157 [76; 284] | 172 [83; 311] | 25 [11; 51] | 33 [14; 66] | 182 [93; 321] | 205 [105; 363] | 4.0 [2.1; 7.1] | 4.5 [2.3; 8.0] | 6 [3; 11] | 7 [4; 13] | 534 [227; 1,082] | 584 [253; 1,178] | 2,560 [1,278; 5,546] | 2,275 [1,134; 4,908] | 304 [137; 745] | 277 [125; 678] |
| Kasai-Oriental | 10,878 | 27% [20%; 34%] | 145 [63; 281] | 159 [70; 304] | 1,336 [582; 2,581] | 1,460 [641; 2,796] | 29 [12; 57] | 38 [16; 73] | 758 [372; 1,362] | 829 [406; 1,505] | 190 [80; 381] | 248 [104; 502] | 948 [490; 1,658] | 1,077 [561; 1,896] | 8.7 [4.5; 15.2] | 9.9 [5.2; 17.4] | 33 [17; 58] | 37 [19; 66] | 587 [250; 1,177] | 642 [273; 1,275] | 1,212 [615; 2,552] | 1,066 [537; 2,250] | 275 [127; 673] | 251 [116; 609] |
| Lowest | 1,506 | 20% [15%; 25%] | 21 [9; 41] | 23 [10; 44] | 1,397 [609; 2,700] | 1,527 [670; 2,925] | 5 [2; 10] | 7 [3; 13] | 160 [79; 288] | 175 [86; 318] | 39 [17; 78] | 51 [22; 103] | 200 [103; 349] | 227 [118; 399] | 13.3 [6.9; 23.2] | 15.1 [7.8; 26.5] | 7 [4; 12] | 8 [4; 14] | 59 [25; 117] | 64 [27; 127] | 800 [408; 1,683] | 704 [356; 1,482] | 264 [123; 645] | 241 [112; 583] |
| Lower | 1,659 | 23% [16%; 28%] | 23 [10; 44] | 25 [11; 48] | 1,384 [603; 2,675] | 1,514 [664; 2,898] | 5 [2; 10] | 6 [3; 12] | 145 [71; 261] | 159 [78; 288] | 35 [15; 69] | 45 [19; 91] | 180 [93; 314] | 204 [106; 359] | 10.8 [5.6; 18.9] | 12.3 [6.4; 21.6] | 6 [3; 11] | 7 [4; 12] | 28 [12; 55] | 30 [13; 60] | 985 [504; 2,071] | 868 [441; 1,827] | 268 [125; 652] | 245 [115; 591] |
| Middle | 1,845 | 25% [18%; 31%] | 26 [11; 49] | 28 [12; 53] | 1,383 [603; 2,672] | 1,512 [663; 2,894] | 5 [2; 10] | 6 [3; 12] | 132 [65; 238] | 145 [71; 263] | 30 [12; 59] | 39 [16; 78] | 162 [83; 283] | 183 [95; 323] | 8.8 [4.5; 15.3] | 9.9 [5.2; 17.5] | 6 [3; 10] | 6 [3; 11] | 76 [33; 153] | 84 [36; 166] | 1,207 [615; 2,550] | 1,066 [538; 2,253] | 266 [124; 651] | 243 [113; 589] |
| Higher | 2,787 | 38% [28%; 47%] | 37 [16; 71] | 40 [18; 77] | 1,325 [578; 2,560] | 1,449 [636; 2,774] | 7 [3; 14] | 10 [4; 19] | 168 [82; 302] | 184 [90; 333] | 47 [20; 94] | 62 [26; 124] | 215 [111; 375] | 245 [128; 431] | 7.7 [4.0; 13.5] | 8.8 [4.6; 15.5] | 7 [4; 13] | 9 [4; 15] | 128 [54; 256] | 140 [59; 277] | 1,372 [698; 2,880] | 1,204 [608; 2,529] | 278 [129; 679] | 254 [118; 614] |
| Highest | 3,080 | 42% [30%; 52%] | 39 [17; 75] | 42 [19; 81] | 1,262 [550; 2,438] | 1,379 [605; 2,641] | 6 [3; 13] | 8 [4; 17] | 152 [75; 274] | 167 [82; 302] | 40 [17; 79] | 52 [22; 105] | 192 [99; 335] | 218 [114; 384] | 6.2 [3.2; 10.9] | 7.1 [3.7; 12.5] | 7 [3; 12] | 8 [4; 13] | 297 [126; 595] | 324 [138; 644] | 1,677 [845; 3,548] | 1,472 [735; 3,123] | 288 [132; 709] | 262 [119; 642] |
| Kasai-Occidental | 8,825 | 31% [23%; 39%] | 121 [53; 233] | 133 [57; 252] | 1,375 [596; 2,640] | 1,503 [646; 2,853] | 27 [12; 53] | 36 [15; 70] | 650 [318; 1,189] | 711 [346; 1,294] | 208 [86; 412] | 272 [114; 536] | 859 [448; 1,510] | 983 [513; 1,710] | 9.7 [5.1; 17.1] | 11.1 [5.8; 19.4] | 30 [16; 52] | 34 [18; 59] | 341 [144; 683] | 373 [156; 740] | 1,092 [553; 2,286] | 953 [482; 2,016] | 268 [124; 660] | 245 [113; 598] |
| Lowest | 1,725 | 31% [22%; 38%] | 23 [10; 45] | 25 [11; 48] | 1,351 [585; 2,593] | 1,477 [635; 2,802] | 6 [2; 11] | 8 [3; 15] | 162 [79; 297] | 177 [86; 323] | 48 [20; 96] | 63 [27; 125] | 211 [110; 371] | 241 [126; 419] | 12.2 [6.4; 21.5] | 14.0 [7.3; 24.3] | 7 [4; 13] | 8 [4; 15] | 49 [21; 98] | 54 [22; 107] | 872 [444; 1,829] | 763 [386; 1,610] | 274 [127; 673] | 250 [116; 609] |
| Lower | 1,660 | 30% [22%; 37%] | 23 [10; 45] | 26 [11; 49] | 1,409 [610; 2,704] | 1,540 [662; 2,922] | 6 [2; 11] | 7 [3; 14] | 161 [79; 295] | 176 [86; 321] | 44 [18; 86] | 57 [24; 112] | 205 [106; 361] | 233 [121; 408] | 12.3 [6.4; 21.7] | 14.1 [7.3; 24.6] | 7 [4; 13] | 8 [4; 14] | 10 [4; 21] | 11 [5; 22] | 867 [444; 1,816] | 762 [387; 1,611] | 264 [123; 646] | 241 [113; 586] |
| Middle | 1,850 | 33% [24%; 41%] | 26 [11; 49] | 28 [12; 53] | 1,388 [601; 2,664] | 1,517 [652; 2,879] | 6 [2; 11] | 7 [3; 14] | 130 [63; 237] | 142 [69; 258] | 41 [17; 82] | 54 [23; 107] | 171 [89; 301] | 196 [102; 340] | 9.2 [4.8; 16.2] | 10.6 [5.5; 18.4] | 6 [3; 10] | 7 [4; 12] | 57 [24; 115] | 63 [26; 124] | 1,152 [584; 2,410] | 1,006 [509; 2,125] | 266 [123; 654] | 243 [113; 593] |
| Higher | 1,601 | 29% [21%; 35%] | 22 [10; 43] | 24 [10; 46] | 1,389 [602; 2,667] | 1,519 [653; 2,882] | 5 [2; 9] | 6 [3; 12] | 95 [46; 173] | 103 [50; 188] | 36 [15; 71] | 47 [20; 92] | 130 [68; 228] | 150 [78; 260] | 8.1 [4.2; 14.2] | 9.4 [4.9; 16.2] | 5 [2; 8] | 5 [3; 9] | 55 [23; 110] | 60 [25; 119] | 1,309 [663; 2,729] | 1,137 [576; 2,398] | 266 [123; 653] | 243 [112; 592] |
| Highest | 1,988 | 35% [26%; 44%] | 27 [12; 51] | 29 [13; 55] | 1,345 [583; 2,582] | 1,470 [632; 2,790] | 5 [2; 11] | 7 [3; 14] | 103 [50; 187] | 112 [55; 204] | 39 [16; 78] | 51 [21; 101] | 142 [74; 248] | 163 [85; 283] | 7.1 [3.7; 12.5] | 8.2 [4.3; 14.2] | 5 [3; 9] | 6 [3; 10] | 170 [71; 340] | 185 [78; 368] | 1,471 [740; 3,086] | 1,275 [638; 2,711] | 271 [123; 671] | 247 [112; 608] |
| Equateur | 7,822 | 22% [16%; 27%] | 107 [47; 205] | 117 [51; 224] | 1,365 [602; 2,625] | 1,492 [657; 2,859] | 18 [8; 35] | 24 [10; 46] | 571 [274; 1,041] | 624 [301; 1,149] | 128 [53; 257] | 167 [71; 338] | 699 [359; 1,229] | 791 [409; 1,401] | 8.9 [4.6; 15.7] | 10.1 [5.2; 17.9] | 24 [12; 43] | 27 [14; 49] | 397 [170; 801] | 434 [186; 865] | 1,183 [594; 2,541] | 1,044 [529; 2,237] | 269 [126; 660] | 246 [116; 594] |
| Lowest | 1,414 | 20% [15%; 25%] | 20 [9; 38] | 21 [9; 41] | 1,390 [613; 2,673] | 1,520 [669; 2,911] | 4 [2; 8] | 5 [2; 10] | 117 [56; 213] | 128 [62; 235] | 29 [12; 58] | 38 [16; 76] | 146 [75; 256] | 165 [86; 292] | 10.3 [5.3; 18.1] | 11.7 [6.1; 20.7] | 5 [3; 9] | 6 [3; 10] | 54 [23; 109] | 59 [25; 117] | 1,030 [519; 2,200] | 907 [461; 1,935] | 265 [125; 649] | 242 [114; 584] |
| Lower | 1,389 | 20% [15%; 25%] | 19 [9; 37] | 21 [9; 41] | 1,394 [615; 2,681] | 1,524 [671; 2,920] | 4 [2; 8] | 5 [2; 10] | 120 [57; 218] | 131 [63; 241] | 31 [13; 61] | 40 [17; 81] | 150 [78; 264] | 171 [89; 302] | 10.8 [5.6; 19.0] | 12.3 [6.4; 21.8] | 5 [3; 9] | 6 [3; 10] | 22 [9; 44] | 24 [10; 48] | 987 [500; 2,101] | 868 [444; 1,845] | 266 [126; 649] | 243 [116; 584] |
| Middle | 1,310 | 19% [14%; 23%] | 18 [8; 35] | 20 [9; 38] | 1,377 [608; 2,649] | 1,506 [663; 2,885] | 3 [1; 6] | 4 [2; 8] | 114 [55; 208] | 125 [60; 229] | 21 [9; 42] | 28 [12; 56] | 135 [69; 239] | 152 [78; 271] | 10.3 [5.3; 18.2] | 11.6 [6.0; 20.7] | 5 [2; 8] | 5 [3; 9] | 53 [23; 107] | 58 [25; 116] | 1,027 [516; 2,211] | 911 [463; 1,963] | 268 [126; 655] | 244 [115; 589] |
| Higher | 1,611 | 23% [17%; 29%] | 22 [10; 42] | 24 [11; 46] | 1,365 [603; 2,626] | 1,493 [657; 2,860] | 4 [2; 7] | 5 [2; 10] | 107 [51; 194] | 117 [56; 214] | 28 [12; 57] | 37 [16; 75] | 135 [70; 237] | 154 [80; 271] | 8.4 [4.3; 14.7] | 9.5 [5.0; 16.9] | 5 [2; 8] | 5 [3; 9] | 70 [30; 141] | 76 [33; 152] | 1,265 [638; 2,704] | 1,111 [564; 2,365] | 270 [127; 660] | 246 [116; 594] |
| Highest | 2,098 | 30% [22%; 37%] | 28 [12; 53] | 30 [13; 58] | 1,321 [583; 2,540] | 1,444 [636; 2,766] | 3 [1; 6] | 4 [2; 7] | 114 [55; 207] | 124 [60; 229] | 19 [8; 38] | 25 [10; 50] | 133 [68; 235] | 149 [76; 265] | 6.3 [3.2; 11.2] | 7.1 [3.6; 12.6] | 5 [2; 8] | 5 [3; 9] | 198 [85; 400] | 216 [93; 432] | 1,649 [819; 3,573] | 1,465 [736; 3,184] | 275 [127; 679] | 251 [116; 610] |
| Bas-Congo | 6,504 | 46% [33%; 57%] | 85 [37; 164] | 93 [41; 180] | 1,306 [567; 2,515] | 1,428 [625; 2,773] | 18 [7; 34] | 23 [10; 45] | 349 [171; 621] | 382 [185; 689] | 111 [47; 220] | 145 [62; 286] | 460 [241; 790] | 527 [276; 905] | 7.1 [3.7; 12.1] | 8.1 [4.2; 13.9] | 16 [8; 27] | 18 [10; 31] | 357 [151; 717] | 391 [169; 780] | 1,495 [774; 3,139] | 1,305 [673; 2,712] | 281 [131; 688] | 257 [119; 620] |
| Lowest | 1,125 | 40% [29%; 50%] | 16 [7; 30] | 17 [7; 33] | 1,382 [600; 2,660] | 1,511 [661; 2,933] | 4 [2; 8] | 5 [2; 10] | 82 [40; 146] | 89 [43; 162] | 27 [12; 54] | 36 [15; 71] | 109 [57; 187] | 125 [66; 215] | 9.7 [5.1; 16.7] | 11.1 [5.8; 19.1] | 4 [2; 7] | 4 [2; 7] | 51 [22; 102] | 56 [24; 111] | 1,092 [567; 2,289] | 951 [492; 1,965] | 266 [124; 651] | 243 [113; 587] |
| Lower | 1,258 | 45% [33%; 56%] | 17 [7; 33] | 18 [8; 36] | 1,345 [584; 2,589] | 1,470 [643; 2,854] | 4 [2; 8] | 5 [2; 10] | 69 [34; 123] | 76 [37; 137] | 25 [11; 50] | 33 [14; 65] | 94 [50; 162] | 108 [57; 186] | 7.5 [3.9; 12.9] | 8.6 [4.5; 14.8] | 3 [2; 6] | 4 [2; 6] | 29 [12; 58] | 32 [14; 63] | 1,426 [743; 2,981] | 1,240 [644; 2,556] | 275 [130; 671] | 252 [118; 605] |
| Middle | 1,340 | 48% [35%; 59%] | 18 [8; 35] | 20 [9; 38] | 1,341 [582; 2,582] | 1,466 [641; 2,846] | 4 [2; 8] | 6 [2; 11] | 89 [43; 158] | 97 [47; 175] | 28 [12; 56] | 37 [16; 73] | 117 [61; 201] | 134 [70; 230] | 8.7 [4.6; 15.0] | 10.0 [5.2; 17.2] | 4 [2; 7] | 5 [2; 8] | 64 [27; 128] | 70 [30; 139] | 1,214 [629; 2,546] | 1,059 [547; 2,196] | 274 [128; 671] | 251 [116; 605] |
| Higher | 1,383 | 50% [36%; 61%] | 18 [8; 34] | 19 [8; 38] | 1,284 [558; 2,472] | 1,404 [614; 2,725] | 3 [1; 6] | 4 [2; 8] | 58 [29; 104] | 64 [31; 115] | 18 [7; 35] | 23 [10; 46] | 76 [40; 131] | 87 [46; 150] | 5.5 [2.9; 9.5] | 6.3 [3.3; 10.8] | 3 [1; 5] | 3 [2; 5] | 70 [30; 141] | 77 [33; 153] | 1,920 [993; 4,040] | 1,680 [866; 3,489] | 286 [133; 701] | 261 [121; 632] |
| Highest | 1,398 | 50% [37%; 62%] | 17 [7; 32] | 18 [8; 36] | 1,200 [521; 2,311] | 1,312 [574; 2,547] | 2 [1; 5] | 3 [1; 6] | 51 [25; 91] | 56 [27; 101] | 12 [5; 24] | 16 [7; 31] | 63 [33; 109] | 72 [37; 124] | 4.5 [2.4; 7.8] | 5.1 [2.7; 8.9] | 2 [1; 4] | 2 [1; 4] | 143 [61; 288] | 157 [68; 313] | 2,304 [1,175; 4,917] | 2,029 [1,033; 4,268] | 302 [139; 746] | 275 [126; 671] |
| Bandundu | 13,757 | 36% [26%; 45%] | 184 [78; 352] | 201 [86; 388] | 1,336 [568; 2,556] | 1,460 [628; 2,819] | 33 [14; 64] | 43 [18; 84] | 823 [398; 1,469] | 900 [432; 1,617] | 168 [68; 338] | 219 [89; 442] | 991 [508; 1,728] | 1,119 [567; 1,963] | 7.2 [3.7; 12.6] | 8.1 [4.1; 14.3] | 34 [18; 60] | 39 [20; 68] | 630 [262; 1,262] | 689 [291; 1,378] | 1,469 [749; 3,152] | 1,300 [661; 2,793] | 276 [130; 693] | 252 [117; 629] |
| Lowest | 2,598 | 35% [25%; 43%] | 36 [15; 69] | 39 [17; 76] | 1,388 [590; 2,656] | 1,517 [653; 2,929] | 8 [3; 15] | 10 [4; 19] | 194 [94; 347] | 213 [102; 382] | 41 [17; 83] | 54 [22; 109] | 236 [121; 411] | 267 [135; 468] | 9.1 [4.7; 15.8] | 10.3 [5.2; 18.0] | 8 [4; 14] | 9 [5; 16] | 90 [37; 180] | 98 [42; 197] | 1,170 [598; 2,503] | 1,034 [527; 2,216] | 266 [126; 668] | 243 [114; 606] |
| Lower | 2,273 | 30% [22%; 38%] | 31 [13; 60] | 34 [15; 66] | 1,372 [583; 2,626] | 1,500 [646; 2,896] | 6 [3; 12] | 8 [4; 16] | 153 [74; 273] | 167 [80; 300] | 33 [13; 66] | 43 [18; 87] | 186 [95; 324] | 210 [107; 369] | 8.2 [4.2; 14.2] | 9.2 [4.7; 16.2] | 6 [3; 11] | 7 [4; 13] | 28 [12; 56] | 31 [13; 61] | 1,307 [670; 2,789] | 1,156 [591; 2,470] | 271 [129; 677] | 247 [117; 614] |
| Middle | 2,834 | 38% [28%; 47%] | 38 [16; 73] | 42 [18; 81] | 1,352 [575; 2,587] | 1,478 [636; 2,853] | 8 [3; 15] | 10 [4; 20] | 205 [99; 366] | 224 [108; 403] | 41 [17; 82] | 53 [22; 107] | 246 [126; 429] | 277 [140; 487] | 8.7 [4.4; 15.1] | 9.8 [5.0; 17.2] | 9 [4; 15] | 10 [5; 17] | 105 [44; 210] | 115 [49; 230] | 1,223 [624; 2,626] | 1,083 [551; 2,323] | 273 [129; 686] | 249 [116; 622] |
| Higher | 2,798 | 37% [27%; 46%] | 37 [16; 71] | 40 [17; 78] | 1,322 [562; 2,530] | 1,445 [622; 2,790] | 6 [2; 11] | 7 [3; 14] | 128 [62; 228] | 140 [67; 251] | 25 [10; 51] | 33 [13; 67] | 153 [78; 267] | 173 [87; 303] | 5.5 [2.8; 9.5] | 6.2 [3.1; 10.8] | 5 [3; 9] | 6 [3; 11] | 112 [46; 223] | 122 [52; 244] | 1,938 [989; 4,164] | 1,717 [872; 3,687] | 279 [131; 701] | 255 [119; 636] |
| Highest | 3,253 | 44% [32%; 54%] | 41 [18; 79] | 45 [19; 87] | 1,266 [538; 2,424] | 1,384 [596; 2,673] | 6 [2; 11] | 7 [3; 14] | 143 [69; 256] | 157 [75; 281] | 27 [11; 55] | 36 [15; 72] | 171 [87; 298] | 192 [97; 338] | 5.2 [2.7; 9.2] | 5.9 [3.0; 10.4] | 6 [3; 10] | 7 [3; 12] | 295 [123; 592] | 323 [137; 646] | 1,993 [1,010; 4,306] | 1,766 [890; 3,817] | 287 [133; 727] | 262 [120; 659] |
| KENYA |  |  |  |  |  |  |  |  |  |  |  |  |  |  |  |  |  |  |  |  |  |  |  |  |
| Western | 10,278 | 50% [36%; 61%] | 159 [69; 307] | 174 [76; 332] | 1,548 [672; 2,988] | 1,692 [741; 3,235] | 18 [8; 36] | 24 [10; 47] | 237 [152; 348] | 259 [163; 378] | 30 [13; 59] | 39 [17; 76] | 266 [171; 390] | 298 [188; 436] | 2.6 [1.7; 3.8] | 2.9 [1.8; 4.2] | 9 [6; 14] | 11 [7; 15] | 869 [346; 1,846] | 950 [376; 2,002] | 3,952 [2,304; 6,956] | 3,534 [2,050; 6,267] | 235 [109; 582] | 215 [99; 535] |
| Lowest | 1,974 | 48% [35%; 59%] | 32 [14; 61] | 35 [15; 66] | 1,607 [698; 3,102] | 1,757 [769; 3,359] | 5 [2; 10] | 7 [3; 13] | 59 [38; 87] | 65 [41; 95] | 9 [4; 18] | 12 [5; 23] | 69 [44; 101] | 77 [49; 113] | 3.5 [2.2; 5.1] | 3.9 [2.5; 5.7] | 2 [2; 4] | 3 [2; 4] | 104 [41; 221] | 114 [45; 239] | 2,972 [1,742; 5,213] | 2,649 [1,549; 4,688] | 228 [107; 563] | 209 [98; 517] |
| Lower | 1,979 | 48% [35%; 59%] | 31 [14; 61] | 34 [15; 66] | 1,591 [691; 3,070] | 1,739 [761; 3,324] | 4 [2; 8] | 5 [2; 10] | 48 [31; 71] | 53 [33; 77] | 6 [3; 12] | 8 [4; 16] | 54 [35; 80] | 61 [39; 89] | 2.7 [1.8; 4.0] | 3.1 [1.9; 4.5] | 2 [1; 3] | 2 [1; 3] | 110 [44; 233] | 120 [47; 253] | 3,758 [2,200; 6,596] | 3,362 [1,964; 5,937] | 231 [107; 568] | 211 [98; 523] |
| Middle | 2,059 | 50% [36%; 62%] | 32 [14; 62] | 35 [15; 67] | 1,561 [678; 3,013] | 1,707 [747; 3,262] | 3 [1; 7] | 4 [2; 9] | 42 [27; 61] | 46 [29; 67] | 5 [2; 11] | 7 [3; 13] | 47 [30; 69] | 52 [33; 77] | 2.3 [1.5; 3.3] | 2.5 [1.6; 3.7] | 2 [1; 2] | 2 [1; 3] | 95 [38; 202] | 104 [41; 219] | 4,539 [2,665; 7,951] | 4,063 [2,379; 7,171] | 236 [110; 580] | 215 [101; 533] |
| Higher | 2,090 | 51% [37%; 63%] | 33 [14; 63] | 36 [16; 68] | 1,566 [680; 3,023] | 1,712 [750; 3,273] | 4 [2; 7] | 5 [2; 9] | 47 [30; 69] | 52 [33; 75] | 6 [2; 11] | 7 [3; 14] | 53 [34; 77] | 59 [37; 86] | 2.5 [1.6; 3.7] | 2.8 [1.8; 4.1] | 2 [1; 3] | 2 [1; 3] | 177 [71; 376] | 194 [77; 408] | 4,057 [2,365; 7,142] | 3,633 [2,109; 6,438] | 232 [107; 576] | 212 [98; 529] |
| Highest | 2,175 | 53% [38%; 65%] | 31 [13; 60] | 34 [15; 65] | 1,425 [619; 2,750] | 1,558 [682; 2,978] | 2 [1; 4] | 3 [1; 6] | 40 [26; 59] | 44 [28; 64] | 4 [2; 7] | 5 [2; 9] | 44 [28; 64] | 48 [31; 71] | 2.0 [1.3; 2.9] | 2.2 [1.4; 3.3] | 2 [1; 2] | 2 [1; 3] | 383 [153; 813] | 418 [166; 882] | 4,975 [2,852; 8,865] | 4,465 [2,533; 7,984] | 249 [111; 627] | 227 [101; 576] |
| Rift Valley | 20,565 | 48% [35%; 59%] | 309 [135; 589] | 338 [148; 649] | 1,501 [655; 2,863] | 1,641 [717; 3,154] | 41 [18; 80] | 54 [23; 105] | 537 [341; 786] | 587 [377; 856] | 61 [26; 119] | 80 [34; 155] | 598 [381; 874] | 667 [428; 972] | 2.9 [1.9; 4.3] | 3.2 [2.1; 4.7] | 21 [14; 31] | 24 [15; 35] | 3,082 [1,223; 6,488] | 3,369 [1,337; 7,153] | 3,457 [2,006; 6,135] | 3,092 [1,784; 5,546] | 238 [106; 598] | 217 [97; 542] |
| Lowest | 3,630 | 43% [31%; 53%] | 60 [26; 115] | 66 [29; 126] | 1,655 [722; 3,155] | 1,809 [791; 3,476] | 10 [4; 19] | 13 [5; 24] | 164 [104; 240] | 179 [115; 262] | 15 [7; 30] | 20 [9; 39] | 179 [114; 263] | 199 [128; 290] | 4.9 [3.1; 7.2] | 5.5 [3.5; 8.0] | 6 [4; 9] | 7 [5; 10] | 405 [161; 853] | 443 [176; 940] | 2,055 [1,204; 3,620] | 1,845 [1,080; 3,288] | 218 [99; 544] | 199 [90; 494] |
| Lower | 4,154 | 49% [36%; 61%] | 67 [29; 127] | 73 [32; 140] | 1,607 [701; 3,063] | 1,757 [768; 3,375] | 10 [4; 20] | 13 [6; 26] | 121 [77; 177] | 132 [85; 193] | 15 [7; 30] | 20 [9; 39] | 136 [87; 199] | 152 [97; 222] | 3.3 [2.1; 4.8] | 3.7 [2.3; 5.3] | 5 [3; 7] | 5 [3; 8] | 473 [188; 995] | 517 [205; 1,097] | 3,097 [1,809; 5,475] | 2,767 [1,613; 4,940] | 225 [102; 561] | 205 [92; 509] |
| Middle | 4,218 | 50% [36%; 62%] | 66 [29; 125] | 72 [31; 138] | 1,560 [681; 2,975] | 1,706 [745; 3,278] | 9 [4; 18] | 12 [5; 23] | 100 [64; 147] | 109 [70; 160] | 13 [6; 26] | 17 [7; 33] | 113 [72; 166] | 127 [81; 185] | 2.7 [1.7; 3.9] | 3.0 [1.9; 4.4] | 4 [3; 6] | 4 [3; 7] | 454 [180; 955] | 496 [197; 1,053] | 3,787 [2,214; 6,690] | 3,382 [1,973; 6,036] | 232 [105; 578] | 212 [96; 524] |
| Higher | 4,246 | 50% [36%; 62%] | 62 [27; 118] | 67 [29; 130] | 1,453 [634; 2,770] | 1,588 [694; 3,051] | 7 [3; 14] | 10 [4; 19] | 83 [53; 122] | 91 [58; 133] | 11 [4; 20] | 14 [6; 27] | 94 [60; 137] | 105 [67; 153] | 2.2 [1.4; 3.2] | 2.5 [1.6; 3.6] | 3 [2; 5] | 4 [2; 5] | 660 [262; 1,389] | 722 [286; 1,532] | 4,545 [2,638; 8,062] | 4,058 [2,333; 7,281] | 246 [110; 618] | 224 [100; 560] |
| Highest | 4,317 | 51% [37%; 63%] | 54 [24; 104] | 60 [26; 114] | 1,262 [551; 2,405] | 1,379 [603; 2,650] | 5 [2; 10] | 7 [3; 13] | 68 [44; 100] | 75 [48; 109] | 7 [3; 13] | 9 [4; 17] | 75 [48; 110] | 84 [54; 122] | 1.7 [1.1; 2.5] | 1.9 [1.2; 2.8] | 3 [2; 4] | 3 [2; 4] | 1,090 [433; 2,296] | 1,192 [473; 2,531] | 5,602 [3,178; 10,044] | 5,008 [2,810; 9,107] | 275 [119; 705] | 250 [107; 636] |
| Nyanza | 13,707 | 49% [36%; 61%] | 210 [92; 399] | 230 [102; 443] | 1,535 [674; 2,912] | 1,678 [743; 3,230] | 23 [10; 44] | 30 [13; 58] | 303 [195; 443] | 331 [211; 482] | 45 [20; 88] | 59 [26; 114] | 348 [225; 508] | 390 [250; 568] | 2.5 [1.6; 3.7] | 2.8 [1.8; 4.1] | 12 [8; 18] | 14 [9; 20] | 1,292 [516; 2,726] | 1,412 [569; 2,956] | 4,026 [2,341; 7,073] | 3,588 [2,070; 6,382] | 237 [110; 582] | 216 [99; 531] |
| Lowest | 2,644 | 48% [35%; 59%] | 43 [19; 81] | 46 [21; 90] | 1,609 [707; 3,053] | 1,759 [779; 3,386] | 6 [3; 11] | 8 [3; 15] | 78 [50; 114] | 85 [54; 124] | 12 [5; 24] | 16 [7; 31] | 90 [58; 132] | 101 [65; 148] | 3.4 [2.2; 5.0] | 3.8 [2.5; 5.6] | 3 [2; 5] | 4 [2; 5] | 155 [62; 326] | 169 [68; 354] | 3,024 [1,773; 5,294] | 2,694 [1,565; 4,778] | 228 [107; 558] | 208 [97; 509] |
| Lower | 2,747 | 50% [36%; 61%] | 44 [19; 83] | 48 [21; 92] | 1,593 [700; 3,023] | 1,742 [771; 3,353] | 6 [3; 12] | 8 [3; 15] | 68 [44; 100] | 75 [48; 109] | 13 [5; 25] | 17 [7; 32] | 81 [52; 118] | 91 [58; 133] | 2.9 [1.9; 4.3] | 3.3 [2.1; 4.8] | 3 [2; 4] | 3 [2; 5] | 171 [68; 360] | 187 [75; 391] | 3,508 [2,054; 6,142] | 3,113 [1,811; 5,532] | 230 [108; 563] | 210 [98; 513] |
| Middle | 2,781 | 50% [37%; 62%] | 44 [19; 83] | 48 [21; 92] | 1,571 [690; 2,981] | 1,718 [761; 3,307] | 4 [2; 8] | 5 [2; 10] | 52 [34; 76] | 57 [36; 83] | 7 [3; 14] | 9 [4; 18] | 59 [38; 87] | 66 [43; 97] | 2.1 [1.4; 3.1] | 2.4 [1.5; 3.5] | 2 [1; 3] | 2 [2; 3] | 156 [62; 329] | 170 [69; 357] | 4,840 [2,837; 8,474] | 4,327 [2,517; 7,650] | 234 [110; 571] | 213 [99; 521] |
| Higher | 2,674 | 48% [35%; 60%] | 41 [18; 78] | 45 [20; 87] | 1,539 [676; 2,920] | 1,682 [745; 3,239] | 4 [2; 8] | 5 [2; 11] | 52 [34; 77] | 57 [37; 83] | 9 [4; 17] | 11 [5; 22] | 61 [39; 89] | 69 [44; 100] | 2.3 [1.5; 3.3] | 2.6 [1.6; 3.7] | 2 [1; 3] | 2 [2; 4] | 261 [104; 551] | 286 [115; 598] | 4,479 [2,601; 7,865] | 3,983 [2,304; 7,102] | 236 [109; 581] | 215 [99; 529] |
| Highest | 2,861 | 52% [38%; 64%] | 39 [17; 74] | 43 [19; 83] | 1,371 [603; 2,602] | 1,499 [664; 2,887] | 3 [1; 5] | 4 [2; 7] | 52 [33; 76] | 57 [36; 83] | 4 [2; 9] | 6 [3; 11] | 57 [36; 83] | 63 [40; 91] | 2.0 [1.3; 2.9] | 2.2 [1.4; 3.2] | 2 [1; 3] | 2 [1; 3] | 549 [219; 1,159] | 600 [242; 1,257] | 5,019 [2,861; 8,894] | 4,503 [2,547; 8,067] | 258 [116; 645] | 234 [104; 587] |
| North Eastern | 3,433 | 37% [27%; 46%] | 53 [23; 102] | 58 [26; 114] | 1,552 [683; 2,980] | 1,697 [749; 3,312] | 7 [3; 13] | 9 [4; 17] | 90 [58; 132] | 98 [63; 145] | 11 [5; 22] | 14 [6; 29] | 101 [65; 149] | 113 [73; 166] | 2.9 [1.9; 4.3] | 3.3 [2.1; 4.8] | 4 [2; 5] | 4 [3; 6] | 478 [191; 1,022] | 523 [211; 1,121] | 3,429 [1,931; 6,111] | 3,063 [1,723; 5,369] | 231 [103; 572] | 211 [92; 526] |
| Lowest | 550 | 31% [22%; 38%] | 9 [4; 18] | 10 [4; 20] | 1,663 [732; 3,194] | 1,818 [803; 3,549] | 1 [1; 3] | 2 [1; 4] | 19 [12; 28] | 21 [13; 30] | 2 [1; 5] | 3 [1; 6] | 21 [14; 31] | 24 [15; 35] | 3.9 [2.5; 5.7] | 4.3 [2.8; 6.3] | 1 [0; 1] | 1 [1; 1] | 50 [20; 108] | 55 [22; 118] | 2,654 [1,514; 4,690] | 2,374 [1,353; 4,132] | 218 [99; 536] | 199 [89; 493] |
| Lower | 681 | 38% [28%; 47%] | 11 [5; 22] | 12 [5; 24] | 1,664 [732; 3,196] | 1,819 [803; 3,551] | 2 [1; 3] | 2 [1; 4] | 24 [15; 35] | 26 [16; 38] | 3 [1; 5] | 4 [2; 7] | 26 [17; 39] | 29 [19; 43] | 3.9 [2.5; 5.7] | 4.3 [2.8; 6.4] | 1 [1; 1] | 1 [1; 2] | 65 [26; 139] | 71 [29; 153] | 2,636 [1,502; 4,664] | 2,361 [1,345; 4,114] | 218 [98; 536] | 199 [89; 492] |
| Middle | 672 | 38% [27%; 47%] | 11 [5; 21] | 12 [5; 23] | 1,617 [712; 3,106] | 1,768 [781; 3,452] | 1 [1; 3] | 2 [1; 4] | 18 [12; 27] | 20 [13; 30] | 3 [1; 5] | 3 [1; 7] | 21 [13; 31] | 24 [15; 35] | 3.1 [2.0; 4.6] | 3.5 [2.3; 5.2] | 1 [0; 1] | 1 [1; 1] | 59 [24; 126] | 64 [26; 138] | 3,267 [1,866; 5,776] | 2,915 [1,659; 5,083] | 225 [102; 552] | 205 [92; 507] |
| Higher | 661 | 37% [27%; 46%] | 10 [5; 20] | 11 [5; 22] | 1,559 [686; 2,995] | 1,705 [753; 3,328] | 1 [1; 2] | 2 [1; 3] | 15 [10; 22] | 17 [11; 24] | 2 [1; 4] | 3 [1; 6] | 17 [11; 25] | 19 [12; 29] | 2.6 [1.7; 3.9] | 2.9 [1.9; 4.3] | 1 [0; 1] | 1 [0; 1] | 88 [35; 188] | 96 [39; 206] | 3,864 [2,177; 6,876] | 3,445 [1,944; 6,041] | 230 [103; 570] | 210 [92; 523] |
| Highest | 868 | 49% [35%; 60%] | 12 [5; 22] | 13 [6; 25] | 1,337 [588; 2,567] | 1,461 [645; 2,853] | 1 [0; 2] | 1 [1; 2] | 14 [9; 20] | 15 [10; 22] | 1 [1; 3] | 2 [1; 3] | 15 [10; 22] | 17 [11; 25] | 1.7 [1.1; 2.5] | 1.9 [1.2; 2.8] | 1 [0; 1] | 1 [0; 1] | 216 [86; 461] | 236 [95; 506] | 5,632 [3,091; 10,134] | 5,037 [2,752; 8,960] | 260 [110; 656] | 236 [99; 602] |
| Nairobi | 5,827 | 51% [36%; 62%] | 74 [30; 129] | 81 [33; 140] | 1,274 [520; 2,213] | 1,393 [565; 2,404] | 6 [2; 10] | 7 [3; 13] | 78 [48; 109] | 85 [52; 118] | 8 [3; 14] | 11 [4; 19] | 86 [53; 119] | 96 [59; 132] | 1.5 [0.9; 2.0] | 1.6 [1.0; 2.3] | 3 [2; 4] | 3 [2; 5] | 1,193 [436; 2,240] | 1,304 [475; 2,424] | 6,704 [3,594; 11,023] | 5,995 [3,247; 9,893] | 276 [111; 628] | 251 [101; 570] |
| Lowest | 1,218 | 53% [37%; 64%] | 18 [7; 31] | 20 [8; 34] | 1,473 [601; 2,558] | 1,610 [653; 2,779] | 3 [1; 5] | 4 [1; 6] | 21 [13; 30] | 23 [14; 32] | 5 [2; 8] | 6 [2; 11] | 26 [16; 36] | 29 [18; 41] | 2.1 [1.3; 3.0] | 2.4 [1.5; 3.4] | 1 [1; 1] | 1 [1; 1] | 220 [80; 412] | 240 [88; 446] | 4,680 [2,514; 7,731] | 4,123 [2,222; 6,850] | 241 [98; 545] | 219 [89; 494] |
| Lower | 1,126 | 49% [34%; 59%] | 16 [6; 27] | 17 [7; 30] | 1,404 [573; 2,438] | 1,535 [622; 2,649] | 1 [0; 2] | 1 [0; 2] | 16 [9; 22] | 17 [10; 24] | 1 [0; 1] | 1 [0; 1] | 16 [10; 22] | 18 [11; 24] | 1.4 [0.9; 2.0] | 1.6 [1.0; 2.2] | 1 [0; 1] | 1 [0; 1] | 203 [74; 381] | 222 [81; 412] | 6,978 [3,764; 11,442] | 6,315 [3,458; 10,403] | 253 [103; 572] | 230 [93; 519] |
| Middle | 1,148 | 50% [35%; 61%] | 16 [6; 27] | 17 [7; 29] | 1,356 [553; 2,355] | 1,482 [601; 2,558] | 1 [1; 2] | 2 [1; 3] | 18 [11; 25] | 20 [12; 28] | 2 [1; 4] | 3 [1; 5] | 20 [13; 28] | 23 [14; 31] | 1.8 [1.1; 2.5] | 2.0 [1.2; 2.7] | 1 [0; 1] | 1 [0; 1] | 195 [71; 367] | 213 [78; 397] | 5,642 [3,059; 9,247] | 5,041 [2,763; 8,292] | 262 [107; 593] | 239 [97; 538] |
| Higher | 1,138 | 50% [35%; 60%] | 14 [6; 24] | 15 [6; 26] | 1,201 [490; 2,086] | 1,313 [532; 2,266] | 1 [0; 1] | 1 [0; 2] | 14 [8; 19] | 15 [9; 21] | 1 [0; 1] | 1 [0; 2] | 15 [9; 20] | 16 [10; 22] | 1.3 [0.8; 1.8] | 1.4 [0.9; 2.0] | 1 [0; 1] | 1 [0; 1] | 236 [86; 443] | 258 [94; 479] | 7,717 [4,143; 12,674] | 6,952 [3,776; 11,448] | 293 [118; 666] | 266 [107; 604] |
| Highest | 1,197 | 52% [37%; 63%] | 11 [5; 20] | 12 [5; 21] | 941 [384; 1,634] | 1,029 [417; 1,775] | 0 [0; 0] | 0 [0; 0] | 9 [6; 13] | 10 [6; 14] | 0 [0; 0] | 0 [0; 0] | 9 [6; 13] | 10 [6; 14] | 0.8 [0.5; 1.1] | 0.8 [0.5; 1.1] | 0 [0; 0] | 0 [0; 0] | 339 [124; 637] | 371 [135; 690] | 12,748 [6,723; 21,103] | 11,570 [6,139; 19,295] | 366 [142; 842] | 332 [127; 763] |
| Eastern | 11,907 | 53% [39%; 65%] | 181 [80; 344] | 198 [87; 377] | 1,522 [673; 2,891] | 1,664 [728; 3,166] | 23 [10; 44] | 30 [13; 59] | 328 [209; 481] | 358 [228; 520] | 34 [15; 65] | 44 [19; 86] | 361 [231; 527] | 402 [257; 585] | 3.0 [1.9; 4.4] | 3.4 [2.2; 4.9] | 13 [8; 19] | 14 [9; 21] | 1,848 [736; 3,870] | 2,021 [808; 4,297] | 3,306 [1,919; 5,975] | 2,961 [1,706; 5,391] | 235 [107; 587] | 214 [96; 541] |
| Lowest | 2,206 | 49% [36%; 61%] | 37 [16; 70] | 40 [18; 76] | 1,663 [735; 3,159] | 1,818 [795; 3,460] | 6 [3; 12] | 8 [3; 15] | 95 [61; 140] | 104 [66; 151] | 10 [4; 19] | 13 [5; 25] | 105 [67; 153] | 117 [75; 170] | 4.8 [3.0; 6.9] | 5.3 [3.4; 7.7] | 4 [2; 5] | 4 [3; 6] | 261 [104; 546] | 285 [114; 606] | 2,131 [1,244; 3,835] | 1,911 [1,112; 3,466] | 217 [100; 539] | 198 [91; 497] |
| Lower | 2,308 | 52% [38%; 64%] | 37 [16; 70] | 40 [18; 76] | 1,593 [704; 3,025] | 1,742 [762; 3,314] | 5 [2; 10] | 7 [3; 14] | 64 [41; 93] | 70 [44; 101] | 8 [3; 15] | 10 [4; 20] | 71 [46; 104] | 80 [51; 116] | 3.1 [2.0; 4.5] | 3.4 [2.2; 5.0] | 3 [2; 4] | 3 [2; 4] | 284 [113; 595] | 310 [124; 660] | 3,281 [1,914; 5,911] | 2,933 [1,702; 5,330] | 226 [104; 563] | 206 [94; 519] |
| Middle | 2,444 | 55% [40%; 67%] | 38 [17; 73] | 42 [18; 80] | 1,574 [695; 2,988] | 1,721 [753; 3,274] | 5 [2; 9] | 6 [3; 12] | 61 [39; 90] | 67 [43; 98] | 6 [3; 12] | 8 [3; 16] | 68 [43; 99] | 75 [48; 109] | 2.8 [1.8; 4.0] | 3.1 [2.0; 4.5] | 2 [2; 4] | 3 [2; 4] | 278 [111; 583] | 304 [122; 647] | 3,674 [2,148; 6,616] | 3,297 [1,921; 5,978] | 229 [106; 570] | 209 [96; 526] |
| Higher | 2,465 | 55% [40%; 68%] | 36 [16; 68] | 39 [17; 75] | 1,463 [647; 2,779] | 1,600 [700; 3,044] | 4 [2; 8] | 5 [2; 10] | 58 [37; 85] | 64 [40; 92] | 6 [2; 11] | 7 [3; 14] | 64 [41; 93] | 71 [45; 103] | 2.6 [1.7; 3.8] | 2.9 [1.8; 4.2] | 2 [1; 3] | 3 [2; 4] | 394 [157; 826] | 431 [172; 917] | 3,877 [2,249; 7,014] | 3,477 [2,001; 6,335] | 244 [111; 611] | 222 [100; 562] |
| Highest | 2,485 | 56% [41%; 69%] | 33 [15; 63] | 36 [16; 69] | 1,339 [592; 2,543] | 1,464 [640; 2,785] | 3 [1; 6] | 4 [2; 7] | 49 [32; 72] | 54 [34; 78] | 5 [2; 9] | 6 [3; 12] | 54 [35; 79] | 60 [38; 87] | 2.2 [1.4; 3.2] | 2.4 [1.5; 3.5] | 2 [1; 3] | 2 [1; 3] | 631 [251; 1,321] | 690 [276; 1,467] | 4,490 [2,545; 8,209] | 4,017 [2,263; 7,410] | 259 [114; 661] | 236 [102; 608] |
| Coast | 6,540 | 50% [36%; 62%] | 99 [43; 191] | 109 [48; 208] | 1,521 [656; 2,914] | 1,663 [735; 3,177] | 13 [6; 26] | 17 [8; 33] | 142 [89; 208] | 155 [98; 226] | 21 [9; 42] | 28 [12; 54] | 163 [103; 238] | 183 [116; 266] | 2.5 [1.6; 3.6] | 2.8 [1.8; 4.1] | 6 [4; 8] | 6 [4; 9] | 1,082 [430; 2,277] | 1,183 [478; 2,480] | 4,021 [2,311; 7,246] | 3,576 [2,056; 6,467] | 234 [106; 592] | 213 [95; 528] |
| Lowest | 1,249 | 48% [35%; 59%] | 20 [9; 39] | 22 [10; 42] | 1,613 [696; 3,091] | 1,764 [780; 3,369] | 3 [1; 7] | 5 [2; 9] | 38 [24; 57] | 42 [27; 61] | 5 [2; 11] | 7 [3; 14] | 44 [28; 64] | 49 [31; 72] | 3.5 [2.2; 5.1] | 3.9 [2.5; 5.7] | 2 [1; 2] | 2 [1; 3] | 150 [60; 316] | 164 [66; 345] | 2,887 [1,681; 5,172] | 2,573 [1,502; 4,632] | 223 [103; 561] | 204 [92; 500] |
| Lower | 1,327 | 51% [37%; 63%] | 21 [9; 40] | 23 [10; 44] | 1,589 [686; 3,045] | 1,738 [768; 3,319] | 3 [1; 6] | 4 [2; 8] | 34 [22; 50] | 37 [24; 55] | 5 [2; 11] | 7 [3; 14] | 40 [25; 58] | 44 [28; 65] | 3.0 [1.9; 4.4] | 3.4 [2.1; 4.9] | 1 [1; 2] | 2 [1; 2] | 158 [63; 333] | 173 [70; 363] | 3,401 [1,975; 6,095] | 3,024 [1,763; 5,449] | 227 [104; 569] | 207 [94; 508] |
| Middle | 1,290 | 49% [36%; 61%] | 20 [9; 38] | 22 [10; 41] | 1,538 [663; 2,946] | 1,681 [743; 3,211] | 3 [1; 5] | 4 [2; 7] | 27 [17; 39] | 29 [19; 43] | 4 [2; 8] | 5 [2; 10] | 31 [19; 45] | 34 [22; 50] | 2.4 [1.5; 3.5] | 2.7 [1.7; 3.9] | 1 [1; 2] | 1 [1; 2] | 156 [62; 327] | 170 [69; 356] | 4,259 [2,478; 7,629] | 3,790 [2,208; 6,832] | 234 [108; 588] | 214 [97; 525] |
| Higher | 1,330 | 51% [37%; 63%] | 20 [9; 38] | 22 [10; 41] | 1,482 [640; 2,840] | 1,621 [716; 3,095] | 2 [1; 3] | 2 [1; 4] | 21 [13; 31] | 23 [15; 34] | 3 [1; 5] | 4 [2; 7] | 24 [15; 35] | 27 [17; 39] | 1.8 [1.1; 2.6] | 2.0 [1.3; 2.9] | 1 [1; 1] | 1 [1; 1] | 249 [99; 524] | 272 [110; 571] | 5,551 [3,175; 10,014] | 4,947 [2,834; 8,973] | 239 [107; 606] | 217 [96; 540] |
| Highest | 1,345 | 51% [38%; 64%] | 19 [8; 36] | 20 [9; 39] | 1,391 [600; 2,664] | 1,520 [672; 2,904] | 2 [1; 4] | 3 [1; 5] | 21 [14; 31] | 23 [15; 34] | 4 [2; 7] | 5 [2; 9] | 25 [16; 36] | 28 [18; 41] | 1.9 [1.2; 2.7] | 2.1 [1.3; 3.0] | 1 [1; 1] | 1 [1; 1] | 369 [147; 776] | 403 [163; 845] | 5,250 [2,916; 9,615] | 4,646 [2,585; 8,575] | 248 [108; 640] | 225 [95; 570] |
| Central | 8,039 | 53% [39%; 66%] | 110 [48; 208] | 120 [53; 229] | 1,366 [600; 2,592] | 1,493 [656; 2,847] | 9 [4; 17] | 12 [5; 23] | 134 [86; 195] | 147 [94; 213] | 9 [4; 18] | 12 [5; 24] | 144 [92; 208] | 159 [102; 230] | 1.8 [1.1; 2.6] | 2.0 [1.3; 2.9] | 5 [3; 7] | 6 [4; 8] | 1,750 [697; 3,676] | 1,914 [762; 4,011] | 5,503 [3,131; 9,908] | 4,945 [2,838; 8,855] | 257 [114; 652] | 234 [103; 588] |
| Lowest | 1,523 | 51% [37%; 63%] | 23 [10; 44] | 25 [11; 48] | 1,522 [669; 2,888] | 1,664 [731; 3,173] | 3 [1; 5] | 3 [1; 6] | 32 [20; 46] | 35 [22; 50] | 2 [1; 5] | 3 [1; 6] | 34 [22; 49] | 38 [24; 55] | 2.2 [1.4; 3.2] | 2.5 [1.6; 3.6] | 1 [1; 2] | 1 [1; 2] | 264 [105; 554] | 288 [115; 604] | 4,446 [2,555; 7,968] | 3,997 [2,314; 7,117] | 233 [105; 588] | 212 [96; 531] |
| Lower | 1,548 | 52% [38%; 64%] | 22 [10; 42] | 24 [11; 47] | 1,441 [633; 2,735] | 1,576 [692; 3,005] | 2 [1; 4] | 3 [1; 6] | 29 [18; 41] | 31 [20; 45] | 2 [1; 4] | 3 [1; 6] | 31 [20; 45] | 34 [22; 49] | 2.0 [1.3; 2.9] | 2.2 [1.4; 3.2] | 1 [1; 2] | 1 [1; 2] | 275 [110; 578] | 301 [120; 631] | 5,014 [2,878; 8,986] | 4,505 [2,606; 8,026] | 246 [111; 620] | 224 [101; 560] |
| Middle | 1,677 | 56% [41%; 69%] | 23 [10; 44] | 25 [11; 48] | 1,375 [604; 2,609] | 1,503 [660; 2,866] | 2 [1; 4] | 3 [1; 5] | 28 [18; 41] | 31 [20; 45] | 2 [1; 4] | 3 [1; 5] | 31 [20; 44] | 34 [22; 49] | 1.8 [1.2; 2.6] | 2.0 [1.3; 2.9] | 1 [1; 2] | 1 [1; 2] | 302 [120; 634] | 330 [131; 692] | 5,470 [3,138; 9,809] | 4,917 [2,842; 8,763] | 258 [116; 650] | 235 [106; 587] |
| Higher | 1,634 | 54% [40%; 67%] | 22 [10; 41] | 24 [10; 45] | 1,328 [584; 2,520] | 1,452 [638; 2,769] | 1 [1; 3] | 2 [1; 3] | 26 [17; 38] | 28 [18; 41] | 1 [1; 3] | 2 [1; 3] | 27 [18; 40] | 30 [19; 44] | 1.7 [1.1; 2.4] | 1.8 [1.2; 2.7] | 1 [1; 1] | 1 [1; 2] | 384 [153; 807] | 420 [167; 880] | 5,855 [3,320; 10,596] | 5,273 [3,020; 9,443] | 263 [116; 670] | 239 [104; 604] |
| Highest | 1,656 | 55% [40%; 68%] | 20 [9; 37] | 21 [9; 41] | 1,180 [519; 2,239] | 1,290 [566; 2,460] | 1 [0; 2] | 1 [1; 2] | 20 [13; 29] | 22 [14; 31] | 1 [1; 3] | 2 [1; 3] | 21 [13; 31] | 23 [15; 34] | 1.3 [0.8; 1.8] | 1.4 [0.9; 2.0] | 1 [0; 1] | 1 [1; 1] | 525 [209; 1,103] | 574 [229; 1,203] | 7,518 [4,174; 13,682] | 6,742 [3,747; 12,244] | 289 [124; 748] | 262 [110; 672] |
| ZAMBIA |  |  |  |  |  |  |  |  |  |  |  |  |  |  |  |  |  |  |  |  |  |  |  |  |
| Western | 2,206 | 47% [34%; 58%] | 34 [15; 65] | 37 [16; 71] | 1,541 [671; 2,942] | 1,684 [732; 3,238] | 6 [2; 11] | 7 [3; 14] | 52 [28; 89] | 57 [31; 97] | 10 [4; 20] | 13 [5; 26] | 62 [35; 104] | 70 [39; 117] | 2.8 [1.6; 4.7] | 3.2 [1.8; 5.3] | 2 [1; 4] | 2 [1; 4] | 315 [126; 661] | 345 [137; 718] | 3,599 [1,861; 7,240] | 3,184 [1,658; 6,440] | 233 [105; 588] | 212 [95; 535] |
| Lowest | 390 | 42% [30%; 52%] | 6 [3; 12] | 7 [3; 13] | 1,611 [702; 3,076] | 1,761 [765; 3,386] | 1 [0; 2] | 1 [1; 3] | 11 [6; 19] | 12 [7; 21] | 2 [1; 4] | 2 [1; 5] | 13 [7; 21] | 14 [8; 24] | 3.3 [1.8; 5.5] | 3.7 [2.1; 6.2] | 0 [0; 1] | 1 [0; 1] | 36 [14; 75] | 39 [15; 81] | 3,120 [1,622; 6,253] | 2,775 [1,460; 5,591] | 226 [104; 565] | 206 [95; 515] |
| Lower | 426 | 46% [33%; 57%] | 7 [3; 13] | 7 [3; 14] | 1,588 [692; 3,032] | 1,736 [754; 3,338] | 1 [1; 3] | 2 [1; 3] | 12 [6; 20] | 13 [7; 22] | 3 [1; 5] | 3 [1; 7] | 14 [8; 24] | 16 [9; 27] | 3.4 [1.9; 5.6] | 3.8 [2.1; 6.3] | 1 [0; 1] | 1 [0; 1] | 39 [16; 83] | 43 [17; 90] | 3,080 [1,609; 6,160] | 2,717 [1,429; 5,450] | 229 [106; 573] | 209 [96; 522] |
| Middle | 432 | 46% [34%; 57%] | 7 [3; 13] | 7 [3; 14] | 1,561 [680; 2,980] | 1,706 [741; 3,280] | 1 [0; 2] | 1 [1; 3] | 10 [5; 17] | 11 [6; 18] | 2 [1; 4] | 2 [1; 5] | 12 [6; 20] | 13 [7; 22] | 2.7 [1.5; 4.5] | 3.1 [1.7; 5.1] | 0 [0; 1] | 0 [0; 1] | 34 [13; 70] | 37 [15; 76] | 3,812 [1,992; 7,622] | 3,377 [1,785; 6,778] | 234 [108; 584] | 213 [99; 532] |
| Higher | 469 | 50% [37%; 62%] | 7 [3; 14] | 8 [3; 15] | 1,549 [675; 2,958] | 1,694 [736; 3,256] | 1 [0; 2] | 1 [1; 3] | 11 [6; 19] | 12 [6; 21] | 2 [1; 3] | 2 [1; 4] | 13 [7; 21] | 14 [8; 24] | 2.7 [1.5; 4.5] | 3.0 [1.7; 5.0] | 0 [0; 1] | 0 [0; 1] | 48 [19; 100] | 52 [21; 109] | 3,807 [1,973; 7,635] | 3,394 [1,781; 6,845] | 234 [107; 587] | 213 [98; 535] |
| Highest | 490 | 53% [38%; 65%] | 7 [3; 13] | 8 [3; 15] | 1,418 [617; 2,707] | 1,550 [673; 2,979] | 1 [0; 2] | 1 [1; 3] | 9 [5; 15] | 9 [5; 16] | 2 [1; 4] | 3 [1; 5] | 11 [6; 18] | 12 [7; 20] | 2.2 [1.2; 3.7] | 2.5 [1.4; 4.2] | 0 [0; 1] | 0 [0; 1] | 159 [64; 333] | 173 [69; 362] | 4,382 [2,179; 9,022] | 3,831 [1,884; 7,954] | 240 [102; 627] | 218 [90; 570] |
| Southern | 3,886 | 45% [33%; 56%] | 56 [25; 108] | 61 [27; 117] | 1,447 [640; 2,779] | 1,582 [689; 3,022] | 9 [4; 18] | 12 [5; 23] | 72 [38; 123] | 79 [42; 133] | 14 [6; 28] | 18 [8; 36] | 86 [47; 144] | 97 [54; 161] | 2.2 [1.2; 3.7] | 2.5 [1.4; 4.2] | 3 [2; 5] | 3 [2; 6] | 691 [275; 1,445] | 756 [300; 1,580] | 4,558 [2,366; 9,331] | 4,026 [2,105; 8,152] | 245 [111; 606] | 223 [100; 553] |
| Lowest | 713 | 41% [30%; 52%] | 11 [5; 21] | 12 [5; 23] | 1,553 [687; 2,984] | 1,698 [740; 3,244] | 2 [1; 4] | 3 [1; 5] | 14 [8; 25] | 16 [8; 27] | 3 [1; 7] | 4 [2; 9] | 18 [10; 30] | 20 [11; 33] | 2.5 [1.4; 4.2] | 2.8 [1.6; 4.7] | 1 [0; 1] | 1 [0; 1] | 83 [33; 174] | 91 [36; 191] | 4,115 [2,160; 8,341] | 3,625 [1,918; 7,278] | 232 [107; 568] | 212 [98; 520] |
| Lower | 745 | 43% [32%; 54%] | 11 [5; 22] | 12 [5; 24] | 1,531 [677; 2,940] | 1,673 [729; 3,197] | 2 [1; 4] | 2 [1; 5] | 15 [8; 26] | 17 [9; 28] | 3 [1; 5] | 4 [2; 7] | 18 [10; 30] | 20 [11; 34] | 2.4 [1.3; 4.1] | 2.7 [1.5; 4.5] | 1 [0; 1] | 1 [0; 1] | 88 [35; 184] | 96 [38; 201] | 4,237 [2,219; 8,619] | 3,756 [1,985; 7,553] | 236 [109; 576] | 215 [99; 527] |
| Middle | 728 | 42% [31%; 53%] | 11 [5; 21] | 12 [5; 23] | 1,516 [670; 2,912] | 1,657 [722; 3,166] | 1 [1; 3] | 2 [1; 4] | 15 [8; 25] | 16 [8; 27] | 2 [1; 4] | 2 [1; 5] | 16 [9; 28] | 18 [10; 31] | 2.3 [1.2; 3.8] | 2.5 [1.4; 4.2] | 1 [0; 1] | 1 [0; 1] | 77 [30; 160] | 84 [33; 175] | 4,557 [2,383; 9,295] | 4,072 [2,152; 8,223] | 239 [111; 583] | 218 [101; 533] |
| Higher | 852 | 50% [36%; 62%] | 13 [6; 24] | 14 [6; 27] | 1,494 [660; 2,869] | 1,633 [711; 3,120] | 2 [1; 4] | 3 [1; 6] | 18 [9; 30] | 19 [10; 32] | 3 [1; 7] | 5 [2; 9] | 21 [12; 35] | 24 [13; 39] | 2.5 [1.4; 4.1] | 2.8 [1.5; 4.6] | 1 [0; 1] | 1 [0; 1] | 124 [49; 259] | 135 [54; 283] | 4,132 [2,158; 8,423] | 3,652 [1,921; 7,365] | 240 [110; 589] | 218 [100; 538] |
| Highest | 848 | 49% [36%; 61%] | 10 [4; 19] | 11 [5; 21] | 1,177 [520; 2,261] | 1,287 [561; 2,459] | 2 [1; 3] | 2 [1; 4] | 10 [5; 17] | 11 [6; 19] | 3 [1; 5] | 3 [1; 7] | 13 [7; 21] | 15 [8; 24] | 1.5 [0.8; 2.5] | 1.7 [1.0; 2.8] | 0 [0; 1] | 1 [0; 1] | 319 [127; 668] | 349 [139; 730] | 6,327 [3,093; 13,268] | 5,512 [2,686; 11,466] | 285 [119; 728] | 257 [107; 663] |
| North Western | 2,803 | 47% [34%; 58%] | 43 [19; 81] | 47 [20; 89] | 1,523 [662; 2,904] | 1,665 [730; 3,162] | 7 [3; 13] | 9 [4; 18] | 62 [33; 104] | 67 [36; 113] | 12 [5; 23] | 15 [6; 30] | 73 [40; 121] | 82 [46; 135] | 2.6 [1.4; 4.3] | 2.9 [1.6; 4.8] | 3 [1; 4] | 3 [2; 5] | 739 [293; 1,536] | 808 [324; 1,686] | 3,769 [1,894; 7,806] | 3,325 [1,681; 6,792] | 227 [98; 585] | 206 [90; 533] |
| Lowest | 492 | 41% [30%; 51%] | 8 [3; 15] | 8 [4; 16] | 1,575 [685; 3,003] | 1,722 [755; 3,270] | 1 [1; 3] | 2 [1; 3] | 12 [6; 20] | 13 [7; 22] | 2 [1; 4] | 3 [1; 6] | 14 [8; 24] | 16 [9; 26] | 2.9 [1.6; 4.8] | 3.3 [1.8; 5.4] | 1 [0; 1] | 1 [0; 1] | 102 [41; 213] | 112 [45; 233] | 3,442 [1,750; 7,091] | 3,043 [1,556; 6,173] | 223 [98; 570] | 203 [90; 520] |
| Lower | 561 | 47% [34%; 59%] | 9 [4; 17] | 10 [4; 18] | 1,555 [676; 2,965] | 1,700 [745; 3,229] | 2 [1; 3] | 2 [1; 4] | 14 [7; 24] | 15 [8; 26] | 3 [1; 6] | 4 [2; 7] | 17 [9; 28] | 19 [10; 31] | 3.0 [1.6; 5.0] | 3.4 [1.9; 5.5] | 1 [0; 1] | 1 [0; 1] | 118 [47; 246] | 129 [52; 270] | 3,344 [1,702; 6,879] | 2,950 [1,509; 5,985] | 226 [99; 577] | 206 [91; 526] |
| Middle | 578 | 49% [35%; 61%] | 9 [4; 17] | 10 [4; 19] | 1,541 [670; 2,938] | 1,685 [738; 3,200] | 1 [1; 3] | 2 [1; 3] | 13 [7; 23] | 15 [8; 24] | 2 [1; 4] | 3 [1; 6] | 16 [9; 26] | 17 [10; 29] | 2.7 [1.5; 4.5] | 3.0 [1.7; 5.0] | 1 [0; 1] | 1 [0; 1] | 112 [45; 233] | 123 [49; 256] | 3,729 [1,905; 7,671] | 3,305 [1,704; 6,713] | 229 [101; 583] | 209 [93; 533] |
| Higher | 560 | 47% [34%; 59%] | 9 [4; 17] | 9 [4; 18] | 1,545 [672; 2,946] | 1,689 [740; 3,208] | 1 [1; 3] | 2 [1; 4] | 12 [7; 21] | 14 [7; 23] | 2 [1; 5] | 3 [1; 6] | 15 [8; 25] | 17 [9; 28] | 2.7 [1.5; 4.4] | 3.0 [1.7; 4.9] | 1 [0; 1] | 1 [0; 1] | 126 [50; 262] | 138 [55; 288] | 3,733 [1,892; 7,689] | 3,293 [1,679; 6,693] | 227 [99; 580] | 206 [91; 529] |
| Highest | 611 | 51% [37%; 64%] | 9 [4; 16] | 9 [4; 18] | 1,414 [615; 2,696] | 1,546 [678; 2,936] | 1 [1; 2] | 2 [1; 3] | 10 [5; 16] | 11 [6; 18] | 2 [1; 4] | 2 [1; 5] | 12 [6; 19] | 13 [7; 21] | 1.9 [1.0; 3.2] | 2.1 [1.2; 3.5] | 0 [0; 1] | 0 [0; 1] | 280 [111; 583] | 307 [123; 640] | 4,885 [2,339; 10,319] | 4,280 [2,013; 8,984] | 231 [91; 616] | 208 [82; 561] |
| Northern | 1,752 | 47% [34%; 58%] | 27 [12; 52] | 30 [13; 56] | 1,547 [668; 2,947] | 1,691 [727; 3,214] | 6 [2; 11] | 8 [3; 14] | 52 [27; 89] | 57 [30; 97] | 14 [6; 28] | 19 [8; 36] | 67 [36; 110] | 76 [41; 126] | 3.8 [2.1; 6.3] | 4.3 [2.4; 7.2] | 2 [1; 4] | 3 [1; 4] | 258 [102; 530] | 282 [112; 586] | 2,683 [1,415; 5,430] | 2,350 [1,237; 4,754] | 231 [105; 591] | 211 [95; 537] |
| Lowest | 350 | 47% [34%; 58%] | 6 [2; 11] | 6 [3; 12] | 1,594 [688; 3,037] | 1,743 [750; 3,313] | 1 [1; 3] | 2 [1; 3] | 14 [7; 23] | 15 [8; 25] | 4 [1; 7] | 5 [2; 9] | 17 [9; 28] | 19 [11; 32] | 4.9 [2.7; 8.1] | 5.6 [3.0; 9.3] | 1 [0; 1] | 1 [0; 1] | 34 [14; 70] | 37 [15; 78] | 2,112 [1,124; 4,248] | 1,852 [988; 3,720] | 228 [104; 576] | 208 [96; 524] |
| Lower | 338 | 46% [33%; 56%] | 5 [2; 10] | 6 [3; 11] | 1,596 [689; 3,041] | 1,745 [751; 3,317] | 1 [0; 2] | 1 [1; 3] | 10 [5; 17] | 11 [6; 19] | 3 [1; 6] | 4 [2; 7] | 13 [7; 22] | 15 [8; 25] | 3.9 [2.1; 6.4] | 4.4 [2.4; 7.3] | 0 [0; 1] | 1 [0; 1] | 33 [13; 68] | 36 [14; 75] | 2,679 [1,426; 5,388] | 2,349 [1,252; 4,717] | 227 [104; 575] | 207 [95; 524] |
| Middle | 330 | 45% [32%; 55%] | 5 [2; 10] | 6 [2; 11] | 1,586 [685; 3,021] | 1,734 [746; 3,295] | 1 [0; 2] | 1 [1; 3] | 10 [5; 18] | 11 [6; 19] | 3 [1; 5] | 4 [1; 7] | 13 [7; 22] | 15 [8; 25] | 4.0 [2.2; 6.6] | 4.5 [2.5; 7.5] | 0 [0; 1] | 1 [0; 1] | 28 [11; 58] | 31 [12; 64] | 2,608 [1,391; 5,234] | 2,291 [1,227; 4,598] | 229 [106; 580] | 209 [97; 528] |
| Higher | 348 | 47% [34%; 58%] | 5 [2; 10] | 6 [3; 11] | 1,535 [663; 2,925] | 1,679 [722; 3,190] | 1 [0; 2] | 1 [1; 3] | 10 [5; 17] | 11 [6; 19] | 3 [1; 5] | 4 [1; 7] | 13 [7; 22] | 15 [8; 25] | 3.8 [2.1; 6.2] | 4.3 [2.3; 7.1] | 0 [0; 1] | 1 [0; 1] | 38 [15; 79] | 42 [17; 87] | 2,743 [1,457; 5,525] | 2,406 [1,281; 4,834] | 235 [108; 597] | 215 [98; 544] |
| Highest | 386 | 52% [38%; 64%] | 6 [2; 11] | 6 [3; 12] | 1,437 [620; 2,738] | 1,571 [676; 2,986] | 1 [0; 2] | 1 [1; 3] | 8 [4; 13] | 9 [5; 15] | 2 [1; 5] | 3 [1; 6] | 10 [6; 17] | 12 [6; 19] | 2.6 [1.4; 4.4] | 3.0 [1.6; 5.0] | 0 [0; 1] | 0 [0; 1] | 124 [49; 255] | 136 [54; 282] | 3,673 [1,855; 7,577] | 3,192 [1,578; 6,654] | 237 [100; 624] | 215 [90; 565] |
| Muchinga | 1,754 | 45% [33%; 56%] | 27 [12; 51] | 29 [13; 55] | 1,522 [659; 2,890] | 1,665 [719; 3,153] | 5 [2; 9] | 6 [3; 12] | 48 [25; 82] | 52 [28; 88] | 11 [4; 21] | 14 [6; 27] | 58 [32; 98] | 66 [36; 109] | 3.3 [1.8; 5.6] | 3.8 [2.0; 6.2] | 2 [1; 3] | 2 [1; 4] | 393 [154; 831] | 430 [172; 913] | 2,990 [1,524; 6,191] | 2,629 [1,348; 5,387] | 230 [102; 589] | 209 [91; 534] |
| Lowest | 319 | 41% [30%; 51%] | 5 [2; 9] | 5 [2; 10] | 1,547 [669; 2,937] | 1,692 [731; 3,204] | 1 [0; 2] | 1 [1; 3] | 11 [6; 20] | 13 [7; 21] | 3 [1; 5] | 4 [1; 7] | 14 [8; 24] | 16 [9; 27] | 4.4 [2.4; 7.4] | 5.0 [2.7; 8.3] | 0 [0; 1] | 1 [0; 1] | 54 [21; 115] | 60 [24; 126] | 2,278 [1,176; 4,677] | 2,003 [1,040; 4,074] | 230 [104; 583] | 209 [93; 529] |
| Lower | 345 | 45% [32%; 55%] | 5 [2; 10] | 6 [3; 11] | 1,541 [667; 2,926] | 1,685 [728; 3,192] | 1 [0; 2] | 1 [1; 2] | 10 [5; 17] | 11 [6; 19] | 2 [1; 4] | 2 [1; 5] | 12 [6; 20] | 13 [7; 22] | 3.5 [1.9; 5.8] | 3.9 [2.1; 6.4] | 0 [0; 1] | 0 [0; 1] | 60 [23; 126] | 65 [26; 138] | 2,911 [1,495; 6,014] | 2,578 [1,340; 5,256] | 231 [104; 585] | 210 [93; 531] |
| Middle | 353 | 46% [33%; 57%] | 5 [2; 10] | 6 [3; 11] | 1,552 [671; 2,946] | 1,697 [733; 3,213] | 1 [0; 2] | 1 [1; 3] | 10 [5; 17] | 11 [6; 18] | 2 [1; 5] | 3 [1; 6] | 12 [7; 20] | 14 [7; 22] | 3.4 [1.8; 5.7] | 3.8 [2.1; 6.3] | 0 [0; 1] | 0 [0; 1] | 59 [23; 124] | 64 [26; 136] | 2,996 [1,547; 6,149] | 2,634 [1,369; 5,357] | 229 [104; 581] | 209 [93; 528] |
| Higher | 358 | 47% [34%; 57%] | 6 [2; 11] | 6 [3; 11] | 1,546 [669; 2,936] | 1,691 [730; 3,202] | 1 [0; 2] | 1 [1; 2] | 9 [5; 16] | 10 [5; 17] | 2 [1; 4] | 3 [1; 5] | 11 [6; 19] | 13 [7; 21] | 3.2 [1.7; 5.3] | 3.6 [2.0; 6.0] | 0 [0; 1] | 0 [0; 1] | 69 [27; 146] | 75 [30; 160] | 3,155 [1,619; 6,501] | 2,778 [1,434; 5,670] | 228 [102; 582] | 208 [92; 528] |
| Highest | 378 | 49% [36%; 61%] | 5 [2; 10] | 6 [3; 11] | 1,435 [621; 2,723] | 1,568 [678; 2,971] | 1 [0; 2] | 1 [0; 2] | 7 [4; 12] | 8 [4; 13] | 2 [1; 4] | 2 [1; 5] | 9 [5; 15] | 10 [6; 17] | 2.4 [1.3; 4.0] | 2.7 [1.5; 4.4] | 0 [0; 1] | 0 [0; 1] | 151 [59; 320] | 165 [66; 351] | 4,009 [1,940; 8,447] | 3,497 [1,674; 7,322] | 232 [95; 613] | 210 [84; 553] |
| Lusaka | 6,205 | 51% [37%; 63%] | 81 [36; 157] | 89 [39; 172] | 1,307 [578; 2,534] | 1,429 [629; 2,772] | 13 [6; 25] | 17 [7; 32] | 74 [39; 125] | 81 [43; 136] | 17 [8; 35] | 23 [10; 45] | 91 [50; 150] | 103 [58; 169] | 1.5 [0.8; 2.4] | 1.7 [0.9; 2.7] | 3 [2; 5] | 4 [2; 6] | 824 [325; 1,750] | 901 [358; 1,936] | 6,952 [3,668; 13,976] | 6,118 [3,215; 12,133] | 275 [124; 690] | 251 [111; 623] |
| Lowest | 1,155 | 48% [35%; 59%] | 17 [7; 32] | 18 [8; 35] | 1,443 [638; 2,797] | 1,577 [694; 3,060] | 3 [1; 7] | 4 [2; 9] | 19 [10; 33] | 21 [11; 36] | 5 [2; 10] | 6 [3; 13] | 24 [13; 40] | 28 [16; 45] | 2.1 [1.2; 3.4] | 2.4 [1.3; 3.9] | 1 [0; 1] | 1 [1; 2] | 84 [33; 179] | 92 [37; 198] | 4,948 [2,648; 9,898] | 4,352 [2,318; 8,537] | 253 [116; 630] | 231 [105; 569] |
| Lower | 1,198 | 50% [36%; 61%] | 17 [7; 33] | 18 [8; 36] | 1,400 [619; 2,714] | 1,530 [673; 2,968] | 3 [1; 5] | 3 [1; 7] | 15 [8; 25] | 16 [8; 27] | 3 [1; 7] | 5 [2; 9] | 18 [10; 30] | 20 [11; 33] | 1.5 [0.8; 2.5] | 1.7 [1.0; 2.8] | 1 [0; 1] | 1 [0; 1] | 130 [51; 275] | 142 [56; 305] | 6,830 [3,628; 13,712] | 6,014 [3,174; 11,872] | 258 [117; 646] | 236 [106; 584] |
| Middle | 1,260 | 52% [38%; 65%] | 17 [8; 34] | 19 [8; 37] | 1,376 [608; 2,668] | 1,505 [662; 2,918] | 3 [1; 5] | 4 [2; 7] | 15 [8; 26] | 17 [9; 28] | 4 [2; 8] | 5 [2; 10] | 19 [11; 32] | 22 [12; 36] | 1.5 [0.9; 2.5] | 1.8 [1.0; 2.8] | 1 [0; 1] | 1 [0; 1] | 123 [48; 261] | 134 [53; 288] | 6,705 [3,565; 13,440] | 5,890 [3,121; 11,574] | 264 [120; 658] | 241 [109; 595] |
| Higher | 1,314 | 55% [40%; 67%] | 18 [8; 34] | 19 [8; 37] | 1,335 [590; 2,589] | 1,460 [642; 2,832] | 2 [1; 5] | 3 [1; 6] | 14 [8; 24] | 16 [8; 26] | 3 [1; 5] | 3 [1; 7] | 17 [9; 28] | 19 [11; 31] | 1.3 [0.7; 2.1] | 1.4 [0.8; 2.4] | 1 [0; 1] | 1 [0; 1] | 143 [56; 304] | 156 [62; 336] | 8,048 [4,269; 16,233] | 7,138 [3,770; 14,197] | 271 [123; 678] | 247 [111; 612] |
| Highest | 1,278 | 53% [39%; 66%] | 13 [6; 25] | 14 [6; 27] | 1,000 [442; 1,939] | 1,093 [481; 2,120] | 2 [1; 3] | 2 [1; 4] | 10 [5; 17] | 11 [6; 19] | 3 [1; 5] | 3 [1; 7] | 13 [7; 21] | 14 [8; 23] | 1.0 [0.6; 1.6] | 1.1 [0.6; 1.8] | 0 [0; 1] | 1 [0; 1] | 345 [136; 731] | 377 [150; 809] | 9,879 [5,021; 20,188] | 8,651 [4,367; 17,426] | 346 [147; 888] | 314 [131; 799] |
| Luapula | 2,237 | 44% [32%; 54%] | 33 [15; 64] | 36 [16; 69] | 1,477 [650; 2,859] | 1,615 [724; 3,086] | 7 [3; 13] | 9 [4; 17] | 39 [21; 67] | 43 [23; 73] | 15 [7; 30] | 20 [9; 39] | 55 [31; 90] | 63 [35; 103] | 2.4 [1.4; 4.0] | 2.8 [1.6; 4.6] | 2 [1; 3] | 2 [1; 4] | 188 [75; 400] | 205 [83; 429] | 4,259 [2,272; 8,381] | 3,690 [1,988; 7,295] | 247 [114; 607] | 225 [105; 547] |
| Lowest | 449 | 44% [32%; 55%] | 7 [3; 14] | 8 [3; 15] | 1,557 [685; 3,013] | 1,702 [763; 3,252] | 1 [1; 3] | 2 [1; 3] | 8 [4; 14] | 9 [5; 15] | 3 [1; 6] | 4 [2; 8] | 11 [6; 19] | 13 [7; 21] | 2.5 [1.4; 4.1] | 2.9 [1.6; 4.7] | 0 [0; 1] | 0 [0; 1] | 15 [6; 31] | 16 [6; 34] | 4,199 [2,260; 8,224] | 3,643 [1,986; 7,145] | 237 [112; 579] | 217 [103; 523] |
| Lower | 376 | 37% [27%; 46%] | 6 [2; 11] | 6 [3; 12] | 1,502 [661; 2,907] | 1,642 [736; 3,137] | 1 [1; 2] | 2 [1; 3] | 7 [4; 13] | 8 [4; 14] | 3 [1; 6] | 4 [2; 8] | 11 [6; 17] | 12 [7; 20] | 2.8 [1.6; 4.6] | 3.2 [1.8; 5.3] | 0 [0; 1] | 0 [0; 1] | 12 [5; 26] | 13 [5; 27] | 3,772 [2,034; 7,384] | 3,266 [1,780; 6,417] | 246 [116; 600] | 225 [106; 542] |
| Middle | 449 | 44% [32%; 55%] | 7 [3; 13] | 7 [3; 14] | 1,487 [654; 2,878] | 1,626 [729; 3,106] | 1 [1; 3] | 2 [1; 4] | 9 [5; 15] | 10 [5; 17] | 3 [1; 7] | 4 [2; 8] | 12 [7; 20] | 14 [8; 23] | 2.7 [1.5; 4.5] | 3.1 [1.7; 5.1] | 0 [0; 1] | 0 [0; 1] | 14 [5; 29] | 15 [6; 31] | 3,879 [2,084; 7,597] | 3,369 [1,839; 6,601] | 249 [117; 607] | 227 [108; 548] |
| Higher | 459 | 45% [33%; 56%] | 7 [3; 13] | 7 [3; 14] | 1,472 [648; 2,850] | 1,610 [722; 3,076] | 1 [1; 3] | 2 [1; 3] | 8 [4; 13] | 9 [5; 15] | 3 [1; 6] | 4 [2; 7] | 11 [6; 18] | 12 [7; 20] | 2.3 [1.3; 3.8] | 2.7 [1.5; 4.4] | 0 [0; 1] | 0 [0; 1] | 19 [7; 39] | 20 [8; 42] | 4,529 [2,431; 8,884] | 3,939 [2,144; 7,732] | 250 [117; 612] | 229 [108; 552] |
| Highest | 504 | 50% [36%; 62%] | 7 [3; 14] | 8 [3; 15] | 1,384 [609; 2,679] | 1,513 [679; 2,891] | 1 [1; 3] | 2 [1; 3] | 7 [4; 12] | 8 [4; 13] | 3 [1; 6] | 4 [2; 7] | 10 [6; 16] | 12 [6; 19] | 2.0 [1.1; 3.3] | 2.3 [1.3; 3.7] | 0 [0; 1] | 0 [0; 1] | 129 [52; 275] | 141 [57; 294] | 5,018 [2,561; 10,065] | 4,316 [2,213; 8,787] | 251 [108; 634] | 228 [99; 571] |
| Eastern | 4,142 | 49% [36%; 61%] | 60 [26; 115] | 66 [29; 127] | 1,454 [638; 2,787] | 1,590 [698; 3,075] | 11 [5; 21] | 14 [6; 28] | 79 [42; 133] | 86 [46; 147] | 25 [11; 50] | 33 [14; 65] | 104 [58; 170] | 119 [67; 195] | 2.5 [1.4; 4.1] | 2.9 [1.6; 4.7] | 4 [2; 6] | 4 [2; 7] | 508 [201; 1,072] | 555 [221; 1,193] | 4,086 [2,157; 8,043] | 3,562 [1,872; 7,012] | 248 [113; 613] | 226 [101; 560] |
| Lowest | 737 | 44% [32%; 55%] | 11 [5; 21] | 12 [5; 23] | 1,501 [658; 2,877] | 1,641 [721; 3,174] | 2 [1; 5] | 3 [1; 6] | 18 [9; 30] | 20 [10; 33] | 6 [3; 13] | 8 [4; 17] | 24 [14; 40] | 28 [16; 46] | 3.3 [1.8; 5.4] | 3.8 [2.1; 6.2] | 1 [0; 1] | 1 [1; 2] | 60 [24; 127] | 66 [26; 141] | 3,147 [1,682; 6,147] | 2,735 [1,452; 5,355] | 243 [112; 596] | 222 [101; 546] |
| Lower | 811 | 49% [35%; 60%] | 12 [5; 23] | 13 [6; 26] | 1,508 [661; 2,890] | 1,648 [724; 3,189] | 2 [1; 5] | 3 [1; 6] | 16 [8; 26] | 17 [9; 29] | 6 [3; 12] | 8 [3; 16] | 22 [12; 35] | 25 [14; 41] | 2.7 [1.5; 4.3] | 3.1 [1.7; 5.1] | 1 [0; 1] | 1 [0; 1] | 66 [26; 140] | 73 [29; 156] | 3,891 [2,070; 7,596] | 3,374 [1,784; 6,609] | 242 [112; 593] | 221 [100; 543] |
| Middle | 864 | 52% [38%; 64%] | 13 [6; 24] | 14 [6; 27] | 1,478 [648; 2,834] | 1,616 [710; 3,127] | 2 [1; 5] | 3 [1; 6] | 18 [9; 30] | 19 [10; 33] | 6 [2; 11] | 7 [3; 15] | 23 [13; 38] | 27 [15; 44] | 2.7 [1.5; 4.4] | 3.1 [1.7; 5.0] | 1 [0; 1] | 1 [1; 2] | 55 [22; 117] | 61 [24; 130] | 3,878 [2,075; 7,582] | 3,385 [1,800; 6,620] | 248 [115; 606] | 226 [104; 556] |
| Higher | 857 | 51% [37%; 64%] | 12 [5; 24] | 14 [6; 26] | 1,444 [633; 2,768] | 1,579 [694; 3,054] | 2 [1; 4] | 2 [1; 5] | 15 [8; 26] | 17 [9; 28] | 4 [2; 8] | 5 [2; 10] | 19 [11; 31] | 22 [12; 36] | 2.2 [1.2; 3.6] | 2.5 [1.4; 4.2] | 1 [0; 1] | 1 [0; 1] | 67 [27; 142] | 73 [29; 158] | 4,640 [2,463; 9,115] | 4,082 [2,157; 8,015] | 253 [117; 620] | 231 [105; 568] |
| Highest | 873 | 52% [38%; 65%] | 12 [5; 23] | 13 [6; 25] | 1,351 [592; 2,589] | 1,477 [649; 2,857] | 2 [1; 3] | 2 [1; 4] | 12 [7; 21] | 14 [7; 23] | 3 [1; 6] | 4 [2; 8] | 16 [9; 25] | 18 [10; 29] | 1.8 [1.0; 2.9] | 2.0 [1.1; 3.4] | 1 [0; 1] | 1 [0; 1] | 259 [102; 546] | 283 [112; 608] | 5,447 [2,766; 10,968] | 4,755 [2,365; 9,625] | 254 [108; 646] | 230 [95; 589] |
| Copperbelt | 5,855 | 52% [36%; 61%] | 69 [26; 115] | 76 [28; 127] | 1,183 [630; 2,783] | 1,294 [686; 3,056] | 11 [5; 21] | 15 [6; 28] | 89 [42; 134] | 98 [46; 146] | 16 [11; 49] | 21 [14; 64] | 105 [58; 171] | 118 [67; 196] | 1.8 [1.0; 2.9] | 2.0 [1.1; 3.3] | 4 [2; 6] | 4 [2; 7] | 1,241 [199; 1,067] | 1,356 [218; 1,188] | 5,548 [2,163; 8,176] | 4,908 [1,862; 7,071] | 297 [114; 628] | 270 [103; 570] |
| Lowest | 1,106 | 50% [32%; 55%] | 17 [5; 21] | 19 [5; 23] | 1,549 [651; 2,872] | 1,693 [708; 3,154] | 3 [1; 5] | 4 [1; 6] | 27 [9; 30] | 30 [11; 33] | 5 [3; 13] | 6 [4; 16] | 32 [13; 40] | 36 [16; 46] | 2.9 [1.2; 3.6] | 3.2 [1.4; 4.2] | 1 [0; 1] | 1 [1; 2] | 107 [24; 126] | 117 [26; 141] | 3,570 [1,678; 6,256] | 3,170 [1,444; 5,403] | 234 [114; 612] | 214 [102; 554] |
| Lower | 1,168 | 53% [35%; 61%] | 17 [5; 23] | 18 [6; 26] | 1,441 [654; 2,886] | 1,576 [711; 3,169] | 3 [1; 5] | 4 [1; 6] | 21 [8; 27] | 23 [9; 29] | 4 [3; 12] | 5 [3; 15] | 25 [12; 36] | 28 [14; 41] | 2.1 [1.0; 3.1] | 2.4 [1.2; 3.5] | 1 [0; 1] | 1 [0; 1] | 216 [26; 139] | 236 [29; 155] | 4,688 [2,077; 7,730] | 4,142 [1,782; 6,671] | 246 [113; 609] | 224 [102; 552] |
| Middle | 1,168 | 53% [38%; 64%] | 15 [6; 24] | 17 [6; 27] | 1,307 [641; 2,830] | 1,429 [698; 3,108] | 2 [1; 5] | 3 [1; 6] | 16 [9; 30] | 18 [10; 33] | 3 [2; 11] | 4 [3; 14] | 19 [13; 38] | 22 [15; 44] | 1.7 [1.1; 3.3] | 1.9 [1.3; 3.7] | 1 [0; 1] | 1 [1; 2] | 214 [22; 116] | 234 [24; 130] | 6,076 [2,071; 7,728] | 5,378 [1,792; 6,668] | 271 [117; 623] | 247 [105; 564] |
| Higher | 1,214 | 55% [37%; 64%] | 12 [5; 24] | 13 [6; 26] | 986 [626; 2,764] | 1,078 [681; 3,035] | 2 [1; 4] | 3 [1; 5] | 16 [8; 26] | 17 [9; 28] | 3 [2; 8] | 4 [2; 10] | 19 [11; 31] | 21 [12; 36] | 1.6 [0.9; 2.6] | 1.8 [1.0; 3.0] | 1 [0; 1] | 1 [0; 1] | 253 [26; 141] | 277 [29; 157] | 6,425 [2,463; 9,259] | 5,680 [2,154; 8,050] | 357 [118; 637] | 324 [106; 576] |
| Highest | 1,199 | 54% [38%; 65%] | 8 [5; 23] | 9 [6; 25] | 675 [586; 2,586] | 738 [637; 2,839] | 1 [1; 3] | 1 [1; 4] | 9 [7; 21] | 10 [7; 23] | 1 [1; 6] | 2 [2; 8] | 10 [9; 26] | 11 [10; 29] | 0.8 [0.7; 2.1] | 0.9 [0.8; 2.4] | 0 [0; 1] | 0 [0; 1] | 451 [101; 543] | 493 [111; 605] | 11,281 [2,766; 11,133] | 9,971 [2,354; 9,693] | 496 [110; 664] | 449 [98; 599] |
| Central | 3,410 | 46% [34%; 57%] | 49 [21; 94] | 53 [23; 103] | 1,425 [624; 2,752] | 1,558 [685; 3,017] | 9 [4; 18] | 12 [5; 24] | 70 [37; 119] | 77 [41; 132] | 19 [8; 37] | 24 [10; 49] | 89 [50; 148] | 101 [56; 169] | 2.6 [1.5; 4.3] | 3.0 [1.6; 4.9] | 3 [2; 5] | 4 [2; 6] | 811 [327; 1,730] | 886 [359; 1,896] | 3,803 [1,949; 7,762] | 3,326 [1,670; 6,785] | 245 [106; 617] | 222 [95; 561] |
| Lowest | 675 | 46% [33%; 57%] | 10 [4; 20] | 11 [5; 21] | 1,495 [654; 2,887] | 1,634 [719; 3,165] | 2 [1; 4] | 3 [1; 6] | 18 [10; 31] | 20 [11; 34] | 5 [2; 9] | 6 [3; 12] | 23 [13; 38] | 26 [14; 43] | 3.4 [1.9; 5.6] | 3.8 [2.1; 6.4] | 1 [0; 1] | 1 [1; 2] | 124 [50; 264] | 135 [55; 289] | 2,981 [1,550; 6,027] | 2,612 [1,337; 5,280] | 237 [104; 592] | 216 [94; 538] |
| Lower | 685 | 47% [34%; 58%] | 10 [4; 19] | 11 [5; 21] | 1,473 [645; 2,845] | 1,610 [708; 3,118] | 2 [1; 4] | 3 [1; 5] | 13 [7; 23] | 15 [8; 25] | 4 [2; 8] | 5 [2; 10] | 17 [10; 29] | 20 [11; 33] | 2.5 [1.4; 4.2] | 2.9 [1.6; 4.8] | 1 [0; 1] | 1 [0; 1] | 128 [52; 273] | 140 [57; 299] | 3,968 [2,059; 8,039] | 3,464 [1,770; 7,023] | 240 [106; 601] | 219 [96; 546] |
| Middle | 618 | 42% [31%; 52%] | 9 [4; 18] | 10 [4; 19] | 1,485 [650; 2,868] | 1,623 [714; 3,144] | 2 [1; 3] | 2 [1; 5] | 13 [7; 22] | 14 [8; 25] | 4 [2; 7] | 5 [2; 9] | 17 [9; 28] | 19 [11; 32] | 2.7 [1.5; 4.5] | 3.1 [1.7; 5.1] | 1 [0; 1] | 1 [0; 1] | 109 [44; 232] | 119 [48; 254] | 3,730 [1,941; 7,547] | 3,265 [1,671; 6,601] | 239 [106; 596] | 218 [96; 542] |
| Higher | 700 | 48% [35%; 59%] | 10 [5; 20] | 11 [5; 22] | 1,474 [645; 2,847] | 1,612 [709; 3,121] | 2 [1; 4] | 2 [1; 5] | 16 [8; 27] | 17 [9; 30] | 4 [2; 7] | 5 [2; 9] | 20 [11; 32] | 22 [12; 37] | 2.8 [1.6; 4.6] | 3.2 [1.7; 5.2] | 1 [0; 1] | 1 [0; 1] | 137 [55; 293] | 150 [61; 321] | 3,601 [1,867; 7,307] | 3,167 [1,620; 6,408] | 239 [105; 599] | 218 [95; 545] |
| Highest | 732 | 50% [36%; 62%] | 9 [4; 17] | 10 [4; 19] | 1,218 [533; 2,353] | 1,332 [586; 2,579] | 2 [1; 3] | 2 [1; 4] | 10 [5; 16] | 11 [6; 18] | 3 [1; 6] | 4 [2; 7] | 13 [7; 21] | 14 [8; 24] | 1.7 [1.0; 2.9] | 2.0 [1.1; 3.3] | 0 [0; 1] | 1 [0; 1] | 313 [126; 668] | 342 [139; 732] | 5,484 [2,643; 11,483] | 4,755 [2,215; 9,973] | 271 [107; 707] | 245 [96; 641] |
| ZIMBABWE |  |  |  |  |  |  |  |  |  |  |  |  |  |  |  |  |  |  |  |  |  |  |  |  |
| Bulawayo | 1,356 | 50% [36%; 62%] | 14 [6; 27] | 15 [7; 29] | 1,034 [453; 1,992] | 1,131 [500; 2,170] | 1 [0; 2] | 1 [1; 2] | 53 [31; 83] | 58 [34; 91] | 2 [1; 4] | 2 [1; 4] | 55 [33; 86] | 60 [36; 94] | 4.1 [2.4; 6.3] | 4.4 [2.6; 6.9] | 2 [1; 3] | 2 [1; 3] | 183 [89; 341] | 200 [98; 374] | 2,499 [1,393; 4,718] | 2,278 [1,261; 4,339] | 347 [158; 844] | 316 [144; 773] |
| Lowest | 260 | 48% [35%; 59%] | 3 [2; 7] | 4 [2; 7] | 1,332 [584; 2,565] | 1,456 [644; 2,794] | 0 [0; 1] | 0 [0; 1] | 14 [8; 22] | 16 [9; 25] | 1 [0; 2] | 1 [0; 2] | 15 [9; 24] | 17 [10; 26] | 5.9 [3.5; 9.1] | 6.4 [3.8; 10.0] | 1 [0; 1] | 1 [0; 1] | 27 [13; 50] | 29 [14; 54] | 1,743 [976; 3,285] | 1,591 [887; 3,000] | 272 [125; 658] | 248 [114; 603] |
| Lower | 270 | 49% [36%; 61%] | 3 [2; 7] | 4 [2; 7] | 1,270 [557; 2,447] | 1,389 [615; 2,665] | 0 [0; 1] | 0 [0; 1] | 12 [7; 19] | 13 [8; 21] | 0 [0; 1] | 0 [0; 1] | 13 [7; 20] | 14 [8; 21] | 4.7 [2.8; 7.3] | 5.1 [3.0; 8.0] | 0 [0; 1] | 0 [0; 1] | 34 [16; 63] | 37 [18; 69] | 2,185 [1,219; 4,124] | 1,993 [1,104; 3,790] | 283 [129; 688] | 258 [118; 630] |
| Middle | 285 | 52% [38%; 65%] | 3 [1; 6] | 3 [1; 6] | 1,035 [454; 1,993] | 1,131 [501; 2,171] | 0 [0; 0] | 0 [0; 1] | 12 [7; 19] | 13 [8; 21] | 0 [0; 1] | 0 [0; 1] | 12 [7; 19] | 13 [8; 21] | 4.3 [2.6; 6.7] | 4.7 [2.8; 7.4] | 0 [0; 1] | 0 [0; 1] | 28 [14; 53] | 31 [15; 58] | 2,367 [1,325; 4,468] | 2,160 [1,201; 4,106] | 350 [160; 847] | 320 [147; 776] |
| Higher | 288 | 53% [38%; 65%] | 2 [1; 4] | 2 [1; 5] | 760 [333; 1,465] | 831 [368; 1,596] | 0 [0; 0] | 0 [0; 0] | 9 [5; 13] | 9 [6; 15] | 0 [0; 1] | 0 [0; 1] | 9 [5; 14] | 10 [6; 15] | 3.1 [1.8; 4.8] | 3.4 [2.0; 5.3] | 0 [0; 0] | 0 [0; 1] | 40 [19; 74] | 43 [21; 81] | 3,291 [1,831; 6,228] | 3,000 [1,660; 5,724] | 472 [214; 1,147] | 430 [195; 1,050] |
| Highest | 253 | 46% [34%; 58%] | 2 [1; 4] | 2 [1; 4] | 788 [345; 1,518] | 862 [381; 1,653] | 0 [0; 0] | 0 [0; 0] | 6 [4; 9] | 6 [4; 10] | 0 [0; 0] | 0 [0; 0] | 6 [4; 9] | 7 [4; 10] | 2.4 [1.4; 3.7] | 2.6 [1.5; 4.1] | 0 [0; 0] | 0 [0; 0] | 54 [27; 102] | 59 [29; 111] | 4,171 [2,289; 7,955] | 3,793 [2,068; 7,329] | 446 [198; 1,094] | 405 [179; 1,001] |
| Harare | 3,706 | 47% [34%; 57%] | 53 [23; 100] | 58 [25; 109] | 1,431 [618; 2,711] | 1,565 [674; 2,951] | 5 [2; 10] | 6 [2; 11] | 387 [228; 603] | 423 [250; 662] | 22 [9; 43] | 23 [10; 47] | 409 [241; 636] | 447 [264; 697] | 11.0 [6.5; 17.2] | 12.0 [7.1; 18.8] | 14 [9; 23] | 16 [9; 25] | 387 [185; 720] | 423 [202; 798] | 929 [522; 1,750] | 848 [479; 1,598] | 253 [118; 640] | 231 [109; 578] |
| Lowest | 656 | 42% [30%; 51%] | 10 [5; 20] | 11 [5; 21] | 1,590 [687; 3,012] | 1,738 [749; 3,278] | 2 [1; 4] | 2 [1; 4] | 90 [53; 140] | 98 [58; 154] | 9 [4; 18] | 10 [4; 19] | 99 [58; 154] | 108 [64; 168] | 15.1 [8.9; 23.4] | 16.5 [9.7; 25.6] | 3 [2; 5] | 4 [2; 6] | 47 [22; 87] | 51 [24; 96] | 687 [389; 1,292] | 627 [357; 1,177] | 230 [108; 579] | 210 [100; 523] |
| Lower | 732 | 46% [34%; 57%] | 12 [5; 23] | 13 [6; 25] | 1,637 [707; 3,101] | 1,790 [771; 3,375] | 2 [1; 3] | 2 [1; 4] | 86 [51; 134] | 94 [56; 147] | 6 [3; 13] | 7 [3; 14] | 93 [54; 144] | 101 [60; 158] | 12.6 [7.4; 19.7] | 13.8 [8.2; 21.5] | 3 [2; 5] | 4 [2; 6] | 67 [32; 125] | 74 [35; 139] | 813 [458; 1,529] | 742 [421; 1,397] | 222 [104; 561] | 203 [96; 506] |
| Middle | 806 | 51% [37%; 63%] | 12 [5; 23] | 14 [6; 25] | 1,533 [662; 2,904] | 1,676 [722; 3,161] | 1 [0; 2] | 1 [0; 2] | 84 [50; 131] | 92 [54; 144] | 1 [1; 3] | 1 [1; 3] | 86 [50; 133] | 94 [55; 146] | 10.6 [6.3; 16.6] | 11.6 [6.9; 18.2] | 3 [2; 5] | 3 [2; 5] | 54 [26; 100] | 59 [28; 111] | 973 [549; 1,825] | 888 [503; 1,673] | 239 [112; 601] | 218 [104; 543] |
| Higher | 716 | 45% [33%; 56%] | 10 [4; 19] | 11 [5; 20] | 1,383 [597; 2,621] | 1,512 [651; 2,852] | 1 [0; 1] | 1 [0; 1] | 59 [35; 92] | 64 [38; 100] | 3 [1; 7] | 4 [2; 8] | 62 [37; 97] | 68 [40; 106] | 8.7 [5.1; 13.5] | 9.5 [5.6; 14.8] | 2 [1; 3] | 2 [1; 4] | 75 [36; 139] | 82 [39; 154] | 1,178 [662; 2,216] | 1,075 [608; 2,026] | 262 [122; 662] | 239 [113; 598] |
| Highest | 798 | 51% [37%; 62%] | 8 [4; 16] | 9 [4; 17] | 1,052 [454; 1,993] | 1,150 [495; 2,169] | 0 [0; 1] | 0 [0; 1] | 68 [40; 106] | 74 [44; 116] | 1 [1; 3] | 1 [1; 3] | 69 [41; 108] | 76 [45; 119] | 8.7 [5.1; 13.6] | 9.5 [5.6; 14.9] | 2 [1; 4] | 3 [2; 4] | 145 [69; 269] | 158 [75; 298] | 1,149 [640; 2,184] | 1,046 [582; 1,990] | 337 [154; 860] | 307 [141; 774] |
| Masvingo | 2,765 | 44% [32%; 54%] | 49 [22; 95] | 54 [23; 104] | 1,788 [782; 3,429] | 1,955 [845; 3,768] | 6 [2; 11] | 6 [3; 12] | 476 [283; 746] | 520 [305; 814] | 36 [15; 71] | 39 [16; 78] | 511 [305; 799] | 559 [329; 875] | 18.5 [11.0; 28.9] | 20.2 [11.9; 31.6] | 18 [11; 28] | 20 [12; 31] | 275 [133; 519] | 301 [145; 564] | 555 [308; 1,030] | 506 [276; 960] | 203 [94; 491] | 185 [85; 456] |
| Lowest | 508 | 40% [29%; 50%] | 10 [5; 20] | 11 [5; 22] | 2,063 [902; 3,956] | 2,255 [975; 4,347] | 2 [1; 3] | 2 [1; 3] | 124 [73; 194] | 135 [79; 212] | 10 [4; 20] | 11 [5; 22] | 134 [80; 209] | 146 [86; 230] | 26.3 [15.7; 41.2] | 28.8 [17.0; 45.1] | 5 [3; 7] | 5 [3; 8] | 34 [16; 64] | 37 [18; 70] | 393 [220; 728] | 359 [197; 678] | 177 [83; 428] | 162 [75; 398] |
| Lower | 589 | 47% [34%; 58%] | 12 [5; 22] | 13 [5; 24] | 1,956 [855; 3,751] | 2,139 [925; 4,122] | 2 [1; 4] | 2 [1; 4] | 114 [68; 179] | 125 [73; 195] | 13 [5; 25] | 14 [6; 28] | 127 [76; 198] | 139 [82; 217] | 21.6 [12.8; 33.7] | 23.6 [13.9; 36.8] | 4 [3; 7] | 5 [3; 8] | 53 [25; 100] | 58 [28; 108] | 478 [265; 888] | 436 [239; 823] | 186 [86; 450] | 170 [78; 418] |
| Middle | 560 | 44% [32%; 55%] | 11 [5; 20] | 12 [5; 22] | 1,894 [828; 3,631] | 2,070 [895; 3,990] | 1 [0; 2] | 1 [0; 2] | 115 [68; 180] | 125 [73; 196] | 4 [2; 9] | 5 [2; 9] | 119 [71; 186] | 130 [76; 203] | 21.2 [12.6; 33.1] | 23.2 [13.6; 36.3] | 4 [3; 7] | 5 [3; 7] | 36 [18; 68] | 40 [19; 74] | 487 [272; 903] | 445 [243; 837] | 193 [90; 466] | 177 [82; 433] |
| Higher | 578 | 46% [33%; 57%] | 10 [5; 20] | 11 [5; 22] | 1,791 [783; 3,435] | 1,959 [847; 3,775] | 1 [0; 2] | 1 [0; 2] | 80 [48; 126] | 88 [51; 137] | 5 [2; 10] | 5 [2; 11] | 85 [51; 133] | 93 [55; 145] | 14.7 [8.8; 23.0] | 16.1 [9.5; 25.2] | 3 [2; 5] | 3 [2; 5] | 59 [28; 111] | 65 [31; 121] | 696 [386; 1,294] | 636 [346; 1,204] | 202 [93; 490] | 185 [84; 455] |
| Highest | 529 | 42% [31%; 52%] | 6 [3; 12] | 7 [3; 14] | 1,221 [534; 2,342] | 1,335 [577; 2,574] | 1 [0; 1] | 1 [0; 1] | 43 [26; 68] | 47 [28; 74] | 3 [1; 7] | 4 [1; 7] | 47 [28; 73] | 51 [30; 80] | 8.8 [5.2; 13.7] | 9.6 [5.7; 15.1] | 2 [1; 3] | 2 [1; 3] | 93 [45; 175] | 101 [49; 190] | 1,142 [627; 2,134] | 1,040 [560; 1,975] | 291 [132; 711] | 265 [118; 660] |
| Midlands | 2,787 | 40% [29%; 49%] | 49 [21; 95] | 53 [23; 102] | 1,750 [756; 3,405] | 1,914 [828; 3,674] | 6 [3; 12] | 7 [3; 13] | 609 [358; 961] | 666 [393; 1,049] | 54 [22; 112] | 59 [24; 121] | 663 [394; 1,046] | 725 [429; 1,142] | 23.8 [14.1; 37.5] | 26.0 [15.4; 41.0] | 23 [14; 37] | 26 [15; 40] | 160 [78; 300] | 175 [85; 326] | 436 [239; 814] | 399 [219; 747] | 210 [96; 523] | 191 [89; 474] |
| Lowest | 570 | 41% [30%; 51%] | 11 [5; 22] | 13 [5; 24] | 2,008 [867; 3,907] | 2,196 [950; 4,216] | 2 [1; 3] | 2 [1; 4] | 147 [86; 231] | 160 [94; 252] | 13 [5; 26] | 14 [6; 28] | 159 [95; 251] | 174 [103; 274] | 27.9 [16.6; 44.0] | 30.5 [18.0; 48.0] | 6 [3; 9] | 6 [4; 10] | 15 [7; 28] | 16 [8; 30] | 375 [207; 696] | 343 [189; 641] | 184 [85; 458] | 168 [79; 414] |
| Lower | 587 | 42% [31%; 52%] | 11 [5; 22] | 12 [5; 24] | 1,914 [827; 3,723] | 2,093 [906; 4,018] | 1 [1; 3] | 2 [1; 3] | 158 [93; 249] | 173 [102; 272] | 11 [5; 24] | 12 [5; 26] | 169 [101; 267] | 185 [109; 291] | 28.9 [17.1; 45.5] | 31.5 [18.6; 49.6] | 6 [4; 9] | 7 [4; 10] | 28 [14; 53] | 31 [15; 58] | 360 [198; 670] | 329 [181; 616] | 192 [88; 479] | 175 [82; 434] |
| Middle | 495 | 35% [26%; 44%] | 9 [4; 18] | 10 [4; 19] | 1,844 [797; 3,587] | 2,016 [873; 3,871] | 1 [1; 3] | 1 [1; 3] | 115 [68; 182] | 126 [74; 199] | 11 [4; 23] | 12 [5; 25] | 126 [75; 199] | 138 [82; 218] | 25.5 [15.1; 40.3] | 27.9 [16.5; 43.9] | 4 [3; 7] | 5 [3; 8] | 11 [5; 21] | 12 [6; 23] | 411 [227; 764] | 376 [207; 703] | 201 [93; 500] | 184 [86; 451] |
| Higher | 575 | 41% [30%; 51%] | 10 [4; 19] | 11 [5; 21] | 1,711 [739; 3,328] | 1,870 [809; 3,591] | 1 [0; 2] | 1 [1; 2] | 98 [58; 155] | 108 [63; 170] | 12 [5; 25] | 13 [5; 27] | 110 [65; 174] | 121 [71; 190] | 19.2 [11.3; 30.3] | 21.0 [12.4; 33.1] | 4 [2; 6] | 4 [3; 7] | 33 [16; 62] | 36 [18; 67] | 542 [297; 1,014] | 495 [271; 932] | 214 [98; 535] | 196 [91; 485] |
| Highest | 559 | 40% [29%; 50%] | 7 [3; 14] | 8 [3; 15] | 1,273 [550; 2,476] | 1,392 [602; 2,672] | 1 [0; 2] | 1 [0; 2] | 91 [53; 143] | 99 [58; 156] | 7 [3; 15] | 8 [3; 16] | 98 [58; 154] | 107 [63; 168] | 17.5 [10.4; 27.6] | 19.1 [11.3; 30.1] | 3 [2; 5] | 4 [2; 6] | 72 [35; 136] | 79 [38; 148] | 581 [315; 1,091] | 530 [286; 1,002] | 283 [127; 711] | 258 [117; 644] |
| Matabeleland South | 1,287 | 44% [32%; 55%] | 22 [10; 43] | 24 [10; 47] | 1,715 [757; 3,317] | 1,875 [815; 3,653] | 3 [1; 5] | 3 [1; 6] | 164 [96; 256] | 179 [106; 280] | 15 [6; 30] | 16 [7; 32] | 179 [105; 279] | 195 [115; 305] | 13.9 [8.2; 21.7] | 15.2 [9.0; 23.7] | 6 [4; 10] | 7 [4; 11] | 127 [60; 237] | 138 [66; 265] | 740 [409; 1,420] | 676 [373; 1,293] | 212 [97; 523] | 193 [88; 478] |
| Lowest | 240 | 42% [30%; 52%] | 5 [2; 9] | 5 [2; 10] | 1,879 [830; 3,635] | 2,055 [894; 4,003] | 1 [0; 1] | 1 [0; 2] | 41 [24; 64] | 45 [27; 70] | 4 [2; 8] | 4 [2; 9] | 45 [27; 71] | 49 [29; 77] | 18.8 [11.1; 29.4] | 20.6 [12.2; 32.2] | 2 [1; 2] | 2 [1; 3] | 16 [8; 30] | 17 [8; 33] | 550 [306; 1,051] | 503 [279; 958] | 195 [90; 479] | 178 [82; 438] |
| Lower | 225 | 39% [29%; 49%] | 4 [2; 8] | 4 [2; 9] | 1,803 [796; 3,488] | 1,972 [858; 3,841] | 1 [0; 1] | 1 [0; 1] | 35 [20; 54] | 38 [22; 59] | 4 [2; 8] | 4 [2; 8] | 38 [23; 60] | 42 [25; 66] | 17.1 [10.1; 26.6] | 18.7 [11.1; 29.1] | 1 [1; 2] | 1 [1; 2] | 20 [10; 37] | 22 [10; 42] | 604 [334; 1,155] | 551 [305; 1,053] | 202 [93; 498] | 184 [84; 455] |
| Middle | 279 | 49% [35%; 60%] | 5 [2; 9] | 5 [2; 10] | 1,752 [773; 3,389] | 1,915 [833; 3,732] | 1 [0; 1] | 1 [0; 1] | 33 [19; 51] | 36 [21; 56] | 4 [2; 8] | 5 [2; 9] | 37 [22; 57] | 40 [24; 63] | 13.2 [7.8; 20.6] | 14.4 [8.5; 22.5] | 1 [1; 2] | 1 [1; 2] | 18 [8; 33] | 19 [9; 37] | 787 [439; 1,504] | 719 [400; 1,370] | 209 [97; 514] | 191 [88; 470] |
| Higher | 277 | 48% [35%; 60%] | 5 [2; 9] | 5 [2; 10] | 1,739 [767; 3,363] | 1,901 [827; 3,703] | 1 [0; 1] | 1 [0; 1] | 34 [20; 53] | 37 [22; 58] | 2 [1; 3] | 2 [1; 4] | 35 [21; 55] | 39 [23; 61] | 12.8 [7.5; 20.0] | 14.0 [8.3; 22.0] | 1 [1; 2] | 1 [1; 2] | 28 [13; 52] | 30 [14; 58] | 799 [442; 1,531] | 729 [403; 1,398] | 209 [96; 516] | 190 [87; 472] |
| Highest | 266 | 46% [34%; 57%] | 4 [2; 7] | 4 [2; 8] | 1,428 [630; 2,761] | 1,561 [679; 3,041] | 0 [0; 0] | 0 [0; 1] | 22 [13; 34] | 24 [14; 37] | 1 [0; 2] | 1 [0; 2] | 23 [13; 35] | 25 [15; 39] | 8.5 [5.0; 13.2] | 9.3 [5.5; 14.6] | 1 [0; 1] | 1 [1; 1] | 45 [22; 85] | 50 [24; 95] | 1,182 [645; 2,281] | 1,077 [588; 2,080] | 249 [113; 622] | 227 [102; 568] |
| Matabeleland North | 1,661 | 51% [37%; 63%] | 30 [13; 57] | 33 [14; 63] | 1,798 [790; 3,456] | 1,966 [859; 3,779] | 3 [1; 6] | 3 [1; 6] | 161 [95; 252] | 176 [104; 277] | 10 [4; 21] | 11 [5; 23] | 171 [102; 268] | 187 [111; 293] | 10.3 [6.1; 16.1] | 11.3 [6.7; 17.6] | 6 [4; 9] | 7 [4; 10] | 311 [151; 590] | 340 [166; 639] | 972 [535; 1,833] | 885 [487; 1,682] | 197 [89; 494] | 179 [81; 450] |
| Lowest | 339 | 52% [38%; 64%] | 7 [3; 13] | 7 [3; 14] | 1,949 [856; 3,746] | 2,131 [931; 4,096] | 1 [0; 1] | 1 [0; 2] | 42 [25; 66] | 46 [27; 73] | 3 [1; 5] | 3 [1; 6] | 45 [27; 70] | 49 [29; 77] | 13.2 [7.8; 20.7] | 14.4 [8.6; 22.6] | 2 [1; 2] | 2 [1; 3] | 52 [25; 99] | 57 [28; 107] | 765 [423; 1,439] | 697 [386; 1,320] | 183 [84; 458] | 167 [76; 417] |
| Lower | 335 | 51% [37%; 64%] | 6 [3; 12] | 7 [3; 13] | 1,899 [834; 3,651] | 2,077 [907; 3,992] | 0 [0; 1] | 0 [0; 1] | 31 [19; 49] | 34 [20; 54] | 1 [0; 1] | 1 [0; 1] | 32 [19; 50] | 35 [21; 55] | 9.6 [5.7; 15.0] | 10.5 [6.2; 16.5] | 1 [1; 2] | 1 [1; 2] | 59 [29; 111] | 64 [31; 121] | 1,044 [575; 1,968] | 950 [524; 1,809] | 187 [85; 469] | 170 [77; 427] |
| Middle | 295 | 45% [33%; 56%] | 6 [2; 11] | 6 [3; 12] | 1,926 [846; 3,702] | 2,105 [920; 4,047] | 1 [0; 2] | 1 [0; 2] | 35 [21; 55] | 38 [23; 61] | 4 [2; 8] | 4 [2; 8] | 39 [23; 61] | 43 [25; 67] | 13.2 [7.9; 20.8] | 14.5 [8.6; 22.6] | 1 [1; 2] | 2 [1; 2] | 44 [22; 84] | 49 [24; 91] | 765 [423; 1,436] | 698 [388; 1,321] | 186 [85; 464] | 169 [77; 423] |
| Higher | 339 | 52% [38%; 64%] | 6 [3; 12] | 7 [3; 13] | 1,820 [799; 3,498] | 1,990 [869; 3,825] | 1 [0; 1] | 1 [0; 1] | 30 [18; 48] | 33 [20; 52] | 2 [1; 5] | 3 [1; 5] | 33 [20; 52] | 36 [21; 56] | 9.7 [5.8; 15.2] | 10.6 [6.3; 16.6] | 1 [1; 2] | 1 [1; 2] | 64 [31; 121] | 70 [34; 131] | 1,031 [567; 1,942] | 939 [517; 1,783] | 194 [88; 488] | 177 [80; 445] |
| Highest | 353 | 54% [39%; 67%] | 5 [2; 10] | 6 [2; 11] | 1,429 [627; 2,746] | 1,562 [683; 3,003] | 0 [0; 1] | 0 [0; 1] | 21 [13; 33] | 23 [14; 37] | 1 [0; 2] | 1 [0; 2] | 22 [13; 35] | 24 [14; 38] | 6.3 [3.7; 9.9] | 6.9 [4.1; 10.8] | 1 [0; 1] | 1 [1; 1] | 92 [45; 174] | 100 [49; 189] | 1,557 [847; 2,961] | 1,414 [768; 2,714] | 243 [108; 614] | 220 [97; 560] |
| Mashonaland West | 2,485 | 39% [29%; 49%] | 44 [19; 87] | 48 [21; 94] | 1,763 [774; 3,487] | 1,928 [843; 3,785] | 6 [3; 12] | 6 [3; 13] | 446 [265; 704] | 487 [287; 766] | 43 [18; 88] | 47 [20; 95] | 489 [292; 765] | 534 [317; 838] | 19.7 [11.8; 30.8] | 21.5 [12.8; 33.7] | 17 [10; 27] | 19 [11; 30] | 133 [64; 257] | 145 [70; 279] | 529 [297; 998] | 483 [272; 905] | 208 [94; 514] | 190 [85; 469] |
| Lowest | 487 | 40% [29%; 49%] | 10 [4; 19] | 11 [5; 21] | 1,979 [869; 3,915] | 2,164 [947; 4,250] | 1 [1; 3] | 1 [1; 3] | 104 [62; 165] | 114 [67; 179] | 10 [4; 20] | 10 [4; 21] | 114 [68; 178] | 124 [74; 195] | 23.4 [14.0; 36.6] | 25.6 [15.2; 40.1] | 4 [2; 6] | 4 [3; 7] | 9 [5; 18] | 10 [5; 20] | 448 [252; 842] | 410 [233; 765] | 187 [85; 460] | 171 [78; 420] |
| Lower | 470 | 38% [28%; 47%] | 9 [4; 18] | 10 [4; 20] | 1,938 [851; 3,833] | 2,119 [927; 4,161] | 2 [1; 3] | 2 [1; 4] | 110 [65; 174] | 120 [71; 189] | 15 [6; 30] | 16 [7; 32] | 125 [75; 195] | 136 [81; 214] | 26.6 [15.9; 41.5] | 29.0 [17.3; 45.5] | 4 [3; 7] | 5 [3; 8] | 20 [9; 38] | 21 [10; 41] | 393 [222; 740] | 359 [203; 672] | 190 [86; 468] | 174 [78; 427] |
| Middle | 445 | 36% [26%; 45%] | 8 [4; 16] | 9 [4; 18] | 1,846 [810; 3,651] | 2,019 [883; 3,964] | 1 [0; 2] | 1 [1; 2] | 98 [58; 154] | 107 [63; 168] | 8 [4; 17] | 9 [4; 19] | 106 [63; 166] | 116 [69; 181] | 23.8 [14.2; 37.3] | 26.0 [15.4; 40.8] | 4 [2; 6] | 4 [2; 6] | 7 [3; 13] | 7 [4; 14] | 441 [248; 828] | 404 [229; 754] | 201 [92; 494] | 184 [84; 451] |
| Higher | 508 | 41% [30%; 51%] | 9 [4; 17] | 9 [4; 19] | 1,699 [746; 3,361] | 1,858 [812; 3,648] | 1 [0; 2] | 1 [0; 2] | 71 [42; 113] | 78 [46; 123] | 5 [2; 10] | 5 [2; 10] | 76 [45; 119] | 83 [49; 130] | 15.0 [9.0; 23.5] | 16.4 [9.7; 25.7] | 3 [2; 4] | 3 [2; 5] | 26 [13; 51] | 29 [14; 55] | 693 [388; 1,307] | 633 [356; 1,189] | 216 [98; 534] | 198 [89; 487] |
| Highest | 575 | 47% [34%; 58%] | 8 [4; 16] | 9 [4; 18] | 1,430 [628; 2,828] | 1,563 [684; 3,070] | 1 [0; 2] | 1 [0; 2] | 62 [37; 99] | 68 [40; 107] | 6 [2; 11] | 6 [3; 12] | 68 [41; 106] | 74 [44; 117] | 11.8 [7.1; 18.5] | 12.9 [7.7; 20.3] | 2 [1; 4] | 3 [2; 4] | 71 [34; 137] | 77 [37; 149] | 863 [480; 1,642] | 787 [436; 1,489] | 252 [112; 626] | 230 [101; 572] |
| Mashonaland East | 3,142 | 47% [34%; 59%] | 55 [23; 106] | 60 [26; 115] | 1,736 [745; 3,363] | 1,898 [823; 3,667] | 6 [3; 12] | 7 [3; 13] | 559 [331; 868] | 611 [361; 963] | 44 [18; 89] | 47 [19; 96] | 602 [358; 938] | 658 [389; 1,037] | 19.2 [11.4; 29.8] | 20.9 [12.4; 33.0] | 21 [13; 33] | 23 [14; 37] | 222 [106; 421] | 243 [117; 457] | 540 [302; 1,018] | 493 [275; 942] | 211 [97; 523] | 192 [90; 480] |
| Lowest | 582 | 44% [32%; 55%] | 12 [5; 22] | 13 [5; 24] | 1,986 [852; 3,848] | 2,172 [942; 4,196] | 2 [1; 3] | 2 [1; 4] | 157 [93; 245] | 172 [102; 271] | 12 [5; 24] | 13 [5; 26] | 169 [101; 264] | 185 [109; 292] | 29.1 [17.3; 45.3] | 31.8 [18.8; 50.1] | 6 [4; 9] | 7 [4; 10] | 22 [10; 41] | 24 [11; 45] | 358 [202; 674] | 328 [184; 624] | 186 [87; 458] | 170 [80; 421] |
| Lower | 662 | 50% [37%; 62%] | 12 [5; 24] | 13 [6; 26] | 1,843 [791; 3,571] | 2,015 [874; 3,893] | 1 [0; 2] | 1 [1; 2] | 113 [67; 176] | 124 [73; 195] | 7 [3; 14] | 7 [3; 15] | 120 [71; 187] | 131 [78; 207] | 18.1 [10.8; 28.3] | 19.8 [11.7; 31.3] | 4 [3; 7] | 5 [3; 7] | 39 [19; 75] | 43 [21; 81] | 572 [321; 1,075] | 522 [293; 996] | 199 [92; 493] | 182 [85; 452] |
| Middle | 612 | 46% [34%; 58%] | 11 [5; 21] | 12 [5; 23] | 1,790 [768; 3,469] | 1,957 [849; 3,782] | 1 [0; 2] | 1 [1; 2] | 101 [60; 158] | 111 [66; 175] | 8 [3; 16] | 9 [4; 18] | 110 [65; 170] | 120 [71; 189] | 17.9 [10.6; 27.9] | 19.6 [11.6; 30.8] | 4 [2; 6] | 4 [3; 7] | 21 [10; 40] | 23 [11; 43] | 584 [329; 1,097] | 533 [299; 1,015] | 206 [96; 509] | 188 [89; 467] |
| Higher | 630 | 48% [35%; 59%] | 11 [5; 21] | 12 [5; 23] | 1,717 [737; 3,327] | 1,877 [815; 3,627] | 1 [1; 3] | 2 [1; 3] | 103 [61; 159] | 112 [66; 177] | 12 [5; 25] | 13 [5; 27] | 115 [68; 178] | 125 [74; 197] | 18.2 [10.8; 28.3] | 19.9 [11.7; 31.3] | 4 [2; 6] | 4 [3; 7] | 45 [22; 85] | 49 [24; 93] | 569 [318; 1,073] | 520 [289; 991] | 213 [98; 528] | 194 [91; 485] |
| Highest | 657 | 50% [36%; 62%] | 9 [4; 17] | 10 [4; 19] | 1,373 [589; 2,659] | 1,501 [651; 2,900] | 1 [0; 1] | 1 [0; 2] | 84 [50; 130] | 92 [54; 145] | 5 [2; 10] | 5 [2; 11] | 89 [53; 138] | 97 [57; 153] | 13.5 [8.0; 21.0] | 14.8 [8.7; 23.3] | 3 [2; 5] | 3 [2; 5] | 95 [45; 180] | 104 [50; 195] | 750 [415; 1,421] | 684 [376; 1,316] | 261 [118; 655] | 238 [109; 600] |
| Mashonaland Central | 2,268 | 45% [33%; 56%] | 42 [18; 81] | 46 [20; 88] | 1,846 [813; 3,579] | 2,018 [881; 3,879] | 5 [2; 10] | 5 [2; 11] | 419 [245; 661] | 458 [268; 716] | 36 [15; 73] | 39 [16; 79] | 455 [269; 714] | 497 [291; 776] | 20.1 [11.9; 31.5] | 21.9 [12.8; 34.2] | 16 [10; 25] | 18 [10; 27] | 103 [50; 193] | 112 [54; 211] | 519 [291; 978] | 475 [265; 894] | 199 [92; 487] | 182 [85; 442] |
| Lowest | 409 | 41% [30%; 51%] | 8 [4; 16] | 9 [4; 17] | 1,994 [878; 3,866] | 2,180 [952; 4,190] | 1 [0; 2] | 1 [0; 2] | 84 [49; 133] | 92 [54; 144] | 5 [2; 10] | 5 [2; 11] | 89 [52; 140] | 97 [57; 152] | 21.8 [12.8; 34.2] | 23.8 [14.0; 37.1] | 3 [2; 5] | 3 [2; 5] | 5 [2; 8] | 5 [2; 9] | 482 [272; 910] | 441 [248; 829] | 186 [87; 453] | 170 [80; 412] |
| Lower | 432 | 43% [32%; 54%] | 8 [4; 16] | 9 [4; 18] | 1,953 [860; 3,786] | 2,135 [933; 4,104] | 1 [1; 3] | 1 [1; 3] | 92 [54; 144] | 100 [58; 156] | 11 [4; 22] | 12 [5; 23] | 102 [60; 161] | 112 [65; 174] | 23.7 [14.0; 37.2] | 25.9 [15.2; 40.4] | 4 [2; 6] | 4 [2; 6] | 14 [7; 27] | 16 [8; 30] | 442 [248; 830] | 404 [226; 760] | 189 [88; 461] | 173 [81; 419] |
| Middle | 481 | 48% [35%; 60%] | 9 [4; 18] | 10 [4; 19] | 1,901 [837; 3,687] | 2,079 [908; 3,996] | 1 [0; 2] | 1 [1; 2] | 102 [59; 160] | 111 [65; 174] | 9 [4; 18] | 10 [4; 19] | 110 [65; 173] | 120 [71; 188] | 22.9 [13.6; 36.0] | 25.1 [14.7; 39.1] | 4 [2; 6] | 4 [2; 7] | 4 [2; 8] | 4 [2; 8] | 459 [258; 863] | 420 [236; 790] | 195 [91; 476] | 179 [84; 432] |
| Higher | 461 | 46% [34%; 57%] | 8 [4; 16] | 9 [4; 17] | 1,787 [787; 3,464] | 1,954 [853; 3,754] | 1 [0; 2] | 1 [1; 2] | 87 [51; 138] | 95 [56; 149] | 8 [3; 16] | 9 [4; 18] | 95 [56; 149] | 104 [61; 163] | 20.7 [12.2; 32.4] | 22.6 [13.2; 35.3] | 3 [2; 5] | 4 [2; 6] | 21 [10; 40] | 23 [11; 44] | 504 [282; 949] | 460 [257; 868] | 206 [96; 503] | 188 [88; 457] |
| Highest | 486 | 49% [36%; 60%] | 8 [3; 15] | 9 [4; 17] | 1,628 [717; 3,156] | 1,780 [777; 3,420] | 1 [0; 1] | 1 [0; 1] | 54 [32; 86] | 60 [35; 93] | 4 [2; 7] | 4 [2; 8] | 58 [34; 91] | 63 [37; 99] | 12.0 [7.1; 18.8] | 13.1 [7.7; 20.4] | 2 [1; 3] | 2 [1; 3] | 59 [28; 110] | 64 [31; 121] | 853 [473; 1,621] | 778 [428; 1,476] | 221 [100; 547] | 202 [92; 496] |
| Manicaland | 2,923 | 38% [28%; 47%] | 50 [21; 96] | 54 [24; 104] | 1,702 [732; 3,283] | 1,861 [805; 3,563] | 6 [3; 12] | 7 [3; 14] | 532 [316; 834] | 582 [343; 910] | 49 [20; 99] | 53 [22; 107] | 581 [346; 909] | 635 [376; 992] | 19.9 [11.8; 31.1] | 21.7 [12.9; 33.9] | 21 [12; 32] | 22 [13; 35] | 291 [142; 551] | 319 [152; 595] | 517 [284; 996] | 471 [263; 889] | 213 [98; 542] | 194 [90; 494] |
| Lowest | 509 | 35% [25%; 43%] | 9 [4; 18] | 10 [4; 20] | 1,859 [800; 3,584] | 2,032 [878; 3,890] | 2 [1; 3] | 2 [1; 3] | 139 [83; 218] | 152 [90; 238] | 13 [5; 27] | 14 [6; 29] | 152 [91; 238] | 166 [99; 260] | 29.9 [17.8; 46.8] | 32.7 [19.4; 51.0] | 5 [3; 8] | 6 [3; 9] | 33 [16; 62] | 36 [17; 67] | 347 [192; 666] | 317 [178; 596] | 197 [92; 499] | 180 [85; 455] |
| Lower | 502 | 34% [25%; 42%] | 10 [4; 19] | 11 [5; 20] | 1,916 [824; 3,695] | 2,095 [906; 4,011] | 1 [1; 3] | 2 [1; 3] | 114 [68; 178] | 124 [73; 195] | 12 [5; 23] | 13 [5; 25] | 126 [75; 196] | 137 [81; 214] | 25.0 [14.9; 39.1] | 27.3 [16.2; 42.7] | 4 [3; 7] | 5 [3; 8] | 43 [21; 82] | 47 [23; 89] | 413 [228; 794] | 377 [211; 710] | 190 [88; 482] | 173 [81; 440] |
| Middle | 562 | 38% [28%; 47%] | 10 [4; 19] | 11 [5; 21] | 1,772 [762; 3,417] | 1,937 [837; 3,708] | 1 [1; 3] | 2 [1; 3] | 107 [64; 168] | 117 [69; 183] | 11 [4; 22] | 12 [5; 24] | 118 [70; 185] | 129 [76; 201] | 21.0 [12.5; 32.8] | 22.9 [13.6; 35.8] | 4 [2; 7] | 5 [3; 7] | 34 [17; 65] | 38 [18; 70] | 495 [274; 950] | 452 [254; 850] | 207 [97; 523] | 189 [89; 478] |
| Higher | 688 | 47% [34%; 58%] | 11 [5; 22] | 12 [5; 24] | 1,648 [709; 3,179] | 1,802 [779; 3,450] | 1 [1; 3] | 1 [1; 3] | 100 [59; 156] | 109 [64; 170] | 9 [4; 19] | 10 [4; 20] | 109 [65; 170] | 119 [70; 186] | 15.8 [9.4; 24.7] | 17.3 [10.2; 27.0] | 4 [2; 6] | 4 [2; 7] | 68 [33; 129] | 74 [36; 139] | 649 [357; 1,252] | 593 [331; 1,118] | 220 [102; 560] | 201 [93; 510] |
| Highest | 661 | 45% [33%; 55%] | 9 [4; 18] | 10 [4; 20] | 1,417 [610; 2,733] | 1,550 [670; 2,967] | 1 [0; 1] | 1 [0; 1] | 73 [43; 114] | 79 [47; 124] | 4 [2; 8] | 4 [2; 8] | 77 [45; 120] | 84 [50; 131] | 11.6 [6.9; 18.1] | 12.7 [7.5; 19.8] | 3 [2; 4] | 3 [2; 5] | 113 [55; 214] | 124 [59; 231] | 868 [472; 1,688] | 791 [434; 1,507] | 251 [114; 644] | 228 [104; 588] |
|  |  |  |  |  |  |  |  |  |  |  |  |  |  |  |  |  |  |  |  |  |  |  |  |  |
